# Supplementary material for: Self-management of chronic conditions including multimorbidity in sub-Saharan Africa: A systematic and meta-synthesis review with focus on diabetes, hypertension, chronic kidney disease, and HIV
Source: PLOS Glob Public Health. 2025 Oct 9;5(10):e0003836. doi: 10.1371/journal.pgph.0003836 (PMC12510608; doi:10.1371/journal.pgph.0003836)
Supplement: S1 Table — (DOCX) [file pgph.0003836.s001.docx]

**S1 Table: Search Strategy- Key terms and MESH terms**

| MeSH Headings | Keywords |
| --- | --- |
| “self- management” OR “self- care” | "self manag*" OR "self-manag*" OR "self car*" OR Self-car*" OR "self care*" OR "self-care*" OR "self monit*" OR "self- monit*" OR "self admin*" OR "Self- admin*" OR MH "Self-Management" OR MH "Self Care+" |
| “Chronic Disease*”  OR  Comorbid OR multimorbidity  Non-communicable diseases  OR  Hypertension  OR  “Diabetes Mellitus”  OR  Human Immunodeficeincy Virus”  OR “HIV Infections”  “Chronic Kidney Disease*” | chronic illnes* OR chronic condition* OR chronic disorder* OR “long-term condition*” OR “long-term disease*” OR “undiagnosed disaeses”  multimorbid* OR "multiple syndrome*" OR "multiple condition*" OR Co?morbidit* OR Multi?morbidit* OR multiple morbidit* OR multi-morbid* OR Comorbid* OR co-morbid* OR “multiple long term condition*” OR “multiple long term disease*” OR “multiple long term illines*” OR polymorbid* OR polypathology OR pluripathology OR multipathology OR “multiple syndrome*" OR "co mobirdity"  “noncommunicable disease” OR "noncommunicable diseases" OR "non-communicable disease" OR "non-communicable diseases" OR NCD OR NCDs OR  “high blood pressure” OR hypertens* OR "heart disease" OR “congest* heart failure" OR "cardiac failure*" OR "heart failure" OR CCF OR HF OR  “diabetes type 1” OR “diabetes type 2” OR diabet* OR diabetes OR  “HIV OR “Acquired Immuno Deficeincy Syndrome” OR AIDS OR HIV+ OR “HIV Disease” OR “HIV Infection” HIV-1 OR HIV-2* OR HIV1 OR HIV2 OR HIV infect* OR Human Immune Deficiency Virus OR “Human Immuno-Deficiency Virus” OR “Human Immune-Deficiency Virus” OR HIV OR hiv-1 OR hiv-2* OR hiv1 OR hiv2 OR hiv infect* OR human immunodeficiency virus OR human immune deficiency virus OR human immuno-deficiency virus OR human immune-deficiency virus OR human immun* OR deficiency virus OR acquired immunodeficiency syndromes OR acquired immune deficiency syndrome OR acquired immuno-deficiency syndrome OR acquired immune-deficiency syndrome OR acquired immun* OR deficiency syndrome  renal insufficiency, chronic" OR chronic kidney disease |
| “Sub-Saharan Africa” OR  “Africa, Southern” OR “Africa South of the Sahara” | Sub-Saharan Africa OR Africa, Sub-Saharan OR sub?sahara* OR SSA  Angola OR Benin OR Botswana OR Burundi OR “Burkina Faso” OR Cameroon OR “Cape Verde” OR “Central African Republic” OR Chad OR Comoros OR Congo (Brazaville) OR Congo OR “Cote de Ivoire” OR Djibout OR “Equatorial Guinea” OR Eritria OR Ethiopia OR Gabon OR Gambia OR Ghana OR Guinea OR “Guinea Bissau” OR Kenya OR Lesotho OR Liberia OR Madgascar OR Malawi OR Mali OR Mauritania OR Mauritius OR Mozambique OR Namibia OR Nigeria OR Niger Rwanda OR Sierra Leone OR Senegal OR SeyShells OR “Sao Tome and Principle” OR Somalia OR “South Africa” OR Sudan OR Swaziland OR Tanzania OR Togo OR Uganda OR “Western Sahara” OR Zambia OR Zimbabwe |
