# Supplementary material for: Self-management of chronic conditions including multimorbidity in sub-Saharan Africa: A systematic and meta-synthesis review with focus on diabetes, hypertension, chronic kidney disease, and HIV
Source: PLOS Glob Public Health. 2025 Oct 9;5(10):e0003836. doi: 10.1371/journal.pgph.0003836 (PMC12510608; doi:10.1371/journal.pgph.0003836)
Supplement: S2 Table — (DOCX) [file pgph.0003836.s002.docx]

**S2_Text.doc**

**Summary of included studies (n=23)**

| **First Author** | **Year published** | **Title** | **Aim** | **Sample** | **Conditions** |
| --- | --- | --- | --- | --- | --- |
| **R. BeLue** | 2012 | A cultural lens to understanding daily experiences with type 2 diabetes self-management among clinic patients in M'bour, Senegal | To examine experiences with diabetes self-management among clinic patients residing in M’bour, Senegal, using the PEN3 model as a cultural framework. | N = 54; Mean age = 52; 65%= females; 10%= employed; Senegalese | Diabetes; comorbid hypertension (57%) |
| **E. Mendenhal** | 2015 | Diabetes care among urban women in Soweto, South Africa (SA): a qualitative study | To investigates women’s experiences with diabetes care in Soweto, South Africa | N = 27; Mean age = 59; 69% less than 12 years education; South Africa | Diabetes; comorbid hypertension (98%), depression or arthritis |
| **P. Matwa** | 2003 | Experiences and guidelines for footcare practices of patients with diabetes mellitus | To explore experiences and footcare practices of diabetic patients in rural Transkei, South Africa. | N = 15; Mean age = 48%; 53%= grade 3 education; 53%= formally or self- employed; South Africa | Diabetes |
| **E. F. Chikumbu** | 2022 | Experiences of multimorbidity in urban and rural Malawi: an interview study of burdens of treatment and lack of treatment | To explore experiences of men and women living with multimorbidity in urban and rural Malawi including their experiences of burden of treatment and examine the utility of Normalization Process Theory (NPT) and Burden of Treatment Theory (BOTT) for structuring analytical accounts of these experiences | N = 32; 53% >50 years; multiple chronic illnesses, urban and rural Malawi | Comorbid diabetes and hypertension (50%); hypertension and HIV (32%); diabetes and HIV (12%); HIV, diabetes and hypertension (6%) |
| **N. D. B. Magobe** | 2017 | Experiences of patients with hypertension at primary health care in facilitating own lifestyle change of regular physical exercise | To explore experiences of patients with hypertension regarding the facilitation of their own health-promoting lifestyle change measure of regular physical exercise | N = 20; 40%= >60 years; Soweto, SA | Hypertension |
| **A. d. G. Aikins** | 2015 | Explanatory models of diabetes in urban poor communities in Accra, Ghana | To examine explanatory models of diabetes and diabetes complications among urban poor Ghanaians living with diabetes and implications for developing secondary prevention strategies. | N = 20; above 50%= 80%; females= 70%; 70%= primary school ed; Ghana | Diabetes; comorbidities: Asthma, Gout, Hypertension |
| **M.S. Abbdulheram** | 2016 | Exploring Cultural Influences of Self-Management of Diabetes in Coastal Kenya: An Ethnography | To describe diabetes SM among the Swahili of Kenya and explore factors that affect diabetes self-management within the context of Swahili culture. | N = 30; Mean age = 53; 53% = Female; 53%= no formal education | Diabetes |
| **D. G Aikins** | 2005 | Healer shopping in Africa: new evidence from rural-urban qualitative study of Ghanaian diabetes experiences | To provide counter evidence to existing literature on healer shopping in Africa through a systematic analysis of illness practices by Ghanaians with diabetes | N = 67; Mean age = 59; 49%= 41-60; 64%= no education; Ghana | Diabetes; comorbid Hypertension (49%), prostate cancer, asthma |
| **C. Moucheraudi** | 2022 | Health behaviours and beliefs among Malawian adults taking antihypertensive medication and antiretroviral therapy: A qualitative study | To understand HIV-positive Malawian adults’ experiences with hypertension management | N = 30; Mean age= 58; 70% Female; Mean age = 58.5; Malawi | HIV; comorbid diabetes, stroke, hypertension (57%) |
| **H. Amu** | 2021 | Management of chronic non-communicable diseases in Ghana: a qualitative study using the chronic care model | To explore the practices and challenges associated with the management of CNCDs by patients and health professionals | N = 82; 69% Female; Mean age = 59; 57%= females; Ghana | Diabetes (6%), hypertension (7%), cancer, chronic kidney disease (10%), chronic obstructive pulmonary disease [COPD], asthma, sickle cell disease, glaucoma, and stroke; comorbid hypertension (24%) |
| **T. Steyly** | 2016 | Management of type 2 diabetes mellitus: Adherence challenges in environments of low socio-economic status | To explore the obstacles encountered by individuals with type 2 diabetes mellitus from an urban community regarding management of their disease. | N = 36; Mean age = 59; 42%= females; South Africa | Diabetes |
| **V. Angwenyi** | 2018 | Patients experiences of self-management and strategies for dealing with chronic conditions in rural Malawi | To explore self-management practices of patients with different chronic conditions, and their strategies to overcome care challenges in a resource constrained setting in Malawi | N = 45; Mean age = 59; 49%= 41-60 years; 64%= no education; 58%= female; Malawi | 15% comorbidity (HIV, cancer, asthma, stroke, hypertension, heart condition. |
| **E. N. Bosire (**[**23**](#_ENREF_23)**)** | 2020 | Patients’ Experiences of Comorbid HIV/AIDS and Diabetes Care and Management in Soweto, South Africa | To explore patients’ experiences seeking care for comorbid HIV and diabetes at a public tertiary hospital in Soweto, South Africa, and self-management at home. | N = 15; Mean age = 50, >50%= unemployed; >30%= no education; South Africa; 53%= females | Hypertension. Diabetes depression, arthritis. |
| **G. Mphwante** | 2021 | Perceived barriers and facilitators to diet and physical activity among adults diagnosed with type 2 diabetes in Malawi | To identify barriers, facilitators and support for diet and physical activity among adults with T2DM in Malawi | N = 39; Mean age = 58; 53%= female; >50%= primary education; multiple chronic illnesses; Ghana | Diabetes, stroke, arthritis |
| **A. I. Okurumeh** | 2022 | Type 2 diabetes mellitus patients’ lived experience at a tertiary hospital in Ekiti State, Nigeria | To explore the lived experience of patients with Type 2 diabetes mellitus attending an endocrinology clinic at a Teaching Hospital in Ekiti State, Nigeria. | N = 24; Mean age = 58; 56%= Female; 64%= tertiary education; majority 28.6%+ 60-69 years; multiple chronic illnesses; Malawi | Diabetes; hypertension (75%) |
| **L. Drown** | 2023 | Living with type 1 diabetes in Neno, Malawi: a qualitative study of self-management and experiences in care | To explore the psychosocial and economic impact of living with T1D, T1D knowledge and self-management, and facilitators and barriers to accessing care. | N = 29 (PLWT1D N = 8, caregivers = 4); Mean age = 32; Malawi | Type 1 Diabetes |
| **B. O. Ukoha-Kalu** | 2023 | A qualitative study of patients’ and carers’ perspectives on factors influencing access to hypertension care and compliance with treatment in Nigeria | To explore patients’ and carers’ perspectives on factors influencing access to hypertension care and compliance with treatment. | N = 38; 69% = female; 92.3% = formal education; Nigeria | Hypertension |
| **P. Bleah** | 2023 | “When I Don’t Have Money, I Don’t Eat”: A Critical Hermeneutic Study of Diabetes in Liberia | To explore participants’ experiences of living with diabetes | N = 10, Mean age = 52; 60%= females; 50% = primary school education; Liberia | Diabetes |
| **P. Bleah** | 2023 | 'The solution is we need to have a centre': a study on diabetes in Liberia | To explore what it is like to live with diabetes in Liberia | N = 10, Mean age = 52; 60%= females; 50% = primary school education; Liberia | Diabetes |
| **S. Tyabazeka** | 2024 | HIV self-management perceptions and experiences of students at one university in South Africa | To explore the HIV self-management perceptions and experiences of university students living with HIV in South Africa. | N = 8; Mean age = 25; 88%= female; 100%= university education; students living with HIV; South Africa | HIV |
| 1. **Alor** | 2024 | Community beliefs and practices about diabetes and their implications for the prevention and management of diabetes in Southeast Ghana | To explore community beliefs and practices about diabetes and how they affect the prevention and management of diabetes in the community. | N = 33; IDIs, Mean age = 51; 30%= primary education; Ghana | Diabetes |
| **S. Amon** | 2024 | Household economic burden of type-2 diabetes and hypertension comorbidity care in urban-poor Ghana: a mixed methods study | To explore the economic burden associated with the rising burden of type-2 diabetes (T2D) and hypertension comorbidity management, and its implications for healthcare seeking in urban Accra. | 13 FGDs, Ghana | Diabetes and hypertension |
| **E. Endrias** | 2024 | Exploring experiences and perspectives of patients on hypertension management in Southern Ethiopia: a phenomenological study | To explore the experiences and perspectives of patients with hypertension regarding their management and care at a comprehensive hospital in Southern Ethiopia. | N=14, IDIs, Mean age= 51, 29 %= no formal education, 29%= college education, Ethiopia | Hypertension |

**Abbreviations:** T2DM- Type 2 Diabetes
