# Supplementary material for: Self-management of chronic conditions including multimorbidity in sub-Saharan Africa: A systematic and meta-synthesis review with focus on diabetes, hypertension, chronic kidney disease, and HIV
Source: PLOS Glob Public Health. 2025 Oct 9;5(10):e0003836. doi: 10.1371/journal.pgph.0003836 (PMC12510608; doi:10.1371/journal.pgph.0003836)
Supplement: S3 Table — (DOCX) [file pgph.0003836.s003.docx]

**S3_Text.doc**

**CASP checklist applied to assess the quality of studies included in the meta-synthsesis review**

| **Studies** | **Clarity of statement of aims** | **Method appropriateness** | **Appropriateness of research design** | **Appropriateness of recruitment strategy** | **Appropriateness of data collection method** | **Participant/researcher relationship p between researcher and participants** | **Any ethical issues worth noting** | **Rigorousness of analysis** | **Clear statement of results** | **Value of results** | **Quality** |
| --- | --- | --- | --- | --- | --- | --- | --- | --- | --- | --- | --- |
| **BeLue et al. 2012** | Yes | Yes | Yes | Yes | Yes | Yes | Yes | Yes | Yes | Yes | Good |
| **Mendenhal and Norris 2015** | Yes | Yes | Yes | Yes | Yes | Yes | Yes | Yes | Yes | Yes | Good |
| **Matwa et al. 2003** | Yes | Yes | Yes | Yes | Yes | Yes | Yes | Yes | Yes | Yes | Good |
| **Chikumbu et al. 2022** | Yes | Yes | Yes | Yes | Yes | Yes | Yes | Yes | Yes | Yes | Good |
| **Magobe et al. 2017** | Yes | Yes | Yes | Yes | Yes | Yes | Yes | Average | Yes | Average | Good |
| **Aikins et al. 2015** | Yes | Yes | Yes | Yes | Yes | Yes | Yes | Yes | Yes | Yes | Good |
| **Abbdulheram 2016** | Yes | Yes | Yes | Yes | Yes | Yes | Yes | Yes | Yes | Yes | Good |
| **Aikins 2005** | Yes | Yes | Yes | Yes | Yes | Yes | Yes | Yes | Yes | Yes | Good |
| **Moucheraudi et al. 2021** | Yes | Yes | Yes | Yes | Yes | Yes | Yes | Yes | Yes | Yes | Good |
| **Amu et al.2021** | Yes | Yes | Yes | Yes | Yes | Yes | Yes | Yes | Yes | Yes | Good |
| **Steyly 2016** | Yes | Yes | Yes | Yes | Yes | Yes | Yes | Yes | Yes | Yes | Good |
| **Angwenyi 2018** | Yes | Yes | Yes | Yes | Yes | Yes | Yes | Yes | Yes | Yes | Good |
| **Bosire 2020** | Yes | Yes | Yes | Yes | Yes | Yes | Yes | Yes | Yes | Yes | Good |
| **Mphwante et al. 2021** | Yes | Yes | Yes | Yes | Yes | Yes | Yes | Yes | Yes | Yes | Good |
| **Okurumeh 2022** | Yes | Yes | Yes | Yes | Yes | Yes | Yes | Yes | Average | Yes | Good |
| **Ukoha-Kalu et al. 2023** | Yes | Yes | Yes | Yes | Yes | Yes | Yes | Yes | Yes | Yes | Good |
| **Drown et al. 2023** | Yes | Yes | Yes | Yes | Yes | Yes | Yes | Yes | Yes | Yes | Good |
| **Bleah et al. 2023** | Yes | Yes | Yes | Yes | Yes | Yes | Yes | Yes | Yes | Yes | Good |
| **Bleah et al. 2023** | Yes | Yes | Yes | Yes | Yes | Yes | Yes | Yes | Yes | Yes | Good |
| **Tyabazek et al. 2024** | Yes | Yes | Yes | Yes | Yes | Yes | Yes | Yes | Yes | Yes | Good |
| **Alor et al. 2024** | Yes | Yes | Yes | Yes | Yes | Yes | Yes | Yes | Yes | Yes | Good |
| **Amon et al. 2024** | Yes | Yes | Yes | Average | Average | Yes | Yes | Yes | Yes | Yes | Good |
| **Endrias et al. 2024** | Yes | Yes | Yes | Yes | Yes | Yes | Yes | Yes | Yes | Yes | Good |
