# Supplementary material for: Self-management of chronic conditions including multimorbidity in sub-Saharan Africa: A systematic and meta-synthesis review with focus on diabetes, hypertension, chronic kidney disease, and HIV
Source: PLOS Glob Public Health. 2025 Oct 9;5(10):e0003836. doi: 10.1371/journal.pgph.0003836 (PMC12510608; doi:10.1371/journal.pgph.0003836)
Supplement: S4 Table — (DOCX) [file pgph.0003836.s004.docx]

**S4 Table Search results October 2024- April 12^th^ 2025**

|  | **Author** | **Year** | **Title** | **Decision** |
| --- | --- | --- | --- | --- |
|  | A. Desclaux |  | [10 years' research in the social sciences on AIDS in Burkina Faso. Elements for prevention] | Invalid |
|  | _ | 2014 | 2008 SNRS abstracts -- D - E | Invalid |
|  | _ | 1997 | 2008 SNRS abstracts -- N - Q | Invalid |
|  | M. Simwinga, M. K. Kumwenda, R. J. Dacombe, L. Kayira, A. Muzumara, C. C. et al. | 2019 | Ability to understand and correctly follow HIV self-test kit instructions for use: applying the cognitive interview technique in Malawi and Zambia | Subject |
|  | S. A. Lippman, H. J. Gilmore, T. Lane, O. Radebe, Y.-H. Chen, N. et at. | 2018 | Ability to use oral fluid and fingerstick HIV self-testing (HIVST) among South African MSM | Subject |
|  | S. A. Lippman, H. J. Gilmore, T. Lane, O. Radebe, Y. H. Chen, N. et al. | 2018 | Ability to use oral fluid and fingerstick HIV self-testing (HIVST) among South African MSM | Repetition |
|  | _ | 2001 | Abstracts presented at the XIIIth International Conference on AIDS, Durban, South Africa, July 9-14, 2000 | Subject |
|  | P. C. Adamson, M. J. Huchko, A. M. Moss, H. F. Kinkel and A. Medina-Marino | 2015 | Acceptability and Accuracy of Cervical Cancer Screening Using a Self-Collected Tampon for HPV Messenger-RNA Testing among HIV-Infected Women in South Africa | Subject |
|  | S. Schwartz, K. Clouse, N. Yende, A. Rie, J. Bassett, M. et al. | 2015 | Acceptability and Feasibility of a Mobile Phone-Based Case Management Intervention to Retain Mothers and Infants from an Option B+ Program in Postpartum HIV Care | Subject |
|  | A. Altaf, M. S. K. Pasha, A. Majeed, W. Ali, A. S. Alaama et al. | 2022 | Acceptability and feasibility of HIV self-testing among transgender people in Larkana, Pakistan: Results from a pilot project | Subject |
|  | A. Mremi, D. S. Linde, B. McHome, J. Mlay, D. Schledermann, J. et al. | 2021 | Acceptability and feasibility of self-sampling and follow-up attendance after text message delivery of human papillomavirus results: A cross-sectional study nested in a cohort in rural Tanzania | Subject |
|  | A. Mremi, D. S. Linde, B. McHome, J. Mlay, D. Schledermann, J. Blaakær, V. et al. | 2021 | Acceptability and feasibility of self-sampling and follow-up attendance after text message delivery of human papillomavirus results: A cross-sectional study nested in a cohort in rural Tanzania | Repetition |
|  | J. Hector, M.-A. Davies, J. Dekker-Boersema, M. M. Aly, C. C. A. Abdalad, E. B. R. et al. | 2018 | Acceptability and performance of a directly assisted oral HIV self-testing intervention in adolescents in rural Mozambique | Subject |
|  | N. Tshuma, O. Mosikare, J. A. Yun, O. A. Alaba, M. S. Maheedhariah, K. et al. | 2017 | Acceptability of community-based adherence clubs among health facility staff in South Africa: a qualitative study | Subject |
|  | C. Harichund, M. Moshabela, P. Kunene and Q. Abdool Karim | 2019 | Acceptability of HIV self-testing among men and women in KwaZulu-Natal, South Africa | Subject |
|  | C. Harichund, M. Moshabela, P. Kunene and Q. A. Karim | 2019 | Acceptability of HIV self-testing among men and women in KwaZulu-Natal, South Africa | Repetition |
|  | https://www.tandfonline.com/loi/caic20 | 2015 |  | Invalid |
|  | S. Tonen-Wolyec, F.-X. Mbopi-Kéou, S. Batina-Agasa, G. C. M. Kalla, M. Noubom, R.-S. et al. | 2019 | Acceptability of HIV self-testing in African students: a cross-sectional survey in the Democratic Republic of Congo | Subject |
|  | C. Harichund and M. Moshabela | 2018 | Acceptability of HIV Self-Testing in Sub-Saharan Africa: Scoping Study | Review |
|  | J. Krause, F. Subklew-Sehume, C. Kenyon and R. Colebunders | 2013 | Acceptability of HIV self-testing: a systematic literature review | Review |
|  | O. A. Offorjebe, R. M. Hoffman, F. Shaba, K. Balakasi, D. J. Davey, M.et al. | 2020 | Acceptability of index partner HIV self-testing among HIV-positive clients in Malawi: A mixed methods analysis | Subject |
|  | A. T. Choko, M. K. Kumwenda, C. C. Johnson, D. W. Sakala, M. C. Chikalipo, et al. | 2017 | Acceptability of woman-delivered HIV self-testing to the male partner, and additional interventions: a qualitative study of antenatal care participants in Malawi | Subject |
|  | S. Tonen-Wolyec, S. Batina-Agasa, J. Muwonga, R.-S. Mboumba Bouassa, C. Kayembe Tshilumba and L. Bélec | 2019 | Acceptability, feasibility, and individual preferences of blood-based HIV self-testing in a population-based sample of adolescents in Kisangani, Democratic Republic of the Congo | Repetition |
|  | S. Tonen-Wolyec, S. Batina-Agasa, J. Muwonga, R. S. Mboumba Bouassa, C. Kayembe Tshilumba et al. | 2019 | Acceptability, feasibility, and individual preferences of blood-based HIV self-testing in a population-based sample of adolescents in Kisangani, Democratic Republic of the Congo | Repetition |
|  | S. Okoboi, A. Twimukye, O. Lazarus, B. Castelnuovo, C. Agaba et al. | 2019 | Acceptability, perceived reliability and challenges associated with distributing HIV self-test kits to young MSM in Uganda: a qualitative study | Subject |
|  | G. G. G. Donders, G. Andabati, F. Donders, T. Michiels, N. Eggermont, G. et al | 2012 | Acceptance of self-testing for increased vaginal pH in different subsets of Ugandan women | Subject |
|  | https://ijsa.rsmjournals.com/ | 2019 |  | Invalid |
|  | R. A. Matter and A. H. Eide | 2018 | Access to assistive technology in two Southern African countries | Subject |
|  | K. Y. Song and H. S. Kim | 1982 | Access to health services in remote rural areas | Subject |
|  | A. E. Kurth, C. M. Cleland, N. Chhun, J. E. Sidle, E. Were, V. et al. | 2016 | Accuracy and Acceptability of Oral Fluid HIV Self-Testing in a General Adult Population in Kenya | Subject |
|  | S. P. Choukem, C. Sih, D. Nebongo, P. Tientcheu and A. P. Kengne | 2019 | Accuracy and precision of four main glucometers used in a Sub-Saharan African Country: a cross-sectional study | Subject |
|  | S. Asiimwe, J. Oloya, X. Song and C. C. Whalen | 2014 | Accuracy of un-supervised versus provider-supervised self-administered HIV testing in Uganda: A randomized implementation trial | Subject |
|  | A. Gebrekidan, G. Gill, D. Wile and S. Tesfaye | 2004 | An accurate and portable system for glycated haemoglobin measurement in the tropics | Subject |
|  | S. Ansari, H. Hosseinzadeh, S. Dennis and N. Zwar | 2020 | Activating primary care COPD patients with multi-morbidity through tailored self-management support | Subject |
|  | J. van der Pijl, J. M. Wilmshurst, M. van Dijk, A. Argent, J. Booth et al. | 2018 | Acute flaccid paralysis in South African children: Causes, respiratory complications and neurological outcome | Subject |
|  | N. Khumsaen and R. Stephenson | 2017 | Adaptation of the HIV/AIDS self-management education program for men who have sex with men in Thailand: an application of the ADAPT-ITT framework | Subject |
|  | S. Ansari, H. Hosseinzadeh, S. Dennis and N. Zwar | 2020 | Activating primary care COPD patients with multi-morbidity through tailored self-management support |  |
|  |  |  |  |  |
|  |  |  |  |  |
|  |  |  |  |  |
|  | https://www.guilford.com | 2018 |  | Invalid |
|  | N. P. Sematlane, L. Knight, C. Masquillier and E. Wouters | 2021 | Adapting to, integrating and self-managing HIV as a chronic illness: a scoping review protocol | Protocol |
|  | https://bmjopen.bmj.com/content/11/6/e047870 | 2021 |  | Invalid |
|  | C. M. Celano, C. Massey, J. Long, S. Kim, O. Velasquez, B. C. et al. | 2021 | An Adaptive, Algorithm-based Text Message Intervention to Promote Health Behavior Adherence in Type 2 Diabetes: Treatment Development and Proof-of-Concept Trial | Subject |
|  | A. Banbury, S. Nancarrow, J. Dart, L. Gray, S. Dodson, R. et al. | 2020 | Adding value to remote monitoring: Co-design of a health literacy intervention for older people with chronic disease delivered by telehealth - The telehealth literacy project | Subject |
|  | A. Walcott-Bryant, W. Ogallo, S. L. Remy, K. Tryon, W. Shena et al. | 2021 | Addressing Care Continuity and Quality Challenges in the Management of Hypertension: Case Study of the Private Health Care Sector in Kenya | Subject |
|  | V. Patel, D. Chisholm, R. Parikh, F. J. Charlson, L. Degenhardt, et al. | 2016 | Addressing the burden of mental, neurological, and substance use disorders: key messages from Disease Control Priorities, 3rd edition | Subject |
|  | V. Mogre, Z. O. Abanga, F. Tzelepis, N. A. Johnson and C. Paul | 2017 | Adherence to and factors associated with self-care behaviours in type 2 diabetes patients in Ghana | Methods |
|  | J. Bagonza, E. Rutebemberwa and W. Bazeyo | 2015 | Adherence to anti diabetic medication among patients with diabetes in eastern Uganda; a cross sectional study | Methods |
|  | K. B. Yusuff, O. Obe and B. Y. Joseph | 2008 | Adherence to anti-diabetic drug therapy and self management practices among type-2 diabetics in Nigeria | Methods |
|  | E. Agidew, M. Z. Wale, H. Kerebih, M. T. Yirsaw, T. H. Zewdie et al. | 2021 | Adherence to diabetes self-care management and associated factors among people with diabetes in Gamo Gofa Zone public health hospitals | Methods |
|  | Y. Geneti, K. Wondwossen, M. Adimasu, D. Deressa, F. Aga, M. et al. | 2022 | Adherence to Diabetes Self-Management and Its Associated Factors Among Adolescents Living with Type 1 Diabetes at Public Hospitals in Addis Ababa, Ethiopia: A Cross-Sectional Study | Population |
|  | Z. Bonger, S. Shiferaw and E. Z. Tariku | 2018 | Adherence to diabetic self-care practices and its associated factors among patients with type 2 diabetes in Addis Ababa, Ethiopia | Methods |
|  | N. Tarantino, A. Lowery and L. K. Brown | 2020 | Adherence to HIV Care and Associated Health Functioning among Youth Living with HIV in Sub-Saharan Africa | Population |
|  | T. Mariye, A. Girmay, T. Birhanu, H. Tasew, G. Teklay, Z. et al. | 2019 | Adherence to insulin therapy and associated factors among patients with diabetes mellitus in public hospitals of Central Zone of Tigray, Ethiopia, 2018: a cross-sectional study | Methods |
|  | S. P. Bruce, F. Acheampong and I. Kretchy | 2015 | Adherence to oral anti-diabetic drugs among patients attending a Ghanaian teaching hospital | Methods |
|  | A. Tibebu, D. Mengistu and L. N. Bulto | 2017 | Adherence to prescribed antihypertensive medications and associated factors for hypertensive patients attending chronic follow-up units of selected public hospitals in Addis Ababa, Ethiopia | Methods |
|  | A. Tibebu, D. Mengistu and L. Negesa | 2017 | Adherence to recommended lifestyle modifications and factors associated for hypertensive patients attending chronic follow-up units of selected public hospitals in Addis Ababa, Ethiopia | Repetition |
|  | F. Nackers, H. Huerga, E. Espié, A. O. Aloo, M. Bastard, J. et al. | 2012 | Adherence to self-administered tuberculosis treatment in a high HIV-prevalence setting: a cross-sectional survey in Homa Bay, Kenya | Subject |
|  | F. Nackers, H. Huerga, E. Espié, A. O. Aloo, M. Bastard, J. et al. | 2021 | Adherence to Self-Administered Tuberculosis Treatment in a High HIV-Prevalence Setting: A Cross-Sectional Survey in Homa Bay, Kenya | Subject |
|  | V. Mogre, N. A. Johnson, F. Tzelepis, J. Shaw and C. Paul | 2017 | Adherence to self-care behaviours and associated barriers in type 2 diabetes patients of low-and middle-income countries: a systematic review protocol | Protocol |
|  | B. W. Tegegn, W. Y. Hussien, A. E. Abebe and M. W. S. Gebre | 2021 | Adherence to Self-Care Practices and Associated Factors Among Outpatient Adult Heart Failure Patients Attending a Cardiac Center in Addis Ababa, Ethiopia in 2020 | Subject |
|  | A. Baymot, D. Gela and T. Bedada | 2022 | Adherence to self-care recommendations and associated factors among adult heart failure patients in public hospitals, Addis Ababa, Ethiopia, 2021: cross-sectional study | Subject |
|  | B. Molla, H. A. Geletie, G. Alem, T. Gualu, B. T. Zewudie, S. et al. | 2022 | Adherence to Self-Care Recommendations and Associated Factors among Adult Heart Failure Patients in West Gojjam Zone Public Hospitals, Northwest Ethiopia | Subject |
|  | M. A. Seid, O. A. Abdela and E. G. Zeleke | 2019 | Adherence to self-care recommendations and associated factors among adult heart failure patients. From the patients' point of view | Subject |
|  | N. A. Sam-Agudu, J. R. Pharr, T. Bruno, C. L. Cross, L. J. Cornelius, P. et al. | 2017 | Adolescent Coordinated Transition (ACT) to improve health outcomes among young people living with HIV in Nigeria: study protocol for a randomized controlled trial | Subject |
|  | G. Mburu, I. Hodgson, S. Kalibala, C. Haamujompa, F. Cataldo, E. et al. | 2014 | Adolescent HIV disclosure in Zambia: barriers, facilitators and outcomes | Population |
|  | T. Crowley, A. van der Merwe and D. Skinner | 2019 | Adolescent HIV Self-management: Lived Experiences of Adolescents, Caregivers, and Health Care Workers in a South African Context | Population |
|  | T. Crowley, A. v. d. Merwe, M. Kidd and D. Skinner | 2020 | Adolescent human immunodeficiency virus self-management: associations with treatment adherence, viral suppression, sexual risk behaviours and health-related quality of life | Subject |
|  | https://sajhivmed.org.za/index.php/hivmed/article/view/1054 | 2022 |  | Population |
|  | T. Crowley, A. van der Merwe, M. Kidd and D. Skinner | 2020 | Adolescent human immunodeficiency virus self-management: Associations with treatment adherence, viral suppression, sexual risk behaviours and health-related quality of life | Population |
|  | L. Adams and T. Crowley | 2021 | Adolescent Human immunodeficiency virus self-management: needs of adolescents in the Eastern Cape | Population |
|  | Adolescent human immunodeficiency virus self-management: Needs of adolescents in the Eastern Cape | 2020 | Adolescent human immunodeficiency virus self-management: Needs of adolescents in the Eastern Cape | Population |
|  | L. Prentice Hoogervorst and P. M. Mayers | 2022 | Adolescent paediatric transplant patients' experiences of transition to adult services | Subject |
|  | L. Prentice-Hoogervorst and P. M. Mayers | 2022 | Adolescent paediatric transplant patients' experiences of transition to adult services | Repetition |
|  | I. N. Njuguna, K. Beima-Sofie, C. W. Mburu, C. Mugo, J. Neary, J. et al. | 2020 | Adolescent transition to adult care for HIV-infected adolescents in Kenya (ATTACH): study protocol for a hybrid effectiveness-implementation cluster randomised trial | Protocol |
|  | I. N. Njuguna, K. Beima-Sofie, C. W. Mburu, C. Mugo, J. Neary, J. et al. | 2015 | Adolescent transition to adult care for HIV-infected adolescents in Kenya (ATTACH): study protocol for a hybrid effectiveness-implementation cluster randomised trial | Protocol |
|  | I. N. Njuguna, K. Beima-Sofie, C. W. Mburu, C. Mugo, J. Neary, J. et al. | 2020 | Adolescent transition to adult care for HIV-infected adolescents in Kenya (ATTACH): study protocol for a hybrid effectiveness-implementation cluster randomised trial | Protocol |
|  | W. Mavhu, K. Hatzold, K. H. Dam, M. R. Kaufman, E. U. Patel, L. M. et al. | 2017 | Adolescent Wound-Care Self-Efficacy and Practices After Voluntary Medical Male Circumcision-A Multicountry Assessment | Subject |
|  | E. A. Abrams, V. M. Burke, K. G. Merrill, C. Frimpong, S. Miti, J. et al. | 2021 | Adolescents do not only require ARVs and adherence counseling: a qualitative investigation of health care provider experiences with an HIV youth peer mentoring program in Ndola, Zambia | Population |
|  | E. A. Abrams, V. M. Burke, K. G. Merrill, C. Frimpong, S. Miti, J. et al. | 2017 | Adolescents do not only require ARVs and adherence counseling: a qualitative investigation of health care provider experiences with an HIV youth peer mentoring program in Ndola, Zambia | Repetition |
|  | S. Okawa, S. Mwanza-Kabaghe, M. Mwiya, K. Kikuchi, M. Jimba, C. et al. | 2017 | Adolescents' Experiences and Their Suggestions for HIV Serostatus Disclosure in Zambia: A Mixed-Methods Study | Population |
|  | M. J. Boivin, M. Chernoff, L. Fairlie, B. Laughton, B. Zimmer, C. et al. | 2019 | African multi-site 2-year neuropsychological study of school-age children perinatally infected, exposed, and unexposed to human immunodeficiency virus | Subject |
|  | M. J. Boivin, M. Chernoff, L. Fairlie, B. Laughton, B. Zimmer, C. et al. | 2022 | African Multi-Site 2-Year Neuropsychological Study of School-Age Children Perinatally Infected, Exposed, and Unexposed to Human Immunodeficiency Virus | Repetition |
|  | M. J. Boivin, M. Chernoff, L. Fairlie, B. Laughton, B. Zimmer, C. et al. | 2020 | African Multi-Site 2-Year Neuropsychological Study of School-Age Children Perinatally Infected, Exposed, and Unexposed to Human Immunodeficiency Virus | Repetition |
|  | P. Sarma, R. Cassidy, S. Corlett and B. Katusiime | 2023 | Ageing with HIV: Medicine Optimisation Challenges and Support Needs for Older People Living with HIV: A Systematic Review | Review |
|  | K. F. Norr, B. J. McElmurry, M. Moeti and S. D. Tlou | 1992 | AIDS prevention for women: a community-based approach | Subject |
|  | K. Henry | 1995 | AIDSCAP seeks a private sector solution to the STD self-treatment dilemma | Subject |
|  | J. Bousquet, P. W. Hellings, I. Agache, F. Amat, I. Annesi-Maesano, I. J. et al. | 2019 | Allergic Rhinitis and its Impact on Asthma (ARIA) Phase 4 (2018): Change management in allergic rhinitis and asthma multimorbidity using mobile technology | Subject |
|  | R. Janssen, N. Engel, A. Esmail, S. Oelofse, A. Krumeich, K. Dheda and N. P. Pai | 2020 | Alone But Supported: A Qualitative Study of an HIV Self-testing App in an Observational Cohort Study in South Africa | Subject |
|  | H. Madden, J. Harris, C. Blickem, R. Harrison and H. Timpson | 2019 | Always paracetamol, they give them paracetamol for everything: a qualitative study examining Eastern European migrants' experiences of the UK health service | Subject |
|  | K. Langwell, C. Keene, M. Zullo and L. C. Ogu | 2020 | An American Indian community implements the chronic care model: evolution and lessons learned | Subject |
|  | https://hpp.sagepub.com/content/by/year | 2020 |  | Invalid |
|  | M. Graven, H. Venkat and L. Bowie | 2022 | Analysing the citizenship agenda in Mathematical Literacy school exit assessments | Subject |
|  | L. Wang, P. Qin, J. Lin, M. Zhan, G. Ren and B. Qu | 2018 | Analysis of chronic disease prevention literacy among primary and secondary school students in Dalian | Subject |
|  | X. Zhang, H. Li, H. Shi and S. Sha | 2016 | Analysis of demand and utilization on the community nursing of the elderly, Xuzhou | Subject |
|  | B. Ma, X. Zhu and Q. Sun | 2018 | Analysis on death causes in floating population in Jinshan district of Shanghai, 2008-2016 | Subject |
|  | Y. Meng, K. Zheng, M. Li, W. Xu, L. Li and Y. Li | 2015 | Analysis on status and demand of home-based elderly care in Baoshan District of Shanghai | Subject |
|  | Y. Bai, X. Cai, S. Jin, D. Mao, Q. Liu, C. et al. | 2011 | Analysis on the early control and prevention of diabetes nephropathy in China | Subject |
|  | Y. Bai, X. Cai, Z. Wang and F. Wu | 2012 | Analysis on the knowledge, attitude and behavior of diabetes nephropathy related early control and prevention in four cities in China | Subject |
|  | E. A. Kelvin, G. George, S. Kinyanjui, E. Mwai, M. L. Romo, F. et al. | 2019 | Announcing the availability of oral HIV self-test kits via text message to increase HIV testing among hard-to-reach truckers in Kenya: a randomized controlled trial | Subject |
|  | J. O. Fadare and I. Tamuno | 2011 | Antibiotic self-medication among university medical undergraduates in Northern Nigeria | Subject |
|  | A. N. H. Bulabula, A. Dramowski and S. Mehtar | 2020 | Antibiotic use in pregnancy: knowledge, attitudes and practices among pregnant women in Cape Town, South Africa | Subject |
|  | Y. A. Adu-Sarkodie | 1997 | Antimicrobial self medication in patients attending a sexually transmitted diseases clinic | Subject |
|  | S. Russell, F. Zalwango, S. Namukwaya, J. Katongole, R. Muhumuza, R. et al. | 2015 | Antiretroviral therapy and changing patterns of HIV stigmatisation in Entebbe, Uganda | Subject |
|  | https://onlinelibrary.wiley.com/doi/10.1111/1467-9566.12341/full | 2015 | Antiretroviral therapy and changing patterns of HIV stigmatisation in Entebbe, Uganda | Invalid |
|  | X. Cheng, F. Jin and T. Wu | 2019 | Application of family-based self-management in patients with type 2 diabetes | Setting |
|  | R. M. Ansari, M. F. Harris, H. Hosseinzadeh and N. Zwar | 2021 | Applications of a Chronic Care Model for Self-Management of Type 2 Diabetes: A Qualitative Analysis | Setting |
|  | L. Tsamlag, H. Wang, Q. Shen, Y. Shi, S. Zhang, R. et al. | 2020 | Applying the information–motivation– behavioral model to explore the influencing factors of self-management behavior among osteoporosis patients | Subject |
|  | Tsamlag, L., Wang, H., Shen, Q. et al. | 2023 | Applying the information-motivation- behavioral model to explore the influencing factors of self-management behaviour among osteoporosis patients | Invalid |
|  | E. L. Sibanda, M. d'Elbée, G. Maringwa, N. Ruhode, M. Tumushime, C. et al. | 2019 | Applying user preferences to optimize the contribution of HIV self-testing to reaching the "first 90" target of UNAIDS Fast-track strategy: results from discrete choice experiments in Zimbabwe | Subject |
|  | T. Decroo, W. v. Damme, G. Kegels, D. Remartinez and F. Rasschaert | 2012 | Are Expert Patients an Untapped Resource for ART Provision in Sub-Saharan Africa? | Subject |
|  | T. Decroo, W. Van Damme, G. Kegels, D. Remartinez and F. Rasschaert | 2012 | Are Expert Patients an Untapped Resource for ART Provision in Sub-Saharan Africa? | Repetition |
|  | E. B. Malembaka, H. Karemere, G. B. Balaluka, S. M. Lwamushi, R. B. Nshobole et al. | 2020 | Are people most in need utilizing health facilities in post-conflict settings? A cross-sectional study from south Kivu, eastern DR Congo | Subject |
|  | E. B. Malembaka, H. Karemere, G. B. Balaluka, S. M. Lwamushi, R. B. Nshobole et al. | 1995 | Are people most in need utilising health facilities in post-conflict settings? A cross-sectional study from South Kivu, eastern DR Congo | Invalid |
|  | A. Spyrelis, S. Abdulla, S. Frade, T. Meyer, M. Mhazo, et al. | 2017 | Are women more likely to self-test? A short report from an acceptability study of the HIV self-testing kit in South Africa | Subject |
|  | https://www.tandfonline.com/loi/caic20 | 2021 |  | Invalid |
|  | M. S. Sever, R. Vanholder, V. Luyckx, K.-U. Eckardt, M. Kolesnyk, et al. | 2022 | Armed conflicts and kidney patients: a consensus statement from the Renal Disaster Relief Task Force of the ERA | Subject |
|  | R. T. Lester | 2013 | Ask, don't tell - mobile phones to improve HIV care | Subject |
|  | L. L. Lim, E. S. H. Lau, A. P. S. Kong, M. J. Davies, N. S. Levitt, et al. | 2018 | Aspects of Multicomponent Integrated Care Promote Sustained Improvement in Surrogate Clinical Outcomes: A Systematic Review and Meta-analysis | Subject |
|  | B. J. Miller | 2011 | Assessing diabetes in rural Tanzania with the community readiness model | Subject |
|  | D. J. T. Campbell, M. Tonelli, B. Hemmelgarn, C. Mitchell, R. Tsuyuki,et al. | 2016 | Assessing outcomes of enhanced chronic disease care through patient education and a value-based formulary study (ACCESS)-study protocol for a 2×2 factorial randomized trial | Protocol |
|  | I. Okoronkwo, S. Ishaku, A. Chinweuba, P. Akpan-Idiok, C. Ihudiebube et al. | 2015 | Assessing self care practices of people living with AIDS attending antiretroviral clinic Kafanchan, Kaduna State, Nigeria | Methods |
|  | J. Daniels, T. Lane, H. Struthers, K. Maleke, W. Moges et al. | 2017 | Assessing the feasibility of smartphone apps for HIV-care research with MSM and transgender individuals in Mpumalanga, South Africa | Subject |
|  | https://journals.sagepub.com/loi/jia | 2011 |  | Invalid |
|  | P. M. Amegbor | 2017 | An assessment of care-seeking behavior in Asikuma-Odoben-Brakwa District: a triple pluralistic health sector approach | Subject |
|  | Y. Bahru and J. Abdulkadir | 1993 | Assessment of diabetes education in the teaching hospital, Addis Ababa, Ethiopia | Subject |
|  | O. C. Okoye and O. A. Ohenhen | 2021 | Assessment of diabetes self-management amongst Nigerians using the diabetes self-management questionnaire: a cross-sectional study | Subject |
|  | E. A. Abioye-Kuteyi, E. O. Ojofeitimi, K. T. Ijadunola and A. O. Fasanu | 2005 | Assessment of dietary knowledge, practices and control in type 2 diabetes in a Nigerian teaching hospital | Subject |
|  | H. Myezwa, A. Stewart, E. Musenge and P. Nesara | 2009 | Assessment of HIV-positive in-patients using the International Classification of Functioning, Disability and Health (ICF) at Chris Hani Baragwanath Hospital, Johannesburg | Subject |
|  | S. Toujani, M. Mjid, H. Daghfous, M. Tiakhane, A. B. Younes et.al. | 1997 | Assessment of knowledge about asthma and allergic rhinitis among patients living in the Maghreb and sub-Saharan Africa | Subject |
|  | S. Toujani, M. Mjid, H. Daghfous, M. Tiakhane, A. Ben Younes, F. Amon Tanoh Dick, et al. | 2018 | Assessment of knowledge about asthma and allergic rhinitis among patients living in the Maghreb and sub-Saharan Africa | Invalid |
|  | U. Ezuruike and J. M. Prieto | 2016 | Assessment of Potential Herb-Drug Interactions among Nigerian Adults with Type-2 Diabetes | Subject |
|  | C. B. Kabeza, L. Harst, P. E. H. Schwarz and P. Timpel | 2019 | Assessment of Rwandan diabetic patients' needs and expectations to develop their first diabetes self-management smartphone application (Kir'App) | Subject |
|  | S. T. Gemeda and Z. B. Woldemariam | 2022 | Assessment of self-care practice amongst patients with type II diabetes attending Adama Hospital Medical College, Ethiopia | Methods |
|  | R. Aronson, R. E. Brown, D. Jiandani, A. Walker, N. Orzech et al. | 2018 | Assessment of self-management in patients with diabetes using the novel LMC Skills, Confidence and Preparedness Index (SCPI) | Subject |
|  | S. Suleman, A. Ketsela and Z. Mekonnen | 2009 | Assessment of self-medication practices in Assendabo town, Jimma zone, southwestern Ethiopia | Subject |
|  | V. Cambiano, D. Ford, T. Mabugu, S. Napierala Mavedzenge, A. et. al. | 2015 | Assessment of the Potential Impact and Cost-effectiveness of Self-Testing for HIV in Low-Income Countries | Subject |
|  | G. Gebremichael Lemlem, A. Abdurahman Amir and P. Gomathi | 2014 | Assessment on knowledge of diabetic patients on their disease and therapeutic goal at Ayder Referral Hospital, Mekelle, Ethiopa | Subject |
|  | I. A. Kretchy, A. Koduah, T. Ohene-Agyei, V. Boima and B. Appiah | 2020 | The Association between Diabetes-Related Distress and Medication Adherence in Adult Patients with Type 2 Diabetes Mellitus: A Cross-Sectional Study | Subject |
|  | F. Zhao, R. Suhonen, J. Katajisto, M. Stolt and H. Leino-Kilpi | 2019 | Association between diabetes-related self-care activities and positive health: a cross-sectional study | Subject |
|  | F.-F. Zhao, R. Suhonen, J. Katajisto, M. Stolt and H. Leino-Kilpi | 2019 | Association between diabetes-related self-care activities and positive health: a cross-sectional study | Repetition |
|  | D. Getenet, W. Fasil, M. Henok, B. Amare, N. Ayenew, G. M. et al. | 2020 | Association between the level of reported good medication adherence and the geographic location of a patient's residence and presence of a glucometer among adult patients with diabetes in Ethiopia: a systematic and meta-analysis | Subject |
|  | D. Getenet, W. Fasil, M. Henok, B. Amare, N. Ayenew, G. M. et al. | 2020 | Association Between the Level of Reported Good Medication Adherence and the Geographic Location of a Patient's Residence and Presence of a Glucometer Among Adult Patients with Diabetes in Ethiopia: A Systematic and Meta-Analysis 2012 | Subject |
|  | P. Braitstein, A. DeLong, D. Ayuku, M. Ott, L. Atwoli et al. | 2021 | Association of Care Environment With HIV Incidence and Death Among Orphaned, Separated, and Street-Connected Children and Adolescents in Western Kenya | Subject |
|  | F.-F. Zhao, R. Suhonen, J. Katajisto and H. Leino-Kilpi | 2018 | The association of diabetes-related self-care activities with perceived stress, anxiety, and fatigue: a cross-sectional study | Subject |
|  | N. A. Nazu, K. Wikström, M.-L. Lamidi, J. Lindström et al. | 2020 | Association of mental disorders and quality of diabetes care - A six-year follow-up study of type 2 diabetes patients in North Karelia, Finland | Subject |
|  | I. S. Okosun, R. S. Cooper, C. N. Rotimi, B. Osotimehin, T. Forrester et al. | 1998 | Association of waist circumference with risk of hypertension and type 2 diabetes in Nigerians, Jamaicans, and African-Americans | Subject |
|  | L. C. Hart, M. A. L. Tilburg, R. Campbell, R. A. Faldowski, M. Nazareth et al. | 2019 | Association of youth health care transition readiness to role overload among parents of children with chronic illness | Subject |
|  | L. C. Hart, M. A. L. Tilburg, R. Campbell, R. A. Faldowski, M. Nazareth et al. | 2019 | Association of youth health care transition readiness to role overload among parents of children with chronic illness | Repetition |
|  | X. Wang, X. Hu, Z. Liu and H. Hui | 2022 | Associations between self-test and pre- or post-exposure prophylaxis of HIV among men who have sex with men in China | Setting |
|  | X. Wang, X. Hu, Z. Liu and H. Hui | 2022 | Associations between self-test and pre- or post-exposure prophylaxis of HIV among men who have sex with men in China | Repetition |
|  | E. Oken, Y. Ning, S. L. Rifas-Shiman, J. S. Radesky, J. W. Rich-Edwards et al. | 2006 | Associations of physical activity and inactivity before and during pregnancy with glucose tolerance | Subject |
|  | E. Oken, Y. Ning, S. L. Rifas-Shiman, J. S. Radesky, J. W. Rich-Edwards et al. | 2006 | Associations of physical activity and inactivity before and during pregnancy with glucose tolerance | Repetition |
|  | koka O, Munthali-Mkandawire S, Mwandira K, Nindi P, Dube A, Nyanjagha I, et al | 2024 | Association between physical multimorbidity and common mental health disorders in rural and urban Malawian settings: Preliminary findings from Healthy Lives Malawi long-term conditions survey. | Subject |
|  | L. F. Zheng, Y. L. E. Koh, U. Sankari and N. C. Tan | 2019 | Asthma care based on Chronic Care Model in an aging Asian community | Subject |
|  | B. Tolesa, G. Alem, K. Muhammedawel and A. Kemal | 2015 | Attitude, reporting behaviour and management practice of occupational needle stick and sharps injuries among hospital healthcare workers in Bale zone, Southeast Ethiopia: a cross-sectional study | Population |
|  | V. Mogre, N. A. Johnson, F. Tzelepis and C. Paul | 2019 | Attitudes towards, facilitators and barriers to the provision of diabetes self-care support: A qualitative study among healthcare providers in Ghana | Population |
|  | L. Dube, S. v. d. Broucke, W. Dhoore, K. Kalweit and M. Housiaux | 2015 | An audit of diabetes self-management education programs in South Africa | Subject |
|  | L. Dube, S. Van den Broucke, W. Dhoore, K. Kalweit and M. Housiaux | 2015 | An Audit of Diabetes Self-Management Education Programs in South Africa | Repetition |
|  | M. R. Kaminski, J. Golledge, J. W. J. Lasschuit, K.-H. Schott, J. Charleset al. | 2022 | Australian guideline on prevention of foot ulceration: part of the 2021 Australian evidence-based guidelines for diabetes-related foot disease | Setting |
|  | T. Nóbrega Mendes, É. L. da Silva Cardoso, N. T. de Araújo Tiburtino Neves, Y. Balduíno de Araújo et al. | 1993 | Avaliação do funcionamento familiar de crianças e adolescentes com doença crônica | Population |
|  | X. Zhong, C. Tanasugarn, E. B. Fisher, S. Krudsood and D. Nityasuddhi | 2011 | Awareness and practices of self-management and influence factors among individuals with type 2 diabetes in urban community settings in Anhui Province, China | Setting |
|  | U. Abaraogu, E. Ezenwankwo, P. Dall, G. Tew, W. Stuart et al. | 2018 | Barriers and enablers to walking in individuals with intermittent claudication: A systematic review to conceptualize a relevant and patient-centered program | Subject |
|  | D. E. McMahon, R. Singh, L. Chemtai, A. Semeere, H. Byakwaga, M. et al. | 2022 | Barriers and facilitators to chemotherapy initiation and adherence for patients with HIV-associated Kaposi's sarcoma in Kenya: a qualitative study | Subject |
|  | M. Mphande, P. Campbell, R. M. Hoffman, K. Phiri, M. Nyirenda et al. | 2021 | Barriers and facilitators to facility HIV self-testing in outpatient settings in Malawi: a qualitative study | Subject |
|  | M. Mphande, P. Campbell, R. M. Hoffman, K. Phiri, M. Nyirenda et al. | 2021 | Barriers and facilitators to facility HIV self-testing in outpatient settings in Malawi: a qualitative study | Subject |
|  | S. Albeik, S. Saleh, Y. Turki, A. Mousa, N. A. Kishk, M. Tan, Y. Shahin and A. Seita | 2022 | Barriers and solutions to implementing a multisectoral action plan to prevent and control non-communicable diseases within UNRWA settings: a mixed-methods study | Subject |
|  | H. Bekele, A. Asefa, B. Getachew and A. M. Belete | 2020 | Barriers and Strategies to Lifestyle and Dietary Pattern Interventions for Prevention and Management of TYPE-2 Diabetes in Africa, Systematic Review | Review |
|  | S. Letta, F. Aga, T. A. Yadeta, B. Geda and Y. Dessie | 2021 | Barriers to Diabetes Patients' Self-Care Practices in Eastern Ethiopia: A Qualitative Study from the Health Care Providers Perspective | Subject |
|  | V. Mogre, N. A. Johnson, F. Tzelepis and C. Paul | 2019 | Barriers to diabetic self-care: A qualitative study of patients' and healthcare providers' perspectives | Population |
|  | V. Mogre, N. A. Johnson, F. Tzelepis and C. Paul | 2017 | Barriers to diabetic self‐care: A qualitative study of patients' and healthcare providers' perspectives | Repetition |
|  | I. Etuk, A. Iwuala, K. Njoku, B. Olagbegi, A. Ogboye et al. | 2023 | Barriers to health in women of reproductive age living with or at risk of non-communicable diseases in Nigeria: a Photovoice study | Subject |
|  | E. Johnson, P. B. Bwititi and E. U. Nwose | 2020 | Barriers to management of diabetes foot ulcer: Experiential note from a setting with free medical services | Subject |
|  | M. Bopape, T. Mothiba, M. Mutambudzi, J. Wens and H. Bastiaens | 2019 | Baseline assessment of knowledge of home based carers for people with diabetes in a rural village in South Africa: a quantitative study | Methods |
|  | C. Lanyon, J. Seeley, S. Namukwaya, V. Musiime, S. Paparini et al. | 2020 | Because we all have to grow up: supporting adolescents in Uganda to develop core competencies to transition towards managing their HIV more independently | Subject |
|  | J. Wuest, P. K. Ericson and P. N. Stern | 1994 | Becoming strangers: the changing family caregiving relationship in Alzheimer's disease | Subject |
|  | S. W. M. Cheng, J. Alison, S. Dennis, E. Stamatakis, L. Spencer et al. | 2017 | A behaviour change intervention to reduce sedentary time in people with chronic obstructive pulmonary disease: protocol for a randomised controlled trial | Subject |
|  | G. A. Riley and D. Baah-Odoom | 2012 | Belief in a just world, generalised self-efficacy and stigma may contribute to unsafe sexual intentions via a reduced perception of vulnerability to HIV/AIDS amongst young people in Ghana | Subject |
|  | K. Hjelm, K. Bard, K. Berntorp and J. Apelqvist | 2009 | Beliefs about health and illness postpartum in women born in Sweden and the Middle East | Setting |
|  | K. Hjelm and G. Nambozi | 2008 | Beliefs about health and illness: a comparison between Ugandan men and women living with diabetes mellitus | Methods |
|  | H. Shilubane, L. Netshikweta and T. Ralineba | 2016 | Beliefs and practices of diabetic patients in Vhembe district of Limpopo Province | Methods |
|  | L. T. Quach, C. S. Ritchie, A. C. Tsai, Z. Reynolds, R. Paul et al. | 2022 | The benefits of care: treated HIV infection and health-related quality of life among older-aged people in Uganda | Subject |
|  | A. Bakiewicz, V. Rasch, J. Mwaiselage and D. S. Linde | 2020 | The best thing is that you are doing it for yourself - perspectives on acceptability and feasibility of HPV self-sampling among cervical cancer screening clients in Tanzania: a qualitative pilot study | Subject |
|  | A. C. Mooney, S. B. Shade, C. K. Campbell, E. Agnew, H. Gilmore et al. | 2018 | Beyond Social Desirability Bias: Investigating Inconsistencies in Self-Reported HIV Testing and Treatment Behaviors Among HIV-Positive Adults in North West Province, South Africa | Subject |
|  | M. B. Krawinkel, C. Ludwig, M. E. Swai, R. Yang, K. Chun and S. D. Habicht | 2018 | Bitter gourd reduces elevated fasting plasma glucose levels in an intervention study among prediabetics in Tanzania | Subject |
|  | Krawinkel MB, Ludwig C, Swai ME, Yang RY, Chun KP, Habicht SD | 2022 | Bitter gourd reduces elevated fasting plasma glucose levels in an intervention study among prediabetics in Tanzania | Invalid |
|  | M. B. Krawinkel, C. Ludwig, M. E. Swai, R.-y. Yang, K. P. Chun and S. D. Habicht | 2018 | Bitter gourd reduces elevated fasting plasma glucose levels in an intervention study among prediabetics in Tanzania | Repetition |
|  | M. B. Krawinkel, C. Ludwig, M. E. Swai, R. Y. Yang, K. P. Chun and S. D. Habicht | 2018 | Bitter gourd reduces elevated fasting plasma glucose levels in an intervention study among prediabetics in Tanzania | Repetition |
|  | T. Brendler, A. Al-Harrasi, R. Bauer, S. Gafner, M. L. Hardy et al. | 2006 | Botanical drugs and supplements affecting the immune response in the time of COVID-19: implications for research and clinical practice | Subject |
|  | Botanical drugs and supplements affecting the immune response in the time of COVID-19: Implications for research and clinical practice | 2019 | Botanical drugs and supplements affecting the immune response in the time of COVID-19: implications for research and clinical practice | Repetition |
|  | S. McKenna, F. Jones, P. Glenfield and S. Lennon | 2015 | Bridges self-management program for people with stroke in the community: A feasibility randomized controlled trial | Methods |
|  | J. Daniels, H. Struthers, J. Soler, E. Ricco, J. Blackmon, S. Teklehaimanot, J. McIntyre and T. Coates | 2021 | Building self-advocacy in HIV care: the use of role-play to examine healthcare access for HIV-positive MSM in rural South Africa | Subject |
|  | J. Daniels, H. Struthers, J. Soler, E. Ricco, J. Blackmon, S. Teklehaimanot, J. McIntyre and T. Coates | 2021 | Building self-advocacy in HIV care: the use of role-play to examine healthcare access for HIV-positive MSM in rural South Africa | Repetition |
|  | D. A. Berhe, M. K. Yenit and A. G. Baraki | 2020 | The Burden of Hypertension and Associated Factors Among Adults Visiting the Adult Outpatient Department at Yekatit 12 Hospital Medical College, Addis Ababa, Ethiopia, 2016 | Methods |
|  | M. E. Loades, B. Coetzee, S. d. Toit and A. Kagee | 2022 | '...But I'm still tired': the experience of fatigue among South African adolescents receiving antiretroviral therapy | Population |
|  | https://www.tandfonline.com/loi/caic20 | 2019 |  | Invalid |
|  | M. E. Loades, B. Coetzee, S. Du Toit and A. Kagee | 2011 | ‘ … But i’m still tired’: the experience of fatigue among South African adolescents receiving antiretroviral therapy | Population |
|  | N. Anumolu, K. Lechleitner, N. Patel, A. Mijumbi, C. Jankowski et al. | 2019 | By the time they run into the hospital, their life is already at stake: a qualitative study of healthcare professional perceptions on priorities for cervical cancer policy in Uganda | Subject |
|  | N. Anumolu, K Lechleitner, N. Patel, A. Mijumbi, C. Jankowski | 2018 | By the time they run into the hospital, their life is already at stake”: a qualitative study of healthcare professional perceptions on priorities for cervical cancer policy in Uganda | Subject |
|  |  | 2022 | Can community carers cope? | Invalid |
|  | S. Kenya, I. S. Okoro, K. Wallace, M. Ricciardi, O. Carrasquillo and G. Prado | 2022 | Can Home-Based HIV Rapid Testing Reduce HIV Disparities Among African Americans in Miami? | Setting |
|  | C. Moucheraud, A. F. Stern, A. Ismail, T. Nsubuga-Nyombi, M. M. Ngonyani et al. | 2020 | Can Self-Management Improve HIV Treatment Engagement, Adherence, and Retention? A Mixed Methods Evaluation in Tanzania and Uganda | Subject |
|  | A. Banbury, D. Chamberlain, S. Nancarrow, J. Dart, L. Gray and L. Parkinson | 2017 | Can videoconferencing affect older people's engagement and perception of their social support in long-term conditions management: a social network analysis from the Telehealth Literacy Project | Subject |
|  | M. Kangasniemi, M. Hirjaba, K. Kohonen, E. Vellone, T. Moilanen and A.-M. Pietilä | 2017 | The cardiac patients' perceptions of their responsibilities in adherence to care: a qualitative interview study | Subject |
|  | S. L. Grace, K. I. Turk-Adawi, A. Contractor, A. Atrey, N. Campbell et al | 2016 | Cardiac rehabilitation delivery model for low-resource settings | Subject |
|  | W. Chen, C. Shiu, L. Zhang and H. Zhao | 2022 | Care engagement with healthcare providers and symptom management self-efficacy in women living with HIV in China: secondary analysis of an intervention study | Setting |
|  | W. Chen, C. Shiu, L. Zhang and H. Zhao | 2022 | Care engagement with healthcare providers and symptom management self-efficacy in women living with HIV in China: secondary analysis of an intervention study | Repetition |
|  | E. R. Locke, J. P. Young, C. Battaglia, T. L. Simpson, R. Trivedi et al. | 2022 | Care-seeking and delay of care during COPD exacerbations | Subject |
|  | M. Suresh, J. Young, V. Fan, C. Simons, C. Battaglia, T. L. Simpson, J. C. Fortney, E. R. Locke and R. Trivedi | 2022 | Caregiver Experiences and Roles in Care Seeking During COPD Exacerbations: A Qualitative Study | Subject |
|  | L. J. Avery, A. Szwajcer, S. Zieroth, B. Temple and J.-A. V. Sawatzky | 2016 | Caregiver experiences of providing care to adult individuals living with a left ventricular assist device: a qualitative systematic review protocol | Protocol |
|  | M. L. Mazur | 2002 | Carole Gale into Africa | Subject |
|  | K. M. Murphy, R. Mash and Z. Malan | 2016 | The case for behavioural change counselling for the prevention of NCDs and improvement of self-management of chronic conditions | Subject |
|  | K. M. Murphy, R. Mash and Z. Malan | 2016 | The case for behavioural change counselling for the prevention of NCDs and improvement of selfmanagement of chronic conditions | Repetition |
|  | K. M. Murphy, R. Mash and Z. Malan | 2016 | The case for behavioural change counselling for the prevention of NCDs and improvement of selfmanagement of chronic conditions | Repetition |
|  | A. Sobry, W. Kizito, R. Van den Bergh, K. Tayler-Smith, P. Isaakidis et al. | 2014 | Caseload, management and treatment outcomes of patients with hypertension and/or diabetes mellitus in a primary health care programme in an informal setting | Repetition |
|  |  | 2021 | Catalysing policy change to introduce and scale up self-care interventions for SRHR: lessons from the Eastern Mediterranean Region | Subject |
|  | A. Kakooza-Mwesige, L. E. Wachtel and D. M. Dhossche | 2008 | Catatonia in autism: implications across the life span | Subject |
|  | L. J. Johnson, L. H. Schopp, F. Waggie and J. M. Frantz | 2022 | Challenges experienced by community health workers and their motivation to attend a self-management programme | Subject |
|  | G. Lan, C. Porr, K. Parsons, J. Allison and C. Donovan | 2017 | Challenges in self- management of Type 2 diabetes mellitus (T2DM) in a rural community of Eastern Canada | Setting |
|  | J. W. McGrath, M. S. Winchester, D. Kaawa-Mafigiri, E. Walakira, F. Namutiibwa et al. | 2014 | Challenging the paradigm: anthropological perspectives on HIV as a chronic disease | Subject |
|  | C. Bowie, N. Gondwe and C. Bowie | 2010 | Changing clinical needs of people living with AIDS and receiving home based care in Malawi - the Bangwe Home Based Care Project 2003-2008 - a descriptive study | Subject |
|  | C. Bowie, N. Gondwe and C. Bowie | 2010 | Changing clinical needs of people living with AIDS and receiving home based care in Malawi--the Bangwe Home Based Care Project 2003-2008--a descriptive study | Repetition |
|  | J. Mwangi, F. Miruka, M. Mugambi, A. Fidhow, B. Chepkwony et al. | 2022 | Characteristics of users of HIV self-testing in Kenya, outcomes, and factors associated with use: results from a population-based HIV impact assessment, 2018 | Subject |
|  | J. Mwangi, F. Miruka, M. Mugambi, A. Fidhow, B. Chepkwony et al. |  | Characteristics of users of HIV self-testing in Kenya, outcomes, and factors associated with use: results from a population-based HIV impact assessment, 2018 | Repetition |
|  | A. Malata and E. Chirwa | 2011 | Childbirth information feeds for first time Malawian mothers who attended antenatal clinics | Subject |
|  | A. Malata and E. Chirwa | 20112019 | Childbirth information needs for first time Malawian mothers who attended antenatal clinics | Repetition |
|  | R. C. Walker, D. Naicker, T. Kara and S. C. Palmer | 2019 | Children's experiences and expectations of kidney transplantation: A qualitative interview study | Population |
|  | E. A. Kelvin, S. Cheruvillil, S. Christian, J. E. Mantell, C. Milford et al. | 2016 | Choice in HIV testing: the acceptability and anticipated use of a self-administered at-home oral HIV test among South Africans | Subject |
|  | https://www.tandfonline.com/loi/raar20 | 2021 |  | Invalid |
|  | S. Palmer | 2003 | Christmas foods around the world | Subject |
|  | S. Duangbubpha, S. Hanucharurnkul, R. Pookboonmee, P. Orathai and C. Kiatboonsri | 2013 | Chronic Care Model Implementation and Outcomes among Patients with COPD in Care Teams with and without Advanced Practice Nurses | Subject |
|  | S. N. Tulu, P. Cook, K. S. Oman, P. Meek and E. Kebede Gudina | 2021 | Chronic disease self-care: A concept analysis | Subject |
|  | S. Duangpaeng, P. Eusawas, S. Laungamornlert, S. Gasemgitvatana and W. Sritanyarat | 2002 | Chronic dyspnea self-management of Thai adults with COPD | Subject |
|  | C. Mattingly, L. Grøn and L. Meinert | 2011 | Chronic homework in emerging borderlands of healthcare | Subject |
|  | C. Mattingly, L. Grøn, L. Meinert, C. Mattingly, L. Grøn and L. Meinert | 2011 | Chronic homework in emerging borderlands of healthcare | Repetition |
|  | C.-T. Chao, R.-S. Yang, L.-W. Hung, K.-S. Tsai, J.-K. Peng et al. | 2018 | Chronic kidney disease predicts a lower probability of improvement in patient-reported experience measures among patients with fractures: a prospective multicenter cohort study | Subject |
|  | J. Martini, A. Tijou Traoré and C. Mahieu | 2019 | Chronic patient as intermittent partner for policy-makers: the case of patient participation in the fight against diabetes and HIV/AIDS in Mali | Subject |
|  | J. Martini, A. T. Traoré and C. Mahieu | 2019 | Chronic patient as intermittent partner for policy-makers: the case of patient participation in the fight against diabetes and HIV/AIDS in Mali | Repetition |
|  | T. Melaku, L. Chelkeba and Z. Mekonnen | 2020 | Clinical care & blood pressure control among hypertensive people living with human immune deficiency virus: Prospective cohort study | Subject |
|  | C.-H. Wei, T.-C. Peng, P.-Y. Wang, J.-J. Lin, M.-L. Chuang and C.-C. Chen | 2009 | Clinical evaluation and alternative site blood glucose testing of the EasyPlus mini R2N blood glucose monitoring system | Subject |
|  | R. A. Afaya, V. Bam, A. Y. Lomotey and A. Afaya | 2022 | Clinical factors influencing knowledge and self-care practice among adults with type 2 diabetes mellitus | Subject |
|  | R. C. Walker, A. Tong, K. Howard and S. C. Palmer | 2020 | Clinicians' experiences with remote patient monitoring in peritoneal dialysis: A semi-structured interview study | Subject |
|  | J. T. Kelly, M. Conley, T. Hoffmann, J. C. Craig, A. Tong et al. | 2020 | A Coaching Program to Improve Dietary Intake of Patients with CKD: ENTICE-CKD | Subject |
|  | T. A. Caller, R. J. Ferguson, R. M. Roth, K. L. Secore, F. P. Alexandre et al. | 2016 | A cognitive behavioral intervention (HOBSCOTCH) improves quality of life and attention in epilepsy | Subject |
|  | O. Mueller, O. Razum, C. Traore and B. Kouyate | 2004 | Community effectiveness of chloroquine and traditional remedies in the treatment of young children with falciparum malaria in rural Burkina Faso | Subject |
|  | C. Blixen, J. Nakibuuka, M. Kaddumukasa, H. Nalubwama, M. Amollo, E. Katabira and M. Sajatovic | 2017 | Community Perceptions of Barriers to Stroke Recovery and Prevention in Greater Kampala, Uganda: Implications for Policy and Practice | Subject |
|  | C. Lygidakis, J. P. Uwizihiwe, P. Kallestrup, M. Bia, J. Condo and C. Vögele | 2019 | Community- and mHealth-based integrated management of diabetes in primary healthcare in Rwanda (D²Rwanda): the protocol of a mixed-methods study including a cluster randomised controlled trial | Subject |
|  | C. N. Igwesi-Chidobe, P. C. Okechi, G. N. Emmanuel and B. C. Ozumba | 2022 | Community-based non-pharmacological interventions for pregnant women with gestational diabetes mellitus: a systematic review | Subject |
|  | C. Franz, S. Atwood, E. J. Orav, C. Curley, C. Brown, L. Trevisi, A. K. Nelson, M.-G. Begay and S. Shin | 2020 | Community-based outreach associated with increased health utilization among Navajo individuals living with diabetes: a matched cohort study | Methods |
|  | C. Franz, S. Atwood, E. J. Orav, C. Curley, C. Brown, L. Trevisi, A. K. Nelson, M. G. Begay and S. Shin | 2020 | Community-based outreach associated with increased health utilization among Navajo individuals living with diabetes: a matched cohort study | Repetition |
|  | C. Franz, S. Atwood, E. J. Orav, C. Curley, C. Brown, L. Trevisi, A. K. Nelson, M. G. Begay and S. Shin | 2020 | Community-based outreach associated with increased health utilization among Navajo individuals living with diabetes: a matched cohort study | Repetition |
|  | F. K. Assah, E. N. Atanga, S. Enoru, E. Sobngwi and J. C. Mbanya | 2015 | Community-based peer support significantly improves metabolic control in people with Type 2 diabetes in Yaoundé, Cameroon | Methods |
|  | C. N. Igwesi-Chidobe, E. L. Godfrey, S. Kitchen, C. N. Onwasigwe and I. O. Sorinola | 2019 | Community-based self-management of chronic low back pain in a rural African primary care setting: a feasibility study | Subject |
|  | I. Maatouk, M. El-Nakib, M. Assi, P. Farah, B. Makso, C. El-Nakib and A. Rady | 2021 | Community-led HIV self-testing for men who have sex with men in Lebanon: lessons learned and impact of COVID-19 | Subject |
|  | I. Maatouk, M. E. Nakib, M. Assi, P. Farah, B. Makso, C. E. Nakib and A. Rady | 2021 | Community-led HIV self-testing for men who have sex with men in Lebanon: lessons learned and impact of COVID-19 | Repetition |
|  | I. Maatouk, M. E. Nakib, M. Assi, P. Farah, B. Makso, C. E. Nakib and A. Rady | 2021 | Community-led HIV self-testing for men who have sex with men in Lebanon: lessons learned and impact of COVID-19 | Repetition |
|  | B. D. Oladeji and O. Gureje | 2013 | The comorbidity between depression and diabetes | Subject |
|  | M. Godongwana, N. De Wet-Billings and M. Milovanovic | 2021 | The comorbidity of HIV, hypertension and diabetes: a qualitative study exploring the challenges faced by healthcare providers and patients in selected urban and rural health facilities where the ICDM model is implemented in South Africa | Subject |
|  | M. Godongwana, N. d. Wet-Billings and M. Milovanovic | 2021 | The comorbidity of HIV, hypertension and diabetes: a qualitative study exploring the challenges faced by healthcare providers and patients in selected urban and rural health facilities where the ICDM model is implemented in South Africa | Repetition |
|  | https://link.springer.com/article/10.1186/s12913-021-06670-3 | 2021 | The comorbidity of HIV, hypertension and diabetes: a qualitative study exploring the challenges faced by healthcare providers and patients in selected urban and rural health facilities where the ICDM model is implemented in South Africa | Repetition |
|  | J. A. Salomon, A. Patel, B. Neal, P. Glasziou, D. E. Grobbee, J. Chalmers and P. M. Clarke | 2011 | Comparability of Patient-reported Health Status: Multicountry Analysis of EQ-5D Responses in Patients With Type 2 Diabetes | Subject |
|  | R. C. Bonadonna, A. Giaccari, R. Buzzetti, G. Perseghin, D. Cucinotta et a. | 2020 | Comparable efficacy with similarly low risk of hypoglycaemia in patient- vs physician-managed basal insulin initiation and titration in insulin-naïve type 2 diabetic subjects: The Italian Titration Approach Study | Subject |
|  | M. Wolde, G. Tarekegn and T. Kebede | 2018 | Comparative Evaluations of Randomly Selected Four Point-of-Care Glucometer Devices in Addis Ababa, Ethiopia | Subject |
|  | F. C. Mukumbang, S. Ndlovu and B. van Wyk | 2022 | Comparing Patients' Experiences in Three Differentiated Service Delivery Models for HIV Treatment in South Africa | Subject |
|  | F. C. Mukumbang, S. Ndlovu and B. v. Wyk | 2022 | Comparing patients' experiences in three differentiated service delivery models for HIV treatment in South Africa | Repetition |
|  | F. C. Mukumbang, S. Ndlovu and B. v. Wyk | 2022 | Comparing patients' experiences in three differentiated service delivery models for HIV treatment in South Africa | Repetition |
|  | B. Kilic, H. S. Cicek and M. Z. Avci | 2021 | Comparing the effects of self-management and hospital-based pulmonary rehabilitation programs in COPD patients | Subject |
|  | O. K. Ajenifuja, N. Z. Ikeri, O. V. Adeteye and A. A. Banjo | 2018 | Comparison between self sampling and provider collected samples for Human Papillomavirus (HPV) Deoxyribonucleic acid (DNA) testing in a Nigerian facility | Subject |
|  | E. L. Sibanda, C. Mangenah, M. Neuman, M. Tumushime, C. Watadzaushe et al. | 2021 | Comparison of community-led distribution of HIV self-tests kits with distribution by paid distributors: a cluster randomised trial in rural Zimbabwean communities | Subject |
|  | E. L. Sibanda, C. Mangenah, M. Neuman, M. Tumushime, C. Watadzaushe et al. | 2021 | Comparison of community-led distribution of HIV self-tests kits with distribution by paid distributors: a cluster randomised trial in rural Zimbabwean communities | Repetition |
|  | T. Crucitti, E. Van Dyck, A. Tehe, S. Abdellati, B. Vuylsteke, A. Buve and M. Laga | 2003 | Comparison of culture and different PCR assays for detection of Trichomonas vaginalis in self collected vaginal swab specimens | Subject |
|  | J. M. Milln, E. Walugembe, S. Ssentayi, H. Nkabura, A. G. Jones and M. J. Nyirenda | 2020 | Comparison of oral glucose tolerance test and ambulatory glycaemic profiles in pregnant women in Uganda with gestational diabetes using the FreeStyle Libre flash glucose monitoring system | Subject |
|  | S. Tonen-Wolyec, C. K. Tshilumba, S. Batina-Agasa, R. M. Djang'Eing'A, M. P. Hayette and L. Belec | 2020 | Comparison of practicability and effectiveness between unassisted HIV self-testing and directly assisted HIV self-testing in the Democratic Republic of the Congo: a randomized feasibility trial | Subject |
|  | S. Tonen-Wolyec, C. K. Tshilumba, S. Batina-Agasa, R. M. Djang'Eing'A, M. P. Hayette and L. Belec | 2020 | Comparison of practicability and effectiveness between unassisted HIV self-testing and directly assisted HIV self-testing in the Democratic Republic of the Congo: a randomized feasibility trial | Repetition |
|  | J. A. Shultz, M. A. Sprague, L. J. Branen and S. Lambeth | 2001 | A comparison of views of individuals with type 2 diabetes mellitus and diabetes educators about barriers to diet and exercise | Repetition |
|  | C. Boult, G. D. Wieland, C. Boult and G. D. Wieland | 2010 | Comprehensive primary care for older patients with multiple chronic conditions: "Nobody rushes you through" | Subject |
|  | E. W. M. A. Bischoff, R. Akkermans, J. Bourbeau, C. van Weel, J. H. Vercoulen and T. R. J. Schermer | 2012 | Comprehensive self management and routine monitoring in chronic obstructive pulmonary disease patients in general practice: randomised controlled trial | Subject |
|  | P. Hakkarainen, F. Munir, L. Moilanen, K. Räsänen and V. Hänninen | 2018 | Concealment of type 1 diabetes at work in Finland: a mixed-method study | Setting |
|  | A. Marais, V. Steenkamp and W. J. Du Plooy | 2017 | Conditions frequently self-treated with herbal remedies by patients visiting a tertiary hospital in Gauteng, South Africa | Methods |
|  | K. Lowther, R. Harding, A. Ahmed, N. Gikaara, Z. Ali, H. Kariuki, L. Sherr, V. Simms and L. Selman | 2016 | Conducting experimental research in marginalised populations: clinical and methodological implications from a mixed-methods randomised controlled trial in Kenya | Methods |
|  | D. Kibira, V. Boydell, L. Mworeko and J. Kiarie | 2021 | Considerations for social accountability in the expansion of self-care for sexual and reproductive health and rights | Methods |
|  | M. O. Adibe, C. N. Aguwa and C. V. Ukwe | 2011 | The construct validity of an instrument for measuring type 2 diabetes self-care knowledge in Nigeria | Subject |
|  | M. O. Adibe, C. N. Aguwa and C. V. Ukwe | 2011 | The construct validity of an instrument for measuring type 2 diabetes self-care knowledge in Nigeria | Repetition |
|  | J. Van Olmen, K. G. Marie, D. Christian, K. J. Clovis, B. Emery et al. | 2015 | Content, participants and outcomes of three diabetes care programmes in three low and middle income countries | Population |
|  | M. C. Nagenda and T. Crowley | 2022 | Contextual factors influencing self-management of adolescents and youth living with HIV: a cross-sectional survey in Lesotho | Population |
|  | https://www.mdpi.com/1660-4601/20/1/238 | 2022 | Contextual factors influencing self-management of adolescents and youth living with HIV: a cross-sectional survey in Lesotho | Subject |
|  | A. Wahl, B. R. Hanestad, I. Wiklund and T. Moum | 1999 | Coping and quality of life in patients with psoriasis | Population |
|  | T. M. Kamau, V. G. Olson, G. P. Zipp and M. Clark | 2011 | Coping self-efficacy as a predictor of adherence to antiretroviral therapy in men and women living with HIV in Kenya | Subject |
|  | https://www.liebertonline.com/loi/apc | 2020 |  | Invalid |
|  | G. Hapunda | 2022 | Coping strategies and their association with diabetes specific distress, depression and diabetes self-care among people living with diabetes in Zambia | Population |
|  | R. Roomaney and A. Kagee | 2016 | Coping strategies employed by women with endometriosis in a public health-care setting | Subject |
|  | J. Changole, V. Thorsen, J. Trovik, U. Kafulafula and J. Sundby | 2019 | Coping with a Disruptive Life Caused by Obstetric Fistula: Perspectives from Malawian Women | Subject |
|  | L. N. Makoae, M. Greeff, R. D. Phetlhu, L. R. Uys, J. R. Naidoo et al. | 2008 | Coping with HIV-related stigma in five African countries | Subject |
|  | G. Hapunda, A. Abubakar, F. Pouwer and F. v. d. Vijver | 2020 | Correlates of fear of hypoglycemia among patients with type 1 and 2 diabetes mellitus in outpatient hospitals in Zambia | Subject |
|  | https://link.springer.com/article/10.1007/s13410-020-00835-2 | 2020 | Correlates of fear of hypoglycemia among patients with type 1 and 2 diabetes mellitus in outpatient hospitals in Zambia | Repetition |
|  | S. Letta, F. Aga, T. A. Yadeta, B. Geda and Y. Dessie | 2022 | Correlates of Glycemic Control Among Patients With Type 2 Diabetes in Eastern Ethiopia: A Hospital-Based Cross-Sectional Study | Subject |
|  | J. Hu, Y. Dong, J. Wei and X. Huang | 2017 | Correlation between self-efficacy, self-management and glycemic control among seniors with diabetes in rural areas | Repetition |
|  | M. K. Nkoana and A. A. Khine | 2019 | Correlation between self-monitored mean blood glucose and average plasma glucose estimated from glycated haemoglobin in patients attending the diabetes clinic at Dr George Mukhari Academic Hospital, Pretoria, South Africa | Subject |
|  | M. M. Kebede and C. R. Pischke | 2019 | Corrigendum: Popular Diabetes Apps and the Impact of Diabetes App Use on Self-Care Behaviour: A Survey Among the Digital Community of Persons With Diabetes on Social Media | Subject |
|  | L. Lebina, M. Kawonga, T. Oni, H. Kim and O. A. Alaba | 2020 | The cost and cost implications of implementing the integrated chronic disease management model in South Africa | Subject |
|  | L. Lebina, M. Kawonga, T. Oni, H. Kim and O. A. Alaba | 2020 | The cost and cost implications of implementing the integrated chronic disease management model in South Africa | Repetition |
|  | L. Lebina, M. Kawonga, T. Oni, H.-Y. Kim and O. A. Alaba | 2020 | The cost and cost implications of implementing the integrated chronic disease management model in South Africa | Repetition |
|  | M. Wilson-Barthes, P. Braitstein, A. DeLong, D. Ayuku, L. Atwoli, E. Sang and O. Galárraga | 2022 | Cost Utility of Supporting Family-Based Care to Prevent HIV and Deaths among Orphaned and Separated Children in East Africa: A Markov Model-Based Simulation | Subject |
|  | E. Oksman, M. Linna, I. Hörhammer, J. Lammintakanen and M. Talja | 2017 | Cost-effectiveness analysis for a tele-based health coaching program for chronic disease in primary care | Subject |
|  | R. Mash, R. Kroukamp, T. Gaziano and N. Levitt | 2015 | Cost-effectiveness of a diabetes group education program delivered by health promoters with a guiding style in underserved communities in Cape Town, South Africa | Subject |
|  | H. Maheswaran, A. Clarke, P. MacPherson, F. Kumwenda, D. G. Lalloo et al. | 2018 | Cost-Effectiveness of Community-based Human Immunodeficiency Virus Self-Testing in Blantyre, Malawi | Subject |
|  | E. I. Broughton, M. Muhire, E. Karamagi and H. Kisamba | 2016 | Cost-effectiveness of implementing the chronic care model for HIV care in Uganda | Subject |
|  | M. O. Adibe, C. N. Aguwa and C. V. Ukwe | 2013 | Cost-Utility Analysis of Pharmaceutical Care Intervention Versus Usual Care in Management of Nigerian Patients with Type 2 Diabetes | Subject |
|  | G. A. Gregory, J. Guo, E. L. Klatman, G. A. Ahmadov, S. Besançon et al. | 2020 | Costs and outcomes of "intermediate" vs "minimal" care for youth-onset type 1 diabetes in six countries | Subject |
|  | E. Kapyepye | 1994 | Country watch. Zambia | Subject |
|  | S. Zhai, N. Xu, S. Liu, Z. Liu, X. Liu and F. Wei | 2022 | Cross-cultural adaptation and validation of the Chinese version of the Spine Oncology Study Group Outcomes Questionnaire | Setting |
|  | S. Amrita, R. Mittal, P. Nirmika, C. Meet, M. Naresh and P. Dipesh | 2018 | A cross-sectional analytical study of knowledge and practice of exercise among hypertensive patients attending NCD clinic in western Gujarat | Setting |
|  | https://www.ijmedph.org/article/608 | 2018 | A cross-sectional analytical study of knowledge and practice of exercise among hypertensive patients attending NCD clinic in western Gujarat | Repetition |
|  | R. Lamptey, M. J. Davies, K. Khunti, S. Schreder, B. Stribling and M. Hadjiconstantinou | 2022 | Cultural adaptation of a diabetes self-management education and support (DSMES) programme for two low resource urban settings in Ghana, during the COVID-19 era | Subject |
|  | H. Alaofè, S. Yeo, A. Okechukwu, P. Magrath, W. Amoussa Hounkpatin, J. Ehiri and C. Rosales | 2021 | Cultural Considerations for the Adaptation of a Diabetes Self-Management Education Program in Cotonou, Benin: Lessons Learned from a Qualitative Study | Repetition |
|  | H. Alaofè, S. Yeo, A. Okechukwu, P. Magrath, W. A. Hounkpatin, J. Ehiri and C. Rosales | 2021 | Cultural considerations for the adaptation of a diabetes self-management education program in Cotonou, Benin: lessons learned from a qualitative study | Repetition |
|  | H. Alaofè, S. Yeo, A. Okechukwu, P. Magrath, W. A. Hounkpatin, J. Ehiri and C. Rosales | 2021 | Cultural considerations for the adaptation of a diabetes self-management education program in Cotonou, Benin: lessons learned from a qualitative study | Repetition |
|  | T. Li-Geng, J. Kilham and K. M. McLeod | 2020 | Cultural influences on dietary self-management of type 2 diabetes in East Asian Americans: a mixed-methods systematic review | Subject |
|  | T. Li-Geng, J. Kilham and K. M. McLeod | 2020 | Cultural influences on dietary self-management of type 2 diabetes in East Asian Americans: a mixed-methods systematic review | Repetition |
|  | R. BeLue, M. Diaw, F. Ndao, T. Okoror, A. Degboe and B. Abiero | 2012 | A cultural lens to understanding daily experiences with type 2 diabetes self-management among clinic patients in M'bour, Senegal | included |
|  | M. Werfalli, K. Murphy, S. Kalula and N. Levitt | 2019 | Current policies and practices for the provision of diabetes care and self-management support programmes for older South Africans | Subject |
|  | J. Wamoyi, P. Balvanz, M. W. Gichane, S. Maman, S. Mugunga, E. Majani and A. Pettifor | 2020 | Decision-making and cash spending patterns of adolescent girls and young women participating in a cash-transfer intervention in Tanzania: Implications for sexual health | Population |
|  | https://www.tandfonline.com/doi/full/10.1080/17441692.2019.1692891 | 2020 | Decision-making and cash spending patterns of adolescent girls and young women participating in a cash-transfer intervention in Tanzania: Implications for sexual health | Repetition |
|  | R.A. Roomaney, B. Van Wyk, V. Van Wyk | 2021 | Decolonising multimorbidity? research gaps in low and middle-income countries | Subject |
|  | M. Luacche Xavier Ferreira de Sousa, K. de Lima Silva, M. M. Lima da N¢brega and N. Collet | 2012 | Deficits de autocuidado em criancas e adolescentes com doenca renal cronica | language |
|  | J. O. Olobo and G. D. F. Reid | 1993 | Delayed-type hypersensitivity responses in vervet monkeys self-cured from experimental cutaneous leishmaniasis | Subject |
|  | A. Abyot, J. Degu and D. Wakgari | 2019 | Delays to anti-tuberculosis treatment intiation among cases on directly observed treatment short course in districts of southwestern Ethiopia: a cross sectional study | Subject |
|  | C. Sun, J. Dohrn, A. Oweis, H. A. S. Huijer, F. Abu ‐ Moghli, H. Dawani, C. Ghazi and E. Larson | 2017 | Delphi Survey of Clinical Nursing and Midwifery Research Priorities in the Eastern Mediterranean Region | Subject |
|  | C. O. Sowunmi, O. A. Lanre-Ogungbile, C. A. Owopetu and J. K. Opele | 2021 | Demographic variables and self-management practices as correlates of glycemic control among reproductive age diabetic women in a tertiary hospital in Ile-Ife, Osun State, Nigeria | Subject |
|  | https://www.texilajournal.com/public-health/article/1874-demographic-variables-and | 2021 | Demographic Variables and Self-Management Practices as Correlates of Glycemic Control among Reproductive Age Diabetic Women in a Tertiary Hospital in Ile-Ife, Osun State, Nigeria | Repetition |
|  | L. Baumann, N. Frederick, N. Betty, E. Jospehine and N. Agatha | 2015 | A Demonstration of Peer Support for Ugandan Adults With Type 2 Diabetes | Subject |
|  | L. C. Baumann, N. Frederick, N. Betty, E. Jospehine and N. Agatha | 2015 | A demonstration of peer support for Ugandan adults with type 2 diabetes | Repetition |
|  | I. A. Al-Khatib, M. Ishtayeh, H. Barghouty and B. Akkawi | 2006 | Dentists' perceptions of occupational hazards and preventive measures in East Jerusalem | Setting |
|  | A. Koyanagi, D. Vancampfort, A. F. Carvalho, J. E. DeVylder, J. M. Haro et al. | 2017 | Depression comorbid with tuberculosis and its impact on health status: cross-sectional analysis of community-based data from 48 low- and middle-income countries | Subject |
|  | G. Hapunda, A. Abubakar, F. Pouwer and F. van de Vijver | 2017 | Depressive Symptoms Are Negatively Associated with Glucose Testing and Eating Meals on Time among Individuals with Diabetes in Zambia | Subject |
|  | L. S. Eller, M. Rivero-Mendez, J. Voss, W. T. Chen, P. Chaiphibalsarisdi et al. | 2014 | Depressive symptoms, self-esteem, HIV symptom management self-efficacy and self-compassion in people living with HIV | Subject |
|  | N. Rouveau, O. Ky-Zerbo, S. Boye, A. S. Fotso, M. D'Elbée et al. | 2021 | Describing, analysing and understanding the effects of the introduction of HIV self-testing in West Africa through the atlas programme in côte d'Ivoire, Mali and Senegal | Subject |
|  | N. Rouveau, O. Ky-Zerbo, S. Boye, A. S. Fotso, M. D'Elbée et al. | 2021 | Describing, analysing and understanding the effects of the introduction of HIV self-testing in West Africa through the ATLAS programme in Côte d’Ivoire, Mali and Senegal | Invalid |
|  | P. Burnard, P. Haji Abd Rahim, D. Hayes and D. Edwards | 2007 | A descriptive study of Bruneian student nurses' perceptions of stress | Setting |
|  | T. A. Caller, K. L. Secore, R. J. Ferguson, R. M. Roth, F. P. Alexandre et al. | 2015 | Design and feasibility of a memory intervention with focus on self-management for cognitive impairment in epilepsy | Subject |
|  | A. Garden, M. D. Taylor, J. Davidson, C. J. Gilbride and C. Bradley | 2022 | Design of an individualised questionnaire to measure the impact of cancer on quality of life: The cancer dependent quality of life (CancerDQoL) questionnaire | Subject |
|  | S. M. Mensa, D. Majid, K. Abbas | 2022 | Current hypertension care practice in Sub-Saharan Africa and need for creating hypertension care ecosystem: An explanatory Review. | Review |
|  | Magodoro IM, Esterhuizen TM, Chivese T. | 2016 | A cross-sectional, facility based study of comorbid non-communicable diseases among adults living with HIV infection in Zimbabwe | Subject |
|  | M. Barbera, F. Mangialasche, S. Jongstra, J. Guillemont, T. Ngandu et al. | 2018 | Designing an Internet-Based Multidomain Intervention for the Prevention of Cardiovascular Disease and Cognitive Impairment in Older Adults: The HATICE Trial | Subject |
|  | G. Jepkemoi, C. Gichunge and P. Masibo | 2021 | Determinants of adherence to dietary guidelines among type 2 diabetes mellitus patients at MOI Teaching and Referral Hospital-Kenya | Subject |
|  | https://www.ajfand.net/Volume21/No3/Jepkemoi19370.pdf | 2021 | Demographic Variables and Self-Management Practices as Correlates of Glycemic Control among Reproductive Age Diabetic Women in a Tertiary Hospital in Ile-Ife, Osun State, Nigeria | Subject |
|  | B. Barasa Masaba and R. M. Mmusi-Phetoe | 2021 | Determinants of Non-Adherence to Treatment Among Patients with Type 2 Diabetes in Kenya: A Systematic Review | Review |
|  | L. Danquah, P. M. Amegbor and D. G. Ayele | 2021 | Determinants of the type of health care sought for symptoms of acute respiratory infection in children: analysis of Ghana demographic and health surveys | Subject |
|  | https://link.springer.com/article/10.1186/s12887-021-02990-9 | 2021 | Determinants of the type of health care sought for symptoms of Acute respiratory infection in children: analysis of Ghana demographic and health surveys | Repetition |
|  | A. Kelly-Hanku, S. Bell, S. Ase, R. Boli-Neo, A. J. Vallely et al. | 2019 | Developing a culturally appropriate illustrated tool for the self-collection of anorectal specimens for the testing of sexually transmitted infections: lessons from Papua New Guinea | Subject |
|  | M. DiGiacomo, A. Green, E. Rodrigues, K. Mulligan and P. M. Davidson | 2015 | Developing a gender-based approach to chronic conditions and women's health: a qualitative investigation of community-dwelling women and service provider perspectives | Subject |
|  | O. Ibiyemi, F. Lawal, M. Osuh, T. Owoaje, E. Idiga, O. Fagbule and O. Ijarogbe | 2022 | Developing an Oral Hygiene Education Song for Children and Teenagers in Nigeria | Subject |
|  | Y.-T. Chiang, C.-W. Chang, H.-Y. Yu, P.-K. Tsay, F.-S. Lo et al. | 2022 | Developing the "Healthcare CEO App" for patients with type 1 diabetes transitioning from adolescence to young adulthood: A mixed-methods study | Subject |
|  | C. Lambert, N. D. Meité, I. Sanogo, S. Lobet and C. Hermans | 2019 | Development and evaluation of appropriate, culturally adapted educational tools for Ivoirian patients with haemophilia, haemophilia carriers and their families | Subject |
|  | S. Jongstra, C. Beishuizen, S. Andrieu, M. Barbera, M. van Dorp et al. | 2017 | Development and Validation of an Interactive Internet Platform for Older People: The Healthy Ageing Through Internet Counselling in the Elderly Study | Subject |
|  | C. Y. Han, X. Zheng, L. F. Lee, C. G. B. Chan, Y. Q. Lee, N. A. Zailani, K. Ng and K. Bhaskaran | 2019 | Development of a diabetes‐related nutrition knowledge questionnaire for individuals with type 2 diabetes mellitus in Singapore | Subject |
|  | S. M. Sefa-Yeboah, K. Osei Annor, V. J. Koomson, F. K. Saalia, M. Steiner-Asiedu and G. A. Mills | 2021 | Development of a Mobile Application Platform for Self-Management of Obesity Using Artificial Intelligence Techniques | Subject |
|  | J. Ebenso, P. Fuzikawa, H. Melchior, R. Wexler, A. Piefer et al. | 2007 | The development of a short questionnaire for screening of activity limitation and safety awareness (SALSA) in clients affected by leprosy or diabetes | Subject |
|  | B. Myers, T. Carney, F. A. Browne and W. M. Wechsberg | 2018 | Development of a trauma-informed substance use and sexual risk reduction intervention for young South African women | Subject |
|  | M. Dekhtyar, C. Colford, E. Whisenant, J. Huber, E. Johnson et al. | 2021 | Development of Learning Objectives to Guide Enhancement of Chronic Disease Prevention and Management Curricula in Undergraduate Medical Education | Subject |
|  | C. L. Edwards, A. G. Kaplan, B. P. Yawn, J. W. H. Kocks, L. Bulathsinhala et al. | 2020 | Development of the Advancing the Patient Experience in COPD Registry: A Modified Delphi Study | Subject |
|  | M. J. Rotheram-Borus, M. Tomlinson, M. Gwegwe, W. S. Comulada, N. Kaufman and M. Keim | 2012 | Diabetes buddies: peer support through a mobile phone buddy system | Subject |
|  | E. Mendenhall and S. A. Norris | 20152 | Diabetes care among urban women in Soweto, South Africa: a qualitative study | Subject |
|  | J. O. Adeleye, N. O. Agada, W. O. Balogun, O. R. Adetunji and H. O. Onyegbutulem | 2006 | Diabetes care in Nigeria: time for a paradigm shift | Subject |
|  | K. Crabtree, N. Sherrer, T. Rushton, A. Willig, A. Agne, T. Shelton and A. Cherrington | 2015 | Diabetes connect: African American men's preferences for a community-based diabetes management program | Subject |
|  | C. Price, D. Shandu and G. Gill | 2007 | Diabetes education and empowerment: lessons from rural South Africa | Subject |
|  | https://onlinelibrary.wiley.com/doi/10.1002/pdi.1101/full | 2007 | Diabetes education and empowerment: lessons from rural South Africa | Repetition |
|  | J. J. Gagliardino, J.-M. Chantelot, C. Domenger, H. Ilkova, A. Ramachandran, G. Kaddaha, J. Claude Mbanya, J. Chan and P. Aschner | 2019 | Diabetes education and health insurance: How they affect the quality of care provided to people with type 1 diabetes in Latin America. Data from the International Diabetes Mellitus Practices Study (IDMPS) | Subject |
|  | O. A. Rashed, H. A. Sabbah, M. Z. Younis, A. Kisa and J. Parkash | 2016 | Diabetes education program for people with type 2 diabetes: An international perspective | Subject |
|  | E. G. O'Hara, B. Nuche-Berenguer, N. K. Kirui, S. Y. Cheng, P. M. Chege et al. | 2016 | Diabetes in rural Africa: what can Kenya show us? | Subject |
|  | M. Azevedo and S. Alla | 2008 | Diabetes in Sub-Saharan Africa: Kenya, Mali, Mozambique, Nigeria, South Africa and Zambia | Subject |
|  | T. Famakinwa, O. Oluwatosin, J. Faronbi, F. Faremi, A. Ogunleye et al. | 2022 | Diabetes knowledge and self-care practices among people living with type 2 diabetes mellitus in a diabetes clinic in Southwestern Nigeria | methods |
|  | J. Njeru, C. Formea, A. Osman, M. Goodson, A. Hared et al. | 2016 | Diabetes Knowledge, Attitudes and Behaviors Among Somali and Latino Immigrants | Population |
|  | J. W. Njeru, C. M. Formea, A. Osman, M. Goodson, A. Hared et al. | 2016 | Diabetes Knowledge, Attitudes and Behaviors Among Somali and Latino Immigrants | Repetition |
|  | J. W. Njeru, C. M. Formea, A. Osman, M. Goodson, A. Hared et al. | 2016 | Diabetes Knowledge, Attitudes and Behaviors Among Somali and Latino Immigrants | Repetition |
|  | G. Rafique, S. I. Azam and F. White | 2008 | Diabetes knowledge, beliefs and practices among people with diabetes attending a university hospital in Karachi, Pakistan | Setting |
|  | A. L. Kasper, L. A. Myers, P. N. Carlson, R. F. Johnson, J. L. Schultz et al. | 2022 | Diabetes Management for Community Paramedics: Development and Implementation of a Novel Curriculum | Subject |
|  | S. D. Navaneethan, S. Zoungas, M. L. Caramori, J. C. N. Chan, H. J. L. Heerspink, C. Hurst, A. Liew, E. D. Michos, W. A. Olowu, T. Sadusky, N. Tandon, K. R. Tuttle, C. Wanner, K. G. Wilkens, J. C. Craig, D. J. Tunnicliffe, M. Tonelli, M. Cheung, A. Earley, P. Rossing, I. H. de Boer and K. Khunti | 2023 | Diabetes Management in Chronic Kidney Disease: Synopsis of the KDIGO 2022 Clinical Practice Guideline Update | Subject |
|  | R. BeLue, K. Ndiaye, P. Y. Miranda, F. Ndao and P. Canagarajah | 2018 | Diabetes management in Senegalese families: A dyadic-narrative illustration | Subject |
|  | H. Berhanu, G. T. Feyissa, D. Geleta and M. Sudhakar | 2022 | Diabetes mellitus self-management education at Jimma University Medical Center: evidence-based implementation project | methods |
|  | M. A. Pienaar and M. Reid | 2021 | A diabetes peer support intervention: patient experiences using the Mmogo-method® | Subject |
|  | T. Kassahun, H. Gesesew, L. Mwanri and T. Eshetie | 2018 | Diabetes related knowledge, self-care behaviours and adherence to medications among diabetic patients in Southwest Ethiopia: a cross-sectional survey | methods |
|  | T. Halliburton | 2017 | Diabetes Risk Factor Assessment within a Rural Farming Community on Maryland's Eastern Shore | Setting |
|  | B. Kalayou Kidanu, D. Asrat, K. Alemayoh Bayeray and G. Haftu Berhe | 2012 | Diabetes self care practices and associated factors among type 2 diabetic patients in Tikur Anbessa Specialized Hospital, Addis Ababa, Ethiopia - a cross sectional study | methods |
|  | B. Smide, L. Ekman and K. Wikblad | 2002 | Diabetes self-care and educational needs in Tanzanian and Swedish diabetic patients: a cross-cultural study | Setting |
|  | G. Molalign Takele, M. A. Weharei, H. T. Kidanu, K. G. Gebrekidan and B. G. Gebregiorgis | 2021 | Diabetes self-care practice and associated factors among type 2 diabetic patients in public hospitals of Tigray regional state, Ethiopia: A multicenter study | methods |
|  | G. Molalign Takele, M. A. Weharei, H. T. M. Kidanu, K. G. Gebrekidan and B. G. Gebregiorgis | 2021 | Diabetes self-care practice and associated factors among type 2 diabetic patients in public hospitals of Tigray regional state, Ethiopia: A multicenter study | Repetition |
|  | B. A. Binhardi, C. R. de Souza Teixeira, B. de Almeida-Pititto, F. Barbosa-Junior et al. | 2021 | Diabetes self-care practices and resilience in the Brazilian COVID-19 pandemic: results of a web survey: DIABETESvid | Subject |
|  | F. B. Hailu, A. Moen and P. Hjortdahl | 2019 | Diabetes Self-Management Education (DSME) - Effect on Knowledge, Self-Care Behavior, and Self-Efficacy Among Type 2 Diabetes Patients in Ethiopia: A Controlled Clinical Trial | methods |
|  | C. M. Murray and B. R. Shah | 2016 | Diabetes self-management education improves medication utilization and retinopathy screening in the elderly | Subject |
|  | E. Kumah, G. Otchere, S. E. Ankomah, A. Fusheini, C. Kokuro, K. Aduo-Adjei and J. A Amankwah | 2021 | Diabetes self-management education interventions in the WHO African Region: A scoping review | Review |
|  | J. De Man, J. Aweko, M. Daivadanam, H. M. Alvesson, P. Delobelle et al. | 2019 | Diabetes self-management in three different income settings: Cross-learning of barriers and opportunities | Setting |
|  | J. De Man, J. Aweko, M. Daivadanam, H. M. Alvesson, P. Delobelle et al. | 2019 | Diabetes self-management in three different income settings: Cross-learning of barriers and opportunities | Repetition |
|  | T. Deakin | 2007 | Diabetes self-management within less affluent populations | Repetition |
|  | T. Steyl | 2017 | Diabetes self-management: a post-intervention evaluation of challenges experienced in a low socio-economic community | Subject |
|  | R. G. Kent de Grey, C. A. Berg, M. S. Campbell, A. K. Munion, K. Luyckx et al. | 2022 | Diabetes support from romantic partners during early emerging adulthood | Subject |
|  | J. Nielsen, S. K. Bahendeka, I. C. Bygbjerg, D. W. Meyrowitsch and S. R. Whyte | 2016 | Diabetes Treatment as "Homework": Consequences for Household Knowledge and Health Practices in Rural Uganda | Subject |
|  | J. Nielsen, S. K. Bahendeka, I. C. Bygbjerg, D. W. Meyrowitsch and S. R. Whyte | 2016 | Diabetes Treatment as “Homework” | Subject |
|  | K. C. DeCoste and L. K. Scott | 2004 | Diabetes update: promoting effective disease management | Subject |
|  | O. M. T. Elseed, I. S. Al-Busaidi and T. A. Abdallah | 2021 | Diabetes-related Foot and Lower Limb Complications and Foot Self-Care Practices in Khartoum, Sudan: A Cross-sectional Study | Subject |
|  | K. Raymaekers, V. S. Helgeson, S. Prikken, J. Vanhalst, P. Moons, E. Goossens, C. A. Berg and K. Luyckx | 2021 | Diabetes-specific friend support in emerging adults with type 1 diabetes: Does satisfaction with support matter? | Subject |
|  | A. Wallin, M. Löfvander and G. Ahlström | 2007 | Diabetes: a cross-cultural interview study of immigrants from Somalia | Subject |
|  | A.-M. Wallin, M. Löfvander and G. Ahlström | 2007 | Diabetes: a cross-cultural interview study of immigrants from Somalia | Repetition |
|  | A. M. Wallin, M. Löfvander and G. Ahlström | 2007 | Diabetes: a cross-cultural interview study of immigrants from Somalia | Repetition |
|  | C. Wimer, D. Shipman and L. Lea | 2017 | Diabetes: Health Literacy Education Improves Veteran Outcomes | Subject |
|  | E. O. Achigbu, R. N. Oputa, K. I. Achigbu and I. U. Ahuche | 2015 | Diabetes: knowledge, psychosocial impact, and attitude of patients | Subject |
|  | O. O. Desalu, F. K. Salawu, A. K. Jimoh, A. O. Adekoya, O. A. Busari and A. B. Olokoba | 2011 | Diabetic foot care: self reported knowledge and practice among patients attending three tertiary hospital in Nigeria | Methods |
|  | N. Hirpha, R. Tatiparthi and T. Mulugeta | 2020 | Diabetic Foot Self-Care Practices Among Adult Diabetic Patients: A Descriptive Cross-Sectional Study | Methods |
|  | L. G. Gebrekirstos, M. T. Abadi, M. H. Gebremedhin, E. A. Lake and T. B. Wube | 2022 | Diabetic Foot Ulcer Among Adults Attending Follow-Up Diabetes Clinics in Wolaita Zone, Southern Ethiopia: An Unmatched, Case-Control Study | Methods |
|  | F. S. Chiwanga and M. A. Njelekela | 2015 | Diabetic foot: prevalence, knowledge, and foot self-care practices among diabetic patients in Dar es Salaam, Tanzania - a cross-sectional study | Subject |
|  | Y. G. Tefera, B. M. Gebresillassie, Y. K. Emiru, R. Yilma, F. Hafiz, H. Akalu and A. A. Ayele | 2020 | Diabetic health literacy and its association with glycemic control among adult patients with type 2 diabetes mellitus attending the outpatient clinic of a university hospital in Ethiopia | Methods |
|  | S. James, K. Annetts, T. Frakking, M. Broadbent, J. Waugh, L. Perry, J. Lowe and S. Clark | 2021 | Diabetic ketoacidosis presentations in a low socio-economic area: are services suitable? | Subject |
|  | C. F. Otieno, J. K. Kayima, E. O. Omonge and G. O. Oyoo | 2005 | Diabetic ketoacidosis: risk factors, mechanisms and management strategies in sub-Saharan Africa: a review | Review |
|  | B. R. Shah, J. E. Hux, A. Laupacis, B. Z. Mdcm, P. C. Austin and C. van Walraven | 2005 | Diabetic patients with prior specialist care have better glycaemic control than those with prior primary care | Subject |
|  | T. Aklilu, D. Hiko, M. A. Mohammed and N. H. Dekema | 2014 | Diabetic Patients' Knowledge of Their Disease, Therapeutic Goals, and Self-management: Association With Goal Attainment at Dessie Referral Hospital, Ethiopia | Methods |
|  | A. Teshager, H. Desta, M. A. Mohammed and N. H. Dekema | 2014 | Diabetic Patients' Knowledge of Their Disease, Therapeutic Goals, and Self-management: Association With Goal Attainment at Dessie Referral Hospital, Ethiopia | Population |
|  | O. V. Adeniyi, P. Yogeswaran, G. Wright and B. Longo-Mbenza | 2015 | Diabetic patients' perspectives on the challenges of glycaemic control | Population |
|  | B. Saddik and N. Al-Dulaijan | 2015 | Diabetic patients' willingness to use tele-technology to manage their disease - A descriptive study | Subject |
|  | N. N. Kabedi, D. L. Kayembe, C. K. Kalobu, E. Mafuta and J. C. Mwanza | 2019 | Diabetic retinopathy in Congolese young adult | Subject |
|  | L. Zerihun Sahile, M. Benayew Shifraew and M. Zerihun Sahile | 2021 | Diabetic Self-Care Knowledge and Associated Factors Among Adult Diabetes Mellitus Patients on Follow-Up Care at North Shewa Zone Government Hospitals, Oromia Region, Ethiopia, 2020 | methods |
|  | P. D. J. Sturm, P. Moodley, G. Nzimande, R. Balkistan, C. Connolly and A. W. Sturm | 2002 | Diagnosis of bacterial vaginosis on self-collected vaginal tampon specimens | Subject |
|  | A. Guillaume and S. Rey | 1988 | [Diarrheal morbidity: what therapeutic recourses?] | Subject |
|  | S. C. Palmer, C. S. Hanson, J. C. Craig, G. F. M. Strippoli, M. Ruospo, et al. | 2015 | Dietary and fluid restrictions in CKD: a thematic synthesis of patient views from qualitative studies | Subject |
|  | M. Schure, R. T. Goins, J. Jones, B. Winchester and V. Bradley | 2019 | Dietary Beliefs and Management of Older American Indians With Type 2 Diabetes | Setting |
|  | A. F. Gebeyehu, F. Berhane and R. M. Yimer | 2022 | Dietary knowledge and practice and its associated factors among type 2 diabetes patients on follow-up at public hospitals of Dire Dawa, Eastern Ethiopia | Methods |
|  | J. Kiguli, H. M. Alvesson, R. W. Mayega, F. X. Kasujja, A. Muyingo, B. Kirunda, et al. | 2019 | Dietary patterns and practices in rural eastern Uganda: Implications for prevention and management of type 2 diabetes | Methods |
|  | J. Kiguli, H. M. Alvesson, R. W. Mayega, F. X. Kasujja, A. Muyingo, B. Kirunda, et al. | 2019 | Dietary patterns and practices in rural eastern Uganda: implications for prevention and management of type 2 diabetes | Repetition |
|  | N. Mathe, P. T. Pisa, J. A. Johnson and S. T. Johnson | 2016 | Dietary Patterns in Adults with Type 2 Diabetes Predict Cardiometabolic Risk Factors | Subject |
|  | M. Jemal, A. Argaw, A. Taye, T. Sintayehu and S. Kedir | 2022 | Dietary self-care and associated factors among diabetic patients in Jimma University Medical Centre, South West Ethiopia; A path analysis | methods |
|  | J. Musa, A. Alemayehu, T. Abonesh, S. Tsion and K. Shemsu | 2022 | Dietary self-care and associated factors among diabetic patients in Jimma University Medical Centre, South West Ethiopia; A path analysis | Repetition |
|  | https://journals.plos.org/plosone/article?id=10.1371/journal.pone.0273074 | 2022 | Dietary self-care and associated factors among diabetic patients in Jimma University Medical Centre, South West Ethiopia; A path analysis | Repetition |
|  | H. C. Janisse, N. Cakan, D. Ellis and K. Brogan | 2011 | Dietary vitamin D intake among high-risk adolescents with insulin dependent diabetes | Subject |
|  | L. A. DiMarco, B. C. Ramger, C. E. Cook, G. P. Howell, A. M. Serrani, D. L. Givens et al | 2019 | Differences in Characteristics and Downstream Drug Use Among Opioid‐Naïve and Prior Opioid Users with Low Back Pain | Subject |
|  | E. Mufunda, B. Albin and K. Hjelm | 20122 | Differences in health and illness beliefs in zimbabwean men and women with diabetes | Subject |
|  | M. L. Taylor, E. E. Thomas, K. Vitangcol, W. Marx, K. L. Campbell, L. J. Caffery et al. | 2022 | Digital health experiences reported in chronic disease management: An umbrella review of qualitative studies | Subject |
|  | D. E. Nkhoma, C. J. Soko, P. Bowrin, Y. B. Manga, D. Greenfield, M. Househ et al. | 2021 | Digital interventions self-management education for type 1 and 2 diabetes: A systematic review and meta-analysis | Review |
|  | J. Goldthorpe, T. Allen, J. Brooks, E. Kontopantelis, F. Holland, C. Moss, D. J. et al. | 2022 | Digital Interventions Supporting Self-care in People With Type 2 Diabetes Across Greater Manchester (Greater Manchester Diabetes My Way): Protocol for a Mixed Methods Evaluation | Subject |
|  | A. Farmer, K. Bobrow, N. Leon, N. Williams, E. Phiri, H. Namadingo, S. et al. | 2021 | Digital messaging to support control for type 2 diabetes (StAR2D): a multicentre randomised controlled trial | Subject |
|  | J. Hearn, S. Wali, P. Birungi, J. A. Cafazzo, I. Ssinabulya, A. R. Akiteng et al. | 2022 | A digital self-care intervention for Ugandan patients with heart failure and their clinicians: User-centred design and usability study | Subject |
|  | J. D. Tucker, J. Hocking, D. Oladele and C. Estcourt | 2022 | Digital sexually transmitted infection and HIV services across prevention and care continuums: evidence and practical resources | Subject |
|  | H. Elrayah-Eliadarous, K. Yassin, M. Eltom, S. Abdelrahman, R. Wahlström and C. G. Ostenson | 2010 | Direct costs for care and glycaemic control in patients with type 2 diabetes in Sudan | Subject |
|  | R. Uwakwe and I. Modebe | 2007 | Disability and care-giving in old age in a Nigerian community | Subject |
|  | M. Mac-Seing, C. Zarowsky, M. Yuan and K. Zinszer | 2022 | Disability and sexual and reproductive health service utilisation in Uganda: an intersectional analysis of demographic and health surveys between 2006 and 2016 | Subject |
|  | https://link.springer.com/article/10.1186/s12889-022-12708-w | 2022 | Disability and sexual and reproductive health service utilisation in Uganda: an intersectional analysis of demographic and health surveys between 2006 and 2016 | Repetition |
|  | P. Hakkarainen, L. Moilanen, V. Hänninen, K. Räsänen and F. Munir | 2017 | Disclosure of Type 1 diabetes at work among Finnish workers | Subject |
|  | A. Santamato, S. Facciorusso, S. Spina, N. Cinone, C. Avvantaggiato, L. Santoro rt al. | 2021 | Discontinuation of botulinum neurotoxin type-A treatment during COVID-19 pandemic: an Italian survey in post stroke and traumatic brain injury patients living with spasticity | Subject |
|  | M. K. Kumwenda, E. L. Corbett, J. Chikovore, M. Phiri, D. Mwale, A. T. et al. | 2018 | Discordance, Disclosure and Normative Gender Roles: Barriers to Couple Testing Within a Community-Level HIV Self-Testing Intervention in Urban Blantyre, Malawi | Subject |
|  | S. James, L. Perry, R. Gallagher and J. Lowe | 2020 | A discussion of healthcare support for adolescents and young adults with long-term conditions: Current policy and practice and future opportunities | Subject |
|  | B. E. Bassey, F. Braka, F. Shuaib, R. Banda, S. G. Tegegne, J. M. Ticha et al. | 2018 | Distribution pattern of poliovirus potentially infectious materials in the phase 1b medical laboratories containment in conformity with the global action plan III | Subject |
|  | K. Luyckx, M. Verschueren, N. Palmeroni, E. R. Goethals, I. Weets and L. Claes | 2019 | Disturbed Eating Behaviors in Adolescents and Emerging Adults With Type 1 Diabetes: A One-Year Prospective Study | Population |
|  | F. A. Yehualashet, E. T. Tegegne, A. D. Ayele and W. W. Takele | 2021 | Do adult patients with diabetes mellitus living in rural part of Ethiopia and having poor social support have disproportionately poor self-care practice? A systematic review and meta-analysis | Subject |
|  | K. Wambui Charity, A. M. V. Kumar, S. G. Hinderaker, P. Chinnakali, S. D. Pastakia and J. Kamano | 2016 | Do diabetes mellitus patients adhere to self-monitoring of blood glucose (SMBG) and is this associated with glycemic control? Experiences from a SMBG program in western Kenya | Subject |
|  | R. Adisa and T. O. Fakeye | 2016 | Do diabetes-specialty clinics differ in management approach and outcome? A cross-sectional assessment of ambulatory type 2 diabetes patients in two teaching hospitals in Nigeria | Methods |
|  | J. Daher, R. Vijh, B. Linthwaite, S. Dave, J. Kim, K. Dheda, T. et al. | 2017 | Do digital innovations for HIV and sexually transmitted infections work? Results from a systematic review (1996-2017) | Subject |
|  | M. Moshabela, H. Schneider, S. M. Cleary, P. M. Pronyk and J. Eyles | 2011 | Does accessibility to antiretroviral care improve after down-referral of patients from hospitals to health centres in rural South Africa? | Subject |
|  | https://www.tandfonline.com/loi/raar20 | 2011 |  | Invalid |
|  | N. Musinguzi, T. Muwonge, K. Thomas, J. M. Baeten, D. R. Bangsberg and J. E. Haberer | 2016 | Does Adherence Change When No One is Looking? Comparing Announced and Unannounced Tenofovir Levels in a PrEP Trial | Subject |
|  | R. Rana, J. Gow, C. Moloney, A. King, G. Keijzers, G. Beccaria and A. Mullens | 2022 | Does distance to hospital affect emergency department presentations and hospital length of stay among chronic obstructive pulmonary disease patients? | Subject |
|  | L. Melkamu, R. Berhe and S. Handebo | 2021 | Does Patients' Perception Affect Self-Care Practices? The Perspective of Health Belief Model | Methods |
|  | R. Jones, H. Muyinda, G. Nyakoojo, B. Kirenga, W. Katagira and J. Pooler | 2018 | Does pulmonary rehabilitation alter patients' experiences of living with chronic respiratory disease? A qualitative study | Subject |
|  | M. M. Werfalli, S. Z. Kalula, K. Manning and N. S. Levitt | 2020 | Does social support effect knowledge and diabetes self-management practices in older persons with Type 2 diabetes attending primary care clinics in Cape Town, South Africa? | Subject |
|  | R. Ratnayake, A. Wittcoff, J. Majaribu, J.-P. Nzweve, L. Katembo, K et al. | 2021 | Early Experiences in the Integration of Non-communicable Diseases into Emergency Primary Health Care, Beni Region, Democratic Republic of the Congo | Subject |
|  | R. Gaines and C. Missiuna | 2007 | Early identification: are speech/language-impaired toddlers at increased risk for Developmental Coordination Disorder? | Subject |
|  | E. Nasuuna, F. Namimbi, P. A. Muwanguzi, D. Kabatesi, M. Apolot et al. | 2022 | Early observations from the HIV self-testing program among key populations and sexual partners of pregnant mothers in Kampala, Uganda: a cross sectional study | Subject |
|  | https://journals.plos.org/globalpublichealth/article?id=10.1371/journal.pgph.0000120 | 2022 | Early observations from the HIV self-testing program among key populations and sexual partners of pregnant mothers in Kampala, Uganda: a cross sectional study | Repetition |
|  | S. L. Lim, S. P. Chan, K. Y. Lee, A. Ching, R. J. Holden et al. | 2019 | An East-West comparison of self-care barriers in heart failure | Subject |
|  | H. Elrayah, M. Eltom, A. Bedri, A. Belal, H. Rosling and C. Östenson | 2005 | Economic burden on families of childhood type 1 diabetes in urban Sudan | Subject |
|  | H. Elrayah, M. Eltom, A. Bedri, A. Belal, H. Rosling and C.-G. Ostenson | 2005 | Economic burden on families of childhood type 1 diabetes in urban Sudan | Repetition |
|  | H. Elrayah, M. Eltom, A. Bedri, A. Belal, H. Rosling and C. G. Ostenson | 2005 | Economic burden on families of childhood type 1 diabetes in urban Sudan | Repetition |
|  | K. E. Lamb, D. Crawford, L. E. Thornton, S. M. S. Islam, R. Maddison and K. Ball | 2021 | Educational differences in diabetes and diabetes self-management behaviours in WHO SAGE countries | Subject |
|  | https://link.springer.com/article/10.1186/s12889-021-12131-7 | 2021 | Educational differences in diabetes and diabetes self-management behaviours in WHO SAGE countries | Repetition |
|  | K. E. Lamb, D. Crawford, L. E. Thornton, S. M. Shariful Islam, R. Maddison and K. Ball | 2021 | Educational differences in diabetes and diabetes self-management behaviours in WHO SAGE countries | Repetition |
|  | L. L. Oakley, D. R, A. Namara, B. Sahu, I. P. Nadal, Y. Ana, H. el. | 2021 | Educational films for improving screening and self-management of gestational diabetes in India and Uganda (GUIDES): study protocol for a cluster-randomised controlled trial | Subject |
|  | S. Liu, D. Fu and A. Bi | 2009 | The effect analysis of team management model in the diabetes management in communities | Subject |
|  | Y. Li, R. Li, W. Zhong, Q. Yang, L. Shi and Y. Ruan | 2015 | Effect assessment of type 2 diabetes self-management in selected communities in Shanghai | Subject |
|  | O. Carrasquillo, C. Lebron, Y. Alonzo, L. Hua, A. Chang, S. et al. | 2017 | Effect of a Community Health Worker Intervention Among Latinos With Poorly Controlled Type 2 Diabetes: The Miami Healthy Heart Initiative Randomized Clinical Trial | Subject |
|  | O. Carrasquillo, C. Lebron, Y. Alonzo, H. Li, A. Chang and S. Kenya | 2017 | Effect of a Community Health Worker Intervention Among Latinos With Poorly Controlled Type 2 Diabetes: The Miami Healthy Heart Initiative Randomized Clinical Trial | Repetition |
|  | UKoha-kalu BO, Adibe MO, Ukwe CV. | 2021 | Effect of a pharmacist intervention on self management practices among hypertensive-diabetic patients receiving care in a Nigerian tertiary hospital | Subject |
|  |  |  |  |  |
|  | G. Dessie, S. Burrowes, H. Mulugeta, D. Haile, A. Negess et al. | 2021 | Effect of a self-care educational intervention to improve self-care adherence among patients with chronic heart failure: a clustered randomized controlled trial in Northwest Ethiopia | Repetition |
|  | https://link.springer.com/article/10.1186/s12872-021-02170-8 | 2021 | Effect of a self-care educational intervention to improve self-care adherence among patients with chronic heart failure: a clustered randomized controlled trial in Northwest Ethiopia | Repetition |
|  | C. J. Smith, M. J. Abrahamson, P. A. Henshilwood and F. Bonnici | 1987 | The effect of an intensive education programme on the glycaemic control of type I diabetic patients | Subject |
|  | A. Yildirim, R. H. Aşilar, N. Bakar and N. Demir | 2013 | Effect of anxiety and depression on self-care agency and quality of life in hospitalized patients with chronic obstructive pulmonary disease: a questionnaire survey | Subject |
|  | P. Zhang, J. Gao, Y. Wang, Q. Sun and X. Sun | 2019 | Effect of chronic disease self-management program on the quality of life of HIV-infected men who have sex with men: an empirical study in Shanghai, China | Setting |
|  | D. Barry, A. H. Frew, H. Mohammed, B. F. Desta, L. Tadesse, Y. et al. | 2014 | The Effect of Community Maternal and Newborn Health Family Meetings on Type of Birth Attendant and Completeness of Maternal and Newborn Care Received During Birth and the Early Postnatal Period in Rural Ethiopia | Setting |
|  | B. B. Bekele, S. Negash, B. Bogale, M. Tesfaye, D. Getachew et al. | 2021 | Effect of diabetes self-management education (DSME) on glycated hemoglobin (HbA1c) level among patients with T2DM: Systematic review and meta-analysis of randomized controlled trials | Subject |
|  | C. W. Gathu, J. Shabani, N. Kunyiha and R. Ratansi | 2018 | Effect of diabetes self-management education on glycaemic control among type 2 diabetic patients at a family medicine clinic in Kenya: A randomised controlled trial | Subject |
|  | J. Huang, T. Zhang, L. Wang, D. Guo, S. Liu et al. | 2019 | The effect of family doctor-contracted services on noncommunicable disease self-management in Shanghai, China | Setting |
|  | https://onlinelibrary.wiley.com/toc/10991751/2019/34/3 | 2019 |  | Invalid |
|  | H. Yu, P. Zhang, X. Wang, Y. Wang and B. Zhang | 2019 | Effect of health education based on behavioral change theories on self-efficacy and self-management behaviors in patients with chronic heart failure | Subject |
|  | J. Chen, L. Tong, P. Liu, L. Xue and E. Chen | 2021 | Effect of intensive intervention for improving the referral rate among preschoolers with vision abnormalities | Subject |
|  | https://www.cjsh.org.cn/en/article/doi/10.16835/j.cnki.1000-9817.2021.12.032 | 2021 | Effect of intensive intervention for improving the referral rate among preschoolers with vision abnormalities | Repetition |
|  | W. Shi, X. Gao and Q. Wei | 2019 | Effect of intervention based on protection motivation theory on patients with gestational diabetes | Subject |
|  | R. O. Ajiboye, N. A. Okafor, T. E. Olajide and E. O. Tosin | 2021 | Effect of Nursing Intervention on Knowledge and Practice of Salt and Diet Modification among Hypertensive Patients in a General Hospital South-West Nigeria | Subject |
|  | P. MacPherson, D. G. Lalloo, E. L. Webb, H. Maheswaran, A. T. Choko, et al. | 2014 | Effect of optional home initiation of HIV care following HIV self-testing on antiretroviral therapy initiation among adults in Malawi: a randomized clinical trial | Subject |
|  | N. O. Frere, S. S. A. Soliman, M. A. Foda, T. K. Eyada and N. E. S. Saad | 2019 | Effect of patient education on metabolic syndrome components among females in Zagazig University outpatient clinics, Egypt: An intervention study | Subject |
|  | S. Sap, E. Kondo, E. Sobngwi, R. Mbono, S. Tatah et al. | 2019 | Effect of patient education through a social network in young patients with type 1 diabetes in a Sub-Saharan context | Subject |
|  | H. A. Tilahun, M. Damen Haile and A. O. Tsui | 2012 | Effect of perceived stigma on adherence to highly active antiretroviral therapy and self-confidence to take medication correctly in Addis Ababa, Ethiopia | Subject |
|  | https://www.tandfonline.com/loi/whiv20 | 2012 |  | Invalid |
|  | J. Van Olmen, G. Kegels, C. Korachais, J. de Man, K. Van Ackeret al. | 20172021 | The effect of text message support on diabetes self-management in developing countries - A randomised trial | methods |
|  | L. O'Toole, D. Connolly, F. Boland and S. M. Smith | 2021 | Effect of the OPTIMAL programme on self-management of multimorbidity in primary care: a randomised controlled trial | Subject |
|  | Z. Pouresmail, F. H. Nabavi, A. Abdollahi, M. T. Shakeri and A. Saki | 2019 | Effect of Using a Simulation Device for Ostomy Self-care Teaching in Iran: A Pilot, Randomized Clinical Trial | Subject |
|  | J. Porter, C. E. Huggins, H. Truby and J. Collins | 2016 | The Effect of Using Mobile Technology-Based Methods That Record Food or Nutrient Intake on Diabetes Control and Nutrition Outcomes: A Systematic Review | Subject |
|  | M. Neuman, P. Indravudh, R. Chilongosi, M. d'Elbée, N. Desmond et al. | 2018 | The effectiveness and cost-effectiveness of community-based lay distribution of HIV self-tests in increasing uptake of HIV testing among adults in rural Malawi and rural and peri-urban Zambia: protocol for STAR (self-testing for Africa) cluster randomized evaluations | Subject |
|  | M. Neuman, P. Indravudh, R. Chilongosi, M. d'Elbée, N. Desmond et al. | 2018 | The effectiveness and cost-effectiveness of community-based lay distribution of HIV self-tests in increasing uptake of HIV testing among adults in rural Malawi and rural and peri-urban Zambia: protocol for STAR (self-testing for Africa) cluster randomized evaluations | Subject |
|  | N. Carter, P. Chahal and K. Chattopadhyay | 2021 | Effectiveness and safety of self-management interventions among adult type 2 diabetes mellitus patients in sub-Saharan Africa: a systematic review protocol | Review |
|  | Q. Lou, Q. Ye, H. Wu, Z. Wang, R. S. Ware, Y. Xiong and F. Xu | 2020 | Effectiveness of a clinic-based randomized controlled intervention for type 2 diabetes management: an innovative model of intensified diabetes management in Mainland China (C-IDM study) | Subject |
|  | https://drc.bmj.com/content/8/1/e001030 | 2020 | Effectiveness of a clinic-based randomized controlled intervention for type 2 diabetes management: an innovative model of intensified diabetes management in Mainland China (C-IDM study) | Repetition |
|  | R. J. Mash, H. Rhode, M. Zwarenstein, S. Rollnick, C. Lombard et al. | 2014 | Effectiveness of a group diabetes education programme in under-served communities in South Africa: a pragmatic cluster randomized controlled trial | Subject |
|  | B. Mash, N. Levitt, K. Steyn, M. Zwarenstein and S. Rollnick | 2012 | Effectiveness of a group diabetes education programme in underserved communities in South Africa: pragmatic cluster randomized control trial | Repetition |
|  | R. Mash, N. Levitt, K. Steyn, M. Zwarenstein and S. Rollnick | 2012 | Effectiveness of a group diabetes education programme in underserved communities in South Africa: pragmatic cluster randomized control trial | Repetition |
|  | M. Kebede, L. Christianson, Z. Khan, T. L. Heise and C. R. Pischke | 2017 | Effectiveness of behavioral change techniques employed in eHealth interventions designed to improve glycemic control in persons with poorly controlled type 2 diabetes: a systematic review and meta-analysis protocol | Subject |
|  | M. Werfalli, P. Raubenheimer, M. Engel, N. Peer, S. Kalula et al. | 2015 | Effectiveness of community-based peer-led diabetes self-management programmes (COMP-DSMP) for improving clinical outcomes and quality of life of adults with diabetes in primary care settings in low and middle-income countries (LMIC): a systematic review and meta-analysis | Subject |
|  | F. A. M. Elfakki, M. K. Elnimeiri, S. M. Assil, E. A. Frah and N. H. I. Abdalla | 2022 | Effectiveness of diabetes self-care education at primary health care centres in Saudi Arabia: A pragmatic randomized trial in Tabuk | Subject |
|  | B. B. Bekele, S. Negash, B. Bogale, M. Tesfaye, D. Getachew et al. | 2020 | The effectiveness of diabetes self-management education (DSME) on glycemic control among T2DM patients randomized control trial: systematic review and meta-analysis protocol | Subject |
|  | K. Odgers-Jewell, L. E. Ball, J. T. Kelly, E. A. Isenring, D. P. Reidlinger and R. Thomas | 2017 | Effectiveness of group-based self-management education for individuals with Type 2 diabetes: a systematic review with meta-analyses and meta-regression | Subject |
|  | A. Mogueo, C. Oga-Omenka, M. Hatem and B. Kuate Defo | 2020 | Effectiveness of interventions based on patient empowerment in the control of type 2 diabetes in sub-Saharan Africa: A review of randomized controlled trials | Subject |
|  | A. Mogueo, C. Oga-Omenka, M. Hatem and B. Kuate Defo | 2020 | Effectiveness of interventions based on patient empowerment in the control of type 2 diabetes in sub-Saharan Africa: A review of randomized controlled trials | Repetition |
|  | T. Siddharthan, S. L. Pollard, P. Jackson, N. M. Robertson, A. C. Wosu, N. Rahman et al. | 2021 | Effectiveness of low-dose theophylline for the management of biomass-associated COPD (LODOT-BCOPD): study protocol for a randomized controlled trial | Repetition |
|  | J. Goris, N. Komaric, A. Guandalini, D. Francis and E. Hawes | 2013 | Effectiveness of multicultural health workers in chronic disease prevention and self-management in culturally and linguistically diverse populations: a systematic literature review | Subject |
|  | https://www.publish.csiro.au/nid/261.htm | 2013 |  | Invalid |
|  | R. C. Nanyonga, L. A. Spies and F. Nakaggwa | 2022 | The effectiveness of nurse-led group interventions on hypertension lifestyle management: A mixed method study | Subject |
|  | R. C. Nanyonga, L. A. Spies and F. Nakaggwa | 2022 | The effectiveness of nurse‐led group interventions on hypertension lifestyle management: A mixed method study | Repetition |
|  | M. Werfalli, P. J. Raubenheimer, M. Engel, A. Musekiwa, K. Bobrow et al. | 2020 | The effectiveness of peer and community health worker-led self-management support programs for improving diabetes health-related outcomes in adults in low-and-middle-income countries: a systematic review | Subject |
|  | https://link.springer.com/content/pdf/10.1186/s13643-020-01377-8.pdf | 2020 | The effectiveness of peer and community health worker-led self-management support programs for improving diabetes health-related outcomes in adults in low-and-middle-income countries: a systematic review | Repetition |
|  | H. Walter, F. Sadeque-Iqbal, R. Ulysse, D. Castillo, A. Fitzpatrick and J. Singleton | 2015 | The effectiveness of school-based family asthma educational programs on the quality of life and number of asthma exacerbations of children aged five to 18 years diagnosed with asthma: a systematic review protocol | Subject |
|  | J. A. Aminde, N. Harris, C. Thng and B. Desbrow | 2022 | Effectiveness of self-managed home and community exercise interventions in improving physical activity, body adiposity and related health indices in adults living with HIV: a protocol for a systematic review | Subject |
|  | J. v. Olmen, G. M. Ku, M. v. Pelt, J. C. Kalobu, H. Hen et al. | 2013 | The effectiveness of text messages support for diabetes self-management: protocol of the TEXT4DSM study in the democratic Republic of Congo, Cambodia and the Philippines | Subject |
|  | J. v. Olmen, G. M. Ku, M. v. Pelt, J. C. Kalobu, H. Hen et al. | 2013 | The effectiveness of text messages support for diabetes self-management: protocol of the TEXT4DSM study in the democratic Republic of Congo, Cambodia and the Philippines | Repetition |
|  | C. S. Tey, R. E. K. Man, E. K. Fenwick, A. T. Aw, V. Drury, P. P.-C. Chiang and E. L. Lamoureux | 2019 | Effectiveness of the "living successfully with low vision" self-management program: Results from a randomized controlled trial in Singaporeans with low vision | Setting |
|  | S. Liu, A. Bi, D. Fu, H. Fu, W. Luo, X. Ma and L. Zhuang | 2012 | Effectiveness of using group visit model to support diabetes patient self-management in rural communities of Shanghai: a randomized controlled trial | Setting |
|  | T. Siddharthan, S. L. Pollard, S. A. Quaderi, A. J. Mirelman, M. K. Cárdenas, B. Kirenga et al. | 2018 | Effectiveness-implementation of COPD case finding and self-management action plans in low- and middle-income countries: global excellence in COPD outcomes (GECo) study protocol | Setting |
|  | Y. Lufei, H. Melody, B. Susan, L. Young, M. Hertzog and S. Barnason | 2016 | Effects of a home-based activation intervention on self-management adherence and readmission in rural heart failure patients: the PATCH randomized controlled trial | Subject |
|  | E. du Pon, A. van Dooren, N. Kleefstra and S. van Dulmen | 2020 | Effects of a Proactive Interdisciplinary Self-Management Program on Patient Self-Efficacy and Participation During Practice Nurse Consultations: A Randomized Controlled Trial in Type 2 Diabetes | Subject |
|  | L. A. Nelson, R. A. Greevy, A. Spieker, K. A. Wallston, T. A. Elasy, S et al. | 2021 | Effects of a Tailored Text Messaging Intervention Among Diverse Adults With Type 2 Diabetes: Evidence From the 15-Month REACH Randomized Controlled Trial | Subject |
|  | D. Cannon, N. Buys, K. B. Sriram, S. Sharma, N. Morris and J. Sun | 2016 | The effects of chronic obstructive pulmonary disease self-management interventions on improvement of quality of life in COPD patients: A meta-analysis | Subject |
|  | P. Lavikainen, M.-L. Lamidi, T. Repo, L. Inglin, J. Martikainen and T. Laatikainen | 2022 | Effects of COVID-19 Pandemic and Lockdown on Monitoring and Treatment Balance of Finnish Coronary Heart Disease and Type 2 Diabetes Patients | Subject |
|  | S. P. Choukem, D. T. Efie, S. Djiogue, F. F. Kaze, Y. Mboue-Djieka et al. | 2018 | Effects of hydroquinone-containing creams on capillary glycemia before and after serial hand washings in Africans | Subject |
|  | M. Lyu, Y. Wu and H. Li | 2019 | Effects of knowledge-attitude-practice theory model combined with nutrition and health care during pregnancy on nutritional status, self-care ability and pregnancy outcomes of pregnant women with gestational diabetes mellitus | methods |
|  | R. Gao, H. Guo, Y. Liu, Y. Pang, X. Zhang, X. Lian, T. Yu, L. Zhu and F. Li | 2022 | Effects of message framing on self-management behaviour among patients with type 2 diabetes: a randomised controlled trial protocol | methods |
|  | B. Chester, J. R. Babu, M. W. Greene and T. Geetha | 2019 | The effects of popular diets on type 2 diabetes management | Subject |
|  | https://onlinelibrary.wiley.com/doi/10.1002/dmrr.3188 | 2019 | The effects of popular diets on type 2 diabetes management | Repetition |
|  | E. du Pon, N. Kleefstra, F. Cleveringa, A. van Dooren, E. R. Heerdink and S. van Dulmen | 2019 | Effects of the Proactive Interdisciplinary Self-Management (PRISMA) Program on Online Care Platform Usage in Patients with Type 2 Diabetes in Primary Care: A Randomized Controlled Trial | Sub |
|  | E. du Pon, N. Kleefstra, F. Cleveringa, A. van Dooren, E. R. Heerdink and S. van Dulmen | 2019 | Effects of the Proactive interdisciplinary self-management (PRISMA) program on self-reported and clinical outcomes in type 2 diabetes: a pragmatic randomized controlled trial | Repetition |
|  | C.-L. Lee, C.-K. Chuang, Y.-M. Syu, H.-C. Chiu, Y.-R. Tu, Y.-T. Lo et al. | 2022 | Efficacy of Intravenous Elosulfase Alfa for Mucopolysaccharidosis Type IVA: A Systematic Review and Meta-Analysis | Subject |
|  | K. Radhakrishnan | 2012 | The efficacy of tailored interventions for self-management outcomes of type 2 diabetes, hypertension or heart disease: a systematic review | methods |
|  | J. De Groot, D. Wu, D. Flynn, D. Robertson, G. Grant and J. Sun | 2021 | Efficacy of telemedicine on glycaemic control in patients with type 2 diabetes: A meta-analysis | Subject |
|  | J. L. Cowand, D. M. Wrisley, M. Walker, B. Strasnick and J. T. Jacobson | 1998 | Efficacy of vestibular rehabilitation | Subject |
|  | E. Mustonen, I. Hörhammer, K. Patja, P. Absetz, J. Lammintakanen, M. et al. | 2021 | Eight-year post-trial follow-up of morbidity and mortality of telephone health coaching | Subject |
|  | W. B. Matuja and N. K. Ndosi | 1994 | The elderly patients as seen at Muhimbili Medical Centre, Tanzania | Subject |
|  | W. B. P. Matuja and N. K. Ndosi | 1994 | The elderly patients as seen at Muhimbili Medical Centre, Tanzania | Repetition |
|  | Y.-T. Chiang, H.-Y. Yu, F.-S. Lo, C.-W. Chen, T.-T. Huang, C.-W. Chang and P. Moons | 2020 | Emergence of a butterfly: the life experiences of type 1 diabetes Taiwanese patients during the 16-25 years old transition period | Setting |
|  | A. Ogbera and A. Adeyemi-Doro | 2011 | Emotional distress is associated with poor self care in type 2 diabetes mellitus | methods |
|  | S. M. Noman, J. Arshad, M. Zeeshan, A. U. Rehman, A. Haider, S. et al. | 20212021 | An Empirical Study on Diabetes Depression over Distress Evaluation Using Diagnosis Statistical Manual and Chi-Square Method | Subject |
|  | S. M. Noman, J. Arshad, M. Zeeshan, A. U. Rehman, A. Haider, S. et al. | 2021 | An empirical study on diabetes depression over distress evaluation using diagnosis statistical manual and chi-square method | Repetition |
|  | https://www.mdpi.com/1660-4601/18/7/3755 | 2021 | An empirical study on diabetes depression over distress evaluation using diagnosis statistical manual and chi-square method | Repetition |
|  | M. Acuña Mora, M. Saarijärvi, C. Sparud-Lundin, P. Moons and E.-L. Bratt | 2020 | Empowering Young Persons with Congenital Heart Disease: Using Intervention Mapping to Develop a Transition Program - The STEPSTONES Project | Subject |
|  | K. Diclemente-Bosco, A. Z. Weber, A. Harrison, N. Tsawe, Z. Rini et al. | 2022 | Empowerment in pregnancy: ART adherence among women living with HIV in Cape Town, South Africa | Subject |
|  | https://www.sciencedirect.com/science/article/abs/pii/S0277953622000417 | 2022 | Empowerment in pregnancy: ART adherence among women living with HIV in Cape Town, South Africa | Repetition |
|  | D. St-Cyr Tribble, F. Gallagher, L. Bell, C. Caron, P. Godbout, J. et al. | 2008 | Empowerment interventions, knowledge translation and exchange: perspectives of home care professionals, clients and caregivers | Subject |
|  | D. St-Cyr Tribble, F. Gallagher, L. Bell, C. Caron, P. Godbout, J. et al. | 2008 | Empowerment interventions, knowledge translation and exchange: perspectives of home care professionals, clients and caregivers | Repetition |
|  | M. L. Richter, W. D. F. Venter and A. Gray | 2012 | Enabling HIV self-testing in South Africa | Subject |
|  | G. Wagner, B. Ghosh-Dastidar and M. Slaughter | 2015 | Engagement in HIV Prevention Advocacy Associated with Increased Consistent Condom Use Among HIV Clients in Uganda | Subject |
|  | J. Richards, M. Nazareth, M. A. L. van Tilburg, N. Jain, L. Hart et al. | 2021 | Engagement in Household Chores in Youth With Chronic Conditions: Health care Transition Implications | Subject |
|  | [\\](file:///C:\) | 2014 | Engagement with care, substance use, and adherence to therapy in HIV/AIDS | Subject |
|  | P. K. Nicholas, S. Willard, C. Thompson, C. Dawson-Rose, I. B. Corless et al. | 2014 | Engagement with Care, Substance Use, and Adherence to Therapy in HIV/AIDS | Repetition |
|  | W.-T. Chen, D. Wantland, P. Reid, I. B. Corless, L. S. Eller, S. Iipinge, W. L. Holzemer, K. Nokes, E. Sefcik, M. Rivero-Mendez, J. Voss, P. Nicholas, J. C. Phillips, J. M. Brion, C. D. Rose, C. J. Portillo, K. Kirksey, K. M. Sullivan, M. O. Johnson, L. Tyer-Viola and A. R. Webel | 2013 | Engagement with Health Care Providers Affects Self- Efficacy, Self-Esteem, Medication Adherence and Quality of Life in People Living with HIV | Subject |
|  | \ | 2013 | Engagement with health care providers affects self-efficacy, self-esteem, medication adherence and quality of life in people living with HIV | Repetition |
|  | T. van Middelaar, C. R. L. Beishuizen, J. Guillemont, M. Barbera, E. Richard and E. P. Moll van Charante | 2018 | Engaging older people in an internet platform for cardiovascular risk self-management: a qualitative study among Dutch HATICE participants | Subject |
|  | M. E. John, P. E. Samson-Akpan, J. B. Etowa, I. I. Akpabio and E. E. John | 2016 | Enhancing self-care, adjustment and engagement through mobile phones in youth with HIV | Subject |
|  | M. E. John, P. E. Samson‐Akpan, J. B. Etowa, I. I. Akpabio and E. E. John | 2016 | Enhancing self-care, adjustment and engagement through mobile phones in youth with HIV | Repetition |
|  | L. Zhang, Y. Zeng, C. Weng, J. Yan and Y. Fang | 2019 | Epidemiological characteristics and factors influencing falls among elderly adults in long-term care facilities in Xiamen, China | Setting |
|  | R. Uwakwe, C. C. Ibeh, A. I. Modebe, E. Bo, N. Ezeama et al. | 2009 | The epidemiology of dependence in older people in Nigeria: prevalence, determinants, informal care, and health service utilization. A 10/66 Dementia Research Group cross-sectional survey | Subject |
|  | P. H. Park, C. K. Wambui, S. Atieno, J. R. Egger, L. Misoi et al. | 2015 | Erratum. Improving diabetes management and cardiovascular risk factors through peer-led self-management support groups in western Kenya. Diabetes Care 2015;38:e110-e111 | methods |
|  | S. Stewart, B. Riegel, C. Boyd, Y. Ahamed, D. R. Thompson et al. | 2016 | Establishing a pragmatic framework to optimise health outcomes in heart failure and multimorbidity (ARISE-HF): A multidisciplinary position statement | Subject |
|  | C. E. Hall, A. B. Hall, J. Mallya, P. Courtright and G. Kok | 2022 | Establishing a screening programme for diabetic retinopathy in Kilimanjaro Region, Tanzania using intervention mapping | Subject |
|  | S. N. Mbalinda, S. Bakeera-Kitaka, D. L. Amooti, E. N. Magongo, P. Musoke and D. K. Kaye | 2021 | Ethical challenges of the healthcare transition to adult antiretroviral therapy (ART) clinics for adolescents and young people with HIV in Uganda | Population |
|  | L. E. Bain, C. M. Ditah, P. K. Awah and N. C. Ekukwe | 2016 | Ethical implications of HIV self-testing: the game is far from being over | Subject |
|  | L. Allais and F. Venter | 2014 | The ethical, legal and human rights concerns raised by licensing HIV self-testing for private use | Subject |
|  | C. O. Oyejide and E. A. Oke | 1995 | An ethnographic study of acute respiratory infections in four local government areas of Nigeria | Subject |
|  | W. J. Fokkens, V. J. Lund, C. Hopkins, P. W. Hellings, R. Kern et al. | 202 | European Position Paper on Rhinosinusitis and Nasal Polyps 2020 | Subject |
|  | S. Kalra, A. K. Das, M. P. Baruah, A. G. Unnikrishnan, A. Dasgupta et al. | 2019 | Euthymia in Diabetes: Clinical Evidence and Practice-Based Opinion from an International Expert Group | Subject |
|  | C. Trujillo, G. Ferrari, G. Ngoga, A. McLaughlin, J. Davies et al. | 2022 | Evaluating implementation of Diabetes Self-Management Education in Maryland County, Liberia: protocol for a pilot prospective cohort study | Subject |
|  | K. Geiger, A. Bergman and J. E. Farley | 2021 | Evaluating Integrated Care for People Living With HIV and Multidrug-Resistant Tuberculosis in South Africa: A Case-Based Approach Using the Chronic Care Model | Subject |
|  | F. Sieverink, S. Kelders, A. Braakman-Jansen and J. van Gemert-Pijnen | 2019 | Evaluating the implementation of a personal health record for chronic primary and secondary care: a mixed methods approach | Subject |
|  | R. Mash, D. Schouw and A. E. Fischer | 2022 | Evaluating the Implementation of the GREAT4Diabetes WhatsApp Chatbot to Educate People With Type 2 Diabetes During the COVID-19 Pandemic: Convergent Mixed Methods Study | Subject |
|  | A. Bonner, K. Gillespie, K. L. Campbell, K. Corones-Watkins, B. Hayeset al. | 2018 | Evaluating the prevalence and opportunity for technology use in chronic kidney disease patients: a cross-sectional study | Subject |
|  | Y. Zhang, S. Liu, X. Sheng, J. Lou, H. Fu and X. Sun | 2019 | Evaluation of a community-based hypertension self-management model with general practitioners | Subject |
|  | J. Duncan-Carnesciali, B. C. Wallace and M. Odlum | 2018 | An Evaluation of a Diabetes Self-Management Education (DSME) Intervention Delivered Using Avatar-Based Technology: Certified Diabetes Educators’ Ratings and Perceptions | Subject |
|  | J. Afemikhe and J. Chipps | 2015 | An evaluation of a multidisciplinary patient centred Type 2 diabetes self-management education programme in Edo State, Nigeria | Subject |
|  | E. U. Nwose, B. Ekotogbo, C. N. Ogbolu, M. Mogbusiaghan, O. Agofure and E. O. Igumbor | 2019 | Evaluation of ADL and BMI in the management of diabetes mellitus at secondary and tertiary health facilities | Subject |
|  | P. Bhattacharjee, D. Rego, H. Musyoki, M. Becker, M. Pickles et al. | 2019 | Evaluation of community-based HIV self-testing delivery strategies on reducing undiagnosed HIV infection, and improving linkage to prevention and treatment services, among men who have sex with men in Kenya: a programme science study protocol | Sub |
|  | W. Belete, T. Deressa, A. Feleke, T. Menna, T. Moshago et al. | 2019 | Evaluation of diagnostic performance of non-invasive HIV self-testing kit using oral fluid in Addis Ababa, Ethiopia: A facility-based cross-sectional study | Subject |
|  | M. O. Adibe, C. Anosike, S. O. Nduka and A. Isah | 2018 | Evaluation of Health Status of Type 2 Diabetes Outpatients Receiving Care in a Tertiary Hospital in Nigeria | Subject |
|  | L. O. Nwoye, P. E. Uwagboe and G. U. Madubuko | 1988 | Evaluation of home-made salt-sugar oral rehydration solution in a rural Nigerian population | Subject |
|  | H. Kahsay, B. Fantahun, T. Nedi and G. T. Demoz | 2019 | Evaluation of Hypoglycemia and Associated Factors among Patients with Type 1 Diabetes on Follow-Up Care at St. Paul's Hospital Millennium Medical College, Addis Ababa, Ethiopia | Subject |
|  | C. Harvey, J. Palmer, D. Hegney, E. Willis, A. Baldwin, C et al. | 2019 | The evaluation of nurse navigators in chronic and complex care | Subject |
|  | Q. Wu, J. Jiang, Y. Zhang, L. Lu, L. Tang, Q. Zhao, W. Jiang and B. Xu | 2022 | Evaluation of quality of life in 975 patients with pulmonary tuberculosis within 3 years after treatment in Shanghai | Subject |
|  | https://www.sjpm.org.cn/article/doi/10.19428/j.cnki.sjpm.2022.21312?pageType=en | 2022 | Evaluation of quality of life in 975 patients with pulmonary tuberculosis within 3 years after treatment in Shanghai | Repetition |
|  | M. Weledegebriel, A. Mulugeta and A. Hailu | 2021 | Evaluation of Self-Care Practice and Its Associated Factors in Adult Diabetic Patients, Ayder Diabetic Clinic, Mekelle, Ethiopia | methods |
|  | T. C. Mosha and H. Rashidi | 2009 | Evaluation of self-care practices and emotional distress among people with Type 2 diabetes mellitus in Dar es Salaam, Tanzania | methods |
|  | T. C. E. Mosha and H. Rashidi | 2009 | Evaluation of self-care practices and emotional distress among people with type 2 diabetes mellitus in Dar Es Salaam, Tanzania | methods |
|  | https://www.informaworld.com/smpp/content˜content=a909178388˜db=all˜order=page | 2012 |  | Invalid |
|  | V. Angwenyi, J. Bunders-Aelen, B. Criel, J. V. Lazarus and C. Aantjes | 2020 | An evaluation of self-management outcomes among chronic care patients in community home-based care programmes in rural Malawi: a 12-month follow-up study | methods |
|  | https://onlinelibrary.wiley.com/doi/10.1111/hsc.13094 | 2020 | An evaluation of self-management outcomes among chronic care patients in community home-based care programmes in rural Malawi: a 12-month follow-up study | Repetition |
|  | V. Angwenyi, J. Bunders-Aelen, B. Criel, J. V. Lazarus and C. Aantjes | 2020 | An evaluation of self-management outcomes among chronic care patients in community home-based care programmes in rural Malawi: A 12-month follow-up study | Repetition |
|  | V. Angwenyi, J. Bunders‐Aelen, B. Criel, J. V. Lazarus and C. Aantjes | 2020 | An evaluation of self‐management outcomes among chronic care patients in community home‐based care programmes in rural Malawi: A 12‐month follow‐up study | Repetition |
|  | F. O. Otieno, R. Ndivo, S. Oswago, J. Ondiek, S. Pals et al. | 2014 | Evaluation of syndromic management of sexually transmitted infections within the Kisumu Incidence Cohort Study | Subject |
|  | A. M. B. van der Does and R. Mash | 2013 | Evaluation of the 'Take Five School': An education programme for people with Type 2 Diabetes in the Western Cape, South Africa | Subject |
|  | A. M. van der Does and R. Mash | 2013 | Evaluation of the "Take Five School": an education programme for people with Type 2 Diabetes in the Western Cape, South Africa | Repetition |
|  | S. Rokni, Z. Rezaei, A. D. Noghabi, M. Sajjadi and A. Mohammadpour | 2022 | Evaluation of the effects of diabetes self-management education based on 5A model on the quality of life and blood glucose of women with gestational diabetes mellitus: an experimental study in eastern Iran | Subject |
|  | Z. Shu, L. Wang and X. Sun | 2019 | An evaluation of the effects of general practitioner-supported patient noncommunicable diseases control model in Shanghai, China | Setting |
|  | Bisika, S. Konyani, I. Chamangwana and G. Khanyizira | 2008 | An epidemiologic study of drug abuse and HIV and AIDS in Malawi  T. | Subject |
|  | https://onlinelibrary.wiley.com/toc/10991751/2019/34/3 | 2022 |  | Invalid |
|  | C. Chen, X. Gong, X. Wang, C. Cao, X. Sun et al. | 2019 | Evaluation of the implementation and effect of the healthcare cloud information platform for diabetes self-management: a case study in Shanghai | Setting |
|  | S. Tonen-Wolyec, S. Batina-Agasa, J. Muwonga, F. Fwamba N'kulu, R.-S. Mboumba et al. | 2018 | Evaluation of the practicability and virological performance of finger-stick whole-blood HIV self-testing in French-speaking sub-Saharan Africa | Subject |
|  | J. Tulloch, D. Irwin, E. Pascuet and R. Vaillancourt | 2012 | Evaluation, modification and validation of a set of asthma illustrations in children with chronic asthma in the emergency department | Subject |
|  | E. M. Joseph-Shehu and B. P. Ncama | 2017 | Evidence on health-promoting lifestyle practices and information and communication technologies: scoping review protocol | Protocol |
|  | D. Bajis, A. Al-Haqan, S. Mhlaba, A. Bruno, L. Bader and I. Bates | 2023 | An evidence-led review of the FIP global competency framework for early career pharmacists training and development | Subject |
|  | S. D. Pastakia, R. Karwa, C. B. Kahn and J. S. Nyabundi | 2011 | The evolution of diabetes care in the rural, resource-constrained setting of western Kenya | Subject |
|  | I. Ricci-Cabello, K. Bobrow, S. M. S. Islam, C. K. Chow, R. Maddison et al. | 2019 | Examining Development Processes for Text Messaging Interventions to Prevent Cardiovascular Disease: Systematic Literature Review | Review |
|  | Z. Jannoo, Y. B. Wah, A. M. Lazim and M. A. Hassali | 2017 | Examining diabetes distress, medication adherence, diabetes self-care activities, diabetes-specific quality of life and health-related quality of life among type 2 diabetes mellitus patients | Methods |
|  | J. L. Wolff, Y. Guan, C. M. Boyd, J. Vick, H. Amjad et al. | 2017 | Examining the context and helpfulness of family companion contributions to older adults' primary care visits | Subject |
|  | E. R. Goethals, J. Lemiere, F. J. Snoek, K. Casteels, K. Luyckx and M. de Wit | 2021 | Executive function mediates the link between externalizing behavior and HbA1c in children and adolescents with type 1 diabetes: A cross-national investigation | Population |
|  | I. H. de Boer, M. L. Caramori, J. C. N. Chan, H. J. L. Heerspink, C. Hurst et al. | 2020 | Executive summary of the 2020 KDIGO Diabetes Management in CKD Guideline: evidence-based advances in monitoring and treatment | Protocol |
|  | I. H. de Boer, M. L. Caramori, J. C. N. Chan, H. J. L. Heerspink, C. Hurst et al. | 2022 | Executive summary of the KDIGO 2022 Clinical Practice Guideline for Diabetes Management in Chronic Kidney Disease: an update based on rapidly emerging new evidence | Protocol |
|  | N. Nelson and C. A. Asplund | 2016 | Exercise Testing: Who, When, and Why? | Subject |
|  | A. J. Adler, C. Trujillo, L. Schwartz, L. Drown, J. Pierre et al. | 2021 | Experience of living with type 1 diabetes in a low-income country: a qualitative study from Liberia | Setting |
|  | K. Pleym, M. M. Iversen and A. Broström | 2022 | Experiences and actions related to living with type 1 diabetes during the COVID-19 pandemic in Norway: a qualitative study conducted during July to December 2020 | Setting |
|  | K. F. Mwila, P. A. Bwembya and C. Jacobs | 2019 | Experiences and challenges of adults living with type 2 diabetes mellitus presenting at the University Teaching Hospital in Lusaka, Zambia | Subject |
|  | P. Matwa, M. M. Chabeli, M. Muller and N. S. Levitt | 2003 | Experiences and guidelines for footcare practices of patients with diabetes mellitus | Included |
|  | D. S. Moura da Cruz, K. de Lima Silva, J. T. Batista de Souza, M. M. Lima da Nóbrega, A. P. da Silva Reichert et al. | 2018 | Experiences of adolescents with diabetes mellitus from the perspective of the ethics of alterity | Population |
|  | E. F. Chikumbu, C. Bunn, S. Kasenda, A. Dube, E. Phiri-Makwakwa et al. | 2022 | Experiences of multimorbidity in urban and rural Malawi: an interview study of burdens of treatment and lack of treatment | Included |
|  | https://journals.plos.org/globalpublichealth/article?id=10.1371/journal.pgph.0000139 | 2022 | Experiences of multimorbidity in urban and rural Malawi: an interview study of burdens of treatment and lack of treatment | Repetition |
|  | N. B. D. Magobe, M. Poggenpoel and C. Myburgh | 2017 | Experiences of patients with hypertension at primary health care in facilitating own lifestyle change of regular physical exercise | Included |
|  |  | 2022 | Experiences of people living with multi morbidity in urban and rural Malawi | Repetition |
|  | Akyirem S, Ekpor E. | 2024 | Experience of stigma among persons with type 2 diabetes in Africa: a systematic review. International health | Review |
|  | D. Wilson, A. Mparadzi and S. Lavelle | 1992 | An experimental comparison of two AIDS prevention interventions among young Zimbabweans | Subject |
|  | A. d. G. Aikins, R. B. Awuah, T. A. Pera, M. Mendez and G. Ogedegbe | 2015 | Explanatory models of diabetes in urban poor communities in Accra, Ghana | Included |
|  | https://www.tandfonline.com/loi/ceth20 | 2013 |  | Invalid |
|  | S. Ansari, H. Hosseinzadeh, S. Dennis and N. Zwar | 2012 | Comparative influence of self-efficacy, social support and perceived barriers on low physical activity development in patients with type 2 diabetes, hypertension or stroke | Subject |
|  | M. S. Abdulrehman, W. Woith, S. Jenkins, S. Kossman and G. L. Hunter | 2014 | Exploring Cultural Influences of Self-Management of Diabetes in Coastal Kenya: An Ethnography | Included |
|  | T. M. Kintu, A. M. Ssewanyana, T. Kyagambiddwa, P. M. Nampijja, P. K. Apio et. al. | 2021 | Exploring drivers and barriers to the utilization of community client-led ART delivery model in South-Western Uganda: patients' and health workers' experiences | Subject |
|  | https://link.springer.com/article/10.1186/s12913-021-07105-9 | 2021 | Exploring drivers and barriers to the utilization of community client-led ART delivery model in South-Western Uganda: patients' and health workers' experiences | Repetition |
|  | C. Harichund, Q. A. Karim, P. Kunene, S. Simelane and M. Moshabela | 2019 | Exploring factors that influence the integration of HIVST with HCT using a qualitative comparative cross-over design in KwaZulu-Natal, South Africa | Repetition |
|  | X. Ji and J. Chi | 2024 | Exploring the Relationship Between eHealth Literacy and Diabetes Knowledge, Self-Efficacy, and Self-Care Behaviors in Chinese Diabetic Patients: A Cross-Sectional Study | Setting |
|  | H. M. Lynch, A. S. Green, R. Clarke Nanyonga, D. D. Gadikota-Klumpers, A. Squires et. al. | 2019 | Exploring patient experiences with and attitudes towards hypertension at a private hospital in Uganda: a qualitative study | Population |
|  | S. Pilusa, H. Myezwa and J. Potterton | 2021 | Exploring prevention and management of secondary health conditions in people with spinal cord injury in South Africa | Subject |
|  | H. Areri, A. Marshall and G. Harvey | 2020 | Exploring Self-Management of Adults Living with HIV on Antiretroviral Therapy in North-West Ethiopia: Qualitative Study | Subject |
|  | M. Hushie | 2019 | Exploring the barriers and facilitators of dietary self-care for type 2 diabetes: a qualitative study in Ghana | Population |
|  | D. A. Omodara, L. Gibson and G. Bowpitt | 2022 | Exploring the impact of cultural beliefs in the self-management of type 2 diabetes among Black sub-Saharan Africans in the UK - a qualitative study informed by the PEN-3 cultural model | Setting |
|  | D. A. Omodara, L. Gibson and G. Bowpitt | 2022 | Exploring the impact of cultural beliefs in the self-management of type 2 diabetes among Black sub-Saharan Africans in the UK – a qualitative study informed by the PEN-3 cultural model | Setting |
|  | A. M. A. Salim and B. Elgizoli | 2017 | Exploring the reasons why pharmacists dispense antibiotics without prescriptions in Khartoum state, Sudan | Subject |
|  | C. Walker, A. Weeks, B. McAvoy and E. Demetriou | 2005 | Exploring the role of self-management programmes in caring for people from culturally and linguistically diverse backgrounds in Melbourne, Australia | Setting |
|  | M. R. Tomita, K. Buckner, S. Saharan, K. Persons and S. H. Liao | 2016 | Extended Occupational Therapy Reintegration Strategies for a Woman With Guillain-Barré Syndrome: Case Report | Subject |
|  | E. Collaborative, E. M. Brady, C. Bamuya, D. Beran, J. Correia, A. Crampin et. al. | 2021 | EXTending availability of self-management structured EducatioN programmes for people with type 2 Diabetes in low-to-middle income countries (EXTEND) - a feasibility study in Mozambique and Malawi | Subject |
|  | https://bmjopen.bmj.com/content/11/9/e047425 | 2021 | EXTending availability of self-management structured EducatioN programmes for people with type 2 Diabetes in low-to-middle income countries (EXTEND) - a feasibility study in Mozambique and Malawi | Subject |
|  | E. M. Brady, C. Bamuya, D. Beran, J. Correia, A. Crampin, A. Damasceno et. al. | 2021 | EXTending availability of self-management structured EducatioN programmes for people with type 2 Diabetes in low-to-middle income countries (EXTEND)-a feasibility study in Mozambique and Malawi | Subject |
|  | A. Tekeba, Y. Ayele, B. Negash and T. Gashaw | 2021 | Extent of and Factors Associated with Self-Medication among Clients Visiting Community Pharmacies in the Era of COVID-19: Does It Relieve the Possible Impact of the Pandemic on the Health-Care System? | Subject |
|  | P. M. Murnane, S.-L. Sigamoney, F. Pinillos, S. Shiau, R. Strehlau, F. Patel et. al. | 2017 | Extent of disclosure: what perinatally HIV-infected children have been told about their own HIV status | Subject |
|  | S. Oluchina, S. Karanja and A. Mbugua | 2022 | Facilitators to diabetes self-management in primary care settings, patient perspectives, phenomenological design | Population |
|  | C. Psaros, J. E. Haberer, Y. Boum, A. C. Tsai, J. N. Martin, P. W. Hunt et. al. | 2015 | The factor structure and presentation of depression among HIV-positive adults in Uganda | Subject |
|  | H. M. Burke, M. Chen, M. Buluzi, R. Fuchs, S. Wevill, L. Venkatasubramanian et. al. | 2019 | Factors Affecting Continued Use of Subcutaneous Depot Medroxyprogesterone Acetate (DMPA-SC): A Secondary Analysis of a 1-Year Randomized Trial in Malawi | Subject |
|  | Y. Zhang, Z. Lin, X. Li, T. Xiaoming, Y. Zhou and X. Zhang | 2019 | Factors affecting ICT use in health communication among the older population in Jiangsu, China | Subject |
|  | https://www.degruyter.com/view/j/libr.2019.69.issue-1/libri-2018-0103/libri-2018-0103.xml | 2019 | Factors affecting ICT use in health communication among the older population in Jiangsu, China | Repetition |
|  | C. Chantakeeree, M. Sormunen, M. Estola, P. Jullamate and H. Turunen | 2022 | Factors Affecting Quality of Life among Older Adults with Hypertension in Urban and Rural Areas in Thailand: A Cross-Sectional Study | Subject |
|  | P. Ngassa Piotie, C. Filmalter, M. G. Mohlala, N. Zulu, A. Segale, C. Koenaite, J. W. Muchiri et. al. | 2022 | Factors affecting the implementation of a complex health intervention to improve insulin management in primary care: A SWOT analysis | Siubj |
|  | B. B. Izizag, H. Situakibanza, T. Mbutiwi, R. Ingwe, F. Kiazayawoko, A. Nkodila et. al. | 2018 | Factors associated with acceptability of HIV self-testing (HIVST) among university students in a Peri-Urban area of the Democratic Republic of Congo (DRC) | Subject |
|  | S. Kalibala, W. Tun, P. Cherutich, A. Nganga, E. Oweya and P. Oluoch | 2014 | Factors associated with acceptability of HIV self-testing among health care workers in Kenya | Subject |
|  | J. B. Villiera, H. Katsabola, M. Bvumbwe, J. Mhango, J. Khosa, A. Silverstein and A. L. Nyondo-Mipando | 2022 | Factors associated with antiretroviral therapy adherence among adolescents living with HIV in the era of isoniazid preventive therapy as part of HIV care | Subject |
|  | Y. Zhang, Z. Lin, X. Li, T. Xiaoming, Y. Zhou, X. Zhang | 2022 | Factors Affecting ICT Use in Health Communication among the Older Population in Jiangsu, China | Subject |
|  | B. G. Walle, C. M. Tiruneh, T. Dessie, N. Selomon, A. Kassaw, B. Chekole, M. Wubneh, T. Lankrew and W. A. Bayih | 2022 | Factors associated with children's HIV- positive status disclosure in Wolaita Zone, Southern Ethiopia: a cross-sectional study | Subject |
|  | K. G. Yazew, D. T. Beshah, M. H. Salih and T. A. Zeleke | 2019 | Factors Associated with Depression among Heart Failure Patients at Cardiac Follow-Up Clinics in Northwest Ethiopia, 2017: A Cross-Sectional Study | Subject |
|  | A. Kassahun, B. Yohannes Ayalew and W. Teshome Habte | 2022 | Factors associated with depression among heart failure patients at selected public hospitals in Addis Ababa, Ethiopia: a cross sectional study | Subject |
|  | https://journals.plos.org/globalpublichealth/article?id=10.1371/journal.pgph.0000853 | 2022 | Factors associated with depression among heart failure patients at selected public hospitals in Addis Ababa, Ethiopia: a cross sectional study | Repetition |
|  | B. Hensen, J. J. Lewis, A. Schaap, M. Tembo, W. Mutale, H. A. Weiss, J. Hargreaves and H. Ayles | 2015 | Factors associated with HIV-testing and acceptance of an offer of home-based testing by men in rural Zambia | Sub |
|  | J. Maradzika, T. Mzorodzi and V. Chikwasha | 2017 | Factors associated with hospital admissions among registered diabetes mellitus patients in Guruve and Mazowe district- Mashonaland central province, 2013 | Subject |
|  | https://www.tandfonline.com/loi/rhpe20 | 2014 |  | Invalid |
|  | X. Yang, L. Wang, C. Ge, B. Hu and T. Chi | 2011 | Factors associated with occupational strain among Chinese teachers: a cross-sectional study | Setting |
|  | https://www.sciencedirect.com/science/journal/00333506 | 2013 |  | Invalid |
|  | M. Moshabela, H. Schneider, S. P. Silal and S. M. Cleary | 2012 | Factors associated with patterns of plural healthcare utilization among patients taking antiretroviral therapy in rural and urban South Africa: a cross-sectional study | Subject |
|  | M. Moshabela, H. Schneider, S. P. Silal and S. M. Cleary | 2012 | Factors associated with patterns of plural healthcare utilization among patients taking antiretroviral therapy in rural and urban South Africa: a crosssectional study | Repetition |
|  | S. A. M. Saghir, A. E. A. Alhariri, S. A. Alkubati, A. A. Almiamn, S. H. Aladaileh and N. A. Alyousefi | 2019 | Factors associated with poor glycemic control among type-2 diabetes mellitus patients in Yemen | Setting |
|  | H. Alaofè, W. Amoussa Hounkpatin, F. Djrolo, J. Ehiri and C. Rosales | 2022 | Factors Associated with Quality of Life in Patients with Type 2 Diabetes of South Benin: A Cross-Sectional Study | Subject |
|  | Y. Gurmu, D. Gela and F. Aga | 2018 | Factors associated with self-care practice among adult diabetes patients in West Shoa Zone, Oromia Regional State, Ethiopia | Subject |
|  | T.-H. Dao-Tran, D. Anderson, A. Chang, C. Seib and C. Hurst | 2018 | Factors associated with self-management among Vietnamese adults with type 2 diabetes | Setting |
|  | C. E. Lloyd, N. Sartorius, H. U. Ahmed, A. Alvarez, S. Bahendeka, A. E. Bobrov et. al. | 2020 | Factors associated with the onset of major depressive disorder in adults with type 2 diabetes living in 12 different countries: results from the INTERPRET-DD prospective study | Subject |
|  | Y. Bao, Y. Xu, B. Shen, K. Gu, Y. Yang, H. Wang, L. Han and Z. Ning | 2021 | Factors associated with the self use of oral fluid HIV rapid test among men who have sex with men in Shanghai | Subject |
|  | G. A. Azeze, B. G. Adema, G. A. Adella, B. W. Demissie and M. S. Obsa | 2020 | Factors Associated with Untreated Depression Among Type 2 Diabetic Patients at Halaba Kulito Hospital, South Ethiopia: A Cross-Sectional Study | Subject |
|  | R. Adisa, M. B. Alutundu and T. O. Fakeye | 2009 | Factors contributing to nonadherence to oral hypoglycemic medications among ambulatory type 2 diabetes patients in Southwestern Nigeria | Subject |
|  | G. N. Nyaaba, L. Masana, A. De-Graft Aikins, E. Beune and C. Agyemang | 2020 | Factors hindering hypertension control: perspectives of front-line health professionals in rural Ghana | Population |
|  | https://www.sciencedirect.com/science/article/abs/pii/S0033350619303646 | 2020 | Factors hindering hypertension control: perspectives of front-line health professionals in rural Ghana | Repetition |
|  | S. A. Tiruneh, A. A. Ayele, Y. K. Emiru, H. G. Tegegn, B. A. Ayele, M. T. Engidaw and A. D. Gebremariam | 2019 | Factors influencing diabetes self-care practice among type 2 diabetes patients attending diabetic care follow up at an Ethiopian General Hospital, 2018 | Methods |
|  | A. Alotaibi, L. Gholizadeh, A. H. A. Al-Ganmi and L. Perry | 2018 | Factors influencing nurses' knowledge acquisition of diabetes care and its management: A qualitative study | Subject |
|  | J. N. Suglo and C. Evans | 2020 | Factors influencing self-management in relation to type 2 diabetes in Africa: A qualitative systematic review | Review |
|  | H. Areri, A. Marshall and G. Harvey | 2020 | Factors influencing self-management of adults living with HIV on antiretroviral therapy in Northwest Ethiopia: a cross-sectional study | Methods |
|  | https://link.springer.com/article/10.1186/s12879-020-05618-y | 2020 | Factors influencing self-management of adults living with HIV on antiretroviral therapy in Northwest Ethiopia: a cross-sectional study | Repetition |
|  | X.-L. Liu, K. Willis, P. Fulbrook, C.-J. J. Wu, Y. Shi and M. Johnson | 2019 | Factors influencing self-management priority setting and decision-making among Chinese patients with acute coronary syndrome and type 2 diabetes mellitus | Setting |
|  | D. C. Diriba, D. Y. P. Leung and L. K. P. Suen | 2023 | Factors predicted quality of life of people with type 2 diabetes in western Ethiopia | Subject |
|  | M. Ocan, G. S. Bbosa, P. Waako, J. Ogwal-Okeng and C. Obua | 2014 | Factors predicting home storage of medicines in Northern Uganda | Methods |
|  | J. A. Denison, C. Packer, R. M. Stalter, H. Banda, S. Mercer et al. | 2018 | Factors Related to Incomplete Adherence to Antiretroviral Therapy among Adolescents Attending Three HIV Clinics in the Copperbelt, Zambia | Population |
|  | L. Chenoweth, R. Gallagher, J. N. Sheriff, J. Donoghue and J. Stein-Parbury | 2008 | Factors supporting self-management in Parkinson's disease: implications for nursing practice | Subject |
|  | N. Abrahams, L. Gilson, N. S. Levitt and J. A. Dave | 2019 | Factors that influence patient empowerment in inpatient chronic care: early thoughts on a diabetes care intervention in South Africa | Subject |
|  | S. Bernays, S. Paparini, S. Namukwaya and J. Seeley | 2019 | A Failed Method? Reflections on Using Audio Diaries in Uganda With Young People Growing Up With HIV in the BREATHER Trial | Subject |
|  | H. Siltanen, V. Jylhä, A. Holopainen and E. Paavilainen | 2019 | Family members' experiences and expectations of self-management counseling while caring for a person with chronic obstructive pulmonary disease: a systematic review of qualitative evidence | Review |
|  | S. T. Roberts, P. Edwards, D. Mulenga, N. Chelwa, L. Nyblade et al. | 2021 | Family Support for Adolescent Girls and Young Women Living With HIV in Zambia: Benefits, Challenges, and Recommendations for Intervention Development | Population |
|  | M. Kara Kaşikçi and J. Alberto | 2007 | Family support, perceived self-efficacy and self-care behaviour of Turkish patients with chronic obstructive pulmonary disease | Subject |
|  | L. Mao, J. Lu, Q. Zhang, Y. Zhao, G. Chen et al. | 2019 | Family-based intervention for patients with type 2 diabetes via WeChat in China: protocol for a randomized controlled trial | Protocol |
|  | https://link.springer.com/article/10.1186/s12889-019-6702-8 | 2019 | Family-based intervention for patients with type 2 diabetes via WeChat in China: protocol for a randomized controlled trial | Protocol |
|  | A. T. v. t. Noordende, A. Moges Wubie, M. Nurilign Abebe, T. Tesfaye and A. P. Schippers | 2022 | Family-based intervention for prevention and self-management of disabilities due to leprosy, podoconiosis and lymphatic filariasis versus usual care in Ethiopia: study protocol for a cluster-randomised controlled trial | Sub |
|  | https://bmjopen.bmj.com/content/12/3/e056620 | 2022 | Family-based intervention for prevention and self-management of disabilities due to leprosy, podoconiosis and lymphatic filariasis versus usual care in Ethiopia: study protocol for a cluster-randomised controlled trial | Repetition |
|  | A. T. van 't Noordende, M. W. Aycheh, N. A. Moges, T. Tadesse and A. P. Schippers | 2022 | Family-based intervention for prevention and self-management of disabilities due to leprosy, podoconiosis and lymphatic filariasis versus usual care in Ethiopia: study protocol for a cluster-randomised controlled trial | Repetition |
|  | A. Ispriantari, R. Agustina, K. D. Konlan and H. Lee | 2023 | Family-centered interventions for children and adolescents with type 1 diabetes mellitus: an integrative review | Subject |
|  | R. F. Hess and D. McKinney | 2007 | Fatalism and HIV/AIDS beliefs in rural Mali, West Africa | Subject |
|  | W. T. Chen, C. S. Shiu, J. P. Yang, S. Y. Lee, S. Lee et al. | 2013 | Fatigue and sleep disturbance related to perceived stress in Chinese HIV-positive individuals: a mixed methods study | Subject |
|  | J. K. B. Matovu, L. M. Bogart, J. Nakabugo, J. Kagaayi, D. Serwadda et al. | 2020 | Feasibility and acceptability of a pilot, peer-led HIV self-testing intervention in a hyperendemic fishing community in rural Uganda | Subject |
|  | X. Ntinga, F. Musiello, A. K. Keter, R. Barnabas and A. van Heerden | 2022 | The Feasibility and Acceptability of an mHealth Conversational Agent Designed to Support HIV Self-testing in South Africa: Cross-sectional Study | Subject |
|  | J. T. Kelly, M. M. Warner, M. Conley, D. P. Reidlinger, T. Hoffmann et al. | 2019 | Feasibility and acceptability of telehealth coaching to promote healthy eating in chronic kidney disease: a mixed-methods process evaluation | Subject |
|  | L. Ng'ang'a, G. Ngoga, S. Dusabeyezu, B. L. Hedt-Gauthier, E. Harerimana et al. | 2022 | Feasibility and effectiveness of self-monitoring of blood glucose among insulin-dependent patients with type 2 diabetes: open randomized control trial in three rural districts in Rwanda | Subject |
|  | P. Bray, M. Roupe, S. Young, J. Harrell, D. M. Cummings and L. M. Whetstone | 2005 | Feasibility and effectiveness of system redesign for diabetes care management in rural areas: the eastern North Carolina experience | Subject |
|  | W. Devillé and H. Tempelman | 2009 | Feasibility and robustness of an oral HIV self-test in a rural community in South-Africa: An observational diagnostic study | Subject |
|  | B. Njau, E. Lisasi, D. J. Damian, D. L. Mushi, A. Boulle and C. Mathews | 2020 | Feasibility of an HIV self-testing intervention: a formative qualitative study among individuals, community leaders, and HIV testing experts in northern Tanzania | Subject |
|  | L. Lebina, N. Seatlholo, N. Taruberekera, M. Radebe, A. Kinghorn et al. | 2019 | Feasibility of community-based HIV self-screening in South Africa: a demonstration project | Subject |
|  | L. Lebina, N. Seatlholo, N. Taruberekera, M. Radebe, A. Kinghorn et al. | 2019 | Feasibility of community-based HIV self-screening in South Africa: a demonstration project | Repetition |
|  | F. Z. Amdie, M. Luctkar-Flude, E. Snelgrove-Clarke, M. Sawhney, S. Alemu and K. Woo | 2022 | Feasibility of Virtual Simulation-Based Diabetes Foot Care Education in Patients with Diabetes in Ethiopia: Protocol for a Randomized Controlled Trial | Subject |
|  | M. Kaddumukasa, J. Nakibuuka, L. Mugenyi, O. Namusoke, D. Birungi et al. | 2018 | Feasibility study of a targeted self-management intervention for reducing stroke risk factors in a high-risk population in Uganda | Subject |
|  | M. Kaddumukasa, J. Nakibuuka, L. Mugenyi, O. Namusoke, D. Birungi et al. | 2021 | Feasibility, acceptability, and efficacy of an adopted novel self-management intervention among people with epilepsy in Uganda | Subject |
|  | K. F. Ortblad, D. K. Musoke, T. Ngabirano, A. Nakitende, J. E. Haberer, M. McConnell, J. A. Salomon, T. Bärnighausen and C. E. Oldenburg | 2018 | Female Sex Workers Often Incorrectly Interpret HIV Self-Test Results in Uganda | Subject |
|  | Z. J. Huang, D. Hu, R. Chang, H. Zaccaro, M. Iguchi, H. Zheng and N. He | 2015 | Female streetwalkers' perspectives on migration and HIV/STI risks in a changing economic and social environment: a qualitative study in Shanghai, China | Subject |
|  | https://www.tandfonline.com/doi/full/10.1080/13691058.2014.990518#abstract | 2015 | Female streetwalkers' perspectives on migration and HIV/STI risks in a changing economic and social environment: a qualitative study in Shanghai, China | Repetition |
|  | S. Tonen-Wolyec, A. Sarassoro, J. Muwonga Masidi, E. Twite Banda et al. | 2020 | Field evaluation of capillary blood and oral-fluid HIV self-tests in the Democratic Republic of the Congo | Subject |
|  | S. Russell, F. Martin, F. Zalwango, S. Namukwaya, R. Nalugya et al. | 2016 | Finding meaning: HIV self-management and wellbeing among people taking antiretroviral therapy in Uganda | Subject |
|  | C. F. Otieno, P. M. Nyamu and G. Atieno-Jalang'o | 2005 | Focus on delay as a strategy for care designs and evaluation of diabetic foot ulcers in developing countries: a review | Subject |
|  | C. F. Otieno, P. M. Nyamu and G. Atieno-Jalango | 2005 | Focus on delay as a strategy for care designs and evaluation of diabetic foot ulcers in developing countries: a review | Subject |
|  | F. Aga, S. B. Dunbar, T. Kebede, S. Guteta, M. K. Higgins and R. A. Gary | 2022 | Foot self-care behaviour in type 2 diabetes adults with and without comorbid heart failure | Subject |
|  | H. Alaofè, A. Okechukwu, S. Yeo, P. Magrath, W. Amoussa et al. | 2022 | Formative Qualitative Research: Design Considerations for a Self-Directed Lifestyle Intervention for Type-2 Diabetes Patients Using Human-Centered Design Principles in Benin | Subject |
|  | Z. Tang, C. Shen, W. Tong, X. Xiang, Z. Feng and B. Han | 2022 | Frailty in community-dwelling adults aged 40 years and over with type 2 diabetes: association with self-management behaviors | Subject |
|  | https://www.mdpi.com/1660-4601/19/15/9092 | 2022 | Frailty in community-dwelling adults aged 40 years and over with type 2 diabetes: association with self-management behaviors | Repetition |
|  | D. G. Morrow, H. C. Lane and W. A. Rogers | 2021 | A Framework for Design of Conversational Agents to Support Health Self-Care for Older Adults | Subject |
|  | S. Russell, S. Namukwaya, F. Zalwango and J. Seeley | 2016 | The framing and fashioning of therapeutic citizenship among people living with HIV taking antiretroviral therapy in Uganda | Subject |
|  | https://qhr.sagepub.com/content/by/year | 2021 |  | Invalid |
|  | J. P. Unger, A. Mbaye and M. Diao | 1990 | From Bamako to Kolda: a case study of medicines and the financing of district health services | Subject |
|  | L. Keyser, E. N. B. Myer, J. McKinney, R. Maroyi, D. Mukwege and C. Chen | 2021 | Function and disability status among women with fistula using WHODAS2.0: a descriptive study from Rwanda and Democratic Republic of Congo | Subject |
|  | <https://obgyn.onlinelibrary.wiley.com/doi/10.1002/ijgo.13740> | 2021 | Function and disability status among women with fistula using WHODAS2.0: a descriptive study from Rwanda and Democratic Republic of Congo | Repetition |
|  | L. Keyser, E. N. B. Myer, J. McKinney, R. Maroyi, D. Mukwege and C. C. G. Chen | 2021 | Function and disability status among women with fistula using WHODAS2.0: A descriptive study from Rwanda and Democratic Republic of Congo | Repetition |
|  | C. J. Presley, N. A. Arrato, S. Janse, P. G. Shields, D. P. Carbone et al. | 2022 | Functional Disability Among Older Versus Younger Adults With Advanced Non-Small-Cell Lung Cancer | Subject |
|  | Y.-M. Syu, C.-L. Lee, C.-K. Chuang, H.-C. Chiu, Y.-H. Chang et al. | 2022 | Functional Independence of Taiwanese Children with Osteogenesis Imperfecta | Siubj |
|  | C. J. Presley, N. A. Arrato, P. G. Shields, D. P. Carbone, M. L. Wong et al. | 2022 | Functional Trajectories and Resilience Among Adults With Advanced Lung Cancer | Subject |
|  | X | 2018 | GABRIC Diabetes School: an innovative education centre for people with diabetes | Subject |
|  | A Esteghamati, F Hosseinpanah, S Jahed, H Mohammad, Taghi Cheraghchi Bashi Astaneh, H Kaykhanzadeh, and S Sedaghat | 2018 | GABRIC Diabetes School: an innovative education centre for people with diabetes | Invalid |
|  | K. Closson, J. Dietrich, N. J. Lachowsky, B. Nkala, Z. Cui et al. | 2018 | Gender differences in prevalence and correlates of high sexual self-efficacy among adolescents in Soweto, South Africa: implications for gender-sensitive research and programming | Subject |
|  | G. Chepngeno-Langat, N. Madise, M. Evandrou and J. Falkingham | 2011 | Gender differentials on the health consequences of care-giving to people with AIDS-related illness among older informal carers in two slums in Nairobi, Kenya | Subject |
|  | https://www.tandfonline.com/loi/caic20 | 2011 |  | Invalid |
|  | B. C. Batch, S. E. Spratt, D. V. Blalock, C. Benditz, A. Weiss et al. | 2021 | General Behavioral Engagement and Changes in Clinical and Cognitive Outcomes of Patients with Type 2 Diabetes Using the Time2Focus Mobile App for Diabetes Education: Pilot Evaluation | Subject |
|  | T. Kapwata and S. Manda | 2018 | Geographic assessment of access to health care in patients with cardiovascular disease in South Africa | Subject |
|  | S. Jones | 2008 | Getting involved in research: evaluating the Positive Self-Management Programme | Subject |
|  | R. Fakhry | 2006 | Glargine dose titration by patients and by physicians were equally effective for preventing severe hypoglycaemia | Subject |
|  | L. S. Elinder, S. Hakimi, A. Lager and E. Patterson | 2017 | Global region of birth is an independent risk factor for type 2 diabetes in Stockholm, Sweden | Subject |
|  | E. D. Bateman, S. S. Hurd, P. J. Barnes, J. Bousquet, J. M. Drazen et al. | 2008 | Global strategy for asthma management and prevention: GINA executive summary | Subject |
|  | C. F. Otieno, J. E. Kanu, E. M. Karari, V. Okech-Helu, M. D. Joshi and K. Mutai | 2017 | Glucose-lowering therapies, adequacy of metabolic control, and their relationship with comorbid depression in outpatients with type 2 diabetes in a tertiary hospital in Kenya | Subject |
|  | E. M. Musenge, C. Michelo, B. Mudenda and A. Manankov | 2015 | Glycaemic Control and Associated Self-Management Behaviours in Diabetic Outpatients: A Hospital Based Observation Study in Lusaka, Zambia | Subject |
|  | E. M. Musenge, C. Michelo, B. Mudenda and A. Manankov | 2016 | Glycaemic Control and Associated Self-Management Behaviours in Diabetic Outpatients: A Hospital Based Observation Study in Lusaka, Zambia | Subject |
|  | O. H. Al-Ma'aitah, D. Demant, S. Jakimowicz and L. Perry | 2022 | Glycaemic control and its associated factors in patients with type 2 diabetes in the Middle East and North Africa: An updated systematic review and meta-analysis | Review |
|  | O. H. Al‐ma'aitah, D. Demant, S. Jakimowicz and L. Perry | 2022 | Glycaemic control and its associated factors in patients with type 2 diabetes in the Middle East and North Africa: An updated systematic review and meta‐analysis | Repetition |
|  | M. Noorani, K. Ramaiya and K. Manji | 2016 | Glycaemic control in type 1 diabetes mellitus among children and adolescents in a resource limited setting in Dar es Salaam - Tanzania | Population |
|  | T. Melaku, L. Chelkeba, Z. Mekonnen and K. Kumela | 2020 | Glycemic Control Among People Living with Diabetes and Human Immunodeficiency Virus in Ethiopia: Leveraging Clinical Care for the Looming  Co-Epidemics | Subject |
|  | A. Fasil, B. Biadgo and M. Abebe | 2018 | Glycemic control and diabetes complications among diabetes mellitus patients attending at University of Gondar Hospital, Northwest Ethiopia | Methods |
|  | A.-K. Aro, M. Karjalainen, M. Tiihonen, H. Kautiainen, J. Saltevo et al. | 2017 | Glycemic control and health-related quality of life among older home-dwelling primary care patients with diabetes | Methods |
|  | T. Alemu, T. Tadesse and G. Amogne | 2021 | Glycemic control and its determinants among patients with type 2 diabetes mellitus at Menelik II Referral Hospital, Ethiopia | Methods |
|  | R. BeLue, K. Ndiaye, N. D. F, F. N. Ba and M. Diaw | 2016 | Glycemic Control in a Clinic-Based Sample of Diabetics in M'Bour Senegal | Methods |
|  | R. BeLue, K. Ndiaye, F. Ndao, F. N. N. Ba and M. Diaw | 2018 | Glycemic Control in a Clinic-Based Sample of Diabetics in M'Bour Senegal | Repetition |
|  | I. A. Scott, P. Scuffham, D. Gupta, T. M. Harch, J. Borchi and B. Richards | 2020 | Going digital: a narrative overview of the effects, quality and utility of mobile apps in chronic disease self-management | Subject |
|  | Y. H. Dang, N. M. Patel-Shori, M. Barros and D. Yu | 2020 | Good Literacy to Enhance Response in Diabetes Mellitus (GLITTER-DM) | Subject |
|  | C. A. Teasdale, J. Odondi, C. Kidiga, M. Choy, R. Fayorsey et al. | 2022 | Group antenatal care for improving retention of adolescent and young pregnant women living with HIV in Kenya | Subject |
|  | https://link.springer.com/article/10.1186/s12884-022-04527-z | 2022 | Group antenatal care for improving retention of adolescent and young pregnant women living with HIV in Kenya | Repetition |
|  | S. J. Appel and S. G. Buxbaum | 2016 | Group Conference Call Diabetes Educational Support Sessions: A Pilot Study | Methods |
|  | K. Odgers-Jewell, R. Hughes, E. Isenring, B. Desbrow and M. Leveritt | 2015 | Group facilitators' perceptions of the attributes that contribute to the effectiveness of group-based chronic disease self-management education programs | Subject |
|  | https://onlinelibrary.wiley.com/journal/10.1111/(ISSN)1747-0080 | 2021 |  | Invalid |
|  | C. L. Peiris, N. F. Taylor, S. Hull, A. Anderson, R. Belski, S. Fourlanos and N. Shields | 2018 | A Group Lifestyle Intervention Program Is Associated with Reduced Emergency Department Presentations for People with Metabolic Syndrome: A Retrospective Case-Control Study | Subject |
|  | K. Odgers-Jewell, E. A. Isenring, R. Thomas and D. P. Reidlinger | 2017 | Group participants' experiences of a patient-directed group-based education program for the management of type 2 diabetes mellitus | Subject |
|  | X. Pan, H. Wang, X. Hong, C. Zheng, Y. Wan, N. Buys, Y. Zhang and J. Sun | 2020 | A Group-Based Community Reinforcement Approach of Cognitive Behavioral Therapy Program to Improve Self-Care Behavior of Patients With Type 2 Diabetes | Subject |
|  | K. Winskell, K. S. Miller, K. A. Allen and C. O. Obong'o | 2016 | Guiding and supporting adolescents living with HIV in sub-Saharan Africa: The development of a curriculum for family and community members | Subject |
|  | J. O. Adewuyi, A. M. Coutts, L. Levy and S. E. Lloyd | 1996 | Haemophilia care in Zimbabwe | Subject |
|  | A. d. G. Aikins | 2005 | Healer shopping in Africa: new evidence from rural-urban qualitative study of Ghanaian diabetes experiences | Included |
|  | A. de-Graft Aikins | 2005 | Healer shopping in Africa: new evidence from rural-urban qualitative study of Ghanaian diabetes experiences | Repetition |
|  | C. Moucheraud, K. Phiri and R. M. Hoffman | 2022 | Health behaviours and beliefs among Malawian adults taking antihypertensive medication and antiretroviral therapy: a qualitative study | Included |
|  | https://www.tandfonline.com/doi/full/10.1080/17441692.2021.1874468 | 2022 | Health behaviours and beliefs among Malawian adults taking antihypertensive medication and antiretroviral therapy: a qualitative study | Repetition |
|  | C. Mulugeta Bayisa, J. Miller, S. Ghahari, W. Yemataw, A. Abey and C. Donnelly | 2022 | Health care providers' understanding of self-management support for people with chronic low back pain in Ethiopia: an interpretive description | Population |
|  | https://link.springer.com/article/10.1186/s12913-022-07610-5 | 2022 | Health care providers' understanding of self-management support for people with chronic low back pain in Ethiopia: an interpretive description | Repetition |
|  | S. D. Gumede and M. N. Sibiya | 2018 | Health care users' knowledge, attitudes and perceptions of HIV self-testing at selected gateway clinics at eThekwini district, KwaZulu-Natal province, South Africa | Subject |
|  | C. Liddy, S. Johnston, K. Nash, N. Ward and H. Irving | 2014 | Health coaching in primary care: a feasibility model for diabetes care | Subject |
|  | Y. Wei, Y. Chen, Y. Zhao, R. Rothman, J. Ming et al. | 2020 | Health literacy and exercise interventions on clinical outcomes in Chinese patients with diabetes: a propensity score-matched comparison | Subject |
|  | https://drc.bmj.com/content/8/1/e001179 | 2020 | Health literacy and exercise interventions on clinical outcomes in Chinese patients with diabetes: a propensity score-matched comparison | Repetition |
|  | J. Thompson, Y. Havenga and S. Naude | 2015 | The health literacy needs of women living with HIV/AIDS | Subject |
|  | https://www.sciencedirect.com/science/article/pii/S1025984815000046 | 2015 | The health literacy needs of women living with HIV/AIDS | Repetition |
|  | P. Harbour and L. Grealish | 2018 | Health literacy of the baby boomer generation and the implications for nursing | Subject |
|  | R. Xu, X. Zhou, S. Cao, B. Huang, C. Wu et al. | 2019 | Health status of the elderly and its influence on their activities of daily living in Shangrao, Jiangxi Province | Subject |
|  | E. Rutebemberwa, K. Nyamurungi, S. Joshi, Y. Olando, H. M. Mamudu and R. P. Pack | 2021 | Health workers' perceptions on where and how to integrate tobacco use cessation services into tuberculosis treatment; a qualitative exploratory study in Uganda | Subject |
|  | D. M. Kindarara, M. M. McEwen, J. D. Crist and L. J. Loescher | 2017 | Health-Illness Transition Experiences With Type 2 Diabetes Self-management of Sub-Saharan African Immigrants in the United States | Population |
|  | R. S. Muliira and J. K. Muliira | 2011 | Health-promoting practices and the factors associated with self-reported poor health in caregivers of children orphaned by AIDS in southwest Uganda | Subject |
|  | https://www.tandfonline.com/loi/raar20 | 2019 |  | Invalid |
|  | E. D. Chem, A. Ferry, J. Seeley, H. A. Weiss and V. Simms | 2022 | Health-related needs reported by adolescents living with HIV and receiving antiretroviral therapy in sub-Saharan Africa: a systematic literature review | Review |
|  | https://onlinelibrary.wiley.com/doi/10.1002/jia2.25921 | 2022 | Health-related needs reported by adolescents living with HIV and receiving antiretroviral therapy in sub-Saharan Africa: a systematic literature review | Review |
|  | O. S. Umukoro | 2019 | Health-Related Optimism and Quality of Life among Diabetes Patients: The Moderating Role of Clinical Factors in a Nigerian Sample | Subject |
|  | A. I. G. Gonzalez, C. Schmucker, J. Nothacker, E. Motschall, T. S. Nguyen et al. | 2019 | Health-related preferences of older patients with multimorbidity: an evidence map | Subject |
|  | S. J. Lubinga, G. A. Levine, A. M. Jenny, J. Ngonzi, P. Mukasa-Kivunike et al. | 2013 | Health-related quality of life and social support among women treated for abortion complications in western Uganda | Subject |
|  | A. Tomita, N. Garrett, L. Werner, J. K. Burns, L. Mpanza et al. | 2014 | Health-related quality of life dynamics of HIV-positive South African women up to ART initiation: evidence from the CAPRISA 002 acute infection cohort study | Subject |
|  | Q. Liu, H. Zeng, R. Xia, G. Chen, S. Liu et al. | 2018 | Health-related quality of life of esophageal cancer patients in daily life after treatment: A multicenter cross-sectional study in China | Subject |
|  | T. Shimels, R. A. Kassu, G. Bogale, M. B. Muleta, G. T. Akalu et al. | 2022 | Health-Related Quality of Life of Patients with Type 2 Diabetes Mellitus and Hypertension in Addis Ababa, Ethiopia | Subject |
|  | S. Tariku, A. Mebratu, G. Melsew, M. B. Muleta, K. Rodas Asrat et al. | 2022 | Health-related quality of life of patients with type 2 diabetes mellitus and hypertension in Addis Ababa, Ethiopia | Subject |
|  | https://www.ajol.info/index.php/ejhs/article/view/223346 | 2022 | Health-related quality of life of patients with type 2 diabetes mellitus and hypertension in Addis Ababa, Ethiopia | Subject |
|  | J. Hughes, J. Jelsma, E. Maclean, M. Darder and X. Tinise | 2004 | The health-related quality of life of people living with HIV/AIDS | Subject |
|  | A. Mogueo, B. K. Defo and J. C. Mbanya | 2022 | Healthcare providers' and policymakers' experiences and perspectives on barriers and facilitators to chronic disease self-management for people living with hypertension and diabetes in Cameroon | Subject |
|  | E. Richard, S. Jongstra, H. Soininen, C. Brayne, E. P. Moll van Charante et al. | 2016 | Healthy Ageing Through Internet Counselling in the Elderly: the HATICE randomised controlled trial for the prevention of cardiovascular disease and cognitive impairment | Subject |
|  | V. Hsieh, G. Paull and B. Hawkshaw | 2020 | Heart Failure Integrated Care Project: overcoming barriers encountered by primary health care providers in heart failure management | Subject |
|  | B. S. Tusa, B. M. Geremew and M. A. Tefera | 2013 | Heath related quality of life and associated factors among adults with and without diabetes in Adama city East Shewa, Ethiopia 2019; using generalized structural equation modeling | Subject |
|  | C. W.-L. Ho, K. Caals and H. Zhang | 2020 | Heralding the Digitalization of Life in Post-Pandemic East Asian Societies | Subject |
|  | K. H. Pitkälä, M. H. Suominen, J. S. Bell and T. E. Strandberg | 2016 | Herbal medications and other dietary supplements. A clinical review for physicians caring for older people | Subject |
|  | R. Prince | 2012 | HIV and the Moral Economy of Survival in an East African City | Subject |
|  | J. E. Korte, R. Kisa, C. J. Vrana-Diaz, A. M. Malek, E. Buregyeya et al. | 2020 | HIV Oral Self-Testing for Male Partners of Women Attending Antenatal Care in Central Uganda: Uptake of Testing and Linkage to Care in a Randomized Trial | Subject |
|  | S. A. Shallo and M. Tassew | 2020 | HIV Positive Status Disclosure and Its Associated Factors Among Children on Antiretroviral Therapy in West Shoa Zone, Western Ethiopia, 2019: A Mixed Method Cross-Sectional Study | Subject |
|  | L. G. Dodzo, H. T. Mahaka, D. Mukona, M. Zvinavashe and C. Haruzivishe | 2017 | HIV self-care practices during pregnancy and maternal health outcomes among HIV-positive postnatal mothers aged 18-35 years at Mbuya Nehanda maternity hospital | Subject |
|  | https://www.tandfonline.com/loi/caic20 | 2017 |  | Invalid |
|  | L. G. Dodzo, H. T. Mahaka, D. Mukona, M. Zvinavashe and C. Haruzivishe | 2017 | HIV self-care practices during pregnancy and maternal health outcomes among HIV-positive postnatal mothers aged 18–35 years at Mbuya Nehanda maternity hospital | Subject |
|  | A. R. Aluisio, S. J. Bergam, J. Sugut, J. Kinuthia, R. Bosire et al. | 2023 | HIV self-testing acceptability among injured persons seeking emergency care in Nairobi, Kenya | Subject |
|  | A. T. Choko, E. L. Corbett, N. Stallard, H. Maheswaran, A. Lepine, C. C. Johnson et al. | 2019 | HIV self-testing alone or with additional interventions, including financial incentives, and linkage to care or prevention among male partners of antenatal care clinic attendees in Malawi: An adaptive multi-arm, multi-stage cluster randomised trial | Subject |
|  | C. Harichund, Q. A. Karim, P. Kunene, S. Simelane and M. Moshabela | 2019 | HIV self-testing as part of a differentiated HIV testing approach: exploring urban and rural adult experiences from KwaZulu-Natal, South Africa using a cross-over study design | Subject |
|  | T. Makusha, L. Knight, M. Taegtmeyer, O. Tulloch, A. Davids et al. | 2025 | HIV self-testing could "revolutionize testing in South Africa, but it has got to be done properly": perceptions of key stakeholders | Subject |
|  | M. B. Moreau, F. D. Kintin, S. Atchekpe, G. Batona, L. Béhanzin et al. | 2022 | HIV self-testing implementation, distribution and use among female sex workers in Cotonou, Benin: a qualitative evaluation of acceptability and feasibility | Subject |
|  | https://link.springer.com/article/10.1186/s12889-022-12917-3 | 2022 | HIV self-testing implementation, distribution and use among female sex workers in Cotonou, Benin: a qualitative evaluation of acceptability and feasibility | Subject |
|  | S. Tonen-Wolyec, D. Koyalta, R. S. Mboumba Bouassa, M. Filali, S. Batina-Agasa and L. Bélec | 2918 | HIV self-testing in adolescents living in sub-Saharan Africa | Subject |
|  | https://www.sciencedirect.com/science/article/abs/pii/S0399077X20306752 | 2018 | HIV self-testing in adolescents living in sub-Saharan Africa | Subject |
|  | S. Tonen-Wolyec, M. Filali, S. Mboup and L. Bélec | 2018 | HIV self-testing in Africa: stakes and challenges | Subject |
|  | A. Zanolini, J. Chipungu, M. J. Vinikoor, S. Bosomprah, M. Mafwenko et al. | 2018 | HIV Self-Testing in Lusaka Province, Zambia: Acceptability, Comprehension of Testing Instructions, and Individual Preferences for Self-Test Kit Distribution in a Population-Based Sample of Adolescents and Adults | Subject |
|  | P. O'Byrne, A. Musten, A. Vandyk, N. Ho, L. Orser et al. | 2021 | HIV self-testing in Ottawa, Canada used by persons at risk for HIV: the GetaKit study | Subject |
|  | https://www.canada.ca/en/public-health/services/reports-publications/canada-communicable-disease-report-ccdr/monthly-issue/2021-47/issue-10-october-2021/hiv-self-testing-ottawa-getakit-study.html | 2020 |  | Invalid |
|  | B. Kebede, T. Abate and D. Mekonnen | 2013 | HIV self-testing practices among Health Care Workers: feasibility and options for accelerating HIV testing services in Ethiopia | Subject |
|  | S. Napierala, N. A. Desmond, M. K. Kumwenda, M. Tumushime, E. L. Sibanda et al. | 2019 | HIV self-testing services for female sex workers, Malawi and Zimbabwe | Subject |
|  | T. D. Ritchwood, A. Selin, A. Pettifor, S. A. Lippman, H. Gilmore et al. | 2019 | HIV self-testing: South African young adults' recommendations for ease of use, test kit contents, accessibility, and supportive resources | Subject |
|  | D. K. Ekouevi, A. M. Bitty-Anderson, F. A. Gbeasor-Komlanvi, A. P. Coffie and S. P. Eholie | 2020 | HIV self-testing: The key to unlock the first 90 in West and Central Africa | Subject |
|  | D. L. Joseph Davey, K. M. Wall, N. Naidoo, D. Naidoo, G. Xaba et al. | 2022 | HIV testing and linkage to ART following secondary distribution of HIV self-test kits to male partners of women living with HIV: a pilot randomized control trial in Mpumalanga, South Africa | Subject |
|  | V. G. Billioux, M. K. Grabowski, J. Ssekasanvu, S. J. Reynolds, A. Berman et al. | 2018 | HIV viral suppression and geospatial patterns of HIV antiretroviral therapy treatment facility use in Rakai, Uganda | Subject |
|  | A. von Braun, C. Sekaggya-Wiltshire, N. Bachmann, D. Ssemwanga, A. U. Scherrer et al. | 2018 | HIV-1 Drug Resistance Among Ugandan Adults Attending an Urban Out-Patient Clinic | Subject |
|  | K. F. Ortblad, J. E. Kearney, K. Mugwanya, E. M. Irungu, J. E. Haberer et al. | 2019 | HIV-1 self-testing to improve the efficiency of pre-exposure prophylaxis delivery: a randomized trial in Kenya | Subject |
|  | N. A. Sukati, S. C. Mndebele, E. T. Makoa, T. S. Ramukumba, L. N. Makoae et al. | 2005 | HIV/AIDS symptom management in Southern Africa | Subject |
|  | https://www.sciencedirect.com/science?_ob=ArticleURL&_udi=B6T8R-4FJGKYK-F&_user=10&_handle=V-WA-A-W-AZ-MsSAYWW-UUW-U-AAACVVCDVW-AAABUWZCVW-BWYDZEAD-AZ-U&_fmt=summary&_coverDate=02%2F01%2F2005&_rdoc=13&_orig=browse&_srch=%23toc%235093%232005%23999709997%23574520!&_cdi=5093&view=c&_acct=C000050221&_version=1&_urlVersion=0&_userid=10&md5=212d8284b68feec7e1e457c24487192c | 2019 |  | Invalid |
|  | N. A. Sukati, S. C. Mndebele, E. T. Makoa, T. S. Rumukumba, L. N. Makoae et al. | 2005 | HIV/AIDS symptom management in Southern Africa | Subject |
|  | N. A. Sukati, S. C. Mndebele, E. T. s. Makoa, T. S. Ramukumba, L. N. Makoae, N. M. Seboni, S. Human and W. L. Holzemer | 2005 | HIV/AIDS symptom management in Southern Africa | Subject |
|  | S. N. Ofori and C. N. Unachukwu | 2014 | Holistic approach to prevention and management of type 2 diabetes mellitus in a family setting | Methods |
|  | Y. Berhane and D. Zakus | 1995 | Home care for persons with AIDS: community attitude in a neighbourhood of Addis Ababa, Ethiopia | Subject |
|  | M. Richter, W. D. F. Venter and A. Gray | 2010 | Home self-testing for HIV: AIDS exceptionalism gone wrong | Subject |
|  | H. D. Mazigo, H. M. Bushahu, B. R. Kidenya, E. E. Ambrose, M. Zinga and J. Heukelbach | 2011 | Home treatments with antipyretics and antimalarials given to underfives with fever in Mwanza, north-western Tanzania | Subject |
|  | [P H Joubert](https://pubmed.ncbi.nlm.nih.gov/?term=Joubert+PH&cauthor_id=6719305), [P D Sebata](https://pubmed.ncbi.nlm.nih.gov/?term=Sebata+PD&cauthor_id=6719305), [W J Bam](https://pubmed.ncbi.nlm.nih.gov/?term=Bam+WJ&cauthor_id=6719305), [D J Skene](https://pubmed.ncbi.nlm.nih.gov/?term=Skene+DJ&cauthor_id=6719305) | 1984 | Home urinary glucose testing. Its impact on a Third World diabetic population | Subject |
|  | F. C. Tanser, H.-Y. Kim, T. Mathenjwa, M. Shahmanesh, J. Seeley et al. | 2021 | Home-Based Intervention to Test and Start (HITS): a community-randomized controlled trial to increase HIV testing uptake among men in rural South Africa | Subject |
|  | A. Amstutz, T. I. Lejone, L. Khesa, J. Muhairwe, B. L. Nsakala et al. | 2019 | The HOSENG trial - Effect of the provision of oral self-testing for absent and refusing individuals during a door-to-door HIV-testing campaign on testing coverage: protocol of a cluster-randomized clinical trial in rural Lesotho | Subject |
|  | N. E. Andrew, M. F. Kilkenny, V. Sundararajan, J. Kim, S. G. Faux et al. | 2020 | Hospital Presentations in Long-Term Survivors of Stroke: Causes and Associated Factors in a Linked Data Study | Subject |
|  | S. James, K. Annetts, T. Frakking, M. Broadbent, J. Waugh et al. | 2022 | Hospital presentations with diabetic ketoacidosis: A retrospective review | Subject |
|  | A. C. Lau, L. Y. Yam and E. Poon | 2001 | Hospital re-admission in patients with acute exacerbation of chronic obstructive pulmonary disease | Subject |
|  | S. Roberts, A. Marshall and W. Chaboyer | 2017 | Hospital staffs' perceptions of an electronic program to engage patients in nutrition care at the bedside: a qualitative study | Subject |
|  | M. Moshabela, N. Msomi, G. C. M. Kalla, G. Maimela, J.-C. Yombi and F.-X. Mbopi-Keou | 2022 | Hosting ICASA 2021 in South Africa amidst the global Omicron scare | Subject |
|  | M. Ocan, E. A. Obuku, F. Bwanga, D. Akena, S. Richard et al. | 2015 | Household antimicrobial self-medication: a systematic review and meta-analysis of the burden, risk factors and outcomes in developing countries | Subject |
|  | I. Lisko, J. Kulmala, M. Annetorp, T. Ngandu, F. Mangialasche and M. Kivipelto | 2021 | How can dementia and disability be prevented in older adults: where are we today and where are we going? | Subject |
|  | C. A. Laurenzi, G. J. Melendez-Torres, D. T. Page, L. S. Vogel, T. Kara et al. | 2022 | How do psychosocial interventions for adolescents and young people living with HIV improve adherence and viral load? A realist review | Subject |
|  | https://www.sciencedirect.com/science/article/pii/S1054139X22004086 | 2022 | How do psychosocial interventions for adolescents and young people living with HIV improve adherence and viral load? A realist review | Subject |
|  | N. Brew-Sam and A. Chib | 2019 | How do Smart Device Apps for Diabetes Self-Management correspond with Theoretical Indicators of Empowerment? An Analysis of App Features-CORRIGENDUM | Subject |
|  | E. J. A. Beune, J. A. Haafkens, C. Agyemang, J. S. Schuster and D. L. Willems | 2008 | How Ghanaian, African-Surinamese and Dutch patients perceive and manage antihypertensive drug treatment: a qualitative study | Subject |
|  | I. P. Obiebi, N. S. Moeteke, G. U. Eze and I. J. Umuago | 2020 | How mindful of their own health are healthcare professionals? Perception and practice of personnel in a tertiary hospital in Nigeria | Subject |
|  | Irikefe P. Obiebi , Nnamdi S. Moeteke , Godson U. Eze and Ibiyemi J. Umuago | 2008 | How mindful of their own health are healthcare professionals? Perception and practice of personnel in a tertiary hospital in Nigeria | Subject |
|  | K. Batt-Rawden and G. Tellnes | 2011 | How music may promote healthy behaviour | Subject |
|  | N. C. Ware, M. A. Wyatt, E. E. Pisarski, B. Kamusiime, V. Kasiita et al. | 2023 | How pregnant women living with HIV and their male partners manage men's HIV self-testing: qualitative analysis of an HIVST secondary distribution process in Kampala, Uganda | Subject |
|  | H. Guo, X. Wang, T. Mao, X. Li, M. Wu and J. Chen | 2018 | How psychosocial outcomes impact on the self-reported health status in type 2 diabetes patients: Findings from the Diabetes Attitudes, Wishes and Needs (DAWN) study in eastern China | Subject |
|  | V. H. Davis, S. A. Nixon, K. Murphy, C. Cameron, V. A. Bond et al. | 2022 | How the Term 'Self-Management' is Used in HIV Research in Low- and Middle-Income Countries: A Scoping Review | Review |
|  | R. E. Kohler, T. Elliott, B. Monare, N. Moshashane, K. Ramontshonyana et al. | 2019 | HPV self-sampling acceptability and preferences among women living with HIV in Botswana | Subject |
|  | R. E. Kohler, T. Elliott, B. Monare, N. Moshashane, K. Ramontshonyana et al. | 2019 | HPV self-sampling acceptability and preferences among women living with HIV in Botswana | Repetition |
|  | L. Janneck, N. Cooper, S. Frehywot, H. Mowafi, K. Hein et al. | 2009 | Human Resources in Humanitarian Health Working Group Report | Subject |
|  | O. G. Egbi, A. N. Ofili and E. Oviasu | 2015 | Hypertension and Diabetes Self-care Activities: A Hospital Based Pilot Survey in Benin City, Nigeria | Methods |
|  | R. Vedanthan, D. J. Lee, J. H. Kamano, O. I. Herasme, P. Kiptoo et al. | 2019 | Hypertension management in rural western Kenya: a needs-based health workforce estimation model | Subject |
|  | D. T. Tebelu, T. A. Tadesse, M. S. Getahun, Y. M. Negussie and A. M. Gurara | 2023 | Hypertension self-care practice and its associated factors in Bale Zone, Southeast Ethiopia: A multi-center cross-sectional study | Methods |
|  | E. A. Muche and B. T. Mekonen | 2020 | Hypoglycemia prevention practice and its associated factors among diabetes patients at university teaching hospital in Ethiopia: Cross-sectional study | Methods |
|  | C. A. Stone, H. Siril, E. Nampanda, M. E. Garcia, J. Tito et al. | 2011 | I didn't know that... patient perceptions of print information, education, and communication related to HIV/AIDS treatment | Subject |
|  | S. Pilusa, H. Myezwa and J. Potterton | 2021 | 'I forget to do pressure relief': Personal factors influencing the prevention of secondary health conditions in people with spinal cord injury, South Africa | Subject |
|  | E. Namukwaya, S. A. Murray, J. Downing, M. Leng and L. Grant | 2017 | 'I think my body has become addicted to those tablets'. Chronic heart failure patients' understanding of and beliefs about their illness and its treatment: A qualitative longitudinal study from Uganda | Subject |
|  | W. S. Lora, N. Desmond, A. Obasi, M. Kumwenda, M. Taegtmeyer et al. | 2020 | I wanted evidence that my status had changed, so that is why I tested: experiences with HIV self-testing among female sex workers in Malawi | Subject |
|  | S. A. Berkowitz, N. N. Shahid, J. Terranova, B. Steiner, M. P. Ruazol et al. | 2020 | I was able to eat what I am supposed to eat-- patient reflections on a medically-tailored meal intervention: a qualitative analysis | Setting |
|  | O. Dirisu, A. Sekoni, L. Vu, S. Adebajo, J. Njab et al. | 2020 | 'I will welcome this one 101%, I will so embrace it': a qualitative exploration of the feasibility and acceptability of HIV self-testing among men who have sex with men (MSM) in Lagos, Nigeria | Subject |
|  | M. Ismayilova and S. Yaya | 2022 | 'I'm usually being my own doctor': women's experiences of managing polycystic ovary syndrome in Canada | Setting |
|  | M. Rijken, J. M. Valderas, M. Heins, F. Schellevis and J. Korevaar | 2020 | Identifying high-need patients with multimorbidity from their illness perceptions and personal resources to manage their health and care: a longitudinal study | Subject |
|  | M. Rijken and I. van der Heide | 2019 | Identifying subgroups of persons with multimorbidity based on their needs for care and support | Subject |
|  | I. Vassilev, A. Rogers, A. Kennedy, C. Oatley and E. James | 2019 | Identifying the processes of change and engagement from using a social network intervention for people with long‐term conditions. A qualitative study | Subject |
|  | P. C. Birchfield | 203 | Identifying women at risk for coronary artery disease | Subject |
|  | D. Diriba | 2022 | IDF21-0155 Effectiveness of a nurse-led community-based self-management program among adults with diabetes-family dyads in Ethiopia | Subject |
|  | H. Kloos, A. Etea, A. Degefa, H. Aga, B. Solomon et al. | 1987 | Illness and health behaviour in Addis Ababa and rural central Ethiopia | Methods |
|  | N. Kugbey, K. O. Asante and K. Adulai | 2017 | Illness perception, diabetes knowledge and self-care practices among type-2 diabetes patients: a cross-sectional study | Methods |
|  | https://bmcresnotes.biomedcentral.com/track/pdf/10.1186/s13104-017-2707-5 | 2017 | Illness perception, diabetes knowledge and self-care practices among type-2 diabetes patients: a cross-sectional study | Methods |
|  | N. Kugbey, K. Oppong Asante and K. Adulai | 2017 | Illness perception, diabetes knowledge and self-care practices among type-2 diabetes patients: a cross-sectional study | Methods |
|  | E. M. Nagourney, N. M. Robertson, N. Rykiel, T. Siddharthan, P. Alupo et al. | 2020 | Illness representations of chronic obstructive pulmonary disease (COPD) to inform health education strategies and research design-learning from rural Uganda | Subject |
|  | J. R. Zuyderduin, V. J. Ehlers and D. M. van der Wal | 2008 | The impact of a buddy system on the self-care behaviours of women living with HIV/AIDS in Botswana | Subject |
|  | J. R. Zuyderduin, V. J. Ehlers and D. M. v. d. Wal | 2008 | The impact of a buddy system on the self-care behaviours of women living with HIV/AIDS in Botswana | Subject |
|  | C. Liddy, S. Johnston, S. Guilcher, H. Irving, M. Hogel and S. Jaglal | 2015 | Impact of a chronic disease self-management program on healthcare utilization in eastern Ontario, Canada | Setting |
|  | L. W. Chang, I. Mbabali, X. Kong, H. Hutton, K. R. Amico et al. | 2017 | Impact of a community health worker HIV treatment and prevention intervention in an HIV hotspot fishing community in Rakai, Uganda (mLAKE): study protocol for a randomized controlled trial | Protocol |
|  | L. W. Chang, I. Mbabali, K. Xiangrong, H. Hutton, K. R. Amico et al. | 2017 | Impact of a community health worker HIV treatment and prevention intervention in an HIV hotspot fishing community in Rakai, Uganda (mLAKE): study protocol for a randomized controlled trial | Protocol |
|  | M. Y. Dehayem, R. Takogue, S.-P. Choukem, O. T. S. Donfack, J.-C. Katte et al. | 2016 | Impact of a pioneer diabetes camp experience on glycemic control among children and adolescents living with type 1 diabetes in sub-Saharan Africa | Population |
|  | M. Y. Dehayem, R. Takogue, S. P. Choukem, O. T. Donfack, J. C. Katte et al. | 2016 | Impact of a pioneer diabetes camp experience on glycemic control among children and adolescents living with type 1 diabetes in sub-Saharan Africa | Population |
|  | B. Lormeau, S. Pichat, L. Dufaitre, A. Chamouine, M. Gataa et al. | 2019 | Impact of a sports project centered on scuba diving for adolescents with type 1 diabetes mellitus: New guidelines for adolescent recreational diving, a modification of the French regulations | Subject |
|  | A. A. Adedimeji, O. O. Alawode and O. Odutolu | 2010 | Impact of care and social support on wellbeing among people living with HIV/AIDS in Nigeria | Subject |
|  | J. J. Gagliardino, J.-M. Chantelot, C. Domenger, A. Ramachandran, G. Kaddaha et al. | 2019 | Impact of diabetes education and self-management on the quality of care for people with type 1 diabetes mellitus in the Middle East (the International Diabetes Mellitus Practices Study, IDMPS) | Subject |
|  | D. Enricho Nkhoma, C. Jenya Soko, K. Joseph Banda, D. Greenfield, Y.-C. J. Li and U. Iqbal | 2021 | Impact of DSMES app interventions on medication adherence in type 2 diabetes mellitus: systematic review and meta-analysis | Review |
|  | S. Nkomani, S. Ruskaniko and R. Blaauw | 2021 | The impact of existing diabetes self-management education interventions on knowledge, attitudes and practices in public health care institutions in Harare, Zimbabwe | Subject |
|  | S. Nkomani, S. Ruskaniko and R. Blaauw | 2018 | The impact of existing diabetes self-management education interventions on knowledge, attitudes and practices in public health care institutions in Harare, Zimbabwe | Subject |
|  | L. Lewis, B. Maughan-Brown, A. Grobler, C. Cawood, D. Khanyile et al. | 2019 | Impact of Home-Based HIV Testing Services on Progress Toward the UNAIDS 90-90-90 Targets in a Hyperendemic Area of South Africa | Subject |
|  | X. Liu, C. Wu, K. Willis, Y. Shi and M. Johnson | 2018 | The impact of inpatient education on self-management for patients with acute coronary syndrome and type 2 diabetes mellitus: a cross-sectional study in China | Subject |
|  | https://academic.oup.com/her/ | 2018 |  | Invalid |
|  | B. A. Bene, S. O'Connor, N. Mastellos, A. Majeed, K. P. Fadahunsi and J. O'Donoghue | 2019 | Impact of mobile health applications on self-management in patients with type 2 diabetes mellitus: protocol of a systematic review | Subject |
|  | E. A. David, R. O. Soremekun, I. O. Abah and R. I. Aderemi-Williams | 2021 | Impact of pharmacist-led care on glycaemic control of patients with uncontrolled type 2 diabetes: a randomised controlled trial in Nigeria | Subject |
|  | D. J. Navarro, P. T. Alpert and C. Cross | 2019 | The Impact of Shift Work on Diabetes Self-Management Activities | Subject |
|  | K. Khunti, V. R. Aroda, P. Aschner, J. C. N. Chan, S. Del Prato et al. | 2022 | The impact of the COVID-19 pandemic on diabetes services: planning for a global recovery | Subject |
|  | E. Ojukwu, C. Chan, B. M. Ibitoye, A. Tawfik, Y. Nguyen et al. | 2023 | Impact of the COVID-19 pandemic on the HIV care continuum: a mixed methods systematic review protocol | Protocol |
|  | A. Razak and A. A. Isaacs | 2017 | Implementation and evaluation of a weight-reduction programme for diabetic patients at a primary health care facility in the Western Cape: a pilot study | Subject |
|  | A.Razak and A.A. Issacs | 2017 | Implementation and evaluation of a weight-reduction programme for diabetic patients at a primary health care facility in the Western Cape: a pilot study | Invalid |
|  | C. Liddy, S. Johnston, K. Nash, H. Irving and R. Davidson | 2016 | Implementation and evolution of a regional chronic disease self-management program | Subject |
|  | https://journal.cpha.ca/index.php/cjph/article/view/5126 |  |  | Invalid |
|  | D. Fu, H. Fu, P. McGowan, E. Shen Yi, L. Zhu et al. | 2003 | Implementation and quantitative evaluation of chronic disease self-management programme in Shanghai, China: randomized controlled trial | Subject |
|  | L. Ng'ang'a, G. Ngoga, S. Dusabeyezu, B. L. Hedt-Gauthier, P. Ngamije, M. Habiyaremye et al. | 2020 | Implementation of blood glucose self-monitoring among insulin-dependent patients with type 2 diabetes in three rural districts in Rwanda: 6 months open randomised controlled trial | Subject |
|  | L. Ng'ang'a, G. Ngoga, S. Dusabeyezu, B. L. Hedt-Gauthier, P. Ngamije, M. Habiyaremye et al. | 2020 | Implementation of blood glucose self-monitoring among insulin-dependent patients with type 2 diabetes in three rural districts in Rwanda: 6 months open randomised controlled trial | Subject |
|  | L. Pasipamire, R. C. Nesbitt, L. Dube, E. Mabena, M. Nzima et al. | 2020 | Implementation of community and facility-based HIV self-testing under routine conditions in southern Eswatini | Subject |
|  | T. Siddharthan, T. Rabin, M. E. Canavan, F. Nassali, P. Kirchhoff et al. | 2016 | Implementation of patient-centered education for chronic-disease management in Uganda: an effectiveness study | Subject |
|  | R. Siddharthan, T. Rabin,M.E. Canavan, F. Nassali, P. Kirchhoff | 2020 | Implementation of Patient-Centered Education for Chronic-Disease Management in Uganda: An Effectiveness Study | Invalid |
|  | C. U. Nwankwo, C. E. Ezenwaka, P. C. Onuoha and N. R. Agbakoba | 2015 | Implementing diabetes self-management education (DSME) in a Nigerian population: perceptions of practice nurses and dieticians | Subject |
|  | M. W. Vander Weg, J. E. Holman, H. Rahman, M. V. Sarrazin, S. L. Hillis et al. | 2017 | Implementing smoking cessation guidelines for hospitalized Veterans: Cessation results from the VA-BEST trial | Siubj |
|  | X. Wang, J. Cheng, C. Guo and X. Xu | 2020 | The implications of childcare on grandparents' health self-management in a Chinese elderly population | Subject |
|  | Wang X, J Cheng, J, Guo C-y, Xu X-r | 2019 | The implications of childcare on grandparents' health self-management in a Chinese elderly population | Repetition |
|  | G. Twigg, T. David and J. Taylor | 2019 | An Improved Comprehensive Medication Review Process to Assess Healthcare Outcomes in a Rural Independent Community Pharmacy | Subject |
|  | C. Liddy, W. Hogg, G. Russell, G. Wells, C. D. Armstrong et al. | 2022 | Improved delivery of cardiovascular care (IDOCC) through outreach facilitation: study protocol and implementation details of a cluster randomized controlled trial in primary care | Subject |
|  | C. B. Nwatu | 2022 | Improving access to diabetes care in Nigeria - the GIFSHIP opening | Subject |
|  | C.B. Nwatu | 2022 | Improving access to diabetes care in Nigeria - the GIFSHIP opening | Repetition |
|  | M. R. Massoud, F. Shakir, N. Livesley, M. Muhire, J. Nabwire et al. | 2015 | Improving care for patients on antiretroviral therapy through a gap analysis framework | Subject |
|  | P. H. Park, C. K. Wambui, S. Atieno, J. R. Egger, L. Misoi et al. | 2015 | Improving Diabetes Management and Cardiovascular Risk Factors Through Peer-Led Self-management Support Groups in Western Kenya | Subject |
|  | G. V. Gill, K. R. Huddle and L. P. Krige | 1986 | Improving diabetic control in adverse social conditions. A home blood glucose monitoring study in Soweto, South Africa | Subject |
|  | G. Barnabee, G. O'Bryan, L. Ndeikemona, I. Billah, L. Silas et al. | 2022 | Improving HIV pre-exposure prophylaxis persistence among adolescent girls and young women: Insights from a mixed-methods evaluation of community, hybrid, and facility service delivery models in Namibia | Subject |
|  | C. K. Or, K. Liu, M. K. P. So, B. Cheung, L. Y. C. Yam et al. | 2004 | Improving Self-Care in Patients With Coexisting Type 2 Diabetes and Hypertension by Technological Surrogate Nursing: Randomized Controlled Trial | Methods |
|  | H. Amu, T. Y. Brinsley, F. O. Kwafo, S. Amu and L. E. Bain | 2023 | Improving investment in chronic disease care in Sub-Saharan Africa is crucial for the achievement of SDG 3.4: application of the chronic care model | Subject |
|  | E. O. Okoro and B. A. Oyejola | 2004 | Inadequate control of blood pressure in Nigerians with diabetes | Methods |
|  | L. Badzek, S. C. Hines and A. H. Moss | 1998 | Inadequate self-care knowledge among elderly hemodialysis patients: assessing its prevalence and potential causes | Methods |
|  | A. Alotaibi, L. Perry, L. Gholizadeh and A. Al-Ganmi | 2017 | Incidence and prevalence rates of diabetes mellitus in Saudi Arabia: An overview | Subject |
|  | J. Dawson, K. Lambert, K. L. Campbell and J. T. Kelly | 2021 | Incorporating digital platforms into nutritional care in chronic kidney disease | Subject |
|  | R. Masekela, C. L. Gray, R. J. Green, A. I. Manjra, F. E. Kritzinger et a. | 2018 | The increasing burden of asthma in South African children: a call to action | Subject |
|  | R. Masekela, C. L. Gray, R. J. Green, A. I. Manjra, F. E. Kritzinger et al. | 2022 | The increasing burden of asthma in South African children: a call to action | Subject |
|  | https://www.samj.org.za/index.php/samj/article/view/12337/8547 | 2022 |  | Invalid |
|  | C. Mutyambizi, M. Pavlova, C. Hongoro and W. Groot | 2020 | Inequalities and factors associated with adherence to diabetes self-care practices amongst patients at two public hospitals in Gauteng, South Africa | Methods |
|  | M. Abdelgadir, M. Elbagir, M. Eltom and C. Berne | 2006 | The influence of glucose self-monitoring on glycaemic control in patients with diabetes mellitus in Sudan | Subject |
|  | A. K. Salami, O. O. Desalu, P. O. Adeoye, A. A. Akanbi Ii, A. E. Oguntoyinbo and A. Fadeyi | 2010 | Influence of performance status on the risk and outcome of nosocomial pneumonia in the elderly admitted to an open medical ward | Subject |
|  | F. A. Amer, M. S. Mohamed, A. I. Elbur, S. I. Abdelaziz and Z. A. Elrayah | 2018 | Influence of self-efficacy management on adherence to self-care activities and treatment outcome among diabetes mellitus type 2 | Subject |
|  | F. A. Amer, M. S. Mohamed, A. I. Elbur, S. I. Abdelaziz and Z. A. Elrayah | 2018 | Influence of self-efficacy management on adherence to self-care activities and treatment outcome among diabetes mellitus type 2 Sudanese patients | Subject |
|  | E. Kumah, E. K. Afriyie, A. A. Abuosi, S. E. Ankomah, A. Fusheini and G. Otchere | 2021 | Influence of the Model of Care on the Outcomes of Diabetes Self-Management Education Program: A Scoping Review | Review |
|  | G. Mattison, O. Canfell, D. Forrester, C. Dobbins, D. Smith et al. | 2022 | The Influence of Wearables on Health Care Outcomes in Chronic Disease: Systematic Review | Review |
|  | C. D. Samuel-Hodge, S. W. Headen, A. H. Skelly, A. F. Ingram, T. C. Keyserling et al. | 2000 | Influences on day-to-day self-management of type 2 diabetes among African-American women: spirituality, the multi-caregiver role, and other social context factors | Subject |
|  | C. D. Samuel-Hodge, S. W. Headen, A. H. Skelly, A. F. Ingram, T. C. Keyserling, E. J. Jackson et al. | 2000 | Influences on day-to-day self-management of type 2 diabetes among African-American women: spirituality, the multi-caregiver role, and other social context factors | Repetition |
|  | H. Bao, J. Liu and J. Ye | 2018 | Influencing Factors of the Diabetes Distress among Chinese Patients with Type 2 Diabetes Mellitus | Population |
|  | A. Zimmerman, L. K. Barcenas, M. Pesambili, F. Sakita, S. Mallya et al. | 2022 | Injury characteristics and their association with clinical complications among emergency care patients in Tanzania | Subject |
|  | G. D. Shannon, H. Haghparast-Bidgoli, W. Chelagat, J. Kibachio and J. Skordis-Worrall | 2019 | Innovating to increase access to diabetes care in Kenya: an evaluation of Novo Nordisk's base of the pyramid project | Subject |
|  | S. Tonen-Wolyec, S. Batina-Agasa, J. D. D. Longo, R.-S. Mboumba Bouassa and L. Bélec | 2019 | Insufficient education is a key factor of incorrect interpretation of HIV self-test results by female sex workers in Democratic Republic of the Congo: A multicenter cross-sectional study | Subject |
|  | S. Tonen-Wolyec, S. Batina-Agasa, J. D. D. Longo, R.-S. Mboumba Bouassa, L. Bélec et al. | 2019 | Insufficient education is a key factor of incorrect interpretation of HIV self-test results by female sex workers in Democratic Republic of the Congo: A multicenter cross-sectional study | Subject |
|  | F. T. Lester | 1985 | Insulin therapy: problems in an African country | Subject |
|  | T. T. Frakking, J. Waugh, H.-J. Teoh, D. Shelton, S. Moloney et al. | 2018 | Integrated children's clinic care (ICCC) versus a self-directed care pathway for children with a chronic health condition: a multi-centre randomised controlled trial study protocol | Subject |
|  | E. Blynn, E. Harris, M. Wendland, C. Chang, D. Kasungami et al. | 2021 | Integrating Human-Centered Design to Advance Global Health: Lessons From 3 Programs | Subject |
|  | E. Blynn, E. Harris, M. Wendland, C. Chang, D. Kasungami et al. | 2021 | Integrating human-centered design to advance global health: lessons from 3 programs | Repetition |
|  | E. Blynn, E. Harris, M. Wendland, C. Chang, D. Kasungami et al. | 2021 | Integrating Human-Centered Design to Advance Global Health: Lessons From 3 Programs | Subject |
|  | E. M. Ba-Essa, E. I. Mobarak, A. Alghamdi and N. M. Al-Daghri | 2015 | Intensified glucose self-monitoring with education in Saudi DM patients | Subject |
|  | H. A. Pérez, A. O. Adeoye, L. Aballay, L. A. Armando and N. H. García | 2021 | An intensive follow-up in subjects with cardiometabolic high-risk | Subject |
|  | O. Essien, A. Otu, V. Umoh, O. Enang, J. P. Hicks and J. Walley | 2017 | Intensive Patient Education Improves Glycaemic Control in Diabetes Compared to Conventional Education: A Randomised Controlled Trial in a Nigerian Tertiary Care Hospital | Subject |
|  | E. S. Albeltagy, S. Y. A. Elaziz, S. Y. Abozaid, H. M. El Zomor and S. S. A. Elhamed | 2021 | Interleukin 6, interleukin 17, disease-related and contextual factor association with depression, and its severity in patients with rheumatoid arthritis | Subject |
|  | F. E. MacVane Phipps | 2021 | Interleukin 6, interleukin 17, disease-related and contextual factor association with depression, and its severity in patients with rheumatoid arthritis | Subject |
|  | W. M. Freysteinson | 2009 | International reflections on knowledge and use of the mirror in nursing practice | Subject |
|  | S. C. Iregbu, W. Duggleby, J. Spiers and B. Salami | 2022 | An Interpretive Description of Sociocultural Influences on Diabetes Self-Management Support in Nigeria | Subject |
|  | H. Bao | 2022 | Intervention effect of mindfulness-based cognitive therapy on diabetes-related distress and self-care | Subject |
|  | H Bao | 2022 | Intervention effect of mindfulness-based cognitive therapy on diabetes-related distress and self-care | Repetition |
|  | H. A. Areri, A. Marshall and G. Harvey | 2020 | Interventions to improve self-management of adults living with HIV on Antiretroviral Therapy: A systematic review | Subject |
|  | E. Asante | 2013 | Interventions to promote treatment adherence in type 2 diabetes mellitus | Subject |
|  | E. Katushabe, J. B. Asiimwe and V. Batwala | 2022 | Intimate partner violence disclosure and associated factors among pregnant women attending a city hospital in South-Western Uganda: a cross-sectional study | Subject |
|  | S. Pillay and C. Aldous | 2016 | Introducing a multifaceted approach to the management of diabetes mellitus in resource-limited settings | Subject |
|  | B. M. Kwan, L. M. Dickinson, R. E. Glasgow, M. Sajatovic, M. Gritz et al. | 2020 | The Invested in Diabetes Study Protocol: a cluster randomized pragmatic trial comparing standardized and patient-driven diabetes shared medical appointments | Subject |
|  | A. T. Choko, K. Fielding, N. Stallard, H. Maheswaran, A. Lepine et al. | 2017 | Investigating interventions to increase uptake of HIV testing and linkage into care or prevention for male partners of pregnant women in antenatal clinics in Blantyre, Malawi: study protocol for a cluster randomised trial | Subject |
|  | S. Cleary, S. Birch, N. Chimbindi, S. Silal and D. McIntyre | 2013 | Investigating the affordability of key health services in South Africa | Subject |
|  | https://www.sciencedirect.com/science/journal/02779536 | 2018 |  | Invalid |
|  | https://www.sciencedirect.com/science/journal/02779536 | 1999 | Is care and support associated with preventive behaviour among people with HIV? | Subject |
|  | P. Oyaro, Z. Kwena, E. A. Bukusi and J. M. Baeten | 2020 | Is HIV Self-Testing a Strategy to Increase Repeat Testing Among Pregnant and Postpartum Women? A Pilot Mixed Methods Study | Subject |
|  | A. E. Strode, H. v. Rooyen and T. Makusha | 2013 | Is it lawful to offer HIV self-testing to children in South Africa? | Subject |
|  | https://www.sajhivmed.org.za/index.php/sajhivmed/article/view/987/876 | 2019 |  | Invalid |
|  | G. N. Nyaaba, K. Stronks, K. Meeks, E. Beune, E. Owusu-Dabo, J. Add et al. | 2019 | Is social support associated with hypertension control among Ghanaian migrants in Europe and non-migrants in Ghana? The RODAM study | Population |
|  | S. Lesage, E. Deacon, E. Van Rensburg and D. Segal | 2021 | 'It kinda sucks': Illness perception of a group of South African adolescents with type 1 diabetes mellitus | Population |
|  | R. C. Bonadonna, A. Giaccari, R. Buzzetti, G. Aimaretti, D. Cucinotta et al. | 2019 | Italian Titration Approach Study (ITAS) with insulin glargine 300 U/mL in insulin-naïve type 2 diabetes: Design and population | Subject |
|  | N. K. Mutea and C. M. Baker | 2008 | Kenyan nurses' involvement in managing hospitalized diabetic patients | Subject |
|  | R. N. Okoro, I. Ummate, J. D. Ohieku, S. I. Yakubu, M. O. Adibe and M. J. Okonta | 2020 | Kidney Disease Knowledge and Its Determinants Among Patients With Chronic Kidney Disease | Population |
|  | P. Roth, C. Y. Tang, B. Rumbold and S. Gupta | 2022 | Knowledge and perceptions around self-management of type 2 diabetes among a Sudanese community in Australia: a qualitative study | Population |
|  | P. Roth, C. Y. Tang, B. Rumbold and S. Gupta | 2022 | Knowledge and perceptions around self-management of type 2 diabetes among a Sudanese community in Australia: a qualitative study | Population |
|  | P. Roth, C. Y. Tang, B. Rumbold and S. Gupta | 2022 | Knowledge and perceptions around self-management of type 2 diabetes among a Sudanese community in Australia: A qualitative study | Population |
|  | P. Manickum, T. Mashamba-Thompson, R. Naidoo, S. Ramklass and T. Madiba | 2021 | Knowledge and practice of diabetic foot care - A scoping review | Review |
|  | L. S. Tuglo, F. K. Nyande, P. D. Agordoh, E. B. Nartey, Z. Pan et al. | 2021 | Knowledge and practice of diabetic foot care and the prevalence of diabetic foot ulcers among diabetic patients of selected hospitals in the Volta Region, Ghana | Methods |
|  | L. S. Tuglo, F. K. Nyande, P. D. Agordoh, E. B. Nartey, Z. Pan et al. | 2015 | Knowledge and practice of diabetic foot care and the prevalence of diabetic foot ulcers among diabetic patients of selected hospitals in the Volta Region, Ghana | Methods |
|  | L. S. Tuglo, F. K. Nyande, P. D. Agordoh, E. B. Nartey, Z. Pan et al. | 2022 | Knowledge and practice of diabetic foot care and the prevalence of diabetic foot ulcers among diabetic patients of selected hospitals in the Volta Region, Ghana | Repetition |
|  | L. S. Tuglo, F. K. Nyande, P. D. Agordoh, E. B. Nartey, Z. Pan et al. | 2022 | Knowledge and practice of diabetic foot care and the prevalence of diabetic foot ulcers among diabetic patients of selected hospitals in the Volta Region, Ghana | Repetition |
|  | S. A. Ahmed, S. Badi, H. Tahir, M. H. Ahmed and A. O. Almobarak | 2019 | Knowledge and practice of diabetic foot care in Sudan: A cross sectional survey | Methods |
|  | A. Tuha, A. Getie Faris, A. Andualem and S. Ahmed Mohammed | 2021 | Knowledge and Practice on Diabetic Foot Self-Care and Associated Factors Among Diabetic Patients at Dessie Referral Hospital, Northeast Ethiopia: Mixed Method | Methods |
|  | B. F. Dele-Ojo, T. H. Raimi, J. O. Fadare, E. A. Ajayi, D. D. Ajayi et al. | 2021 | Knowledge and Prevalence of Heart Disease Risk Factors Among Staff of a Tertiary Institution in Nigeria | Methods |
|  | E. G. Wamucii, F. Kyallo and B. Kiage | 2020 | Knowledge and self-care practices among diabetic patients- a case study of Thika level 5 Hospital, Kenya. | Methods |
|  | H. L. Wella and J. Protas | 2016 | Knowledge evaluation and educating diabetic patients on self-foot care at diabetic out patients' clinic, Muhimbili National Hospital | Methods |
|  | Lawrece Sena Tuglo | 2022 | Knowledge and practice of diabetic foot care and the prevalence of diabetic foot ulcers among diabetic patients of selected hospitals in the Volta Region, Ghana | Repetition |
|  | S. Yang, W. Kong, C. Hsue, A. F. Fish, Y. Chen et al. | 2016 | Knowledge of A1c Predicts Diabetes Self-Management and A1c Level among Chinese Patients with Type 2 Diabetes | Population |
|  | R. A. Afaya, V. Bam, T. B. Azongo and A. Afaya | 2020 | Knowledge of chronic complications of diabetes among persons living with type 2 diabetes mellitus in northern Ghana | Methods |
|  | M. J. Hashim, H. Mustafa and H. Ali | 2016 | Knowledge of diabetes among patients in the United Arab Emirates and trends since 2001: a study using the Michigan Diabetes Knowledge Test | Population |
|  | M. J. Hashim, H. Mustafa and H. Ali | 2017 | Knowledge of diabetes among patients in the United Arab Emirates and trends since 2001: a study using the Michigan Diabetes Knowledge Test | Population |
|  | D. P. Perera, R. E. E. De Silva and W. L. S. P. Perera | 2013 | Knowledge of diabetes among type 2 diabetes patients attending a primary health care clinic in Sri Lanka | Setting |
|  | E. O. Owolabi, D. T. Goon, A. I. Ajayi and O. V. Adeniyi | 2022 | Knowledge of diabetes and associated factors in rural Eastern Cape, South Africa: A cross sectional study | Methods |
|  | D. Magurová, L. Majerníkova, S. Hloch, H. Tozan and K. Goztepe | 2012 | Knowledge of diabetes in patients with type 2 diabetes on insulin therapy from eastern Slovakia | Setting |
|  | U. S. Jasper, M. C. Opara, E. B. Pyiki and O. Akinrolie | 2014 | Knowledge of insulin use and its determinants among Nigerian insulin requiring diabetes patients | Subject |
|  | I. L. Jackson, M. O. Adibe, M. J. Okonta and C. V. Ukwe | 2014 | Knowledge of self-care among type 2 diabetes patients in two states of Nigeria | Methods |
|  | A. Ejegi, A. J. Ross and K. Naidoo | 2016 | Knowledge of symptoms and self-management of hypoglycaemia amongst patients attending a diabetic clinic at a regional hospital in KwaZulu-Natal | Methods |
|  | B. A. Owusu, P. Ofori-Boateng, A. Forbes and D. T. Doku | 2022 | Knowledge of young people living with type 1 diabetes and their caregivers about its management | Population |
|  | B. A. Owusu, P. Ofori-Boateng, A. Forbes and D. T. Doku | 2022 | Knowledge of young people living with type 1 diabetes and their caregivers about its management | Population |
|  | A. R. Khan, Z. N. Al Abdul Lateef, M. B. Khamseen, M. A. Al Aithan et al. | 2011 | Knowledge, attitude and practice of ministry of health primary health care physicians in the management of type 2 diabetes mellitus: a cross-sectional study in the Al Hasa District of Saudi Arabia, 2010 | Subject |
|  | N. Beshir Bedru, B. Miftah Shafi and M. Oumer Sada | 2021 | Knowledge, attitude and practice towards insulin self-administration and associated factors among diabetic patients at Zewditu Memorial Hospital, Ethiopia | Methods |
|  | B. B. Nasir, M. S. Buseir and O. S. Muhammed | 2021 | Knowledge, attitude and practice towards insulin self-administration and associated factors among diabetic patients at Zewditu Memorial Hospital, Ethiopia | Repetition |
|  | B. B. Nasir, M. S. Buseir and O. S. Muhammed | 2021 | Knowledge, attitude and practice towards insulin self-administration and associated factors among diabetic patients at Zewditu Memorial Hospital, Ethiopia | Subject |
|  | M. Enyew Getaneh | 2021 | Knowledge, attitude, and practice towards self-management among diabetic patients at Debre Tabor General Hospital chronic diseases follow-up clinic, Northwest Ethiopia | Methods |
|  | <https://link.springer.com/article/10.1007/s13410-020-00896-3> | 2022 | Knowledge, attitude, and practice towards self-management among diabetic patients at Debre Tabor General Hospital chronic diseases follow-up clinic, Northwest Ethiopia | Repetition |
|  | E. G. Mekonen | 2021 | Knowledge, attitude, and practice towards self-management among diabetic patients at Debre Tabor General Hospital chronic diseases follow-up clinic, Northwest Ethiopia | Methods |
|  | M. Kaddumukasa, J. Kayima, M. N. Kaddumukasa, E. Ddumba, L. Mugenyi et al. | 2015 | Knowledge, attitudes and perceptions of stroke: a cross-sectional survey in rural and urban Uganda | Subject |
|  | Y. Kebede, Y. Yitayih, Z. Birhanu, S. Mekonen and A. Ambelu | 2021 | Knowledge, perceptions and preventive practices towards COVID-19 early in the outbreak among Jimma university medical center visitors, Southwest Ethiopia | Subject |
|  | K. Yohannes, Y. Yimenu, B. Zewdie, M. Seblework and A. Argaw | 2020 | Knowledge, perceptions and preventive practices towards COVID-19 early in the outbreak among Jimma University medical center visitors, Southwest Ethiopia | Subject |
|  | https://journals.plos.org/plosone/article?id=10.1371/journal.pone.0233744 | 2019 | Knowledge, perceptions and preventive practices towards COVID-19 early in the outbreak among Jimma University medical center visitors, Southwest Ethiopia | Subject |
|  | J. C. N. Chan, L.-L. Lim, N. J. Wareham, J. E. Shaw, T. J. Orchard, P. Zhang et al. | 2021 | The Lancet Commission on diabetes: using data to transform diabetes care and patient lives | Subject |
|  | A de G. Aikins, A Anum , C Agyemang , J Addo , O Ogedegbe et al. | 2012 | Lay Representations of Chronic Diseases in Ghana: Implications for Primary Prevention | Subject |
|  | A. Siika, L. McCabe, M. Bwakura-Dangarembizi, C. Kityo, J. Mallewa et al. | 2018 | Late Presentation With HIV in Africa: Phenotypes, Risk, and Risk Stratification in the REALITY Trial | Repetition |
|  | A. Siika, L. McCabe, M. Bwakura-Dangarembizi, C. Kityo, J. Mallewa et al. | 2008 | Late Presentation With HIV in Africa: Phenotypes, Risk, and Risk Stratification in the REALITY Trial | Repetition |
|  | I. Hatzipapas, M. J. Visser and E. Janse van Rensburg | 2017 | Laughter therapy as an intervention to promote psychological well-being of volunteer community care workers working with HIV-affected families | Repetition |
|  | I. Hatzipapas, M. J. Visser and E. J. v. Rensburg | 2017 | Laughter therapy as an intervention to promote psychological well-being of volunteer community care workers working with HIV-affected families | Repetition |
|  | https://www.tandfonline.com/doi/full/10.1080/17290376.2017.1402696 | 2017 | Laughter therapy as an intervention to promote psychological well-being of volunteer community care workers working with HIV-affected families | Repetition |
|  | G. Gilworth, S. Lewin, A. J. Wright, S. J. Taylor, R. Tuffnell et al. | 2019 | The lay health worker-patient relationship in promoting pulmonary rehabilitation (PR) in COPD: What makes it work? | Subject |
|  | H. Hunt-Smith and M. Butler | 2018 | LEADS Case Study: Partnering to Offer a Diabetes Management e-Learning Module to Nursing and Allied Health Staff | Subject |
|  | S. Kalra, G. Priya, E. Grewal, T. T. Aye, B. K. Waraich et al. | 2018 | Lessons for the Health-care Practitioner from Buddhism | Subject |
|  | N. Yaima, S. Ningthouja, D. Sharma, L. Bijaya, K. Shyamkanhai et al. | 1995 | Lessons from home based care for persons affecting with HIV and AIDS | Subject |
|  | E. R. Goethals, B. Soenens, M. de Wit, M. Vansteenkiste, L. M. Laffel et al. | 2019 | Let's talk about it The role of parental communication in adolescents' motivation to adhere to treatment recommendations for type 1 diabetes | Subject |
|  | E. Mufunda, K. Wikby, A. Björn and K. Hjelm | 2012 | Level and determinants of diabetes knowledge in patients with diabetes in Zimbabwe: a cross-sectional study | Subject |
|  | D. B. Ketema, C. T. Leshargie, G. D. Kibret, M. A. Assemie, A. A. Alamneh et al. | 2020 | Level of self-care practice among diabetic patients in Ethiopia: a systematic review and meta-analysis | Subject |
|  | A. A. Uagboe, A. Ogboye, M. M. Munee, K. J. Rahman, J. K. Akpakli and N. Mobisson | 2022 | Leveraging technology to support people living with hypertension and diabetes during the COVID-19 pandemic | Subject |
|  | E. Mohr, L. Snyman, Z. Mbakaz, J. Caldwell, V. DeAzevedo et al. | 2018 | Life continues: Patient, health care and community care workers perspectives on self-administered treatment for rifampicin-resistant tuberculosis in Khayelitsha, South Africa | Subject |
|  | K. E. Chademana and B. van Wyk | 2021 | Life in a child-headed household: Exploring the quality of life of orphans living in child-headed households in Zimbabwe | Subject |
|  | D. Burnett and C. Condren | 2016 | Life-Threatening Asthma in Pediatric Patients: Identifying and Minimizing the Risk | Subject |
|  | J. Wu, J. Huang, M. C. Battié and Y. Wang | 2020 | Lifestyle and lifetime occupational exposures may not play a role in the pathogenesis of Modic changes on the lumbar spine MR images | Subject |
|  | L. Rawal, B. W. Sahle, B. J. Smith, K. Kanda, E. Owusu-Addo and A. M. N. Renzaho | 2021 | Lifestyle interventions for type 2 diabetes management among migrants and ethnic minorities living in industrialized countries: a systematic review and meta-analyses | Review |
|  | E. Mufunda, Å. Ernersson and K. Hjelm | 2018 | Limited knowledge of diabetes in patients attending an outpatient diabetes clinic at a referral hospital in Zimbabwe: a cross-sectional study | Subject |
|  | J. Hearn, Q. Pham, J. I. Schwartz, I. Ssinabulya, A. R. Akiteng et al. | 2020 | Lived Experiences and Technological Literacy of Heart Failure Patients and Clinicians at a Cardiac Care Centre in Uganda | Subject |
|  | J. Hearn, Q. Pham, J. I. Schwartz, I. Ssinabulya, A. R. Akiteng et al. | 2020 | Lived Experiences and Technological Literacy of Heart Failure Patients and Clinicians at a Cardiac Care Centre in Uganda | Repetition |
|  | L. S. Kahn, B. M. Vest, R. Karl, L. Tumiel-Berhalter, R. Taylor et al. | 2013 | Living with diabetes on Buffalo, New York’s culturally diverse West Side | Setting |
|  | M. M. Popoola | 2005 | Living with diabetes: The holistic experiences of Nigerians and African Americans | Population |
|  | L. Mbuagbaw, R. Aronson, A. Walker, R. E. Brown and N. Orzech | 2017 | The LMC Skills, Confidence & Preparedness Index (SCPI): development and evaluation of a novel tool for assessing self-management in patients with diabetes | Subject |
|  | D. F. Conserve, J. Michel, J. E. Adrien Demes, J. M. Chéry et al. | 2020 | Local and national stakeholders' perceptions towards implementing and scaling up HIV self-testing and secondary distribution of HIV self-testing by Option B+ patients as an assisted partner service strategy to reach men in Haiti | Subject |
|  | W. Anderson-Loftin and L. Moneyham | 2000 | Long-term disease management needs of southern African Americans with diabetes | Subject |
|  | N. M. Ngongo, G. Darcis, H. S. Nanituna, M. M. Mambimbi, N. Maes, M. L. Mashi et al. | 2021 | Longitudinal analysis of sociodemographic, clinical and therapeutic factors of HIV-infected individuals in Kinshasa at antiretroviral therapy initiation during 2006-2017 | Subject |
|  | N. M. Ngongo, G. Darcis, H. S. Nanituna, M. M. Mambimbi, N. Maes, M. L. Mashi et al. | 2020 | Longitudinal analysis of sociodemographic, clinical and therapeutic factors of HIV-infected individuals in Kinshasa at antiretroviral therapy initiation during 2006-2017 | Repetition |
|  | A. A. Andargie and M. A. Zeru | 2018 | A longitudinal data analysis on risk factors for developing type-2 diabetes mellitus at the University of Gondar Comprehensive Specialized Hospital, Gondar, Ethiopia | Subject |
|  | <https://academicjournals.org/journal/JPHE/article-abstract/> | 2019 |  | Invalid |
|  | A. Greco, E. R. Cappelletti, K. Luyckx, M. D'Addario, C. Giannattasio and P. Steca | 2018 | A longitudinal inquiry into directionality of effects between coping and information needs in hypertensive patients | Subject |
|  | R. B. Strandberg, M. Graue, T. Wentzel-Larsen, M. Peyrot et al. | 2015 | Longitudinal relationship between diabetes-specific emotional distress and follow-up HbA1c in adults with Type 1 diabetes mellitus | Subject |
|  | W. Sun, H. Lu, F. Huang, C. Shiu, L. Zhang and W. Chen | 2022 | Longitudinal trajectory of the association between quality of life and depression among people living with HIV in China: a mixed effects model | Subject |
|  | <https://www.tandfonline.com/doi/full/10.1080/09540121.2021> |  |  | Invalid |
|  | F. Bekele and D. Berhanu | 2021 | Loss of a limb is not loss of a life. Knowledge and attitude on diabetic foot ulcer care and associated factors among diabetic mellitus patients on chronic care follow-up of southwestern Ethiopian hospitals: A multicenter cross-sectional study | Methods |
|  | C. J. Vrana-Diaz, J. E. Korte, M. Gebregziabher, L. Richey, A. Selassie et al. | 2021 | Low acceptance of intimate partner violence by pregnant women in Uganda predicts higher uptake of HIV self-testing among their male partners | Subject |
|  | https://www.tandfonline.com/doi/abs/10.2989/16085906.2021.2000449 | 2021 | Low acceptance of intimate partner violence by pregnant women in Uganda predicts higher uptake of HIV self-testing among their male partners | Repetition |
|  | A. Oluma, M. Abadiga, G. Mosisa and W. Etafa | 2021 | Magnitude and predictors of poor glycemic control among patients with diabetes attending public hospitals of Western Ethiopia | Subject |
|  | T. Mariye, H. Tasew, G. Teklay, H. Gerensea and W. Daba | 2018 | Magnitude of diabetes self-care practice and associated factors among type two adult diabetic patients following at public Hospitals in central zone, Tigray Region, Ethiopia, 2017 | Methods |
|  | A. K. Wahl, R. H. Osborne, E. Langeland, T. Wentzel-Larsen, A. M. Mengshoel et al. | 2016 | Making robust decisions about the impact of health education programs: Psychometric evaluation of the Health Education Impact Questionnaire (heiQ) in diverse patient groups in Norway | Subject |
|  | F. Bekele, F. Kelifa and B. Sefera | 2013 | A male ' s foot is being shot by an ulcer, not a gunshot! The magnitude and associated factors of diabetic foot ulcer among diabetes mellitus patients on chronic care follow-up of southwestern Ethiopian hospital: A cross-sectional study | Methods |
|  | J. C. Mbanya, P. Naidoo, B. A. Kolawole, E. Tsymbal, A. McMaster, S. Karamchand et al. | 2020 | Management of adult patients with type 1 diabetes mellitus in Africa: A post-hoc cohort analysis of 12 African countries participating in the International Diabetes Management Practices Study (Wave 7) | Methods |
|  | J. C. Mbanya, P. Naidoo, B. A. Kolawole, E. Tsymbal, A. McMaster, S. Karamchand et al. | 2020 | Management of adult patients with type 1 diabetes mellitus in Africa: a post-hoc cohort analysis of 12 African countries participating in the international diabetes management practices study (Wave 7) | Methods |
|  | https://journals.lww.com/md-journal/Fulltext/2020/06190/Management_of_adult_patients_with_type_1_diabetes.24.aspx | 2020 | Management of adult patients with type 1 diabetes mellitus in Africa: A post-hoc cohort analysis of 12 African countries participating in the International Diabetes Management Practices Study (Wave 7) | Methods |
|  | J. v. Olmen, F. Schellevis, W. v. Damme, G. Kegels and F. Rasschaert | 2012 | Management of chronic diseases in sub-Saharan Africa: cross-fertilisation between HIV/AIDS and diabetes care | Subject |
|  | L. Drown; A. J. Adler; L. N. Schwartz; J. Sichali; F. Valeta et al. | 2023 | Living with type 1 diabetes in Neno, Malawi: a qualitative study of self-management and experiences in care | Included |
|  | J. van Olmen, F. Schellevis, W. Van Damme, G. Kegels and F. Rasschaert | 2012 | Management of Chronic Diseases in Sub-Saharan Africa: Cross-Fertilisation between HIV/AIDS and Diabetes Care | Repetition |
|  | H. Amu, E. K. M. Darteh, E. E. Tarkang and A. Kumi-Kyereme | 2021 | Management of chronic non-communicable diseases in Ghana: a qualitative study using the chronic care model | Included |
|  | https://link.springer.com/article/10.1186/s12889-021-11170-4 | 2021 | Management of chronic non-communicable diseases in Ghana: a qualitative study using the chronic care model | Repetition |
|  | J. Hill | 2012 | Management of diabetes in South Asian communities in the UK | Population |
|  | K. Hjelm, K. Bard, P. Nyberg and J. Apelqvist | 2007 | Management of gestational diabetes from the patient's perspective - a comparison of Swedish and Middle-Eastern born women | Subject |
|  | K. Hjelm, K. Bard, P. Nyberg and J. Apelqvist | 2007 | Management of gestational diabetes from the patient's perspective – a comparison of Swedish and Middle-Eastern born women | Repetition |
|  | K. Hjelm, K. Bard, P. Nyberg and J. Apelqvist | 2007 | Management of gestational diabetes from the patient's perspective--a comparison of Swedish and Middle-Eastern born women | Repetition |
|  | L. Bernhard, P. Bernhard and P. Magnussen | 2003 | Management of patients with lymphoedema caused by filariasis in north-eastern Tanzania: alternative approaches | Subject |
|  | T. Steyl and J. Phillips | 2014 | Management of type 2 diabetes mellitus: adherence challenges in environments of low socio-economic status | Included |
|  | R. R. Jin, J. J. Li, J. Zhang, J. L. Li, F. Bian et al. | 2018 | [Management programs on diabetes among Chinese adults in the National Demonstration Areas for Comprehensive Prevention and Control of Non-communicable Diseases] | Population |
|  | I. Njuguna, K. Beima-Sofie, C. Mburu, C. Mugo, D. A. Black et al. | 2019 | Managing the transition from paediatric to adult care for HIV, Kenya | Population |
|  | C. Boult, B. Leff, C. M. Boyd, J. L. Wolff, J. A. Marsteller et al. | 2013 | A matched-pair cluster-randomized trial of guided care for high-risk older patients | Methods |
|  | A. Baumgart, K. E. Manera, D. W. Johnson, J. C. Craig, J. I. Shen et al. | 2020 | Meaning of empowerment in peritoneal dialysis: focus groups with patients and caregivers | Subject |
|  | T. Crowley, A. Van der Merwe, M. Kidd and D. Skinner | 2020 | Measuring Adolescent HIV Self-management: An Instrument Development Study | Population |
|  | C. M. Keene, A. Ragunathan, J. Euvrard, M. English, J. McKnight and C. Orrell | 2022 | Measuring patient engagement with HIV care in sub-Saharan Africa: a scoping study | Review |
|  | N. Desmond, N. Nagelkerke, W. Lora, E. Chipeta, M. Sambo et al. | 2018 | Measuring sexual behaviour in Malawi: a triangulation of three data collection instruments | Subject |
|  | The GBD 2019 Human Resources for Health Collaborators | 2022 | Measuring the availability of human resources for health and its relationship to universal health coverage for 204 countries and territories from 1990 to 2019: a systematic analysis for the Global Burden of Disease Study 2019 | Subject |
|  | E. Hill, R. Gagnon, R. Ramsing, J. Goff, B. Kennedy and T. Hooker | 2015 | Measuring the Impact of a Medical Specialty Camp | Subject |
|  | M. Pantelic, L. Cluver, M. Boyes, E. Toska, C. Kuo and M. Moshabela | 2015 | Medical pluralism predicts non-ART use among parents in need of ART: a community survey in KwaZulu-Natal, South Africa | Subject |
|  | G. Fetensa, B. Yadecha, T. Tolossa and T. T. Bekuma | 2019 | Medication Adherence and Associated Factors among Chronic Heart Failure Clients on Follow Up Oromia Region, West Ethiopia | Subject |
|  | Z. Jannoo and N. Mamode Khan | 2019 | Medication Adherence and Diabetes Self-Care Activities Among Patients With Type 2 Diabetes Mellitus | Methods |
|  | R. A. Afaya, V. Bam, T. B. Azongo, A. Afaya, A. Kusi-Amponsah et al. | 2020 | Medication adherence and self-care behaviours among patients with type 2 diabetes mellitus in Ghana | Methods |
|  | R. A. Afayaid, V. Bamid, T. B. Azongoid, A. Afaya, A. Kusi-Amponsah et al. | 2020 | Medication adherence and self-care behaviours among patients with type 2 diabetes mellitus in Ghana | Repetition |
|  | https://journals.plos.org/plosone/article?id=10.1371/journal.pone.0237710 | 2020 | Medication adherence and self-care behaviours among patients with type 2 diabetes mellitus in Ghana | Methods |
|  | N. T. Wabe, M. T. Angamo and S. Hussein | 2011 | Medication adherence in diabetes mellitus and self management practices among type-2 diabetics in Ethiopia | Methods |
|  | S. J. Liau, S. Lalic, J. K. Sluggett, M. Cesari, G. Onder et al. | 2021 | Medication Management in Frail Older People: Consensus Principles for Clinical Practice, Research, and Education | Subject |
|  | C. R. Sewani-Rusike and M. Mammen | 2014 | Medicinal plants used as home remedies: a family survey by first year medical students | Subject |
|  | M. Ocan, M. Aono, C. Bukirwa, E. Luyinda, C. Ochwo et al. | 2017 | Medicine use practices in management of symptoms of acute upper respiratory tract infections in children (≤12 years) in Kampala city, Uganda | Subject |
|  | https://bmcpublichealth.biomedcentral.com/track/pdf/10.1186/s12889-017-4770-1 | 2017 | Medicine use practices in management of symptoms of acute upper respiratory tract infections in children (≤12 years) in Kampala city, Uganda | Repetition |
|  | R. Bonadonna | 2003 | Meditation's impact on chronic illness | Subject |
|  | L. Hattingh | 2017 | Medscheme Mental Health Programme | subject |
|  | M. Hlongwa, T. Mashamba-Thompson, S. Makhunga, C. Muraraneza and K. Hlongwana | 2020 | Men's perspectives on HIV self-testing in sub-Saharan Africa: a systematic review and meta-synthesis | Review |
|  | S. Russell | 2019 | Men's Refashioning of Masculine Identities in Uganda and Their Self-Management of HIV Treatment | Subject |
|  | P. Memiah, L. Nkinda, M. Majigo, F. Humwa, Z. Haile et al. | 2021 | Mental health symptoms and inflammatory markers among HIV infected patients in Tanzania | Subject |
|  | S.-F. Lo, F.-T. Lu, A.-C. O Yang, J.-L. Zeng, Y.-Y. Yang et al. | 2023 | Metabolic Syndrome-Related Knowledge, Attitudes, and Behavior among Indigenous Communities in Taiwan: A Cross-Sectional Study | Subject |
|  | H. Aboumatar, M. Naqibuddin, J. Neiman, J. Saunders, S. Kim, H. Chaudhry et al. | 2020 | Methodology and baseline characteristics of a randomized controlled trial testing a health care professional and peer-support program for patients with chronic obstructive pulmonary disease: The BREATHE2 study | subject |
|  | E. C. Uk, V. Cooper, J. Clatworthy and J. Whetham | 2017 | mHealth interventions to support self-management in HIV: a systematic review | Subject |
|  | https://benthamopen.com/contents/pdf/TOAIDJ/TOAIDJ-11-119.pdf | 2017 | mHealth interventions to support self-management in HIV: a systematic review | Subject |
|  | S. A. Quandt, A. Groeschel-Johnson, H. T. Kinzer, A. Jensen, K. Miles et al. | 2018 | Migrant Farmworker Nutritional Strategies: Implications for Diabetes Management | Subject |
|  | K. Hjelm, K. Bard and J. Apelqvist | 2021 | Migrant Middle Eastern women with gestational diabetes seven years after delivery - positive long-term development of beliefs about health and illness shown in follow-up interviews | Subject |
|  | P. B. Aronowitz, D. M. Williams, M. C. Henderson and L. G. Winston | 2019 | Mind the Base Rate: an Exercise in Clinical Reasoning | subject |
|  | M. K. Matsuba and L. Williams | 2020 | Mindfulness and yoga self-care workshop for Northern Ugandan teachers: A pilot study | Subject |
|  | S. S. Maharaj and J. M. Nuhu | 2019 | Mini-trampoline rebound exercises: A 'self-care' initiative for glycated hemoglobin, body mass index and emotional distress for mildly obese females with non-insulin dependent type 2 diabetes | Subject |
|  | E. Mehraeen, S. SeyedAlinaghi, Z. Pashaei, P. Mirzapour, A. Barzegary et al. | 2022 | Mobile applications in HIV self-management: A systematic review of scientific literature | Subject |
|  | D. Constant, K. de Tolly, J. Harries and L. Myer | 2014 | Mobile phone messages to provide support to women during the home phase of medical abortion in South Africa: a randomised controlled trial | Subject |
|  | L. A. Nelson, K. A. Wallston, S. Kripalani, R. A. G. Jr, T. A. Elasy, E. M. Bergner et al. | 2018 | Mobile Phone Support for Diabetes Self-Care Among Diverse Adults: Protocol for a Three-Arm Randomized Controlled Trial | Subject |
|  | K. Bobrow, A. J. Farmer, D. Springer, M. Shanyinde, L.-M. Yu et al. | 2016 | Mobile Phone Text Messages to Support Treatment Adherence in Adults With High Blood Pressure (SMS-Text Adherence Support [StAR]): A Single-Blind, Randomized Trial | Subject |
|  | L. Mbuagbaw, S. Mursleen, L. Lytvyn, M. Smieja, L. Dolovich and L. Thabane | 2015 | Mobile phone text messaging interventions for HIV and other chronic diseases: an overview of systematic reviews and framework for evidence transfer | Subject |
|  | M. A. Olamoyegun, O. J. Emuoyibofarhe, O. A. Ala and E. Ugwu | 2020 | Mobile Phone Use in the Management of Diabetes in Nigeria: A New Potential Weapon | Subject |
|  | K. Berner, A. Gouelle, H. Strijdom, M. F. Essop, I. Webster and Q. Louw | 2021 | Mobility Deviations in Adults With Human Immunodeficiency Virus: A Cross-Sectional Assessment Using Gait Analysis, Functional Performance, and Self-Report | Subject |
|  | T. D. Bui, O. Kadzakumanja and C. Munthali | 2014 | Mobilizing for the Lilongwe Diabetes Peer Support Programme in Malawi | Subject |
|  | E. M. Robinson, P. Weaver, R. Chen, R. Streisand and C. S. Holmes | 2016 | A model of parental distress and factors that mediate its link with parental monitoring of youth diabetes care, adherence, and glycemic control | Subject |
|  | T. G. Diedricks, C. P. H. Myburgh and M. Poggenpoel | 2020 | A model to facilitate self-management of human immunodeficiency virus in students within a university setting and promoting their mental health | Subject |
|  | https://hsag.co.za/index.php/hsag/article/view/1069/html | 2020 | A model to facilitate self-management of human immunodeficiency virus in students within a university setting and promoting their mental health | Subject |
|  | S.-J. Chiou, Y.-J. Chang, K. Liao and C.-D. Chen | 2022 | Modest association between health literacy and risk for peripheral vascular disease in patients with type 2 diabetes | Subject |
|  | M. Kaddumukasa, J. Kayima, J. Nakibuuka, C. Blixen, E. Welter, E. Katabira et al. | 2017 | Modifiable lifestyle risk factors for stroke among a high risk hypertensive population in Greater Kampala, Uganda; a cross-sectional study | Subject |
|  | P. D. Home, G. B. Bolli, C. Mathieu, C. Deerochanawong, W. Landgraf, C. Candelas et al. | 2015 | Modulation of insulin dose titration using a hypoglycaemia-sensitive algorithm: insulin glargine versus neutral protamine Hagedorn insulin in insulin-naïve people with type 2 diabetes | Subject |
|  | J. de Klerk | 2020 | Monitoring the body: grandmothers' ability to provide 'expert' care for grandchildren living with HIV in northwest Tanzania | Subject |
|  | L. K. Twells, S. Driscoll, D. M. Gregory, K. Lester, J. M. Fardy and D. Pace | 2017 | Morbidity and health-related quality of life of patients accessing laparoscopic sleeve gastrectomy: a single-centre cross-sectional study in one province of Canada | Subject |
|  | M. Newmeyer, B. Keyes, S. Gregory, K. Palmer, D. Buford, P. Mondt et al. | 2014 | The Mother Teresa Effect: the modulation of spirituality in using the CISM model with mental health service providers | Subject |
|  | M. Newmeyer, B. Keyes, S. Gregory, K. Palmer, D. Buford, P. Mondt et al. | 2014 | The Mother Teresa Effect: the modulation of spirituality in using the CISM model with mental health service providers | Subject |
|  | S. K. Schmidt, L. Hemmestad, C. S. MacDonald, H. Langberg and L. S. Valentiner | 2020 | Motivation and Barriers to Maintaining Lifestyle Changes in Patients with Type 2 Diabetes after an Intensive Lifestyle Intervention (The U-TURN Trial): A Longitudinal Qualitative Study | Subject |
|  | O. Kähkönen, P. Kankkunen, T. Saaranen, H. Miettinen, H. Kyngäs and M.-L. Lamidi | 2015 | Motivation is a crucial factor for adherence to a healthy lifestyle among people with coronary heart disease after percutaneous coronary intervention | Subject |
|  | J. Mantell, J. Franks, A. Zerbe, M. R. Lamb, D. M. Reed, D. Omollo et al. | 2022 | MPrEP+ study protocol: a prospective cohort study assessing the feasibility and acceptability of an HIV pre-exposure prophylaxis (PrEP) strategy for male clients of female sex workers in Kisumu, Kenya | Subject |
|  | M. Darcy, J. Parkinson, L. Ball, L. Norton and J. Hobby | 2022 | Multicomponent approaches to promoting healthy behaviours in people with Type 2 diabetes: an integrative review | Subject |
|  | K. N. Kimani, S. A. Murray and L. Grant | 2018 | Multidimensional needs of patients living and dying with heart failure in Kenya: a serial interview study | Subject |
|  | J. C. Chan, J. J. Gagliardino, S. H. Baik, J. M. Chantelot, S. R. Ferreira et al. | 2009 | Multifaceted determinants for achieving glycemic control: the International Diabetes Management Practice Study (IDMPS) | Subject |
|  | J. C. N. Chan, J. J. Gagliardino, S. H. Baik, J.-M. Chantelot, S. R. G. Ferreira et al. | 2009 | Multifaceted determinants for achieving glycemic control: the International Diabetes Management Practice Study (IDMPS) | Subject |
|  | N. Bollegala, M. Barwick, N. Fu, A. M. Griffiths, L. Keefer et al. | 2022 | Multimodal intervention to improve the transition of patients with inflammatory bowel disease from pediatric to adult care: protocol for a randomized controlled trial | Subject |
|  | K. Palmer, A. Marengoni, M. J. Forjaz, E. Jureviciene, T. Laatikainen, F. Mammarella, C. Muth et al. | 2018 | Multimorbidity care model: Recommendations from the consensus meeting of the Joint Action on Chronic Diseases and Promoting Healthy Ageing across the Life Cycle (JA-CHRODIS) | Subject |
|  | W. S. Harris, K. F. Kennedy, T. M. Maddox, S. Kutty and J. A. Spertus | 2016 | Multiple differences between patients who initiate fish oil supplementation post-myocardial infarction and those who do not: the TRIUMPH Study | Subject |
|  | I. Aboderin and A. Nanyonjo | 2017 | Musculoskeletal health conditions among older populations in urban slums in sub-Saharan Africa | Subject |
|  | I. Aboderin and A. Nanyonjo | 2017 | Musculoskeletal health conditions among older populations in urban slums in sub-Saharan Africa | Repetition |
|  | C. A. O'Brien, D. Van Rooyen and S. Carlson | 2006 | National guidelines for the management of diabetes mellitus: a nursing perspective | Subject |
|  | J. W. Muchiri, G. J. Gericke and P. Rheeder | 2012 | Needs and preferences for nutrition education of type 2 diabetic adults in a resource-limited setting in South Africa | Subject |
|  | A. Ayitewala, I. Ssewanyana and C. Kiyaga | 2021 | Next generation sequencing based in-house HIV genotyping method: validation report | Subject |
|  | https://link.springer.com/article/10.1186/s12981-021-00390-8 | 2021 | Next generation sequencing based in-house HIV genotyping method: validation report | Subject |
|  | J. J. Bousquet, H. J. Schünemann, A. Togias, M. Erhola, P. W. Hellings, T. Zuberbier et al. | 2019 | Next-generation ARIA care pathways for rhinitis and asthma: a model for multimorbid chronic diseases | Subject |
|  | Y. Obirikorang, E. Acheampong, E. O. Anto, E. Afrifa-Yamoah, E. Adua, J. Taylor et al. | 2022 | Nexus between constructs of social cognitive theory model and diabetes self-management among Ghanaian diabetic patients: a mediation modelling approach | Subject |
|  | https://journals.plos.org/globalpublichealth/article?id=10.1371/journal.pgph.0000736 | 2022 | Nexus between constructs of social cognitive theory model and diabetes self-management among Ghanaian diabetic patients: a mediation modelling approach | Subject |
|  | S. Iregbu, J. Spiers, W. Duggleby, B. Salami and K. Schick-Makaroff | 2023 | Nigerian Health Care Providers and Diabetes Self-Management Support: Their Perspectives and Practices | Population |
|  | T. W. Abate, G. Dessie, Y. Workineh, H. Gedamu, M. Birhanu, E. Ayalew et al. | 2021 | Non-adherence to self-care and associated factors among diabetes adult population in Ethiopian: A systemic review with meta-analysis | Subject |
|  | B. K. Gelaw, A. Mohammed, G. T. Tegegne, A. D. Defersha, M. Fromsa, E. Tadesse et al. | 2014 | Nonadherence and contributing factors among ambulatory patients with antidiabetic medications in Adama Referral Hospital | Methods |
|  | A. D. Wake, T. S. Tuji, A. T. Sime, M. T. Mekonnin, T. M. Taji and A. A. Hussein | 2021 | Nonadherence to Self-Care Practices, Antihypertensive Medications, and Associated Factors among Hypertensive Patients in a Follow-up Clinic at Asella Referral and Teaching Hospital, Ethiopia: A Cross-Sectional Study | Methods |
|  | S. A. Greenfield, G. M. Cole, C. W. Coen, S. Frautschy, R. P. Singh, M. Mekkittikul et al. | 2022 | A novel process driving Alzheimer's disease validated in a mouse model: Therapeutic potential | Subject |
|  | F. Mano, K. Ikeda, Y. Uchida, I. T. H.-C. Liu, E. Joo et al. | 2019 | Novel psychosocial factor involved in diabetes self-care in the Japanese cultural context | Subject |
|  | R. E. Glasgow, C. E. Knoepke, D. Magid, G. K. Grunwald, T. J. Glorioso et al. | 2021 | The NUDGE trial pragmatic trial to enhance cardiovascular medication adherence: study protocol for a randomized controlled trial | Subject |
|  | H. Cooper, A. McMurray and J. Sands | 2017 | The Nurse Navigator: An Innovative Role to Optimise Patient Transitions Across Primary and Secondary Care Settings | Subject |
|  | S. Kirby, M. Moore, T. McCarron, D. Perkins and D. Lyle | 2015 | Nurse-led diabetes management in remote locations | Subject |
|  | F. B. Hailu, P. Hjortdahl and A. Moen | 2018 | Nurse-Led Diabetes Self-Management Education Improves Clinical Parameters in Ethiopia | Subject |
|  | A. Singh and M. Nichols | 2020 | Nurse-Led Education and Engagement for Diabetes Care in Sub-Saharan Africa: Protocol for a Mixed Methods Study | Subject |
|  | D. C Diriba, D. Y. P. Leung and L. K. P. Suen | 2021 | A nurse-led, community-based self management program for people living with type 2 diabetes in Western Ethiopia: A feasibility and pilot study protocol | Subject |
|  | C. D. D, D. Y. P. Leung and L. K. P. Suen | 2021 | A nurse-led, community-based self management program for people living with type 2 diabetes in Western Ethiopia: A feasibility and pilot study protocol | Subject |
|  | D. Diriba, D. Y. P. Leung and L. K. P. Suen | 2021 | A nurse‐led, community‐based self management program for people living with type 2 diabetes in Western Ethiopia: A feasibility and pilot study protocol | Subject |
|  | P. DeCola, D. Benton, C. Peterson and D. Matebeni | 2012 | Nurses' potential to lead in non-communicable disease global crisis | Subject |
|  | T. Ilori and R. A. Sanusi | 2022 | Nutrition-related knowledge, practice, and weight status of patients with chronic diseases attending a district hospital in Nigeria | Subject |
|  | T, Illori and R.A. Sanusi | 2022 | Nutrition-related knowledge, practice, and weight status of patients with chronic diseases attending a district hospital in Nigeria | Repetition |
|  | K. Opoku-Addai, K. A. Korsah and G. P. Mensah | 2022 | Nutritional self-care practices and skills of patients with diabetes mellitus: A study at a tertiary hospital in Ghana | Subject |
|  | Y. Wang, A. Yin, T. Bian, X. Zhao, S. Zheng et al. | 2021 | Observation of efficacy of Internet-based chronic disease management model combined with modified therapy of Bushenyiliu decoction in treating patients with type 2 diabetes mellitus and prostate cancer and its effect on disease control rate | Subject |
|  | https://www.hindawi.com/journals/ecam/2021/7767186/ | 2021 | Observation of efficacy of Internet-based chronic disease management model combined with modified therapy of Bushenyiliu decoction in treating patients with type 2 diabetes mellitus and prostate cancer and its effect on disease control rate | Subject |
|  | Y. Hu, N. Wang, P. Chen, M. Fan and H. Han | 2010 | Observation of nursing treatment effects for community diabetes | Subject |
|  | M. Thompson | 2014 | Occupations, habits, and routines: perspectives from persons with diabetes | Subject |
|  | A. N. Odili, L. Thijs, W.-Y. Yang, J. O. Ogedengbe, M. M. Nwegbu et al. | 2017 | Office and Home Blood Pressures as Determinants of Electrocardiographic Left Ventricular Hypertrophy Among Black Nigerians Compared With White Flemish | Subject |
|  | N. Coley, A. Rosenberg, T. van Middelaar, A. Soulier, M. Barbera et al. | 2019 | Older Adults' Reasons for Participating in an eHealth Prevention Trial: A Cross-Country, Mixed-Methods Comparison | Subject |
|  | G. McHugh, A. Koris, V. Simms, T. Bandason, L. Sigwadhi et al. | 2023 | On Campus HIV Self-Testing Distribution at Tertiary Level Colleges in Zimbabwe Increases Access to HIV Testing for Youth | Subject |
|  | A. Sekoni, W. Tun, O. Dirisu, T. Ladi-Akinyemi, E. Shoyemi, S. Adebajo et al. | 2022 | Operationalizing the distribution of oral HIV self-testing kits to men who have sex with men (MSM) in a highly homophobic environment: the Nigerian experience | Subject |
|  | Sekoni, A., Tun, W., Dirisu, O. et al | 2022 | Operationalizing the distribution of oral HIV self-testing kits to men who have sex with men (MSM) in a highly homophobic environment: the Nigerian experience | Subject |
|  | X. Jiang, Y. Wang, A. Xiao and S. Feng | 2016 | Opinions, Attitudes, and Beliefs About Self-Treatment Practices in a Nigerian urban Setting: Implications for Health Education | Subject |
|  | X. Jiang, Y. Wang, A. Xiao and S. Feng | 2019 | Opportunities and challenges to integrating mental health into HIV programs in a low- and middle-income country: insights from the Nigeria implementation science Alliance | Subject |
|  | B. O. Bashiru and I. N. Anthony | 2014 | Oral self-care practices among university students in Port Harcourt, Rivers State | Subject |
|  | H. Ruokonen, K. Nylund, J. H. Meurman, A. M. Heikkinen, J. Furuholm et al. | 2019 | Oral symptoms and oral health-related quality of life in patients with chronic kidney disease from predialysis to posttransplantation | Subject |
|  | M. Solano, E. Daguindau, C. Faure, P. Loriod, C. Pain et al. | 2021 | Oral therapy adherence and satisfaction in patients with multiple myeloma | Subject |
|  | R. A. Adewole, F. I. Ojini, J. A. Akinwande and M. A. Danesi | 2011 | Oro-dental and maxillofacial trauma in epilepsy at a tertiary hospital in Lagos | Subject |
|  | B. J. Beard | 2005 | Orphan care in Malawi: current practices | Subject |
|  | F. J. Afolabi, P. d. Beer and J. A. Haafkens | 2020 | Orthodox or traditional medicine? Private or public healthcare? Exploring treatment pathways for occupational health problems among informal automobile artisans | Subject |
|  | F. J. Afolabi, P. De Beer and J. A. Haafkens | 2020 | Orthodox or traditional medicine? Private or public healthcare? Exploring treatment pathways for occupational health problems among informal automobile artisans | Repetition |
|  | F. J. Afolabi, P. De Beer and J. A. Haafkens | 2020 | Orthodox or traditional medicine? Private or public healthcare? Exploring treatment pathways for occupational health problems among informal automobile artisans | Repetition |
|  | A. K. Rana, S. Rohit, V. K. Sharma, M. Ashish and S. Rachana | 2020 | Otorhinolaryngological myiasis: the problem and its presentations in the weak and forgotten | Subject |
|  | Rana AK, Sharma R, Sharma VK, Mehrotra A, Singh R.  Et al. | 2020 | Otorhinolaryngological myiasis: the problem and its presentations in the weak and forgotten | Subject |
|  | A. K. Rana, R. Sharma, V. K. Sharma, A. Mehrotra and R. Singh | 2020 | Otorhinolaryngological myiasis: the problem and its presentations in the weak and forgotten | Subject |
|  | D. Maher and A. D. Harries | 1996 | An out-patient audit of the insulin administration technique of patients with diabetes mellitus in Blantyre, Malawi | Subject |
|  | B. Smide | 2009 | Outcome of foot examinations in Tanzanian and Swedish diabetic patients, a comparative study | Subject |
|  | M. K. Mashola, E. Korkie and D. J. Mothabeng | 2021 | Pain and its impact on functioning and disability in manual wheelchair users with spinal cord injury: a protocol for a mixed-methods study | Subject |
|  | L. Jibb, P. C. Nathan, V. Breakey, C. Fernandez, D. Johnston, V. Lewis et al. | 2020 | Pain Squad+ smartphone app to support real-time pain treatment for adolescents with cancer: protocol for a randomised controlled trial | Subject |
|  | M. Skovdal, R. Maswera, N. Kadzura, C. Nyamukapa, R. Rhead et al. | 2020 | Parental obligations, care and HIV treatment: How care for others motivates self-care in Zimbabwe | Subject |
|  | E. Coyne, J. Carlini, T. Doherty, W. Harlow, M. L. Mitchell and L. Grealish | 2020 | Partnership between Nurse Navigators and adult persons living with complex chronic disease-An exploratory study | Subject |
|  | P. Shih, H. Worth, J. Travaglia and A. Kelly-Hanku | 2017 | Pastoral power in HIV prevention: converging rationalities of care in Christian and medical practices in Papua New Guinea | Subject |
|  | https://www.sciencedirect.com/science/journal/02779536 | 2021 |  | Invalid |
|  | M. Ploughman, M. W. Austin, M. Murdoch, A. Kearney, M. Godwin and M. Stefanelli | 2012 | The path to self-management: a qualitative study involving older people with multiple sclerosis | Subject |
|  | K. E. Manera, D. W. Johnson, J. C. Craig, J. I. Shen, L. Ruiz, A. Y.-M. Wang et al. | 2019 | Patient and Caregiver Priorities for Outcomes in Peritoneal Dialysis: Multinational Nominal Group Technique Study | Subject |
|  | J.-A. Walkden, P. J. McCullagh and W. G. Kernohan | 2019 | Patient and carer survey of remote vital sign telemonitoring for self-management of long-term conditions | Subject |
|  | A. Marr, A. Tsampalieros, J. Courtney, J. Seid Hamid, J. St-Denis-Murphy et al. | 2022 | Patient and family perspectives of a Pre-Transition Visit in a paediatric tertiary care diabetes clinic | Subject |
|  | E. Venables, Z. Ndlovu, D. Munyaradzi, G. Martínez-Pérez, E. Mbofana et al. | 2019 | Patient and health-care worker experiences of an HIV Viral Load intervention using SMS: a qualitative study | Subject |
|  | https://journals.plos.org/plosone/article?id=10.1371/journal.pone.0215236 | 2019 | Patient and health-care worker experiences of an HIV Viral Load intervention using SMS: a qualitative study | Subject |
|  | J. Aweko, J. De Man, P. Absetz, C.-G. Östenson, S. Swartling Peterson et al. | 2018 | Patient and Provider Dilemmas of Type 2 Diabetes Self-Management: A Qualitative Study in Socioeconomically Disadvantaged Communities in Stockholm | Subject |
|  | L. Liu, Y. Li, K. Sha, Y. Wang and X. He | 2013 | Patient assessment of chronic illness care, glycemic control and the utilization of community health care among the patients with type 2 diabetes in Shanghai, China | Subject |
|  | Liu LJ, Li Y, Sha K, Wang Y, He X. | 2013 | Patient assessment of chronic illness care, glycemic control and the utilization of community health care among the patients with type 2 diabetes in Shanghai, China | Subject |
|  | M. Acuña Mora, K. Luyckx, C. Sparud-Lundin, M. Peeters, A. van Staa et al. | 2018 | Patient empowerment in young persons with chronic conditions: Psychometric properties of the Gothenburg Young Persons Empowerment Scale (GYPES) | Review |
|  | R. C. Walker, A. Tong, K. Howard and S. C. Palmer | 2019 | Patient expectations and experiences of remote monitoring for chronic diseases: Systematic review and thematic synthesis of qualitative studies | review |
|  | E. Kim, P. K. Ndege, E. Jackson, D. J. Clauw and V. L. Ellingrod | 2019 | Patient perspectives on medication self-management in rural Kenya: a cross-sectional survey | Methods |
|  | J. Kerklaan, E. Hannan, A. Baumgart, K. E. Manera, A. Ju et al. | 2020 | Patient- and parent proxy-reported outcome measures for life participation in children with chronic kidney disease: a systematic review | Subject |
|  | L. Fisher, C. A. Chesla, K. M. Chun, M. M. Skaff, J. T. Mullan et al. | 2013 | Patient-appraised couple emotion management and disease management among Chinese American patients with type 2 diabetes | subject |
|  | D. B. Bekelman, M. E. Plomondon, M. D. Sullivan, K. Nelson, B. Hattler et al. | 2013 | Patient-centered disease management (PCDM) for heart failure: study protocol for a randomised controlled trial | Subject |
|  | A. A. Ahonkhai, I. Onwuatuelo, S. Regan, A. Adegoke, E. Losina et al. | 2017 | The patient-centered medical home: a reality for HIV care in Nigeria | Subject |
|  | https://academic.oup.com/intqhc/article-abstract/29/5/654/4079829 | 2017 | The patient-centered medical home: a reality for HIV care in Nigeria | Repetition |
|  | S. O. Gbadamosi, C. Eze, J. O. Olawepo, J. Iwelunmor, D. F. Sarpong et al. | 2018 | A Patient-Held Smartcard With a Unique Identifier and an mHealth Platform to Improve the Availability of Prenatal Test Results in Rural Nigeria: Demonstration Study | Subject |
|  | M. M. Duwell, A. R. Knowlton, J. B. Nachega, A. Efron, R. Goliath et al. | 2013 | Patient-Nominated, Community-Based HIV Treatment Supporters: Patient Perspectives, Feasibility, Challenges, and Factors for Success in HIV-Infected South African Adults | Subject |
|  | V. Angwenyi, C. Aantjes, J. Bunders-Aelen, J. V. Lazarus and B. Criel | 2019 | Patient-provider perspectives on self-management support and patient empowerment in chronic care: A mixed-methods study in a rural sub-Saharan setting | Subject |
|  | V. Angwenyi, C. Aantjes, J. Bunders‐Aelen, J. V. Lazarus and B. Criel | 2019 | Patient–provider perspectives on self‐management support and patient empowerment in chronic care: A mixed‐methods study in a rural sub‐Saharan setting | Repetition |
|  | V. Angwenyi, C. Aantjes, M. Kajumi, J. De Man, B. Criel and J. Bunders-Aelen | 2018 | Patients experiences of self-management and strategies for dealing with chronic conditions in rural Malawi | Included |
|  | V. Angwenyi, C. Aantjes, M. Kajumi, J. d. Man, B. Criel and J. Bunders-Aelen | 2018 | Patients experiences of self-management and strategies for dealing with chronic conditions in rural Malawi | Repetition |
|  | https://journals.plos.org/plosone/article?id=10.1371/journal.pone.0199977 | 2018 | Patients experiences of self-management and strategies for dealing with chronic conditions in rural Malawi | Repetition |
|  | M. Hirjaba, A. Häggman-Laitila, A.-M. Pietilä and M. Kangasniemi | 2015 | Patients have unwritten duties: experiences of patients with type 1 diabetes in health care | Methods |
|  | X. Jiang, Y. Wang, A. Xiao and S. Feng | 2021 | Patients undergoing assisted peritoneal dialysis to show a better technique survival: a competing risk analysis | Subject |
|  | https://onlinelibrary.wiley.com/doi/10.1111/ijcp.14192 | 2021 | Patients undergoing assisted peritoneal dialysis to show a better technique survival: a competing risk analysis | Repetition |
|  | A. Nakiwogga-Muwanga, J. Musaazi, E. Katabira, W. Worodria, S. A. Talisuna and R. Colebunders | 2015 | Patients who return to care after tracking remain at high risk of attrition: experience from a large HIV clinic, Uganda | Subject |
|  | https://std.sagepub.com/content/26/1/42.full | 2015 | Patients who return to care after tracking remain at high risk of attrition: experience from a large HIV clinic, Uganda | Subject |
|  | A. Mogueo and B. K. Defo | 2022 | Patients' and family caregivers' experiences and perceptions about factors hampering or facilitating patient empowerment for self-management of hypertension and diabetes in Cameroon | Population |
|  | H. N. Shilubane and E. Potgieter | 2007 | Patients' and family members' knowledge and views regarding diabetes mellitus and its treatment | Population |
|  | H. Shilubane, L. Netshikweta and T. Ralineba | 2016 | Beliefs and practices of diabetic patients in Vhembe district of Limpopo Province | Methods |
|  | J. J. Gagliardino, P. Aschner, S. H. Baik, J. Chan, J. M. Chantelot, et al. | 2012 | Patients' education, and its impact on care outcomes, resource consumption and working conditions: data from the International Diabetes Management Practices Study (IDMPS) | Subject |
|  | E. N. Bosire | 2021 | Patients' Experiences of Comorbid HIV/AIDS and Diabetes Care and Management in Soweto, South Africa | Included |
|  | I. F. Bossman, S. Dare, B. A. Oduro, P. K. Baffour, T. K. Hinneh and J. E. Nally | 2021 | Patients' knowledge of diabetes foot complications and self-management practices in Ghana: A phenomenological study | Population |
|  | T. Al-Rousan, M. A. Pesantes, S. Dadabhai, N. R. Kandula, M. D. Huffman et al. | 2020 | Patients' perceptions of self-management of high blood pressure in three low- and middle-income countries: findings from the BPMONITOR study | Subject |
|  | N. Campo Guinea and M. C. Portillo | 2013 | [Patients' self-management of type 2 diabetes: a narrative review] | Review |
|  | A. C. Enikuomehin, M. A. Olamoyegun, O. A. Ojo, G. D. Ajani, T. A. Akinlade and O. A. Ala | 2021 | Pattern of Self-care practices among type 2 diabetes patients in Southwest, Nigeria | Methods |
|  | X. Zhang, W. Zhou and B. Jiang | 2022 | Patterns of living lost? Measuring community participation and other influences on the health of older migrants in China | Subject |
|  | https://www.mdpi.com/1660-4601/19/8/4542 | 2022 | Patterns of living lost? Measuring community participation and other influences on the health of older migrants in China | Repetition |
|  | S. Okoboi, O. Lazarus, B. Castelnuovo, M. Nanfuka, A. Kambugu, A. Mujugira and R. King | 2020 | Peer distribution of HIV self-test kits to men who have sex with men to identify undiagnosed HIV infection in Uganda: A pilot study | Subject |
|  | H. Otanga, B. Semujju, L. Mwaniki and J. Aungo | 2022 | Peer support and social networking interventions in diabetes self-management in Kenya and Uganda: A scoping review | Methods |
|  | H. M. Lauckner and S. L. Hutchinson | 2016 | Peer support for people with chronic conditions in rural areas: a scoping review | Methods |
|  | E. B. Fisher, R. e. I. Boothroyd, M. M. Coufal, L. C. Baumann, J. C. Mbanya et al. | 2012 | Peer Support for Self-Management of Diabetes Improved Outcomes In International Settings | Methods |
|  | E. B. Fisher, R. I. Boothroyd, M. M. Coufal, L. C. Baumann, J. C. Mbanya et al. | 2012 | Peer support for self-management of diabetes improved outcomes in international settings | Repetition |
|  | EB Fisher et al. | 2012 | Peer support for self-management of diabetes improved outcomes in international settings | Repetition |
|  | E. B. Fisher, R. I. Boothroyd, M. M. Coufal, L. C. Baumann, J. C. Mbanya et al. | 2012 | Peer support for self-management of diabetes improved outcomes in international settings | Repetition |
|  | S. H. J. du Toit, Y. L. Chan, G. M. Jessup and J. Weaver | 2020 | Peer-enabled staff training in residential care settings as means for promoting person-centred dementia care | Subject |
|  | L. Chen, X. Zhou, X. Tu, H. Cheng, Z. Duan, G. Lu and Y. Yuan | 2022 | People's perceptions and experience of managing life after recurrent pancreatitis: a qualitative study in eastern China | Subject |
|  | F. Shaba, O. A. Offorjebe, K. Phiri, E. Lungu, P. Kalande a at. Al. | 2019 | Perceived Acceptability of a Facility-Based HIV Self-Test Intervention in Outpatient Waiting Spaces Among Adult Outpatients in Malawi: A Formative Study | Subject |
|  | G. Mphwanthe, M. Carolan, D. Earnesty and L. Weatherspoon | 2021 | Perceived barriers and facilitators to diet and physical activity among adults diagnosed with type 2 diabetes in Malawi | Included |
|  | U. S. Dankoly, D. Vissers, S. B. El Mostafa, A. Ziyyat, B. Van Rompaey et al. | 2016 | Perceived barriers, benefits, facilitators, and attitudes of health professionals towards type 2 diabetes management in Oujda, Morocco: a qualitative focus group study | Subject |
|  | L. Holmgren and O. Söderhamn | 2005 | Perceived health and well-being in a group of physically active older Swedish people | Subject |
|  | F. Haffejee, B. Maughan-Brown, T. Buthelezi and A. B. M. Kharsany | 2018 | Perceived HIV-related stigma among university students in South Africa: implications for HIV testing | Subject |
|  | K. F. Ortblad, M. M. Chanda, M. Mwale, J. E. Haberer, M. McConnell, C. E. Oldenburg and T. Bärnighausen | 2020 | Perceived Knowledge of HIV-Negative Status Increases Condom Use Among Female Sex Workers in Zambian Transit Towns | Subject |
|  | P. Coppo, L. Pisani and A. Keita | 1992 | Perceived morbidity and health behaviour in a Dogon community | Subject |
|  | A. S. Bhagavathula, E. A. Gebreyohannes, T. M. Abegaz and T. B. Abebe | 2018 | Perceived Obstacles Faced by Diabetes Patients Attending University of Gondar Hospital, Northwest Ethiopia | Subject |
|  | P. N. Mbeje and N. Mtshali | 2021 | Perceived predictors of quality of life in patients with end-stage renal disease on dialysis | Methods |
|  | F. Babafunke, O. Olajumoke, L. Oluwatofunmi, A. Oluyemi, A. Oluseyi et al. | 2022 | Perceived risk and protective behaviors related to COVID-19 among the Nigerian general population: an application of the extended parallel process model | Methods |
|  | https://www.sciencedirect.com/science/article/pii/S2468227622001661 | 2022 | Perceived risk and protective behaviors related to COVID-19 among the Nigerian general population: an application of the extended parallel process model | Repetition |
|  | A. Oluma, M. Abadiga, G. Mosisa, G. Fekadu and E. Turi | 2020 | Perceived Self-Efficacy and Associated Factors Among Adult Patients with Type 2 Diabetes Mellitus at Public Hospitals of Western Ethiopia, 2020 | Subject |
|  | S. Kaai, S. Bullock, A. Sarna, M. Chersich, S. Luchters et al. | 2010 | Perceived stigma among patients receiving antiretroviral treatment: a prospective randomised trial comparing an m-DOT strategy with standard-of-care in Kenya | Subject |
|  | A. Ajose, O. O. Odukoya and B. Akodu | 2022 | Perceived stress, depression and medication adherence among hypertensive patients attending secondary care hospital in Lagos, Nigeria. | Subject |
|  | Q. Li, E. Mpofu, C. Yin and K. W. Turner | 2019 | Perception of Falls and Confidence in Self-Management of Falls among Older Adults | Subject |
|  | J. Tseng, L. Halperin, M. D. Ritholz and W. C. Hsu | 2013 | Perceptions and management of psychosocial factors affecting type 2 diabetes mellitus in Chinese Americans | Population |
|  | F. Saleem, M. A. Hassali, A. A. Shafie, S. Bashir and M. Atif | 2011 | Perceptions of disease state management among Pakistani hypertensive patients: findings from a focus group discussion | Population |
|  | M. Mutumba, V. Musiime, H. Mugerwa, H. Nakyambadde, A. Gautam et al. | 2019 | Perceptions of HIV Self-Management Roles and Challenges in Adolescents, Caregivers, and Health Care Providers | Population |
|  | C. N. Nyoni and M. Reid | 2019 | Perceptions of patients regarding diabetes-related health communication strategies in the Free State, South Africa | Subject |
|  | M. Mutumba, H. Mugerwa, V. Musiime, A. Gautam, H. Nakyambadde, C. Matama and R. Stephenson | 2019 | Perceptions of strategies and intervention approaches for HIV self-management among Ugandan adolescents: a qualitative study | Subject |
|  | https://journals.sagepub.com/doi/full/10.1177/2325958218823246 | 2019 | Perceptions of strategies and intervention approaches for HIV self-management among Ugandan adolescents: a qualitative study | Repetition |
|  | M. Mwangome, E. Geubbels, P. Klatser and M. Dieleman | 2017 | Perceptions on diabetes care provision among health providers in rural Tanzania: a qualitative study | Subject |
|  | P. Bwana, L. Ochieng and M. Mwau | 2018 | Performance and usability evaluation of the INSTI HIV self-test in Kenya for qualitative detection of antibodies to HIV | Subject |
|  | J. M. Burrin and C. P. Price | 1984 | Performance of three enzymic methods for filter paper glucose determination | Subject |
|  | T. Elliott, R. E. Kohler, B. Monare, N. Moshashane, K. Ramontshonyana et al. | 2019 | Performance of vaginal self-sampling for human papillomavirus testing among women living with HIV in Botswana | Subject |
|  | P. Aschner, J. J. Gagliardino, H. Ilkova, F. Lavalle, A. Ramachandran et al. | 2020 | Persistent poor glycaemic control in individuals with type 2 diabetes in developing countries: 12 years of real-world evidence of the International Diabetes Management Practices Study (IDMPS) | Subject |
|  | S. Horter, A. Daftary, T. Keam, S. Bernays, K. Bhanushali et al. | 2021 | Person-centred care in TB | Subject |
|  | T. R. Mathuniwa-Dlamini, F. A. Gary, H. A. Yarandi and M. D. Mathunjwa | 2011 | Personal Characteristics and Health Status among Southern Rural African-American Menopausal Women | Subject |
|  | T. R. Mathunjwa-Dlamini, F. A. Gary, H. A. Yarandi and M. D. Mathunjwa | 2011 | Personal characteristics and health status among southern rural African-American menopausal women | Subject |
|  | I. B. Molla, M. A. Berhie, K. A. Debele, G. N. Germossa and F. B. Hailu | 2023 | Persons with Diabetes' Perceptions of Family Burden and Associated Factors | Subject |
|  | C. Wilson and G. Twigg | 2018 | Pharmacist-led depression screening and intervention in an underserved, rural, and multi-ethnic diabetic population | Subject |
|  | S. I. Bello, K. A. Ganiyu, Y. O. Dakop and P. O. Erah | 2012 | Pharmacist's intervention in the control of blood sugar levels in randomised diabetes patients at a primary health care setting in Benin City | Subject |
|  | T. Greacen, A. Simon, A. Troisoeufs and K. Champenois | 2020 | [Pharmacists’ and key populations’ points of view on HIV self-test dispensation in pharmacies in France] | Subject |
|  | R. Z. Sangeda, F. Mosha, M. Prosperi, S. Aboud, J. Vercauteren et al. | 2014 | Pharmacy refill adherence outperforms self-reported methods in predicting HIV therapy outcome in resource-limited settings | Subject |
|  | M. Abboah-Offei, K. Bristowe, N. A. Vanderpuye-Donton, G. Ansa, Y. D. Oppong-Agyei et al. | 2020 | Phase II mixed methods' feasibility cluster randomised controlled trial of a novel community-based enhanced care intervention to improve person-centred outcomes for people living with HIV in Ghana | Subject |
|  | B. Ovbiagele | 2015 | Phone-based intervention under nurse guidance after stroke: concept for lowering blood pressure after stroke in Sub-Saharan Africa | Subject |
|  | D. Vancampfort, B. Stubbs and J. Mugisha | 2018 | Physical activity and HIV in sub-Saharan Africa: a systematic review of correlates and levels | Subject |
|  | Z. Zhu, W. Yan, X. Wang, D. Hu, Y. Zhu and J. Chen | 2021 | Physical activity, blood pressure control, and health-related quality of life among hypertensive individuals: a cross-sectional study in Jiangsu Province, China | Subject |
|  | https://journals.sagepub.com/doi/full/10.1177/10105395211014650 | 2021 | Physical activity, blood pressure control, and health-related quality of life among hypertensive individuals: a cross-sectional study in Jiangsu Province, China | Subject |
|  | E. Potter, F. Burstein, D. Flynn, I. D. Hwang, T. Dinh et al. | 2022 | Physician-Authored Feedback in a Type 2 Diabetes Self-management App: Acceptability Study | Subject |
|  | S. Cobbing, J. Hanass-Hancock and M. Deane | 2014 | Physiotherapy rehabilitation in the context of HIV and disability in KwaZulu-Natal, South Africa | Subject |
|  | V. Omollo, E. A. Bukusi, L. Kidoguchi, F. Mogaka, J. B. Odoyo et al. | 2021 | A Pilot Evaluation of Expedited Partner Treatment and Partner Human Immunodeficiency Virus Self-Testing Among Adolescent Girls and Young Women Diagnosed With Chlamydia trachomatis and Neisseria gonorrhoeae in Kisumu, Kenya | Subject |
|  | M. L. Wieland, J. W. Njeru, M. M. Hanza, D. H. Boehm, D. Singh et al. | 2017 | Pilot Feasibility Study of a Digital Storytelling Intervention for Immigrant and Refugee Adults With Diabetes | Subject |
|  | E. Asante, V. Bam, A. K. Diji, A. Y. Lomotey, A. Owusu Boateng, O. Sarfo-Kantanka et al. | 2020 | Pilot Mobile Phone Intervention in Promoting Type 2 Diabetes Management in an Urban Area in Ghana: A Randomized Controlled Trial | Subject |
|  | E. Asante, V. Bam, A. K.-A. Diji, A. Y. Lomotey, A. Owusu Boateng et al. | 2020 | Pilot Mobile Phone Intervention in Promoting Type 2 Diabetes Management in an Urban Area in Ghana: A Randomized Controlled Trial | Subject |
|  | M. Odenwald, B. Lingenfelder, W. Peschel, F. A. Haibe, A. M. Warsame et al. | 2012 | A pilot study on community-based outpatient treatment for patients with chronic psychotic disorders in Somalia: Change in symptoms, functioning and co-morbid khat use | Subject |
|  | K. J. Train and N. Butler | 2013 | A pilot study to test psychophonetics methodology for self-care and empathy in compassion fatigue, burnout and secondary traumatic stress | Subject |
|  | R. J. Mash and J. Cairncross | 2023 | Piloting of virtual group education for diabetes in Cape Town: An exploratory qualitative study | Subject |
|  | W. R. Brieger and E. Edozien | 1983 | Pioneering patient education in Nigeria | Subject |
|  | E. Buch, L. M. Irwig, K. R. L. Huddle, L. P. Krige, L. H. Krut and J. M. Kuyl | 1983 | Pointers to preventing hyperglycaemic emergencies in Soweto | Subject |
|  | A. E. Joham, R. J. Norman, E. Stener-Victorin, R. S. Legro, S. Franks et al. | 2022 | Polycystic ovary syndrome | Subject |
|  | M. Bouaoudate, J. El-Harti, R. Abouqal, K. Abidi and A. A. Zeggwagh | 2022 | Polypharmacy-related polypathology in the elderly: a case study at Ibn Sina Hospital, Morocco | Subject |
|  | https://www.tjpr.org/admin/12389900798187/2022_21_10_17.pdf | 2022 | Polypharmacy-related polypathology in the elderly: a case study at Ibn Sina Hospital, Morocco | Subject |
|  | M. S. Erkocho, D. T. Adugna, T. T. Arficho and A. G. Azene | 2022 | Poor dietary practice and associated factors among type-2 diabetes mellitus patients on follow-up in Nigist Eleni Mohammed Memorial Teaching Hospital, Ethiopia | Methods |
|  | D. Havlir, M. Kamya, M. Petersen, Maya | 2024 | Integrated HIV, diabetes, and hypertension care in sub-Saharan Africa. | Subject |
|  | E. Mulugeta Selassie, A. Dessalegn Tamiru, A. Tegegn Tadesse and A. Adisalem Gizachew | 2022 | Poor dietary practice and associated factors among type-2 diabetes mellitus patients on follow-up in Nigist Eleni Mohammed Memorial Teaching Hospital, Ethiopia | Repetition |
|  | https://www.panafrican-med-journal.com/content/article/41/164/full/ | 2022 | Poor dietary practice and associated factors among type-2 diabetes mellitus patients on follow-up in Nigist Eleni Mohammed Memorial Teaching Hospital, Ethiopia | Repetition |
|  | A. Camara, N. M. Baldé, J. Sobngwi-Tambekou, A. P. Kengne, M. M. Diallo et al. | 2015 | Poor glycemic control in type 2 diabetes in the South of the Sahara: the issue of limited access to an HbA1c test | subject |
|  | S. Letta, F. Aga, T. A. Yadeta, B. Geda and Y. Dessie | 2022 | Poor Self-Care Practices and Being Urban Resident Strongly Predict Chronic Complications Among Patients with Type 2 Diabetes in Eastern Ethiopia: A Hospital-Based Cross-Sectional Study | subject |
|  | M. M. Kebede and C. R. Pischke | 2019 | Popular Diabetes Apps and the Impact of Diabetes App Use on Self-Care Behaviour: A Survey Among the Digital Community of Persons With Diabetes on Social Media | subject |
|  | A. R. Basengere, B. A. Birindwa, C. Baharanyi, B. Patrick, T. Kashosi et al. | 2022 | Population behavior and practices during the first wave of COVID-19 pandemic in low resource-conditions of south Kivu, east of Democratic Republic of Congo | subject |
|  | https://www.jepublichealth.com/index.php?journal=jepublichealth&page=article&op=view&path%5B%5D=429 | 2022 | Population behavior and practices during the first wave of COVID-19 pandemic in low resource-conditions of south Kivu, east of Democratic Republic of Congo | subject |
|  | T. Hartley, M. Burger and G. Inglis-Jasiem | 2022 | Post stroke health-related quality of life, stroke severity and function: a longitudinal cohort study | Subject |
|  | https://ajod.org/index.php/ajod/article/view/947 | 2022 | Post stroke health-related quality of life, stroke severity and function: a longitudinal cohort study | Repetition |
|  | T. Hartley, M. Burger and G. Inglis-Jassiem | 2022 | Post stroke health-related quality of life, stroke severity and function: A longitudinal cohort study | Repetition |
|  | M. J. Lyonnais, P. Bray, A. P. Rafferty, N. L. Winterbauer and R. A. Bell | 2020 | Potential Effect of Vouchers on the Completion of Self-management Education Among Patients With Diabetes | Subject |
|  | J. Pons, W. E. Mapham, B. Newsome, L. Myer, R. Anderson et al. | 2012 | The potential impact of a cataract surgery programme on the care of orphans and vulnerable children in Swaziland | Subject |
|  | C. N. Onyishi, C. Eseadi, L. C. Ilechukwu, K. N. Okoro, C. N. Okolie et al. | 2022 | Potential influences of religiosity and religious coping strategies on people with diabetes | Subject |
|  | T. J. Copeland | 2011 | Poverty, nutrition, and a cultural model of managing HIV/AIDS among women in Nairobi, Kenya | Subject |
|  | https://onlinelibrary.wiley.com/doi/10.1111/j.2153-9588.2011.01068.x/full | 2022 |  | Invalid |
|  | Y. L. Niriayo, S. Ibrahim, T. D. Kassa, S. W. Asgedom, T. M. Atey, K. Gidey, G. T. Demoz and D. Kahsay | 2019 | Practice and predictors of self-care behaviors among ambulatory patients with hypertension in Ethiopia | Method |
|  | N. Yirga Legesse, I. Seid, K. Tesfaye Dessale, A. Solomon Weldegebreal, A. Tesfay Mahari et al. | 2019 | Practice and predictors of self-care behaviors among ambulatory patients with hypertension in Ethiopia | Repetition |
|  | O. Ekúndayò, D. Tataw and M. Qobadi | 2015 | Predictors and contextual factors in prostate cancer screening service utilization among African-American men in an urban sample in Mississippi, USA | Method |
|  | https://www.tandfonline.com/loi/rhpe20 | 2015 |  | Invalid |
|  | A. Oluma, G. Mosisa, M. Abadiga, R. Tsegaye, A. Habte and E. Abdissa | 2020 | Predictors of Adherence to Self-Care Behavior Among Patients with Diabetes at Public Hospitals in West Ethiopia | Method |
|  | G. Degefa, K. Wubshet, S. Tesfaye and A. T. Hirigo | 2020 | Predictors of Adherence Toward Specific Domains of Diabetic Self-Care Among Type-2 Diabetes Patients | Method |
|  | P. M. Amegbor, V. Z. Kuuire, H. Robertson and O. A. Kuffuor | 2018 | Predictors of basic self-care and intermediate self-care functional disabilities among older adults in Ghana | Method |
|  | T. M. Gulentie, E. M. Yesuf, T. S. Yazie and B. Kefale | 2020 | Predictors of Diabetes Self-Care Practice Among Patients with Type 2 Diabetes in Public Hospitals in Northeastern Ethiopia: A Facility-Based Cross-Sectional Study | Method |
|  | J. K. Muthoka and H. d. Toit | 2016 | Predictors of HIV self-testing among health workers at Nyeri Provincial Hospital in Kenya | Method |
|  | B. R. Yanos, K. K. Saules, L. M. Schuh and S. Sogg | 2015 | Predictors of Lowest Weight and Long-Term Weight Regain Among Roux-en-Y Gastric Bypass Patients | Subject |
|  | G. M. Kassa, T. Abilo, G. Yalemzewod Assefa, A. Temesgen Tadesse, T. Adino Tesfahun et al. | 2020 | Predictors of mortality among multidrug-resistant tuberculosis patients in central Ethiopia: a retrospective follow-up study | Subject |
|  | G. M. Kassa, T. Abilo, G. Yalemzewod Assefa, A. Temesgen Tadesse, T. Adino Tesfahun et al. | 2020 | Predictors of mortality among multidrugresistant tuberculosis patients in central Ethiopia: a retrospective follow-up study | Repetition |
|  | G. M. Kassa, A. Tadesse, Y. A. Gelaw, T. T. Alemayehu, A. T. Tsegaye et al. | 2020 | Predictors of mortality among multidrug-resistant tuberculosis patients in central Ethiopia: a retrospective follow-up study | Repetition |
|  | M. G. Dedefo, S. K. Abate, B. M. Ejeta and A. T. Korsa | 2020 | Predictors of poor glycemic control and level of glycemic control among diabetic patients in west Ethiopia | Method |
|  | A. R. Kamuhabwa and E. Charles | 2014 | Predictors of poor glycemic control in type 2 diabetic patients attending public hospitals in Dar es Salaam | Method |
|  | B. H. Ayele, M. M. Mengesha and T. Tesfa | 2019 | Predictors of self-care activities of outpatient diabetic residents in Harar and Dire Dawa: A hospital-based cross-sectional study | Method |
|  | D. Dereje Chala, B. Tariku Tesfaye and B. Firew Tekle | 2020 | Predictors of self-management practices among diabetic patients attending hospitals in western Oromia, Ethiopia | Method |
|  | D. C. Diriba, T. T. Bekuma and F. T. Bobo | 2020 | Predictors of self-management practices among diabetic patients attending hospitals in western Oromia, Ethiopia | Method |
|  | D. C. Diriba, T. T. Bekuma and F. T. Bobo | 2020 | Predictors of self-management practices among diabetic patients attending hospitals in western Oromia, Ethiopia | Repetition |
|  | J. Rujumba, J. Homsy, F. B. Mbazzi, Z. Namukwaya, A. Amone et al. | 2021 | Pregnant women, their male partners and health care providers' perceptions of HIV self-testing in Kampala, Uganda: Implications for integration in prevention of mother-to-child transmission programs and scale-up | Subject |
|  | R. Pelzer, E. H. Mathews and L. Liebenberg | 2011 | Preliminary application of a new bolus insulin model for type 1 diabetes | Subject |
|  | J. R. Falvey, M. J. Bade, C. Hogan, J. E. Forster and J. E. Stevens-Lapsley | 2020 | Preoperative Activities of Daily Living Dependency is Associated With Higher 30-Day Readmission Risk for Older Adults After Total Joint Arthroplasty | Subject |
|  | P. J. Bjertrup, N. Mmema, V. Dlamini, I. Ciglenecki, Q. Mpala et al. | 2021 | PrEP reminds me that I am the one to take responsibility of my life: a qualitative study exploring experiences of and attitudes towards pre-exposure prophylaxis use by women in Eswatini | Subject |
|  | X.-L. Liu, K. Willis, C.-J. J. Wu, P. Fulbrook, Y. Shi and M. Johnson | 2019 | Preparing Chinese patients with comorbid heart disease and diabetes for home management: a mixed methods study | Subject |
|  | R. Ansumana, K. H. Jacobsen, A. A. Gbakima, M. H. Hodges, J. M. Lamin et al. | 2013 | Presumptive self-diagnosis of malaria and other febrile illnesses in Sierra Leone | Subject |
|  | T. Tadesse, M. Belayneh, M. Diriba, F. Getahun, T. Ebisa and A. Abajobir | 2020 | Prevalence and associated factors of foot ulcer among diabetic patients in Ethiopia: a systematic review and meta-analysis | Protocol |
|  | https://rd.springer.com/article/10.1186/s12889-019-8133-y | 2020 | Prevalence and associated factors of foot ulcer among diabetic patients in Ethiopia: a systematic review and meta-analysis | Repetition |
|  | T. Tolossa, B. Mengist, D. Mulisa, G. Fetensa, E. Turi and A. Abajobir | 2020 | Prevalence and associated factors of foot ulcer among diabetic patients in Ethiopia: a systematic review and meta-analysis | Review |
|  | A. O. Ala, O. A. Ojo, C. A. Enikuomehin, G. O. Ajani, M. A. Olamoyegun et al. | 2020 | Prevalence and Determinants of Complementary and Alternative Medicine (CAM) Use among Diabetes Patients in Southwestern Nigeria | Subject |
|  | M. S. Salama, J. B. Isunju, S. K. David, F. Muneza, S. Ssemanda and N. M. Tumwesigye | 2021 | Prevalence and factors associated with alcohol consumption among persons with diabetes in Kampala, Uganda: a cross sectional study | Subject |
|  | Y. Fitaw and J. M. F. Boersma | 2006 | Prevalence and impact of disability in north-western Ethiopia | Subject |
|  | L. Busha Gamachu, A. Muktar Beshir, M. Ginenus Fekadu and D. Fekede Bekele | 2019 | Prevalence and predictors of self care practices among hypertensive patients at Jimma University Specialized Hospital, Southwest Ethiopia: cross-sectional study | Methods |
|  | L. Busha Gamachu, A. Muktar Beshir, M. Ginenus Fekadu and D. Fekede Bekele | 2017 | Prevalence and predictors of self care practices among hypertensive patients at Jimma University Specialized Hospital, Southwest Ethiopia: cross-sectional study | Repetition |
|  | E. Elayeh, A. Akour and R. N. Haddadin | 2021 | Prevalence and predictors of self-medication drugs to prevent or treat COVID-19: experience from a middle eastern country | Subject |
|  | <https://onlinelibrary.wiley.com/doi/10.1111/ijcp> | 2022 |  | Invalid |
|  | A. Al-Ansari and M. Nazir | 2020 | Prevalence of Dental Trauma and Receipt of Its Treatment among Male School Children in the Eastern Province of Saudi Arabia | Subject |
|  | D. G. McLarty, A. B. Swai, H. M. Kitange, G. Masuki, B. L. Mtinangi et al. | 1989 | Prevalence of diabetes and impaired glucose tolerance in rural Tanzania | Subject |
|  | T. G. Mariam, A. Alemayehu, E. Tesfaye, W. Mequannt, K. Temesgen et al. | 2017 | Prevalence of Diabetic Foot Ulcer and Associated Factors among Adult Diabetic Patients Who Attend the Diabetic Follow-Up Clinic at the University of Gondar Referral Hospital, North West Ethiopia, 2016: Institutional-Based Cross-Sectional Study | Subject |
|  | G. Alemu Mersha, Y. A. Alimaw and A. T. Woredekal | 2022 | Prevalence of diabetic retinopathy among diabetic patients in Northwest Ethiopia-A cross sectional hospital based study | Subject |
|  | T. Masupe, J. De Man, S. Onagbiye, T. Puoane and P. Delobelle | 2021 | Prevalence of disease complications and risk factor monitoring amongst diabetes and hypertension patients attending chronic disease management programmes in a South African Township | Subject |
|  | T. T. Habebo, E. J. Pooyan, A. M. Mosadeghrad, G. O. Babore and B. K. Dessu | 2020 | Prevalence of Poor Diabetes Self-Management Behaviors among Ethiopian Diabetes Mellitus Patients: A Systematic Review and Meta-Analysis | Review |
|  | V. Rajamanickam, K. Legesse, T. Girma, A. Bersisa and A. Tesfaye | 2017 | Prevalence of prediabetes and its risk factors among the employees of Ambo University, Oromia Region, Ethiopia | Subject |
|  | https://rmm.mazums.ac.ir/article-1-252-en.pdf | 2021 |  | Invalid |
|  | Elaine Kwan, Brian Draper, Zoltan H. Endre, Samuel B. Harvey, Mark A. Brown | 2021 | Prevalence, Risk Factors, and Cost Burden of Fall-Related Hospital Admissions in india | subject |
|  | E. Kwan, B. Draper, Z. H. Endre, S. B. Harvey and M. A. Brown | 2021 | Prevalence, types and recognition of cognitive impairment in dialysis patients in South Eastern Sydney | subject |
|  | W. Negash, T. Assefa, B. Sahiledengle, A. Tahir, Z. Regassa et al. | 2022 | Prevalences of diabetic foot ulcer and foot self-care practice, and associated factors in adult patients with diabetes in south-east Ethiopia | subject |
|  | B. H. Ayele, H. S. Roba, A. S. Beyene and M. M. Mengesha | 2020 | Prevalent, uncontrolled, and undiagnosed diabetes mellitus among urban adults in Dire Dawa, Eastern Ethiopia: A population-based cross-sectional study | Method |
|  | J. N. Suglo, K. Winkley and J. Sturt | 2020 | Prevention and Management of Diabetes-Related Foot Ulcers through Informal Caregiver Involvement: A Systematic Review | subject |
|  | E. G. Mekonen and T. Gebeyehu Demssie | 2022 | Preventive foot self-care practice and associated factors among diabetic patients attending the university of Gondar comprehensive specialized referral hospital, Northwest Ethiopia, 2021 | Method |
|  | C. J. van Zyl-Schalekamp | 1991 | Preventive self-care in three Free State communities | subject |
|  | F. Gu, H. Zhang and X. Li | 2010 | Primary exploration of tertiary disease-prevention application to the exercising therapy of the diabetes in the community | subject |
|  | E. Y. Oyewole, L. Y. Ojewale and O. O. Abimbola | 2022 | Primary Health Care Nurses' Competencies and Resources Availability for Diabetes Mellitus Care at Local Government Areas of Ibadan | subject |
|  | Z. K. Landu and T. Crowley | 2023 | Primary health care nurses' knowledge, self-efficacy and performance of diabetes self-management support | subject |
|  | D. B. Bekelman, M. E. Plomondon, E. P. Carey, M. D. Sullivan, K. M. Nelson, B. Hattler et al. | 2015 | Primary Results of the Patient-Centered Disease Management (PCDM) for Heart Failure Study: A Randomized Clinical Trial | subject |
|  | M. Rijken, R. Stüssgen, C. Leemrijse, M. J. L. Bogerd and J. C. Korevaar | 2021 | Priorities and preferences for care of people with multiple chronic conditions | subject |
|  | X. Lin, H. Xu and L. Ji | 2014 | Problems and nursing countermeasures of the insulin use of rural people with diabetes | subject |
|  | N. Leon, H. Namadingo, S. Cooper, K. Bobrow, C. Mwantisi et al. | 2021 | Process evaluation of a brief messaging intervention to improve diabetes treatment adherence in sub-Saharan Africa | subject |
|  | J. Van Olmen, M. Van Pelt, B. Malombo, G. M. Ku, D. Kanda et al. | 2017 | Process evaluation of a mobile health intervention for people with diabetes in low income countries - the implementation of the TEXT4DSM study | subject |
|  | J. Van Olmen, M. Van Pelt, B. Malombo, G. M. Ku, D. Kanda et al. | 2017 | Process evaluation of a mobile health intervention for people with diabetes in low income countries - the implementation of the TEXT4DSM study | Repetition |
|  | J. van Olmen, P. Absetz, R. W. Mayega, L. Timm, P. Delobelle et al. | 2022 | Process evaluation of a pragmatic implementation trial to support self-management for the prevention and management of type 2 diabetes in Uganda, South Africa and Sweden in the SMART2D project | subject |
|  | J. van Olmen, P. Absetz, R. W. Mayega, L. Timm, P. Delobelle et al. | 2022 | Process evaluation of a pragmatic implementation trial to support self-management for the prevention and management of type 2 diabetes in Uganda, South Africa and Sweden in the SMART2D project | subject |
|  | L. Lebina, O. Alaba, A. Ringane, K. Hlongwane, P. Pule et al. | 2019 | Process evaluation of implementation fidelity of the integrated chronic disease management model in two districts, South Africa | subject |
|  | https://link.springer.com/article/10.1186/s12913-019-4785-7 | 2018 | Process evaluation of implementation fidelity of the integrated chronic disease management model in two districts, South Africa | Repetition |
|  | R. B. Roaeid and A. A. Kablan | 2007 | Profile of diabetes health care at Benghazi Diabetes Centre, Libyan Arab Jamahiriya | subject |
|  | S. Kenya, C. N. Lebron, A. Y. H. Chang, H. Li, Y. A. Alonzo and O. Carrasquillo | 2015 | A profile of Latinos with poorly controlled diabetes in South Florida | subject |
|  | H. Thirumurthy, S. H. Masters, S. N. Mavedzenge, S. Maman, E. Omanga and K. Agot | 2016 | Promoting male partner HIV testing and safer sexual decision making through secondary distribution of self-tests by HIV-negative female sex workers and women receiving antenatal and post-partum care in Kenya: a cohort study | subject |
|  | M. K. Iseselo and J. S. Ambikile | 2020 | Promoting Recovery in Mental Illness: The Perspectives of Patients, Caregivers, and Community Members in Dar es Salaam, Tanzania | subject |
|  | H. Farley | 2019 | Promoting self-efficacy in patients with chronic disease beyond traditional education: A literature review | subject |
|  | C. O'Callaghan, A. Tran, N. Tam, L. M. Wen and R. Harris | 2022 | Promoting the get healthy information and coaching service (GHS) in Australian-Chinese communities: facilitators and barriers | subject |
|  | M. Shepherd-Banigan, S. D. Hohl, C. Vaughan, G. Ibarra, E. Carosso and B. Thompson | 2014 | The Promotora Explained Everything...Participant Experiences During a Household-Level Diabetes Education Program | subject |
|  | A. D. Wake | 2022 | Protective effects of physical activity against health risks associated with type 1 diabetes: "Health benefits outweigh the risks" | subject |
|  | A. J. Adler, T. Ruderman, F. Valeta, L. Drown, C. Trujillo et al. | 2022 | Protocol for a feasibility randomised control trial for continuous glucose monitoring in patients with type 1 diabetes at first-level hospitals in rural Malawi | subject |
|  | H. McGloin, D. Devane, C. D. McIntosh, K. Winkley and G. Gethin | 2021 | Psychological interventions for treating foot ulcers, and preventing their recurrence, in people with diabetes | subject |
|  | V. Mogre, Z. O. Abanga, F. Tzelepis, N. A. Johnson and C. Paul | 2019 | Psychometric evaluation of the summary of diabetes self-care activities measure in Ghanaian adults living with type 2 diabetes | subject |
|  | E. Watanabe-Fujinuma, H. Origasa, L. Bamber, L. Roessig, T. Toyoda et al. | 2020 | Psychometric properties of the Japanese version of the Kansas City Cardiomyopathy Questionnaire in Japanese patients with chronic heart failure | subject |
|  | K. B. Pressly | 1995 | Psychosocial characteristics of CAPD patients and the occurrence of infectious complications | subject |
|  | K. B. Pressly | 1995 | Psychosocial characteristics of CAPD patients and the occurrence of infectious complications... including commentary by Prowant B with author response | subject |
|  | M. Amankwah-Poku, A. G. B. Amoah, A. Sefa-Dedeh and J. Akpalu | 2020 | Psychosocial distress, clinical variables and self-management activities associated with type 2 diabetes: a study in Ghana | subject |
|  | J. S. Grant, L. J. Graven, G. Schluck and L. Abbott | 2021 | Psychosocial predictors of adverse outcomes in rural heart failure caregivers | subject |
|  | https://www.rrh.org.au/journal/article/6497 | 2022 | Psychosocial predictors of adverse outcomes in rural heart failure caregivers | subject |
|  | H. d'Emden, B. McDermott, N. D'Silva, T. Dover, T. Ewais et al. | 2017 | Psychosocial screening and management of young people aged 18-25 years with diabetes | subject |
|  |  | 2019 | Public health round-up | subject |
|  | J. Jones, R. T. Goins, M. Schure, B. Winchester and V. Bradley | 2020 | Putting Self-Management in the Context of Community-Dwelling American Indians Living With Type 2 Diabetes | subject |
|  | P. J. Klein, J. Baumgarden and R. Schneider | 2019 | Qigong and Tai Chi as Therapeutic Exercise: Survey of Systematic Reviews and Meta-Analyses Addressing Physical Health Conditions | subject |
|  | V. F. Malo, T. D. Ritchwood, L. J. Hart and M. Atujuna | 2022 | A qualitative analysis of family support for adolescent HIV care in South Africa | subject |
|  | M. O. Ogunlana, P. Govender, O. O. Oyewole, A. C. Odole, J. L. Falola et al. | 2021 | Qualitative exploration into reasons for delay in seeking medical help with diabetic foot problems | Population |
|  | W. Shahin, G. A. Kennedy and I. Stupans | 2021 | A qualitative exploration of the impact of knowledge and perceptions about hypertension in medication adherence in Middle Eastern refugees and migrants | Population |
|  | T. Ogunrinu, T. Gamboa-Maldonado, R. N. Ngewa, J. Saunders, J. Crounse and J. Misiri | 2017 | A qualitative study of health education experiences and self-management practices among patients with type 2 diabetes at Malamulo Adventist Hospital in Thyolo District, Malawi | Population |
|  | T. Ogunrinu, T. Gamboa-Maldonado, R. N. Ngewa, J. Saunders, J. Crounse and J. Misiri | 2017 | A qualitative study of health education experiences and self-management practices among patients with type 2 diabetes at Malamulo Adventist Hospital in Thyolo District, Malawi | Population |
|  | K. Murphy, T. Chuma, C. Mathews, K. Steyn and N. Levitt | 2015 | A qualitative study of the experiences of care and motivation for effective self-management among diabetic and hypertensive patients attending public sector primary health care services in South Africa | Population |
|  | C. B. Kabeza, L. Harst, P. E. H. Schwarz and P. Timpel | 2020 | A qualitative study of users' experiences after 3 months: the first Rwandan diabetes self-management Smartphone application "Kir'App" | subject |
|  | R. Matima, K. Murphy, N. S. Levitt, R. BeLue and T. Oni | 2018 | A qualitative study on the experiences and perspectives of public sector patients in Cape Town in managing the workload of demands of HIV and type 2 diabetes multimorbidity | Methods |
|  | T. Muhoma, M. W. Waruiru, O. Sanni, L. D. Knecht and M. McFarland | 2020 | A quality improvement project to improve diabetes self-management and patient satisfaction in a low-resourced central Kenyan hospital | Subject |
|  | V. Krishnappa, E. George, M. Oravec, R. Jones, A. Lee and D. Sweet | 2022 | Quality improvement project to improve providers' goal-setting activity for chronic disease self-management | Subject |
|  | A. Kirkegaard, L. Ball, L. Mitchell, B. Brickley and L. T. Williams | 2022 | Quality improvement strategies enhance primary care dietetics: A systematic review and meta-analysis | Review |
|  | B. Mash, H. Rhode, M. Pather, G. Ainslie, E. Irusen et al. | 2009 | Quality of asthma care: Western Cape province, South Africa | Subject |
|  | C. F. Otieno, M. Kariuki and L. Ng'ang'a | 2003 | Quality of glycaemic control in ambulatory diabetics at the out-patient clinic of Kenyatta National Hospital, Nairobi | Subject |
|  | T. Heikinheimo and D. Chimbayo | 2015 | Quality of life after first-ever stroke: an interview-based study from Blantyre, Malawi | Subject |
|  | Z. Abbas, M. A. Elseed and I. N. Mohammed | 2014 | The quality of life among Sudanese children with epilepsy and their care givers | Subject |
|  | M. B. Pastor-Robles, A. Mayo-Íscar, R. M. Cárdaba-García and V. Niño-Martín | 2022 | Quality of Life in Women over 65 Years of Age Diagnosed with Osteoporosis | Subject |
|  | O. S. Ojo, S. O. Malomo, A. O. Egunjobi, A. O. A. Jimoh and M. O. Olowere | 2018 | Quality of primary care physicians' communication of diabetes self-management during medical encounters with persons with diabetes mellitus in a resource-poor country | Subject |
|  | O. S. Ojo, S. O. Malomo, A. O. Egunjobi, A. O. A. Jimoh, M. O. Olowere | 2022 | Quality of primary care physicians’ communication of diabetes self-management during medical encounters with persons with diabetes mellitus in a resource-poor country | Subject |
|  | L. S. Eller, K. M. Kirksey, P. K. Nicholas, I. B. Corless, W. L. Holzemer et al. | 2013 | A randomized controlled trial of an HIV/AIDS Symptom Management Manual for depressive symptoms | Subject |
|  | https://www.tandfonline.com/loi/caic20 | 2022 |  | Invalid |
|  | J. B. Nachega, R. E. Chaisson, R. Goliath, A. Efron, M. A. Chaudhary et al. | 2010 | Randomized controlled trial of trained patient-nominated treatment supporters providing partial directly observed antiretroviral therapy | Subject |
|  | G. Zisis, Q. Huynh, Y. Yang, C. Neil, M. J. Carrington et al. | 2020 | Rationale and design of a risk-guided strategy for reducing readmissions for acute decompensated heart failure: the Risk-HF study | Subject |
|  | SJC Taylor, H Pinnock, E Epiphaniou, G Pearce, HL Parke et al. | 2014 | A rapid synthesis of the evidence on interventions supporting self-management for people with long-term conditions: PRISMS – Practical systematic RevIew of Self-Management Support for long-term conditions | Review |
|  | Bhandari B, Narasimhan P, Vaidya A, Subedi M, Jayasuriya R. | 2021 | [Barriers and facilitators for treatment and control of high blood pressure among hypertensive patients in Kathmandu, Nepal: a qualitative study informed by COM-B model of behavior change.](https://pubmed.ncbi.nlm.nih.gov/34372808/) |  |
|  | Bhattarai S, Bajracharya S, Shrestha A, Skovlund E, Åsvold BO, Mjolstad BP, Sen A. | 2023 | Facilitators and barriers to hypertension management in urban Nepal: findings from a qualitative study. | Settings |
|  | O. Moonian, A. Jodheea-Jutton, K. K. Khedo, S. Baichoo, S. D. Nagowah et al. | 2020 | Recent advances in computational tools and resources for the self-management of type 2 diabetes | Subject |
|  | K. C. Anukam, E. O. Osazuwa, G. Reid and M. N. Katsivo | 2004 | Receptivity for probiotic products among premenopausal female students in an African University | Subject |
|  | A, Kingsley C. O, Emmanuel; R, Gregor, K, Melanie N. | 1969 | Receptivity for Probiotic Products Among Premenopausal Female Students in an African University | Subject |
|  | K. C. Anukam, E. O. Osazuwa, G. Reid, M. N. Katsivo, K. C. Anukam et al. | 2002 | Receptivity for probiotic products among premenopausal female students in an African university | Subject |
|  | T. K. Masupe, K. Ndayi, L. Tsolekile, P. Delobelle and T. Puoane | 2018 | Redefining diabetes and the concept of self-management from a patient's perspective: implications for disease risk factor management | Subject |
|  | R. Chafe, D. Albrechtsons, D. Hagerty and L. A. Newhook | 2015 | Reducing episodes of diabetic ketoacidosis within a youth population: a focus group study with patients and families | Subject |
|  | T. Yordanos Mequanint | 2012 | Refashioning stigma: Experiencing and managing HIV/AIDS in the biomedical era | Subject |
|  | J. S. Lewis, E. K. Stokes, B. Gojanovic, P. Gellatly, C. Mbada et al. | 2021 | Reframing how we care for people with persistent non-traumatic musculoskeletal pain. Suggestions for the rehabilitation community | Subject |
|  | H. Bao | 2021 | Relationship among family support, mental resilience and diabetic distress in patients with type 2 diabetic mellitus during COVID-19 | Subject |
|  | H. Bao | 2021 | Relationship among Family Support, Mental Resilience and Diabetic Distress in Patients with Type 2 Diabetic Mellitus during COVID-19 | Invalid |
|  | N. Tusa, H. Kautiainen, P. Elfving, S. Sinikallio and P. Mäntyselkä | 2020 | Relationship between patient activation measurement and self-rated health in patients with chronic diseases | Subject |
|  | T. Tarwirei, G. Nkhoma, D. Mukona and M. Zvinavashe | 2013 | The relationship between perceived self-efficacy and adherence to self-care activities in type 2 diabetic clients | Method |
|  | J. T. Onyango, J. F. Namatovu, I. K. Besigye, M. Kaddumukasa and S. N. Mbalinda | 2022 | The relationship between perceived social support from family and diabetes self-management among patients in Uganda | Method |
|  | D. Calli and A. Kartal | 2021 | The relationship between self-efficacy of diabetes management and well-being in patients with type 2 diabetes | Method |
|  | S. O. Iwuala, M. A. Olamoyegun, A. A. Sabir and O. A. Fasanmade | 2015 | The relationship between self-monitoring of blood glucose and glycaemic control among patients attending an urban diabetes clinic in Nigeria | Method |
|  | F. Aga, S. B. Dunbar, T. Kebede, M. K. Higgins and R. Gary | 2020 | Relationships of diabetes self-care behaviours to glycaemic control in adults with type 2 diabetes and comorbid heart failure | Method |
|  | C. Figueroa, C. Johnson, N. Ford, A. Sands, S. Dalal et al. | 2018 | Reliability of HIV rapid diagnostic tests for self-testing compared with testing by health-care workers: a systematic review and meta-analysis | Subject |
|  | C. Harichund, P. Kunene, S. Simelane, Q. Abdool Karim and M. Moshabela | 2019 | Repeat HIV testing practices in the era of HIV self-testing among adults in KwaZulu-Natal, South Africa | Subject |
|  | W. Ekezie | 2022 | Resilience actions of Internally Displaced Persons (IDPs) living in camp-like settings: a Northern Nigeria case study | Subject |
|  | S. Sihvola, L. Kuosmanen and T. Kvist | 2022 | Resilience and related factors in colorectal cancer patients: A systematic review | Subject |
|  | N. Beckmann | 2013 | Responding to medical crises: AIDS treatment, responsibilisation and the logic of choice | Subject |
|  | M. J. Abawari, D. Amdisa, Z. Birhanu and Y. Kebede | 2021 | Response to self-care practice messages among patients with diabetes mellitus visiting Jimma University medical center facility based cross sectional design application of extended parallel process model | Subject |
|  | S. W. Darzins, C. Imms, N. Shields and N. F. Taylor | 2015 | Responsiveness, construct and criterion validity of the Personal Care-Participation Assessment and Resource Tool (PC-PART) | Subject |
|  | T. S. Tang, P. S. Sohal and A. K. Garg | 2015 | Rethinking peer support for diabetes in Vancouver's South-Asian community: a feasibility study | Subject |
|  | D. Sagastume, E. Mertens, D. K. Sibongwere, J.-C. Dimbelolo, J. C. K. Kabundi et al. | 2022 | A retrospective database study of the demographic features and glycemic control of patients with type 2 diabetes in Kinshasa, Democratic Republic of the Congo | Subject |
|  | D. Sagastume, E. Mertens, D. K. Sibongwere, J. C. Dimbelolo, J. C. K. Kabundi et al. | 2022 | A retrospective database study of the demographic features and glycemic control of patients with type 2 diabetes in Kinshasa, Democratic Republic of the Congo | Repetition |
|  | P. Smith, J. Clayton, C. Pike and L.-G. Bekker | 2019 | A review of the atomoRapid HIV self-testing device: an acceptable and easy alternative to facilitate HIV testing | Subject |
|  | P. N. Nyamu, C. F. Otieno, E. O. Amayo and S. O. McLigeyo | 2003 | Risk factors and prevalence of diabetic foot ulcers at Kenyatta National Hospital, Nairobi | Subject |
|  | Y. Yao, G. Ding, L. Wang, Y. Jin, J. Lin et al. | 2019 | Risk factors for depression in empty nesters: a cross-sectional study in a coastal city of Zhejiang Province and China | Subject |
|  | Y. Yao, G. Ding, L. Wang, Y. Jin, J. Lin et al. | 2009 | Risk Factors for Depression in Empty Nesters: A Cross-Sectional Study in a Coastal City of Zhejiang Province and China | Repetition |
|  | C.-T. Chao, R.-S. Yang, W.-J. Huang, K.-S. Tsai and D.-C. D. Chan | 2019 | Risk Factors for Poor Functional Recovery, Mortality, Recurrent Fractures, and Falls Among Patients Participating in a Fracture Liaison Service Program | Subject |
|  | A. Sotiropoulos, E. A. Skliros, C. Tountas, U. Apostolou, T. A. Peppas and S. I. Pappas | 2005 | Risk factors for severe hypoglycaemia in type 2 diabetic patients admitted to hospital in Piraeus, Greece | Subject |
|  | C. Frobisher, A. Glaser, G. A. Levitt, D. J. Cutter, D. L. Winter et al. | 2017 | Risk stratification of childhood cancer survivors necessary for evidence-based clinical long-term follow-up | Subject |
|  | M. V. Zakhele and S. Peng | 2019 | Risky behaviour among intravenous drug users (IDUS) with respect to the self-management of needle-inflicted wounds, damaged veins, and limb ulcers: a comparative descriptive review between South Africa and China | Subject |
|  | [https://krepublishers.com/02-Journals/S-EM/EM-13-0-000-19-Web/S-EM-13-2-000-19-Abst-PDF/S-EM-13-02-094-19-589-Zakhele-M-V/S-EM-13-02-094-19-589-%20Zakhele-M-V-Tx[5].pmd.pdf](https://krepublishers.com/02-Journals/S-EM/EM-13-0-000-19-Web/S-EM-13-2-000-19-Abst-PDF/S-EM-13-02-094-19-589-Zakhele-M-V/S-EM-13-02-094-19-589-%20Zakhele-M-V-Tx%5b5%5d.pmd.pdf) | 2019 |  | Invalid |
|  | G. R. Arthur, G. Ngatia, C. Rachier, R. Mutemi, J. Odhiambo and C. F. Gilks | 2005 | The role for government health centers in provision of same-day voluntary HIV counseling and testing in Kenya | Subject |
|  | R. Nalugya, S. Russell, F. Zalwango and J. Seeley | 2018 | The role of children in their HIV-positive parents' management of antiretroviral therapy in Uganda | Subject |
|  | https://www.tandfonline.com/loi/raar20 | 44268 |  | Invalid |
|  | B. A. Egbujie, P. A. Delobelle, N. Levitt, T. Puoane, D. Sanders and B. van Wyk | 2018 | Role of community health workers in type 2 diabetes mellitus self-management: A scoping review | Review |
|  | F. Aga, S. B. Dunbar, T. Kebede and R. A. Gary | 2019 | The role of concordant and discordant comorbidities on performance of self-care behaviors in adults with type 2 diabetes: a systematic review | Review |
|  | M. M. Kebede, C. Schuett and C. R. Pischke | 2019 | The Role of Continuous Glucose Monitoring, Diabetes Smartphone Applications, and Self-Care Behavior in Glycemic Control: Results of a Multi-National Online Survey | subject |
|  | M. Sahu, A. Grover and A. Joshi | 2014 | Role of mobile phone technology in health education in Asian and African countries: a systematic review | subject |
|  | M. Narasimhan, C. H. Logie, K. Moody, J. Hopkins, O. Montoya and A. Hardon | 2021 | The role of self-care interventions on men's health-seeking behaviours to advance their sexual and reproductive health and rights | subject |
|  | S. S. Mohamed, A. A. Mahmoud and A. A. Ali | 2014 | The role of Sudanese community pharmacists in patients' self-care | subject |
|  | A. Idriss, K. Diaconu, G. Zou, R. G. Senesi, H. Wurie and S. Witter | 2020 | Rural-urban health-seeking behaviours for non-communicable diseases in Sierra Leone | subject |
|  | A. Idriss, K. Diaconu, G. Zou, R. G. B. Senesi, H. Wurie and S. Witter | 2020 | Rural-urban health-seeking behaviours for non-communicable diseases in Sierra Leone | subject |
|  | https://gh.bmj.com/content/5/2/e002024 | 2018 | Rural–urban health-seeking behaviours for non-communicable diseases in Sierra Leone | Invalid |
|  | H. Natvig, A.H. Eide , M.H. Døving, A. Linge Hessen, E. Hoel et al | 2014 | Self- and collateral spouse-reported alcohol use in Malawi: Exploring social drinking norms’ potential for alcohol prevention | Subject |
|  | R. Kasirye, C. Laurenzi, A. Nabulya and B. Nakijoba | 2023 | Safe spaces, vocational training, and prevention programs protect young Ugandan women: findings from Uganda Youth Development Link's DREAMS initiative for rural communities | subject |
|  | E. Renard, H. Ikegami, A. G. Daher Vianna, P. Pozzilli, S. Brette et al. | 2021 | The SAGE study: Global observational analysis of glycaemic control, hypoglycaemia and diabetes management in T1DM | subject |
|  | J. van Olmen, S. Menon, A. Poplas Susič, P. Ir, K. Klipstein-Grobusch et al. | 2020 | Scale-up integrated care for diabetes and hypertension in Cambodia, Slovenia and Belgium (SCUBY): a study design for a quasi-experimental multiple case study | subject |
|  | M. Chimara, L. v. Niekerk and H. M. v. Biljon | 2022 | Scoping review exploring vocational rehabilitation interventions for mental health service users with chronic mental illness in low-income to upper-middle-income countries | subject |
|  | https://bmjopen.bmj.com/content/12/5/e059211 | 2015 | Scoping review exploring vocational rehabilitation interventions for mental health service users with chronic mental illness in low-income to upper-middle-income countries | Repetition |
|  | K. Ayele, B. Tesfa, L. Abebe, T. Tilahun and E. Girma | 2012 | Self care behavior among patients with diabetes in Harari, Eastern Ethiopia: the health belief model perspective | Method |
|  | M. Luacche Xavier Ferreira de Sousa, K. de Lima Silva, M. M. Lima da NÃ³brega and N. Collet | 2012 | Self-care deficits in children and adolescents with chronic kidney disease | subject |
|  | E. T. Ugwu, C. J. G. Orjioke and E. E. Young | 2018 | Self Monitoring of Blood Glucose Among Patients with Type 2 Diabetes Mellitus in Eastern Nigeria: Need for Multi-strategic Interventions | Method |
|  | A. Getie, B. Geda, T. Alemayhu, A. Bante, Z. Aschalew and B. Wassihun | 2020 | Self- care practices and associated factors among adult diabetic patients in public hospitals of Dire Dawa administration, Eastern Ethiopia | Method |
|  | D. M. Mukona and M. Zvinavashe | 2020 | Self- management of diabetes mellitus during the Covid-19 pandemic: Recommendations for a resource limited setting | subject |
|  | C. Tangus, G. Githemo and L. Onsongo | 2022 | Self-care abilities of patients on maintenance hemodialysis at Kenyatta National Hospital Renal Unit, Kenya | Method |
|  | I. L. Jackson, S. I. Onung and E. P. Oiwoh | 2021 | Self-care activities, glycaemic control and health-related quality of life of patients with type 2 diabetes in a tertiary hospital in Nigeria | Method |
|  | L. M. Geteri and E. M. Angogo | 2013 | Self-care among caregivers of people living with HIV and AIDS in Kakola location, Nyando District, Kisumu County, Kenya | subject |
|  | https://www.tandfonline.com/doi/full/10.1080/17290376.2013.807065 | 2013 | Self-care among caregivers of people living with HIV and AIDS in Kakola location, Nyando District, Kisumu County, Kenya | Subject |
|  | M. E. Smith, N. Robinowitz, P. Chaulk and K. E. Johnson | 2014 | Self-care and risk reduction habits in older injection drug users with chronic wounds: a cross-sectional study | subject |
|  | T. Hailu Gebru, K. Kidanu Berhe, W. Tilahun Tsehaye, H. Hagos Mekonen, K. Gemechu Kiros et al. | 2021 | Self-Care Behavior and Associated Factors among Heart Failure Patients in Tigray, Ethiopia: A Cross-Sectional Study | subject |
|  | J. K. Kemppainen, D. Wantland, J. Voss, P. Nicholas, K. M. Kirksey et al. | 2012 | Self-Care Behaviors and Activities for Managing HIV-Related Anxiety | subject |
|  | J. K. Kemppainen, D. Wantland, J. Voss, P. Nicholas, K. M. Kirksey et al. | 2012 | Self-care behaviors and activities for managing HIV-related anxiety | subject |
|  | L. C. Baumann, C. K. Opio, M. Otim, L. Olson and S. Ellison | 2010 | Self-care beliefs and behaviors in Ugandan adults with type 2 diabetes | subject |
|  | A. Nyatela, S. Nqakala, L. Singh, T. Johnson and S. Gumede | 2022 | Self-care can be an alternative to expand access to universal health care: What policy makers, governments and implementers can consider for South Africa | subject |
|  | M. V. Bayem, M. B. Oriaran, A. C. Olugbade and D. O. Ogbu | 2019 | Self-Care Challenges among Diabetic Patients in a South-Southern Teaching Hospital, Nigeria | subject |
|  | S. Pettersson, T. Jaarsma, K. Hedgärd and L. Klompstra | 2022 | Self-care in migrants with type 2 diabetes, during the COVID-19 pandemic | subject |
|  | K. Rahel Nega, H. Hana Abera, H. Mekdes Hailegebreal and G. Altayework Mekonnen | 2021 | Self-care knowledge, attitude and associated factors among outpatients with diabetes mellitus in Arsi Zone, Southeast Ethiopia | Method |
|  | K. Rahel Nega, H. Hana Abera, H. Mekdes Hailegebreal and G. Altayework Mekonnen | 2021 | Self-care knowledge, attitude and associated factors among outpatients with diabetes mellitus in Arsi Zone, Southeast Ethiopia | Invalid |
|  | B. H. Makhubela | 2002 | The self-care model of best practice: home based care | subject |
|  | K. Alsaqer and H. Bebis | 2022 | Self-care of hypertension of older adults during COVID-19 lockdown period: a randomized controlled trial | Method |
|  | A. van Puffelen, M. Kasteleyn, L. de Vries, M. Rijken, M. Heijmans et. al. | 2020 | Self-care of patients with type 2 diabetes mellitus over the course of illness: implications for tailoring support | Method |
|  | T. Melaku, B. Bayisa, H. Fekeremaryam, A. Feyissa and A. Gutasa | 2022 | Self-care practice among adult hypertensive patients at ambulatory clinic of tertiary teaching Hospital in Ethiopia: a cross-sectional study | method |
|  | M. Tsegaye, B. Bodena, F. Helen, F. Alemayehu and G. Alemayehu | 2022 | Self-care practice among adult hypertensive patients at ambulatory clinic of tertiary teaching Hospital in Ethiopia: a cross-sectional study | Method |
|  | M. Tsegaye, B. Bodena, F. Helen, F. Alemayehu and G. Alemayehu | 2022 | Self-care practice among adult hypertensive patients at ambulatory clinic of tertiary teaching Hospital in Ethiopia: a cross-sectional study | Repetition |
|  | M. Tsegaye, B. Bodena, F. Helen, F. Alemayehu and G. Alemayehu | 2021 | Self-care practice and associated factors among adult asthmatic patients on follow-up care at Northwest Amhara referral hospitals, Northwest Ethiopia 2020 | Subject |
|  | S. W. Chali, M. H. Salih and A. T. Abate | 2021 | Self-care practice and associated factors among Diabetes Mellitus patients on follow up in Benishangul Gumuz Regional State Public Hospitals, Western Ethiopia: a cross-sectional study | Method |
|  | B. Assefa, H. Zeleke, T. Sergo, M. Misganaw and N. Mekonnen | 2022 | Self-care practice and associated factors among hypertensive follow-up patients at East Gojam zone public hospitals, North West Ethiopia, 2021 | Method |
|  | A. Wondmieneh, G. Gedefaw, A. Getie and A. Demis | 2021 | Self-Care Practice and Associated Factors among Hypertensive Patients in Ethiopia: A Systematic Review and Meta-Analysis | Review |
|  | F. M. Hussen, H. A. Adem, H. S. Roba, B. Mengistie and N. Assefa | 2020 | Self-care practice and associated factors among hypertensive patients in public health facilities in Harar Town, Eastern Ethiopia: A cross-sectional study | Method |
|  | S. Z. Gebre, B. Zegeye and M. M. Taderegew | 2020 | Self-Care Practice and Associated Factors Among Individuals with Diabetes Mellitus in Northeast Ethiopia | Method |
|  | A. Y. Aschalew, M. Yitayal, A. Minyihun and T. A. Bisetegn | 2019 | Self-care practice and associated factors among patients with diabetes mellitus on follow up at University of Gondar Referral Hospital, Gondar, Northwest Ethiopia | Method |
|  | S. Zewdie, G. Moges, A. Andargie and B. M. Habte | 2022 | Self-Care Practice and Associated Factors Among Patients with Type 2 Diabetes Mellitus at a Referral Hospital in Northern Ethiopia - A Mixed Methods Study | Method |
|  | M. Wolderufael and N. Dereje | 2022 | Self-Care Practice and Associated Factors Among People Living with Type 2 Diabetes in Addis Ababa, Ethiopia: A Facility-Based Cross-Sectional Study | Method |
|  | H. Endalew, W. H. Mariam, B. Tefera and B. Zewdie | 2012 | Self-care practice and glycaemic control amongst adults with diabetes at the Jimma University Specialized Hospital in south-west Ethiopia: a cross-sectional study | Method |
|  | A. Abebe, Y. Wobie, B. Kebede, A. Wale, A. Destaw and A. S. Ambaye | 2022 | Self-care practice and glycemic Control among type 2 diabetes patients on follow up in a developing country: a prospective observational study | Method |
|  | M. S. Emire, B. T. Zewudie, T. T. Tarekegn, F. A. GebreEyesus, B. T. Amlak et al. | 2022 | Self-care practice and its associated factors among diabetic patients attending public hospitals in Gurage zone southwest, Ethiopia | Method |
|  | S. D. Bayable, A. Misganaw and Y. Guadie Ashebir | 2022 | Self-care practice and its predictors among adult diabetic patients on follow-up at public health care diabetic referral clinics, Debre Markos, Ethiopia | Methods |
|  | R. N. Kassa, I. Y. Ibrahim, H. A. Hailemariam and M. H. Habte | 2021 | Self-care practice and its predictors among adults with diabetes mellitus on follow up at public hospitals of Arsi zone, southeast Ethiopia | Methods |
|  | J. Burman, A. Bhattacharya, A. Chattopdhyay, I. Dey, S. Sembiah and R. Negi | 2021 | Self-care practice and its predictors amongst Type-2 Diabetes Mellitus patients in the outpatient department of a tertiary hospital of Kolkata, Eastern India - A cross-sectional study | Methods |
|  | B. Jayeeta, B. Aritra, C. Amitabha, D. Indira, S. Sembagamuthu and N. Rudresh | 2021 | Self-care practice and its predictors amongst Type-2 Diabetes Mellitus patients in the outpatient department of a tertiary hospital of Kolkata, Eastern India - a cross-sectional study | Setting |
|  | https://www.jfmpc.com/article.asp?issn=2249-4863;year=2021;volume=10;issue=3;spage=1377;epage=1382;aulast=Burman |  |  | Invalid |
|  | https://www.jfmpc.com/article.asp?issn=2249-4863;year=2021;volume=10;issue=3;spage=1377;epage=1382;aulast=Burman |  |  | Invalid |
|  | E. K. Bogale, H. Wondiye, Y. Debela, T. Fentabil Anagaw, L. Worku and N. Kebede | 2022 | Self-care practice, lived experience of type 1 diabetes mellitus patients at Kemisse General Hospital, North Eastern Ethiopia: Phenomenological study | Population |
|  | M. Gode, F. Aga and A. Hailu | 2022 | Self-Care Practices Among Adult Type 2 Diabetes Patients With and Without Peripheral Neuropathy: A Cross-Sectional Study at Tertiary Healthcare Settings in Ethiopia | Methods |
|  | D. Tewahido and Y. Berhane | 2017 | Self-Care Practices among Diabetes Patients in Addis Ababa: A Qualitative Study | Population |
|  | T. W. Abate, M. Tareke and M. Tirfie | 2018 | Self-care practices and associated factors among diabetes patients attending the outpatient department in Bahir Dar, Northwest Ethiopia | Methods |
|  | L. Abdisa, B. Balis, K. Shiferaw, A. Debella, H. Bekele et al. | 2022 | Self-care practices and associated factors among hypertension patients in public hospitals in Harari regional state and Dire Dawa City administration, Eastern Ethiopia: A multi-center cross-sectional study | Methods |
|  | S. Letta, F. Aga, T. Assebe Yadeta, B. Geda and Y. Dessie | 2022 | Self-care practices and correlates among patients with type 2 diabetes in Eastern Ethiopia: A hospital-based cross-sectional study | Methods |
|  | Gizachew Ambaw Kassie et al | 2024 | Self-care practices and its associated factors among adult diabetes mellitus patients in public hospitals of Sidama region, Southern Ethiopia: a cross-sectional study | Methods |
|  | M. Musheke, V. Bond and S. Merten | 2013 | Self-care practices and experiences of people living with HIV not receiving antiretroviral therapy in an urban community of Lusaka, Zambia: implications for HIV treatment programmes | Population |
|  | M. G. Dedefo, B. M. Ejeta, G. B. Wakjira, G. F. Mekonen and B. G. Labata | 2019 | Self-care practices regarding diabetes among diabetic patients in West Ethiopia | Methods |
|  | O. Titilayo and A. Modupe | 2022 | Self-Care Related Knowledge and Self-care Practices among Type 2 Diabetic Patients Attending Selected Hospitals in Oyo State, Nigeria | Methods |
|  | Y. Mekonnen and N. Hussien | 2021 | Self-care Related Knowledge, Attitude, and Practice and Associated Factors Among Patients with Type 2 Diabetes in JMC, Ethiopia | Methods |
|  | N. Haftom, B. Goitom, F. Girmatsion, D. Tesfaye and G. Goitom | 2019 | Self-care related knowledge, attitude, practice and associated factors among patients with diabetes in Ayder Comprehensive Specialized Hospital, North Ethiopia | Methods |
|  | H. Niguse, G. Belay, G. Fisseha, T. Desale and G. Gebremedhn | 2019 | Self-care related knowledge, attitude, practice and associated factors among patients with diabetes in Ayder Comprehensive Specialized Hospital, North Ethiopia | Methods |
|  | R. R. M. Modeste and S. J. Majeke | 2010 | Self-care symptom-management strategies amongst women living with hiv/aids in an urban area in kwazulu-natal | Population |
|  | R. S. D. Mandishora, T. B. Rounge, M. Fitzpatrick, I. K. Christiansen, O. H. Ambur et al. | 2021 | Self-collected and clinician-collected anal swabs show modest agreement for HPV genotyping | Subject |
|  | Dube Mandishora RS, Rounge TB, Fitzpatrick M, Christiansen IK, Ambur OH, et al. | 1984 | Self-collected and clinician-collected anal swabs show modest agreement for HPV genotyping | Invalid |
|  | D. Obiri-Yeboah, Y. Adu-Sarkodie, F. Djigma, A. Hayfron-Benjamin, L. Abdul et al. | 2017 | Self-collected vaginal sampling for the detection of genital human papillomavirus (HPV) using careHPV among Ghanaian women | Subject |
|  | S. M. Mitchell, H. N. Pedersen, E. Eng Stime, M. Sekikubo, E. Moses et al. | 2017 | Self-collection based HPV testing for cervical cancer screening among women living with HIV in Uganda: a descriptive analysis of knowledge, intentions to screen and factors associated with HPV positivity | Subject |
|  | E. Chamand | 2014 | Self-dialysis nursing practice in Mayotte] | Subject |
|  | H. A. Areri, A. Marshall and G. Harvey | 2021 | Self-efficacy for self-management and its influencing factors among adults living with HIV on antiretroviral therapy in northwest Ethiopia | Subject |
|  | A. Habtamu Abera, A. Marshall and G. Harvey | 2022 | Self-efficacy for self-management and its influencing factors among adults living with HIV on antiretroviral therapy in northwest Ethiopia | Subject |
|  | https://www.tandfonline.com/doi/full/10.1080/09540121.2021.2007839 | 2011 | Self-efficacy for self-management and its influencing factors among adults living with HIV on antiretroviral therapy in northwest Ethiopia | Repetition |
|  | E. Van't Hof, P. Cuijpers and D. J. Stein | 2009 | Self-help and Internet-guided interventions in depression and anxiety disorders: a systematic review of meta-analyses | Review |
|  | E. Van't Hof, P. Cuijpers and D. J. Stein | 2009 | Self-help and Internet-guided interventions in depression and anxiety disorders: a systematic review of meta-analyses | Repetition |
|  | D. Gela and D. Mengistu | 2018 | Self-management and associated factors among patients with end-stage renal disease undergoing hemodialysis at health facilities in Addis Ababa, Ethiopia | Population |
|  | E. Kumah, A. A. Abuosi, S. E. Ankomah and C. Anaba | 2021 | Self-management education program: the case of glycemic control of type 2 diabetes | Subject |
|  | https://www.omjournal.org/articleDetails.aspx?coType=2&aId=2846 | 2018 |  | Invalid |
|  | C. A. O'Brien, D. van Rooyen and E. Ricks | 2015 | SELF-MANAGEMENT EXPERIENCES OF PERSONS LIVING WITH DIABETES MELLITUS TYPE 2 | Population |
|  | S. D. Hlophe and K. Jooste | 2022 | Self-management experiences of youth following the unexpected loss of a family member to HIV | Population |
|  | M. Pienaar and M. Reid | 2020 | Self-management in face-to-face peer support for adults with type 2 diabetes living in low- or middle-income countries: a systematic review | Review |
|  | M. Pienaar and M. Reid | 2020 | Self-management in face-to-face peer support for adults with type 2 diabetes living in low- or middle-income countries: a systematic review | Review |
|  | M. Pienaar and M. Reid | 2020 | Self-management in face-to-face peer support for adults with type 2 diabetes living in low- or middle-income countries: a systematic review | Repetition |
|  | M. Pienaar and M. Reid | 2015 | Self-management in face-to-face peer support for adults with type 2 diabetes living in low- or middle-income countries: a systematic review | Repetition |
|  | T. Crowley and A. Rohwer | 2021 | Self-management interventions for adolescents living with HIV: a systematic review | Population |
|  | <https://link.springer.com/article/10.1186/s12879-021-06072-98> | 2021 |  | Invalid |
|  | A. Sav, M. A. King, F. Kelly, S. S. McMillan, E. Kendall et a. | 2015 | Self-management of chronic conditions in a rural and remote context | Population |
|  | V. Stephani, D. Opoku and D. Beran | 2018 | Self-management of diabetes in Sub-Saharan Africa: a systematic review | Review |
|  | J. Hearn, I. Ssinabulya, J. I. Schwartz, A. R. Akiteng, H. J. Ross and J. A. Cafazzo | 2019 | Self-management of non-communicable diseases in low- and middle-income countries: A scoping review | Review |
|  | C. O'Brien, D. v. Rooyen and E. Ricks | 2020 | Self-management of persons living with diabetes mellitus type 2: experiences of diabetes nurse educators | Subject |
|  | <https://hsag.co.za/index.php/hsag/article/view/1541/html> |  |  | Invalid |
|  | C. O'Brien, D. van Rooyen and E. Ricks | 2020 | Self-management of persons living with diabetes mellitus type 2: Experiences of diabetes nurse educators | Repetition |
|  | A. T. Nguyen | 2011 | Self-management of type 2 diabetes: perspectives of Vietnamese Americans | Setting |
|  | K. A. Al Johani, G. E. Kendall and P. D. Snider | 2013 | Self-management practices among type 2 diabetes patients attending primary health-care centres in Medina, Saudi Arabia | Setting |
|  | K. A. Al-Johani, G. E. Kendall and P. D. Snider | 2013 | Self-management practices among type 2 diabetes patients attending primary health-care centres in Medina, Saudi Arabia | Repetition |
|  | A. A. Druye, K. Nelson and B. Robinson | 2020 | Self-management recommendations for sickle cell disease: A content analysis of websites | subject |
|  | L. Dube, K. Rendall-Mkosi, S. Van den Broucke, A.-M. Bergh and N. G. Mafutha | 2017 | Self-Management Support Needs of Patients with Chronic Diseases in a South African Township: A Qualitative Study | Repetition |
|  | T. Copeland | 2018 | Self-managing HIV/AIDS: cultural competence and health among women in Nairobi, Kenya | Population |
|  | <https://onlinelibrary.wiley.com/doi/10.1111/hsc.13828> | 2022 |  | Invalid |
|  | M. J. T. van Het Bolscher-Niehuis, M. J. Uitdehaag, A. Bergsma and A. L. Francke | 2021 | Self-managing physical and mental health: A qualitative study on older adults' views and support needs in the Netherlands | Setting |
|  | M. J. T. van Het Bolscher-Niehuis, M. J. Uitdehaag, A. Bergsma and A. L. Francke | 2021 | Self-managing physical and mental health: A qualitative study on older adults' views and support needs in the Netherlands | Setting |
|  | A. Tuha, A. G. Faris, S. A. Mohammed and M. Y. Gobezie | 2020 | Self-Medication and Associated Factors Among Pregnant Women Attending Antenatal Care at Kemisie General Hospital, North East Ethiopia | Population |
|  | S. M. Ahmed, J. Sundby, Y. A. Aragaw and F. Abebe | 2020 | Self-medication and safety profile of medicines used among pregnant women in a tertiary teaching hospital in Jimma, Ethiopia: a cross-sectional study | subject |
|  | <https://www.mdpi.com/1660-4601/17/11/3993> | 2019 | Self-medication and safety profile of medicines used among pregnant women in a tertiary teaching hospital in Jimma, Ethiopia: a cross-sectional study | Repetition |
|  | N. Khadim, J. A. D. Tine, M. F. Zahra, B. Omar, F. B. Diongue, A. I. Diallo and F. Adama | 2020 | Self-medication of Senegalese women through social networks | Methods |
|  | <https://www.scirp.org/journal/paperinformation.aspx?> | 2022 |  | Invalid |
|  | A. Hounsa, L. Kouadio and P. d. Mol | 2010 | Self-medication with antibiotics obtained from private pharmacies in Abidjan, Ivory Coast | subject |
|  | https://www.sciencedirect.com/science/journal/0399077X | 2020 |  | Invalid |
|  | L. Grigoryan, F. M. Haaijer-Ruskamp, J. G. M. Burgerhof, R. Mechtler, R. Deschepper et al. | 2006 | Self-medication with antimicrobial drugs in Europe | Setting |
|  | J. Claude Mbanya, P. Aschner, J. C. N. Chan, J. Jose Gagliardino and J. Saji | 2017 | Self-monitoring of blood glucose (SMBG) and glycaemic control in Cameroon: Results of the International Diabetes Management Practices Study (IDMPS) | Methods |
|  | N. G. Malangu | 2008 | Self-reported adverse effects as barriers to adherence to antiretroviral therapy in HIV-infected patients in Pretoria | Methods |
|  | J. E. Prynn, A. Dube, E. Mwaiyeghele, O. Mwiba, S. Geis et al. | 2019 | Self-reported disability in rural Malawi: prevalence, incidence, and relationship to chronic conditions | subject |
|  | J. E. Prynn, A. Dube, E. Mwaiyeghele, O. Mwiba, S. Geis et al. | 2020 | Self-reported disability in rural Malawi: prevalence, incidence, and relationship to chronic conditions | Repetition |
|  | J. E. Prynn, A. Dube, E. Mwaiyeghele, O. Mwiba, S. Geis et al. | 2020 | Self-reported disability in rural Malawi: prevalence, incidence, and relationship to chronic conditions | Repetition |
|  | https://wellcomeopenresearch.org/articles/4-90 | 2019 | Self-reported disability in rural Malawi: prevalence, incidence, and relationship to chronic conditions | Repetition |
|  | J. E. Prynn, A. Dube, E. Mwaiyeghele, O. Mwiba, S. Geis et al. | 2021 | Self-reported disability in rural Malawi: prevalence, incidence, and relationship to chronic conditions | Repetition |
|  | J. E. Prynn, A. Dube, E. Mwaiyeghele, O. Mwiba, S. Geis et al. | 2019 | Self-reported disability in rural Malawi: prevalence, incidence, and relationship to chronic disease | Repetition |
|  | B. Smide, J. Lukwale, A. Msoka and K. Wikblad | 2002 | Self-reported health and glycaemic control in Tanzanian and Swedish diabetic patients | Subject |
|  | M. Chelenyane and R. Endacott | 2006 | Self-reported infection control practices and perceptions of HIV/AIDS risk amongst emergency department nurses in Botswana | Methods |
|  | W. Smyth, D. Lindsay, C. Holmes, A. Gardner and K. M. Rahman | 2016 | Self-reported long-term conditions of nurses and midwives across a northern Australian health service: A survey | subject |
|  | E. Giarelli | 2006 | Self-surveillance for genetic predisposition to cancer: behaviors and emotions | subject |
|  | A. C. van Dyk | 2013 | Self-testing as strategy to increase the uptake of HIV testing in South Africa | subject |
|  | S. Tonen-Wolyec, R. M. Djang'eing, S. Batina-Agasa, C. K. Tshilumba, J. M. Masidi, M. P. Hayette and L. Bélec | 2021 | Self-testing for HIV, HBV, and HCV using finger-stick whole-blood multiplex immunochromatographic rapid test: a pilot feasibility study in sub-Saharan Africa | subject |
|  | <https://journals.plos.org/plosone/article?id=10.1371/journal.pone.0249701> | 2 | Self-testing for HIV, HBV, and HCV using finger-stick whole-blood multiplex immunochromatographic rapid test: a pilot feasibility study in sub-Saharan Africa | Subject |
|  | S. Tonen-Wolyec, R. M. Djang'eing'a, S. Batina-Agasa, C. Kayembe Tshilumba, J. Muwonga Masidi, M. P. Hayette and L. Bélec | 2021 | Self-testing for HIV, HBV, and HCV using finger-stick whole-blood multiplex immunochromatographic rapid test: A pilot feasibility study in sub-Saharan Africa | Subject |
|  | T. K. Ruebush, M. K. Kern, C. C. Campbell and A. J. Oloo | 1995 | Self-treatment of malaria in a rural area of western Kenya | Subject |
|  | S. Pettersson, T. Jaarsma, K. Hedgärd and L. Klompstra | 2023 | Self‐care in migrants with type 2 diabetes, during the COVID‐19 pandemic | Subject |
|  | M. J. T. van het Bolscher‐Niehuis, M. J. Uitdehaag, A. Bergsma and A. L. Francke | 2022 | Self‐managing physical and mental health: A qualitative study on older adults' views and support needs in the Netherlands | Subject |
|  | Al Johani KA, Kendall GE, Snider | 2015 | Self-management practices among type 2 diabetes patients attending primary health-care centres in Medina, Saudi Arabia. Eastern Mediterranean | Setting |
|  | C. Winkler | 2021 | Personalised medicine and chronic kidney disease in sub-Saharan Africa: advances and challenges | Review |
|  | M. Alimohammadian, A. Majidi, M. Yaseri, B. Ahmadi, F. Islami, M. Derakhshan et al. | 2017 | Multimorbidity as an important issue among women: results of a gender difference investigation in a large population-based cross-sectional study in West Asia. | Setting |
|  | S. Nkomani, S. Ruskaniko and R. Blaauw | 2021 | The impact of existing diabetes self-management education interventions on knowledge, attitudes and practices in public health care institutions in Harare, Zimbabwe | Subject |
|  | L. L. Nkonki and K. L. Daniels | 2010 | Selling a service: experiences of peer supporters while promoting exclusive infant feeding in three sites in South Africa | Subject |
|  | M. Neuman, A. Mwinga, K. Kapaku, L. Sigande, C. Gotsche et al. | 2022 | Sensitivity and specificity of OraQuick® HIV self-test compared to a 4th generation laboratory reference standard algorithm in urban and rural Zambia | Subject |
|  | L. Perry, J. Dunbabin, X. Xu, J. Lowe, S. Acharya, S. James and K. S. Steinbeck | 2020 | Service use of young people with Type 1 diabetes after transition from paediatric to adult-based diabetes health care | Subject |
|  | C. H. Logie, H. Abela, T. Turk, S. Parker and K. Gholbzouri | 2021 | Sexual and reproductive health self-care interventions in the Eastern Mediterranean Region: findings from a cross-sectional values and preferences survey to inform WHO normative guidance on self-care interventions | Subject |
|  | https://link.springer.com/article/10.1186/s12961-020-00659-w | 2020 | Sexual and reproductive health self-care interventions in the Eastern Mediterranean Region: findings from a cross-sectional values and preferences survey to inform WHO normative guidance on self-care interventions | Invalid |
|  | Conserve DF, Bay C, Kilonzo MN, Makyao NE, Kajula L, Maman | 2019 | Sexual and social network correlates of willingness to self-test for HIV among ever-tested and never-tested men: implications for the Tanzania STEP project | Subject |
|  | https://www.tandfonline.com/loi/caic20 | 2011 |  | Invalid |
|  | K. Atkins, K. Rucinski, M. Mudavanhu, L. Holmes, L. Mutunga et al. | 2021 | Sexual Relationship Types, Partner HIV Self-Testing, and Pre-Exposure Prophylaxis Among South African Adolescent Girls and Young Women: A Latent Class Analysis | Subject |
|  | K. Malama, C. H. Logie, M. Narasimhan, L. Ouedraogo, C. Asmani, H. Elamin ert al. | 2022 | Short Communication: Awareness of HIV Self-Care Interventions Across Global Regions: Results from a Values and Preferences Survey | Subject |
|  | S. Helleringer, J. Mkandawire, G. Reniers, L. Kalilani-Phiri and H.-P. Kohler | 2013 | Should home-based HIV testing and counseling services be offered periodically in programs of ARV treatment as prevention? A case study in Likoma (Malawi) | Subject |
|  | G. Alemu Mersha, A. Tsegaw Woredekal and M. Tilahun Tesfaw | 2020 | Sight-threatening Diabetic Retinopathy and Associated Risk Factors Among Adult Diabetes Patients at Debre Tabor General Hospital, Northwest Ethiopia | Subject |
|  | A. Grimsrud, P. Ehrenkranz and I. Sikazwe | 2021 | Silver linings: how COVID-19 expedited differentiated service delivery for HIV | Subject |
|  | A. Giaccari, R. C. Bonadonna, R. Buzzetti, G. Perseghin, D. Cucinotta, C. Fanelli et al. | 2021 | Similar glycaemic control and risk of hypoglycaemia with patient- versus physician-managed titration of insulin glargine 300 U/mL across subgroups of patients with T2DM: a post hoc analysis of ITAS | Subject |
|  | R. Dowse, K. Barford and S. H. Browne | 2014 | Simple, illustrated medicines information improves ARV knowledge and patient self-efficacy in limited literacy South African HIV patients | Subject |
|  | C. Masquillier, L. Knight, L. Campbell, N. Sematlane, A. Delport, T. Dube and E. Wouters | 2020 | Sinako, a study on HIV competent households in South Africa: a cluster-randomised controlled trial protocol | Protocol |
|  | J. A. Denison, H. Banda, A. C. Dennis, C. Packer, N. Nyambe et al. | 2015 | The sky is the limit: adhering to antiretroviral therapy and HIV self-management from the perspectives of adolescents living with HIV and their adult caregivers | Population |
|  | Y. Getahun, W. R. Demissie and H. Amare | 2021 | Sleep quality among cardiac patients on follow up at Jimma Medical Center, southwestern Ethiopia | Subject |
|  | P. Absetz, J. Van Olmen, D. Guwatudde, T. Puoane, H. M. Alvesson et al. | 2020 | SMART2D-development and contextualization of community strategies to support self-management in prevention and control of type 2 diabetes in Uganda, South Africa, and Sweden | Subject |
|  | A. Mehbodniya, A. Suresh Kumar, K. P. Rane, K. K. Bhatia and B. K. Singh | 2021 | Smartphone-Based mHealth and Internet of Things for Diabetes Control and Self-Management | Subject |
|  | C. Déglise, L. S. Suggs and P. Odermatt | 2012 | SMS for disease control in developing countries: a systematic review of mobile health applications | Review |
|  | J. A. Nhavoto, Grönlund, ke and W. P. Chaquilla | 2015 | SMSaúde: Design, Development, and Implementation of a Remote/Mobile Patient Management System to Improve Retention in Care for HIV/AIDS and Tuberculosis Patients | Subject |
|  | J. A. Nhavoto, Å. Grönlund and W. P. Chaquilla | 2015 | SMSaúde: Design, Development, and Implementation of a Remote/Mobile Patient Management System to Improve Retention in Care for HIV/AIDS and Tuberculosis Patients | Repetition |
|  | K. G. Merrill, J. C. Campbell, C. E. Kennedy, V. M. Burke, S. Miti et al. | 2022 | 'So hurt and broken': a qualitative study of experiences of violence and HIV outcomes among Zambian youth living with HIV | Subject |
|  | K. G. Merrill, J. C. Campbell, C. E. Kennedy, V. M. Burke, S. Miti et al. | 2021 | 'So hurt and broken': a qualitative study of experiences of violence and HIV outcomes among Zambian youth living with HIV | Invalid |
|  | J. Seeley and S. Russel | 2010 | Social rebirth and social transformation? Rebuilding social lives after ART in rural Uganda | Subject |
|  | J. Seeley and S. Russell | 2010 | Social rebirth and social transformation? Rebuilding social lives after ART in rural Uganda | Subject |
|  | S. Ramkisson, B. J. Pillay and W. Sibanda | 2017 | Social support and coping in adults with type 2 diabetes | Subject |
|  | A. K. Tusubira, C. K. Nalwadda, A. R. Akiteng, E. Hsieh, C. Ngaruiya et al. | 2021 | Social Support for Self-Care: Patient Strategies for Managing Diabetes and Hypertension in Rural Uganda | Subject |
|  | W.-A. Lugaya, M. Mwenda, C. Syombua and E. M. Washika | 2022 | Social support received by diabetes mellitus Type II patients attending a country referral hospital in Kenya | Subject |
|  | E. Banchani, E. Y. Tenkorang, O. Sarfo-Kantaka and F. S. Sarfo | 2020 | Social Support Systems and the Self-Management of Non-Communicable Diseases (NCDs) in Ghana | Subject |
|  | F. Aga, S. B. Dunbar, T. Kebede, M. K. Higgins and R. Gary | 2020 | Sociodemographic and clinical correlates of diabetes self-efficacy in adults with type 2 diabetes and comorbid heart failure | Methods |
|  | S. Tonen-Wolyec, R.-S. Mboumba Bouassa, S. Batina-Agasa, A. T. Tepungipame, C. K. Tshilumba and L. Bélec | 2020 | Sociodemographic characteristics of adolescents preferring home-based HIV self-testing over facility-based voluntary counseling and testing: a cross-sectional study in Kisangani, Democratic Republic of the Congo | Population |
|  | N. M. Kamel, Y. A. Badawy, N. A. el-Zeiny and I. A. Merdan | 1999 | Sociodemographic determinants of management behaviour of diabetic patients. Part I. Behaviour of patients in relation to management of their disease | Methods |
|  | N. M. Kamel, Y. A. Badawy, N. A. el-Zeiny and I. A. Merdan | 1999 | Sociodemographic determinants of management behaviour of diabetic patients. Part II. Diabetics' knowledge of the disease and their management behaviour | Methods |
|  | G. D. Coronado, B. Thompson, S. Tejeda, R. Godina and L. Chen | 2007 | Sociodemographic factors and self-management practices related to type 2 diabetes among Hispanics and non-Hispanic whites in a rural setting | Population |
|  | G. D. Coronado, B. Thompson, S. Tejeda, R. Godina and L. Chen | 2007 | Sociodemographic factors and self-management practices related to type 2 diabetes among Hispanics and non-Hispanic whites in a rural setting | Population |
|  | <https://www.blackwell-synergy.com/doi/pdf/10.1111/j.1548-0301.2006.00067.x> | 2022 |  | Invalid |
|  | M. Lays | 2013 | A social science perspective on care for chronically ill people: relevance for public health and health care policy making | Subject |
|  | S. E. Mengoni, B. Gates, G. Parkes, D. Wellsted, G. Barton et al. | 2016 | Sometimes, it just stops me from doing anything: A qualitative exploration of epilepsy management in people with intellectual disabilities and their carers | Subject |
|  | W. Anderson-Loftin, S. Barnett, P. Bunn, P. Sullivan, J. Hussey and A. Tavakoli | 2005 | Soul food light: culturally competent diabetes education | Subject |
|  | Y. A. Tefera, K. G. Bishu, M. Gebregziabher, A. Z. Dawson and L. E. Egede | 2019 | Source of Education, Source of Care, Access to Glucometers, and Independent Correlates of Diabetes Knowledge in Ethiopian Adults with Diabetes | Methods |
|  | R. R. Marie Modeste and S. J. Majeke | 2014 | Sources and types of information on self-care symptom management strategies for HIV and AIDS | Subject |
|  | N. Copeling and K. Jooste | 2020 | A spotlight on the surfacing of self-management of employees with diabetes seen by professional nurses in selected occupational health clinics in Cape Town | Subject |
|  | K. B. Nkhoma, G. T. Mwalabu, K. Bristowe, E. A. Lungu and R. Harding | 2020 | Stakeholders' perspectives and requirements on pain self-management for adolescents living with HIV/AIDS in Malawi: a cross-sectional qualitative study | Population |
|  | M. Strauss, G. George, J. E. Mantell, M. L. Romo, E. Mwai et al. | 2018 | Stated and revealed preferences for HIV testing: can oral self-testing help to increase uptake amongst truck drivers in Kenya? | Subject |
|  | P. MacPherson, E. L. Webb, A. T. Choko, N. Desmond, K. Chavula et al. | 2011 | Stigmatising attitudes among people offered home-based HIV testing and counselling in Blantyre, Malawi: construction and analysis of a stigma scale | Subject |
|  | S. A. Everson, J. W. Lynch, G. A. Kaplan, T. A. Lakka, J. Sivenius and J. T. Salonen | 2001 | Stress-induced blood pressure reactivity and incident stroke in middle-aged men | Subject |
|  | W. Chen, C. Shiu, J. P. Yang, P. Chuang, L. Zhang, M. Bao and H. Lu | 2018 | A structural equation model of patient-healthcare provider relationships and HIV-infected patient outcomes in Chinese populations | Subject |
|  | W. Chen, C. Shiu, J. P. Yang, P. Chuang, L. Zhang, M. Bao and H. Lu | 2018 | A structural equation model of patient-healthcare provider relationships and HIV-infected patient outcomes in Chinese populations | Subject |
|  | <https://www.tandfonline.com/loi/900> | 2013 |  | Invalid |
|  | R. Lamptey, M. P. Robben, M. Amoakoh-Coleman, D. Boateng, D. E. Grobbee et al. | 2022 | Structured diabetes self-management education and glycaemic control in low- and middle-income countries: a systematic review | Review |
|  | R. Lamptey, M. P. Robben, M. Amoakoh-Coleman, D. Boateng, D. E. Grobbee et al. | 2022 | Structured diabetes self-management education and glycaemic control in low- and middle-income countries: a systematic review | Review |
|  | <https://onlinelibrary.wiley.com/doi/103.6611/dme.14812> | 2021 |  | Invalid |
|  | X. Debussche, S. Besançon, M. Balcou-Debussche, C. Ferdynus, H. Delisle et al. | 2018 | Structured peer-led diabetes self-management and support in a low-income country: The ST2EP randomised controlled trial in Mali | Subject |
|  | P. O. Anum, B. P. Anto and A. G. Forson | 2017 | Structured pharmaceutical care improves the health-related quality of life of patients with asthma | Subject |
|  | https://download.springer.com/static/pdf/215/ | 2022 |  | Invalid |
|  | J. A. Ndako, A. O. Owolabi, J. A. Olisa, J. A. Akinwumi, V. T. Dojumo et al. | 2020 | Studies on the prevalence of Hepatitis C virus infection in diabetic patients attending a tertiary health-care facility South-west Nigeria | Subject |
|  | D. Diallo, S. Fongoro, S. Doumbia, H. Maïga and C. Arama | 2011 | [Study of the quality of life of hemodialysis patients at the University Hospital of Point G (CHU) in Bamako- the study of 30 cases] | Subject |
|  | J. Zhang, Y. Zhang, Z. Luan, X. Zhang, H. Jiang and A. Wang | 2020 | A study on depression of the elderly with different sleep quality in pension institutions in Northeastern China | Subject |
|  | https://link.springer.com/article/10.1186/s12877-020-01777-4 | 2007 | A study on depression of the elderly with different sleep quality in pension institutions in Northeastern China | Setting |
|  | X. Zhong, Z. Wang and L. Zhang | 2010 | Study on health status and KAP of patients with diabetes mellitus in the urban communities in Anhui Province | Subject |
|  | C. Hu, W. Yu, Y. Lv, H. Chen, Q. Deng and L. Zhang | 2017 | Study on the health status and health service utilization of the elderly of a remote and poor village in a mountainous area in Jinzhai, Anhui | Subject |
|  | https://www.mdpi.com/1660-4601/14/4/408/htm | 2008 | Study on the health status and health service utilization of the elderly of a remote and poor village in a mountainous area in Jinzhai, Anhui | Setting |
|  | J. J. Li, J. L. Li, J. Zhang, R. R. Jin, S. Ma et al. | 2018 | [Study on the overall implementation status of the National Demonstration Areas for Comprehensive Prevention and Control of Non-communicable Diseases] | Subject |
|  | D. Guwatudde, P. Absetz, P. Delobelle, C.-G. Östenson, J. Olmen Van et al. | 2018 | Study protoco for the SMART2D adaptive implementation trial: a cluster randomised tial comparing facility-only care with integrated facility and community are to improve type 2 diabetes outcomes in Uganda, South Africa and Swede | Protocol |
|  | D. Guwatudde, P. Absetz, P. Delobelle, C. G. Östenson, J. Olmen Van et al. | 2018 | Study proocol for the SMART2D adaptive implementation trial: a cluster randomised trial comparing facility-only care with integrated facility and community care to improve type 2 diabetes outcomes in Uganda, South Africa and Sweden | Protocol |
|  | D. M. Kindarara | 2016 | Sub-Saharan African immigrants' health-illness transition experiences with Type 2 diabetes self-management in the United States | Population |
|  | W. Chen, C. Shiu, J. P. Yang, C. S. R. Li, K. Wang et al. | 2018 | Substance use, anxiety, and self-management efficacy in HIV-positive individuals: a mediation analysis | Population |
|  | https://www.tandfonline.com/loi/ijsu20 | 2019 |  | Subject |
|  | A. H. Heald, S. G. Anderson, A. Khan, J. Stocker, S. Davies et al. | 2017 | Success Rates in a Diabetes Specialist Nurse-Led Education Programme: Re-setting the Glucostat | Subject |
|  | P. Greenham | 2007 | Successful participation | Subject |
|  | Z. Jannoo and N. Mamode Khan | 2018 | Summary of Diabetes Self-care Activities: A confirmatory factor analytic approach | Subject |
|  | G. Martínez Pérez, S. J. Steele, I. Govender, G. Arellano, A. Mkwamba et al. | 2016 | Supervised oral HIV self-testing is accurate in rural KwaZulu-Natal, South Africa | Subject |
|  | G. Martínez Pérez, S. J. Steele, I. Govender, G. Arellano, A. Mkwamba et al. | 2016 | Supervised oral HIV self-testing is accurate in rural KwaZulu-Natal, South Africa | Subject |
|  | A. Sancho, S. Carrera, M. Arietaleanizbeascoa, V. Arce, N. M. Gallastegui at al. | 2015 | Supervised physical exercise to improve the quality of life of cancer patients: the EFICANCER randomised controlled trial | Subject |
|  | A. Sancho, S. Carrera, M. Arietaleanizbeascoa, V. Arce, N. Mendizabal Gallastegui et al. | 2015 | Supervised physical exercise to improve the quality of life of cancer patients: the EFICANCER randomised controlled trial | Subject |
|  | L. Perry, S. James, R. Gallagher, J. Dunbabin, K. Steinbeck and J. Lowe | 2017 | Supporting patients with type 1 diabetes using continuous subcutaneous insulin infusion therapy: Difficulties, disconnections, and disarray | Subject |
|  | H. Siltanen and V. Jylhä | 2019 | Supporting the supporter: a focus on families of patients with chronic obstructive pulmonary disease | Subject |
|  | W. J. Kalk, Y. Veriawa and C. Osler | 2000 | A survey of hospital outpatient services for chronic diseases in Gauteng | Subject |
|  | O. Esan, J. Appiah-Poku, C. Othieno, L. Kola, B. Harris et al. | 2019 | A survey of traditional and faith healers providing mental health care in three sub-Saharan African countries | Subject |
|  | L. Y. Ojewale, A. O. Oluwatosin, A. A. Fasanmade and O. Odusan | 2019 | A survey on patients' characteristics, perception of family support and diabetes self-management among type 2 diabetes patients in South-West Nigeria | Subject |
|  | L. Y. Ojewale, A. O. Oluwatosin, A. A. Fasanmade and O. Odusan | 2019 | A survey on patients' characteristics, perception of family support and diabetes self‐management among type 2 diabetes patients in South‐West Nigeria | Subject |
|  | F. Rasschaert, T. Decroo, D. Remartinez, B. Telfer, F. Lessitala, M. Biot et al. | 2014 | Sustainability of a community-based anti-retroviral care delivery model - a qualitative research study in Tete, Mozambique | Subject |
|  | K. Hjelm, K. Bard, P. Nyberg and J. Apelqvist | 2005 | Swedish and Middle-Eastern-born women's beliefs about gestational diabetes | Subject |
|  | K. Peltzer and N. Phaswana-Mafuya | 2008 | The symptom experience of people living with HIV and AIDS in the Eastern Cape, South Africa | Subject |
|  | K. Peltzer, N. Phaswana-Mafuya, K. Peltzer and N. Phaswana-Mafuya | 2008 | The symptom experience of people living with HIV and AIDS in the Eastern Cape, South Africa | Subject |
|  | C. Sri-Pathmanathan, D. Nhamo, T. Mamvuto, G. Chapwanya, F. Terris-Prestholt et al. | 2022 | Syphilis self-testing to expand test uptake among men WHO have sex with men: a theoretically informed mixed methods study in Zimbabwe | Subject |
|  | <https://sti.bmj.com/content/98/3/197> | 2020 | Syphilis self-testing to expand test uptake among men WHO have sex with men: a theoretically informed mixed methods study in Zimbabwe | Repetition |
|  | B. Dagnew, G. Debalkie Demissie and D. Abebaw Angaw | 2021 | Systematic Review and Meta-Analysis of Good Self-Care Practice among People Living with Type 2 Diabetes Mellitus in Ethiopia: A National Call to Bolster Lifestyle Changes | Review |
|  | V. Mogre, N. A. Johnson, F. Tzelepis, J. E. Shaw and C. Paul | 2021 | A systematic review of adherence to diabetes self-care behaviours: Evidence from low- and middle-income countries | Review |
|  | K. B. Nkhoma, A. Cook, A. Giusti, L. Farrant, R. Petrus et. al. | 2022 | A systematic review of impact of person-centred interventions for serious physical illness in terms of outcomes and costs | Review |
|  | K. B. Nkhoma, A. Cook, A. Giusti, L. Farrant, R. Petrus et al. | 2022 | A systematic review of impact of person-centred interventions for serious physical illness in terms of outcomes and costs | Review |
|  | T. N. M. Nguyen, L. Whitehead, R. Saunders and G. Dermody | 2022 | Systematic review of perception of barriers and facilitators to chronic disease self-management among older adults: Implications for evidence-based practice | Review |
|  | R. L. Zemek, S. K. Bhogal and F. M. Ducharme | 2008 | Systematic review of randomized controlled trials examining written action plans in children: what is the plan? | Review |
|  | D. A. Greenwood, P. M. Gee, K. J. Fatkin and M. Peeples | 2017 | A Systematic Review of Reviews Evaluating Technology-Enabled Diabetes Self-Management Education and Support | Review |
|  | Z. A. Nodjikouambaye, C. Adawaye, R.-S. Mboumba Bouassa, D. Sadjoli and L. Bélec | 2020 | A systematic review of self-sampling for HPV testing in Africa | Review |
|  | B. Kent, E. Cull and N. M. Phillips | 2011 | A systematic review of the effectiveness of current interventions to assist adults with heart failure to comply with therapy and enhance self-care behaviours | Review |
|  | C. J. Aantjes, L. Ramerman and J. F. G. Bunders | 2014 | A systematic review of the literature on self-management interventions and discussion of their potential relevance for people living with HIV in sub-Saharan Africa | Review |
|  | B. Ovbiagele | 2015 | Tackling the growing diabetes burden in Sub-Saharan Africa: a framework for enhancing outcomes in stroke patients | Subject |
|  | N. Angotti, S. A. Mojola, E. Schatz, J. R. Williams and F. X. Gómez-Olivé | 2018 | 'Taking care' in the age of AIDS: older rural South Africans' strategies for surviving the HIV epidemic | Subject |
|  | C. N. Lebron, E. Reyes-Arrechea, A. Castillo, O. Carrasquillo and S. Kenya | 2015 | Tales from the Miami Healthy Heart Initiative: The Experiences of Two Community Health Workers | Subject |
|  | K. Zewdie, C. Kiptinness, K. Ngure, N. Kipkurui, N. Wairimu et al. | 2022 | Targeted implementation of HIV self-testing increases testing uptake among partners of index persons known to have HIV in Kenya | Subject |
|  | K. Zewdie, C. Kiptinness, K. Ngure, N. Kipkurui, N. Wairimu et al. | 2020 | Targeted implementation of HIV self-testing increases testing uptake among partners of index persons known to have HIV in Kenya | Subject |
|  | K. Zewdie, C. Kiptinness, K. Ngure, N. Kipkurui, N. Wairimu et al. | 2013 | Tasks and strategies of self-management of living with antiretroviral therapy in Uganda | Invalid |
|  | <https://online.liebertpub.com/lia/vwc> | 2022 |  | Subject |
|  | P. Carswell | 2015 | Te Whiringa Ora: person-centred and integrated care in the Eastern Bay of Plenty, New Zealand | Subject |
|  | A. Banbury, S. Nancarrow, J. Dart, L. Gray and L. Parkinson | 2018 | Telehealth Interventions Delivering Home-based Support Group Videoconferencing: Systematic Review | Subject |
|  | C. K. Chow, S. M. S. Islam, A. Farmer, K. Bobrow, R. Maddision et al. | 2016 | Text2PreventCVD: protocol for a systematic review and individual participant data meta-analysis of text message-based interventions for the prevention of cardiovascular diseases | Protocol |
|  | L. Gavish and N. N. Houreld | 2019 | Therapeutic Efficacy of Home-Use Photobiomodulation Devices: A Systematic Literature Review | Protocol |
|  | J. E. Mantell, A. L. DiCarlo, R. H. Remien, A. Zerbe, D. Morris et al. | 2014 | 'There's no place like home': perceptions of home-based HIV testing in Lesotho | Subject |
|  | T. Copeland | 2018 | To keep this disease from killing you: cultural competence, consonance, and health among HIV-positive women in Kenya | Methods |
|  | P. Sadlon, D. Charron-Prochownik and S. Sullivan-Bolyai | 2020 | Together We Can Return to Balance-Eastern Woodlands Native Perspectives and Type 2 Diabetes: A Qualitative Study | Setting |
|  | P. Sadlon, D. Charron-Prochownik and S. Sullivan-Bolyai | 2020 | Together We Can Return to Balance—Eastern Woodlands Native Perspectives and Type 2 Diabetes: A Qualitative Study | Setting |
|  | P. Sadlon, D. Charron-Prochownik and S. Sullivan-Bolyai | 2020 | Together We Can Return to Balance—Eastern Woodlands Native Perspectives and Type 2 Diabetes: A Qualitative Study | Setting |
|  | J. A. Oketch, M. Paterson, E. W. Maunder and N. C. Rollins | 2011 | Too little, too late: comparison of nutritional status and quality of life of nutrition care and support recipient and non-recipients among HIV-positive adults in KwaZulu-Natal, South Africa | Subject |
|  | https://www.sciencedirect.com/science?_ob=ArticleURL&_udi= | 2013 |  | Invalid |
|  | M. M. Othman, H. Khudadad, R. Dughmosh, A. Syed, J. Clark et al. | 2021 | Towards a better understanding of self-management interventions in type 2 diabetes: A meta-regression analysis | Methods |
|  | N. Z. Nyazema | 1984 | Towards better patient drug compliance and comprehension: a challenge to medical and pharmaceutical services in Zimbabwe | Subject |
|  | L. Chetty, N. Govender and P. Reddy | 2022 | Traditional medicine use among type 2 diabetes patients in KZN | Methods |
|  | https://www.hindawi.com/journals/aph/2022/7334080/ | 2020 |  | Invalid |
|  | J. R. Zelnick, B. Seepamore, A. Daftary, K. R. Amico, X. Bhengu et al. | 2018 | Training social workers to enhance patient-centered care for drug-resistant TB-HIV in South Africa | Subject |
|  | <https://ingentaconnect.com/contentone/iuatld/pha/2018/00000XXX> | 2020 |  | Invalid |
|  | I. N. Njuguna, K. Beima-Sofie, C. W. Mburu, C. Mugo, J. Itindi et al. | 2022 | Transition to independent care for youth living with HIV: a cluster randomised clinical trial | Subject |
|  | G. Chepngeno-Langat and M. Evandrou | 2013 | Transitions in Caregiving and Health Dynamics of Caregivers for People With AIDS: A Prospective Study of Caregivers in Nairobi Slums, Kenya | Subject |
|  | A. McGlynn, C. O'Callaghan, B. McDougall, J. Osborne and B. Harris-Roxas | 2022 | Translating Health Coaching Training into Clinical Practice | Subject |
|  | S. A. Safren, C. O'Cleirigh, L. S. Andersen, J. F. Magidson, J. S. Lee et al. | 2021 | Treating depression and improving adherence in HIV care with task-shared cognitive behavioural therapy in Khayelitsha, South Africa: a randomized controlled trial | Subject |
|  | M. Hassen, D. Mekonnen and O. S. Muhammed | 2022 | Treatment burden among patients with heart failure attending cardiac clinic of Tikur Anbessa Specialized Hospital: an explanatory sequential mixed methods study | Subject |
|  | M. Hassen, D. Mekonnen and O. S. Muhammed | 2022 | Treatment burden among patients with heart failure attending cardiac clinic of Tikur Anbessa Specialized Hospital: an explanatory sequential mixed methods study | Subject |
|  | T. Shimels, M. Abebaw, A. I. Bilal and T. Tesfaye | 2018 | Treatment Pattern and Factors Associated with Blood Pressure and Fasting Plasma Glucose Control among Patients with Type 2 Diabetes Mellitus in Police Referral Hospital in Ethiopia | Subject |
|  | S. Tariku, A. Melesse, A. I. Bilal and T. Tariku | 2018 | Treatment pattern and factors associated with blood pressure and fasting plasma glucose control among patients with type 2 diabetes mellitus in police referral hospital in Ethiopia | Subject |
|  | K. O. Adu | 2022 | Treatment-seeking behaviour among persons with chronic diseases in Ghana: does national health insurance status matter? | Subject |
|  | K.O Adu | 2021 | Treatment-seeking behaviour among persons with chronic diseases in Ghana: does national health insurance status matter? | Invalid |
|  | J. Chuma, L. Gilson and C. Molyneux | 2007 | Treatment-seeking behaviour, cost burdens and coping strategies among rural and urban households in Coastal Kenya: an equity analysis | Subject |
|  | P. Farthing, J. Bally, D. C. Rennie, M. Dietrich Leurer, L. Holtslander and M. A. Nour | 2022 | Type 1 diabetes management responsibilities between adolescents with T1D and their parents: An integrative review | Subject |
|  | E. Elkon-Tamir, Y. Lebenthal, I. Laurian, A. Dorfman, E. Chorna, H. Interator et al. | 2021 | Type 1 diabetes outcomes of children born in Israel of Eritrean asylum seekers | Subject |
|  | R. T. Goins, J. Jones, M. Schure, B. Winchester and V. Bradley | 2020 | Type 2 diabetes management among older American Indians: beliefs, attitudes, and practices | Population |
|  | A. I. Okurumeh, O. A. Akpor, O. E. Okeya and O. B. Akpor | 2022 | Type 2 diabetes mellitus patients' lived experience at a tertiary hospital in Ekiti State, Nigeria | Included |
|  | L. Dube, S. Van den Broucke, M. Housiaux, W. Dhoore and K. Rendall-Mkosi | 2015 | Type 2 diabetes self-management education programs in high and low mortality developing countries: a systematic review | Review |
|  | Akyirem S, Ekpor E, Namumbejja Abwoye D, Batten J, Nelson LE. | 2023 | Type 2 diabetes stigma and its association with clinical, psychological, and behavioral outcomes: A systematic review and meta-analysis. Diabetes research and clinical practice. | Review |
|  | L. J. Leikin | 1990 | Type A behaviour and health locus of control: another view on Perloff et al. (1988) | Subject |
|  | J. Cover, A. Namagembe, J. Tumusiime, J. Lim and C. M. Cox | 2018 | Ugandan providers' views on the acceptability of contraceptive self-injection for adolescents: a qualitative study | Subject |
|  | G. B. Gebremichael, K. K. Berhe and T. M. Zemichael | 2019 | Uncontrolled hypertension and associated factors among adult hypertensive patients in Ayder comprehensive specialized hospital, Tigray, Ethiopia, 2018 | Methods |
|  | G. Gebrewahd Bezabh, B. Kalayou Kidanu and Z. Teklewoini Mariye | 2019 | Uncontrolled hypertension and associated factors among adult hypertensive patients in Ayder comprehensive specialized hospital, Tigray, Ethiopia, 2018 | Methods |
|  | L. Abdisa, S. Girma, M. Lami, A. Hiko, E. Yadeta et al. | 2022 | Uncontrolled hypertension and associated factors among adult hypertensive patients on follow-up at public hospitals, Eastern Ethiopia: A multicenter study | Methods |
|  | D. E. McMahon, L. Chemtai, M. Grant, R. Singh, A. Semeere et al. | 2022 | Understanding diagnostic delays for Kaposi sarcoma in Kenya: a qualitative study | Subject |
|  | D. E. McMahon, L. Chemtai, M. Grant, R. Singh, A. Semeere et al. | 2022 | Understanding diagnostic delays for Kaposi sarcoma in Kenya: a qualitative study | Subject |
|  | D. E. McMahon, L. Chemtai, M. Grant, R. Singh, A. Semeere et al. | 2020 | Understanding engagement in HIV programmes: how health services can adapt to ensure no one is left behind | Subject |
|  | https://link.springer.com/article/10.1007/s11904-020-00522-1 | 2000 | Understanding engagement in HIV programmes: how health services can adapt to ensure no one is left behind | Invalid |
|  | A. Grimsrud, L. Wilkinson, I. Eshun-Wilson, C. Holmes, I. Sikazwe and I. T. Katz | 2020 | Understanding Engagement in HIV Programmes: How Health Services Can Adapt to Ensure No One Is Left Behind | Subject |
|  | D. Gumede, A. Meyer-Weitz, A. Edwards and J. Seeley | 2022 | Understanding older peoples' chronic disease self-management practices and challenges in the context of grandchildren caregiving: a qualitative study in rural KwaZulu-Natal, South Africa | Population |
|  | <https://journals.plos.org/globalpublichealth/article?id=17.1371/journal.pgph.000089587> | 2022 |  | Invalid |
|  | F. M. Siad, X. Y. Fang, M. J. Santana, S. Butalia, M. A. Hebert and D. M. Rabi | 2018 | Understanding the Experiences of East African Immigrant Women With Gestational Diabetes Mellitus | Subject |
|  | A.-L. Byrne, A. Baldwin, C. Harvey, J. Brown, E. Willis et al. | 2021 | Understanding the impact and causes of 'failure to attend' on continuity of care for patients with chronic conditions | Subject |
|  | T. Palmer, C. Waliaula, G. Shannon, F. Salustri, G. Grewal et al. | 2022 | Understanding the Lived Experience of Children With Type 1 Diabetes in Kenya: Daily Routines and Adaptation Over Time | Population |
|  | F. F. Teng, S. M. Mitchell, M. Sekikubo, C. Biryabarema, J. K. Byamugisha et al. | 2014 | Understanding the role of embarrassment in gynaecological screening: a qualitative study from the ASPIRE cervical cancer screening project in Uganda | Subject |
|  | F. F. Teng, S. M. Mitchell, M. Sekikubo, C. Biryabarema, J. K. Byamugisha et al. | 2014 | Understanding the role of embarrassment in gynaecological screening: a qualitative study from the ASPIRE cervical cancer screening project in Uganda | Subject |
|  | J. O. Edah, S. G. Goar, G. Odoh, B. Lawal, P. S. Dayom et al. | 2020 | Undiagnosed depression among adults with diabetes mellitus in Jos | Subject |
|  | J. M. Brion, C. Dawson Rose, P. K. Nicholas, R. Sloane, J. G. Voss et al. | 2011 | Unhealthy substance-use behaviors as symptom-related self-care in persons with HIV/AIDS | Subject |
|  | M. Jessri, P. Sadighi, N. Toofani, L. Woods and M. L'Abbe | 2015 | Unheard voices of Iranian immigrants with type 2 diabetes: a Canadian focused ethnographic study | Subject |
|  | S. A. McMahon, D. K. Musoke, J. Wachinger, A. Nakitende, J. Amongin et al. | 2021 | Unintended uses, meanings, and consequences: HIV self-testing among female sex workers in urban Uganda | Setting |
|  | A. Hoffman | 2010 | Universal principles for culturally sensitive diabetic education | Subject |
|  | L. Pan, C. Wang, X. Cao, H. Zhu and L. Luo | 2022 | Unmet healthcare needs and their determining factors among unwell migrants: a comparative study in Shanghai | Subject |
|  | <https://www.mdpi.com/1760-4871/19/9/54999> | 2019 |  | Invalid |
|  | P. M. Mugo, M. Micheni, J. Shangala, M. H. Hussein, S. M. Graham et al. | 2017 | Uptake and Acceptability of Oral HIV Self-Testing among Community Pharmacy Clients in Kenya: A Feasibility Study | Subject |
|  | W. Tun, L. Vu, O. Dirisu, A. Sekoni, E. Shoyemi, J. Njab, S. Ogunsola and S. Adebajo | 2018 | Uptake of HIV self-testing and linkage to treatment among men who have sex with men (MSM) in Nigeria: A pilot programme using key opinion leaders to reach MSM | Subject |
|  | C. C. Iwuji, J. Orne-Gliemann, J. Larmarange, N. Okesola, F. Tanser et al. | 2016 | Uptake of Home-Based HIV Testing, Linkage to Care, and Community Attitudes about ART in Rural KwaZulu-Natal, South Africa: Descriptive Results from the First Phase of the ANRS 12249 TasP Cluster-Randomised Trial | Subject |
|  | C. C. Iwuji, J. Orne-Gliemann, J. Larmarange, N. Okesola, F. Tanser et al. | 2016 | Uptake of Home-Based HIV Testing, Linkage to Care, and Community Attitudes about ART in Rural KwaZulu-Natal, South Africa: Descriptive Results from the First Phase of the ANRS 12249 TasP Cluster-Randomised Trial | Subject |
|  | C. C. Iwuji, J. Orne-Gliemann, J. Larmarange, N. Okesola, F. Tanser et al. | 2016 | Uptake of Home-Based HIV Testing, Linkage to Care, and Community Attitudes about ART in Rural KwaZulu-Natal, South Africa: Descriptive Results from the First Phase of the ANRS 12249 TasP Cluster-Randomised Trial | Subject |
|  | A. E. Fischer, M. Phatsoane, M. Majam, L. Shankland, M. Abrahams et al. | 2021 | Uptake of the Ithaka mobile application in Johannesburg, South Africa, for human immunodeficiency virus self-testing result reporting | Subject |
|  | https://sajhivmed.org.za/index.php/hivmed/article/view/1197 | 2018 | Uptake of the Ithaka mobile application in Johannesburg, South Africa, for human immunodeficiency virus self-testing result reporting | Subject |
|  | A. T. Choko, P. MacPherson, E. L. Webb, B. A. Willey, H. Feasy et al. | 2015 | Uptake, Accuracy, Safety, and Linkage into Care over Two Years of Promoting Annual Self-Testing for HIV in Blantyre, Malawi: A Community-Based Prospective Study | Subject |
|  | Z. Zheng, H. Yan, G. Mengdi and A. B. Williams | 2019 | Urban and Rural Differences: Unmet Needs for Symptom Management in People Living With HIV in China | Repetition |
|  | Z. Zhu, Y. Hu, M. Guo and A. B. Williams | 2019 | Urban and Rural Differences: Unmet Needs for Symptom Management in People Living With HIV in China | Repetition |
|  | S. M. Lacourse, D. Leon, N. Panpradist, B. A. Richardson, E. Maleche-Obimbo et al. | 2021 | Urine biomarker assessment of infant adherence to isoniazid prophylaxis | Subject |
|  | <https://journals.lww.com/pidj/Fulltext/2021/01000/Urine_Biomarker_Assessment_of_Infant_Adherence_to.29.aspx> | 2014 | Urine biomarker assessment of infant adherence to isoniazid prophylaxis | Subject |
|  | A. J. Shelley, K. A. McDonald, A. McEvoy, M. Sauder, N. Kanigsberg et al. | 2018 | Usability, Satisfaction, and Usefulness of an Illustrated Eczema Action Plan | Subject |
|  | C. Johnson, M. Neuman, P. MacPherson, A. Choko, C. Quinn et al. | 2020 | Use and awareness of and willingness to self-test for HIV: an analysis of cross-sectional population-based surveys in Malawi and Zimbabwe | Subject |
|  | D. Serwadda, M. J. Wawer, K. V. Shah, N. K. Sewankambo, R. Daniel et al. | 1999 | Use of a hybrid capture assay of self-collected vaginal swabs in rural Uganda for detection of human papillomavirus | Subject |
|  | A. H. Vallerand, J. M. Fouladbakhsh and T. Templin | 2003 | The use of complementary/alternative medicine therapies for the self-treatment of pain among residents of urban, suburban, and rural communities | Subject |
|  | O. Flores, Z. Tyack, K. Stockton and J. D. Paratz | 2020 | The use of exercise in burns rehabilitation: A worldwide survey of practice | Subject |
|  | A. DiCarlo, A. Zerbe, Z. J. Peters, K. Frederix, J. P. Nkonyana et al. | 2017 | Use of Index Patients to Enable Home-Based Testing in Lesotho | Subject |
|  | H. N. Obilor, M. Achore and K. Woo | 2022 | Use of Information Communication Technology Tools in Diabetic Foot Ulcer Prevention Programs: A Scoping Review | Subject |
|  | T. Gedif and H.-J. Hahn | 2003 | The use of medicinal plants in self-care in rural central Ethiopia | Subject |
|  | T. Gedif and H. J. Hahn | 2003 | The use of medicinal plants in self-care in rural central Ethiopia | Repetition |
|  | A. C. Allabi, K. Busia, V. Ekanmian and F. Bakiono | 2011 | The use of medicinal plants in self-care in the Agonlin region of Benin | Subject |
|  | <https://www.sciencedirect.com/science?_ob=ArticleURL> | 2023 |  | Invalid |
|  | K. A. Korsah | 2023 | The Use of Religious Capital as a Coping Strategy in Self-care by Type 2 Diabetes Patients in a Ghanaian Hospital | Population |
|  | E. O. Owolabi and D. T. Goon | 2019 | The use of text messaging for improving adherence to anti-diabetic regimen and glycaemic control in low-resource settings of South Africa: A study protocol for a randomised controlled trial | Subject |
|  | C. Bamuya, J. C. Correia, E. M. Brady, D. Beran, D. Harrington et al. | 2021 | Use of the socio-ecological model to explore factors that influence the implementation of a diabetes structured education programme (EXTEND project) inLilongwe, Malawi and Maputo, Mozambique: a qualitative study | Subject |
|  | E. Juré, J. Iguenane, A. Toudonou, A. Azondekon and R. Gagnayre | 2010 | [The usefulness of a genogram as a tool in therapeutic patient education: an exploratory study of parents of children living with HIV/AIDS in Benin] | Subject |
|  | U. Fors, J. T. Kamwesiga, G. M. Eriksson, L. von Koch and S. Guidetti | 2019 | User evaluation of a novel SMS-based reminder system for supporting post-stroke rehabilitation | Subject |
|  | I. K. Ledel Solem, C. Varsi, H. Eide, O. B. Kristjansdottir, E. Børøsund et al. | 2020 | A User-Centered Approach to an Evidence-Based Electronic Health Pain Management Intervention for People With Chronic Pain: Design and Development of EPIO | Subject |
|  | J. K. Gona, C. R. Newton, J. Geere and S. Hartley | 2013 | Users' experiences of physiotherapy treatment in a semi-urban public hospital in Kenya | Subject |
|  | J. v. Olmen, P. Delobelle, D. Guwatudde, P. Absetz, D. Sanders et al. | 2018 | Using a cross-contextual reciprocal learning approach in a multisite implementation research project to improve self-management for type 2 diabetes | Subject |
|  | <https://gh.bmj.com/content/3/6/e001068> | 2012 |  | Invalid |
|  | J. van Olmen, P. Delobelle, D. Guwatudde, P. Absetz et al. | 2018 | Using a cross-contextual reciprocal learning approach in a multisite implementation research project to improve self-management for type 2 diabetes | Subject |
|  | M. Spires, P. Delobelle, D. Sanders and T. Puoane | 2020 | Using photography to explore people with diabetes' perspectives on food environments in urban and rural South Africa | Subject |
|  | <https://academic.oup.com/heapro/article-abstract/36/1/120/5828359?> | 2020 |  | Invalid |
|  | M. Spires, P. Delobelle, D. Sanders and T. Puoane | 2020 | Using photography to explore people with diabetes' perspectives on food environments in urban and rural South Africa | Subject |
|  | H. S. Banks, T. Girmay, W. Moges, T. Abreham, G. Davey and M. Cooper | 2016 | Using qualitative methods to explore lay explanatory models, health-seeking behaviours and self-care practices of podoconiosis patients in north-west Ethiopia | Subject |
|  | <https://journals.plos.org/plosntds/article?id=10.18u771/journal.pntd.0004878> | 2021 |  | Invalid |
|  | K. Bobrow, A. Farmer, N. Cishe, N. Nwagi, M. Namane et al. | 2018 | Using the Medical Research Council framework for development and evaluation of complex interventions in a low resource setting to develop a theory-based treatment support intervention delivered via SMS text message to improve blood pressure control | Subject |
|  | A. E. Johnson, M. Yin and G. Berg | 2003 | Utilization and financial outcomes of an asthma disease management program delivered to Medicaid members: results of a three-group comparison study | Subject |
|  | A. Develay, R. Sauerborn and H. J. Diesfeld | 1996 | Utilization of health care in an African urban area: results from a household survey in Ouagadougou, Burkina-Faso | Subject |
|  | K. Peltzer, S. Pengpid, A. Puckpinyo, S. Yi and L. V. Anh | 2016 | The utilization of traditional, complementary and alternative medicine for non-communicable diseases and mental disorders in health care patients in Cambodia, Thailand and Vietnam | Subject |
|  | H. Maimaiti, J. Lu, X. Guo, L. Zhou, L. Hu and Y. Lu | 2022 | Vaccine uptake to prevent meningitis and encephalitis in Shanghai, China | Subject |
|  | https://www.mdpi.com/2076-393X/10/12/2054 | 2012 | Vaccine uptake to prevent meningitis and encephalitis in Shanghai, China | Invalid |
|  | S. C. Francis, S. S. Lees, B. Andrew, F. Zalwango, J. Vandepitte et a. | 2012 | Vaginal practices diary: development of a pictorial data collection tool for sensitive behavioral data | Subject |
|  | S. C. Francis, S. S. Lees, B. Andrew, F. Zalwango, J. Vandepitte et al. | 2012 | Vaginal practices diary: development of a pictorial data collection tool for sensitive behavioral data | Subject |
|  | M. El Tantawi, M. O. Folayan, A. L. Nguyen, N. M. Aly, O. Ezechi et al. | 2022 | Validation of a COVID-19 mental health and wellness survey questionnaire | Subject |
|  | B. O. A. Adegoke, A. C. Odole, A. T. Adekunle-Balogun and I. Umar | 2016 | Validation of a Yoruba version of the arthritis self-efficacy scale | Subject |
|  | P. A. Muwanguzi, E. M. Nasuuna, F. Namimbi, C. P. Osingada and T. D. Ngabirano | 2021 | Venues and methods to improve professional men's access to HIV self-testing and linkage to HIV prevention or treatment: a qualitative study | Subject |
|  | <https://link.springer.com/article/10.1876/s12913-021-07259-f5> |  |  | Invalid |
|  | S. Serfontein and R. J. Mash | 2013 | Views of patients on a group diabetes education programme using motivational interviewing in South African primary care: a qualitative study | Subject |
|  | M. A. Seid, A. Ambelu, M. Diress, Y. Yeshaw, Y. Akalu and B. Dagnew | 2022 | Visual impairment and its predictors among people living with type 2 diabetes mellitus at Dessie town hospitals, Northeast Ethiopia: institution-based cross-sectional study | Subject |
|  | M. A. Seid, A. Ambelu, M. Diress, Y. Yeshaw, Y. Akalu and B. Dagnew | 2022 | Visual impairment and its predictors among people living with type 2 diabetes mellitus at Dessie town hospitals, Northeast Ethiopia: institution-based cross-sectional study | Subject |
|  | Ukoha-Kalu BO, Adibe MO, Ukwe CV. | 2023 | A qualitative study of patients' and carers' perspectives on factors influencing access to hypertension care and compliance with treatment in Nigeria | Included |
|  | S. C. Thomsen, D. Skinner, Y. Toefy, T. Esterhuizen, M. McCaul, M. Petzold and V. Diwan | 2016 | Voice-Message-Based mHealth Intervention to Reduce Postoperative Penetrative Sex in Recipients of Voluntary Medical Male Circumcision in the Western Cape, South Africa: Protocol of a Randomized Controlled Trial | Subject |
|  | C. J. Roberts | 2017 | We can all live extraordinary lives--my journey has been remarkable | Subject |
|  | A. R. Davidson, J. Kelly, L. Ball, M. Morgan and D. P. Reidlinger | 2022 | What do patients experience? Interprofessional collaborative practice for chronic conditions in primary care: an integrative review | Subject |
|  | E. Mendenhall, A. Musau, E. Bosire, V. Mutiso, D. Ndetei and M. Rock | 2020 | What drives distress? Rethinking the roles of emotion and diagnosis among people with diabetes in Nairobi, Kenya | Subject |
|  | V. Prakash and M. Ganesan | 2021 | What matters to patients with stroke in India and why: a qualitative study | Subject |
|  | R. B. Peck, J. M. Lim, H. van Rooyen, W. Mukoma, L. Chepuka et al. | 2014 | What should the ideal HIV self-test look like? A usability study of test prototypes in unsupervised HIV self-testing in Kenya, Malawi, and South Africa | Subject |
|  | J. Hanass-Hancock, H. Myezwa, S. A. Nixon and A. Gibbs | 2015 | “ When I was no longer able to see and walk, that is when I was affected most” : experiences of disability in people living with HIV in South Africa | Subject |
|  | M. Dattalo, E. R. Giovannetti, D. Scharfstein, C. Boult, S. Wegener et al. | 2012 | Who participates in chronic disease self-management (CDSM) programs? Differences between participants and nonparticipants in a population of multimorbid older adults | Methods |
|  | J. Rozanova, R. Marcus, F. S. Taxman, M. J. Bojko, L. Madden et al. | 2017 | Why People Who Inject Drugs Voluntarily Transition Off Methadone in Ukraine | Subject |
|  | J. Sewell and T. Moyo | 2019 | Winston Churchill Travelling Fellowship -- travel to learn, return to inspire...21st National HIV Nurses Association Annual Conference, 27-28 June 2019, Manchester Conference Centre, Manchester, UK | Subject |
|  | T. R. Mathunjwa and F. A. Gary | 2006 | Women and HIV/AIDS in the kingdom of Swaziland: culture and risks | Subject |
|  | C. Wanyama | 2002 | Women see little hope of anti-retroviral treatment yet | Subject |
|  | K. Mills, M. L. Gatton, R. Mahoney and A. Nelson | 2017 | 'Work it out': evaluation of a chronic condition self-management program for urban Aboriginal and Torres Strait Islander people, with or at risk of cardiovascular disease | Subject |
|  | W. S. Shaw, R. K. McLellan, E. Besen, S. Namazi, M. K. Nicholas et al. | 2022 | A Worksite Self-management Program for Workers with Chronic Health Conditions Improves Worker Engagement and Retention, but not Workplace Function | Subject |
|  | M. J. Hirschfeld | 1987 | The World Health Organization's regions of the Eastern Mediterranean and Europe: ageing of the population and nursing care | Subject |
|  | M. West, S. Sadler, J. Charles, F. Hawke, S. Lanting et al. | 2022 | Yarning about foot care: evaluation of a foot care service for Aboriginal and Torres Strait Islander Peoples | Subject |
|  | Z. Ahmed | 2011 | Youth at the nexus: ideology in HIV prevention in Nairobi, Kenya | Subject |
|  | K. Hjelm and E. Mufunda | 2010 | Zimbabwean diabetics' beliefs about health and illness: an interview study | Subject |
|  | P. Bleah, R. Wilson, D. Macdonald and P. Camargo Plazas | 2023 | “When I Don’t Have Money, I Don’t Eat”: A Critical Hermeneutic Study of Diabetes in Liberia | Included |
|  | P. Bleah, R. Wilson, D. Macdonald and P. Camargo-Plazas | 2023 | ‘The solution is we need to have a centre’: a study on diabetes in Liberia | Included |
|  | S. Tyabazeka, W. Phiri and R. R. Marie Modeste | 2024 | HIV self-management perceptions and experiences of students at one university in South Africa | Included |
|  | E. Kim, P. K. Ndege, E. Jackson, D. J. Clauw and V. L. Ellingrod | 2019 | Patient perspectives on medication self-management in rural Kenya: a cross-sectional survey | Methods |
|  |  | 2025 | Are children and adolescents living with HIV in Europe and South Africa at higher risk of SARS-CoV-2 and poor COVID-19 outcomes? | Population |
|  | T. Abadi; T. Teklu; T. Wondmagegn; M. Alem; G. Desalegn | 2025 | Helicobacter pylori infection and associated risk factors among HIV-positive and HIV-negative individuals in Northern Ethiopia | Subject |
|  | G. Abate; E. Amentie; A. A. Abdulahi; S. Nigussie | 2024 | Patterns, treatment outcome and associated factors of surgically treated thyroid disease at public hospitals in Eastern Ethiopia: a retrospective cross-sectional study | Subject |
|  | M. Abayneh; Y. Habtemariam; T. Duguma; M. Abera | 2024 | Prevalence of intestinal parasites and associated factors among patients with HIV/AIDS at the anti-retroviral treatment clinic of Mizan-Tepi University Teaching Hospital, Southwest Ethiopia | Subject |
|  | R. Abbott; K. Landsiedel; M. Atukunda; S. B. Puryear; G. Chamie et al. | 2025 | Incident Tuberculosis Infection Is Associated With Alcohol Use in Adults in Rural Uganda | Subject |
|  | S. Abdelmenan; M. Demissie; E. Wujira; S. Tsegaye; H. Gulema et al. | 2024 | Prevalence of Self-Reported Chronic Non-Communicable Diseases among Adults in Addis Health and Demographic Surveillance System (Addis-HDSS), Addis Ababa, Ethiopia | Subject |
|  | A. A. Abdi; N. Louis; A. M. Hirsi; I. A. Nur; M. H. Mohamud; W. R. Ali; N. K. Imtiaz | 2024 | Patterns and factors associated with electrolyte abnormalities among patients with heart failure in Uganda | Subject |
|  | R. Abdulai; E. Phalane; K. Atuahene; R. N. Phaswana-Mafuya | 2024 | Consistent and Correct Use of Condoms With Lubricants and Associated Factors Among Men Who Have Sex With Men from the Ghana Men's Study II: Protocol for a Mixed Methods Study | Subject |
|  | Z. Abdulkadir; Y. O. Sule; A. Shuaibu; A. M. Abiso; F. M. Damagum et al. | 2024 | Effect of fasting on cardiovascular risk factors among healthy adult Muslims attending primary care, Kano, Northern Nigerian | Subject |
|  | H. Abdullahi; A. Tola; MaledaTefera | 2024 | Quality of life and associated factors among patients with epilepsy at selected public hospitals of Somali region, Eastern Ethiopia | Subject |
|  | K. B. Abebe | 2025 | Predictors associated with time to default for HIV/AIDS patients under HAART at Debre Tabor Referral Hospital: a Cox regression model | Subject |
|  | M. Abebe; Y. S. Asgedom; A. Y. Gebrekidan; Y. A. Wondimagegne; H. E. Hareru; T. M. Tebeje | 2025 | Factors associated with HIV testing among young women in Tanzania: Insights from the 2022 Tanzanian Demographic and Health Survey using Anderson's Behavioral Model | Subject |
|  | A. Abera; E. H. Fenta; B. T. Woldehanna; F. B. Wolde; M. Legesse et al. | 2024 | Impact of COVID-19 on essential healthcare services in Addis Ababa, Ethiopia: Implications for future pandemics | Subject |
|  | E. G. Abera; K. N. Tukeni; T. K. Chala; D. Yilma; E. K. Gudina | 2024 | Clinical profiles and mortality predictors of hospitalized patients with COVID-19 in Ethiopia | Subject |
|  | S. Abgrall; H. Selinger-Leneman; E. Lanoy; A. Becker; S. Matheron et al. | 2025 | Viral rebound on antiretroviral therapy in France according to region of origin, sex, and HIV acquisition group. Results from the French Hospital Database on HIV (ANRS CO4-FHDH) | Subject |
|  | C. A. Abidha; K. A. C. Meeks; F. P. Chilunga; A. Venema; R. Schindlmayr et al. | 2024 | A comprehensive lifestyle index and its associations with DNA methylation and type 2 diabetes among Ghanaian adults: the rodam study | Subject |
|  | O. O. Abiodun; T. Anya; V. T. Adekanmbi; D. Ojji | 2024 | Family History of Hypertension and Echocardiographic Left Ventricular Hypertrophy in Hypertensive Nigerians | Subject |
|  | D. Addisu; Y. Mitiku; W. Yazie Ferede; B. Y. Mekuriaw; B. B. Erega et al. | 2025 | Determinants of new-onset postpartum preeclampsia among mothers who delivered in hospitals in the South Gondar Zone, Northwest Ethiopia: a multicenter case-control study | Subject |
|  | D. Adei; W. Agyemang-Duah; B. O. Boateng; A. A. Mensah | 2025 | Predictors of food security status among informal caregivers of older adults residing in slums in Ghana | Subject |
|  | B. A. Adelekan; T. Femi-Adebayo; B. I. Adebayo; E. O. Somefun; A. Haruna; B. F. Popoola; F. O. Adepoju; M. U. Samuel; O. T. Akinyemi; O. Fisher et al. | 2025 | Sexual and reproductive health needs and barriers among youth living with HIV/AIDS in Lagos State, Nigeria | Subject |
|  | K. K. Adeleye; E. O. Owolabi; O. V. Adeniyi; D. A. Okunlola; A. I. Ajayi | 2024 | Relationship between social capital and post-partum antiretroviral therapy adherence among women living with HIV in the Eastern Cape, South Africa | Subject |
|  | A. B. Ademoyegun; A. G. Ibitoye; W. A. Rasaq; O. A. Adeniyi; D. O. Fabuluje et al. | 2025 | Eating difficulties among Nigerian community-dwelling stroke survivors: prevalence, correlates, and association with quality of life | Subject |
|  | M. Adeoye; H. Hamdallah; A. M. Adeoye | 2025 | Homocysteine levels and cardiovascular disease risk factors in chronic kidney disease (CKD), hypertensive and healthy Nigerian adults: a comparative retrospective study | Subject |
|  | V. A. Adepoju; D. C. Udah; Q. E. S. Adnani | 2024 | Prevalence, Risk Factors, and Clinical Profiles of Hepatitis D Virus in Nigeria: A Systematic Review, 2009-2024 | Subject |
|  | V. A. Adepoju; D. C. Udah; C. A. Ezenwa; J. Ganiyu; S. M. Lawal et al. | 2024 | Toward Universal Health Coverage: What Socioeconomic and Clinical Factors Influence Health Insurance Coverage and Restrictions in Access to Viral Hepatitis Services in Nasarawa State, Nigeria? | Subject |
|  | O. S. Adewuyi; M. S. Balogun; H. Otomaru; A. l. Abimiku; A. A. Ahumibe et al. | 2025 | Molecular Epidemiology, Drug-Resistant Variants, and Therapeutic Implications of Hepatitis B Virus and Hepatitis D Virus Prevalence in Nigeria: A National Study | Subject |
|  | W. Adraro; G. Abeshu; F. Abamecha | 2024 | Physical and psychological impact of HIV/AIDS toward youths in Southwest Ethiopia: a phenomenological study | Subject |
|  | R. Adu-Gyamfi; S. A. Addo; N. A. Baddoo; E. Kenu; A. Ashinyo et al. | 2025 | HIV retesting prevalence among clients accessing anti-retroviral therapy and HIV testing services in Ghana | Subject |
|  | M. Adugna; K. Asmare; A. Wondim | 2025 | Meconium aspiration syndrome and associated factors among neonates admitted at neonatal intensive care unit at Northwest Ethiopia comprehensive specialized hospitals Northwest Ethiopia 2023 | Subject |
|  | A. D. Afenigus; B. Kassahun; M. Dessalegn; A. Getnet; M. Gedfew; T. Kebede | 2024 | Prostatectomy outcomes for patients with benign prostatic hyperplasia and its associated factors in East and West Gojjam zones comprehensive specialized hospitals, Northwest Ethiopia | Subject |
|  | T. O. Afolaranmi; B. Chaplin; A. I. Zoakah; P. J. Kanki | 2025 | HIV-related stigma among young men who have sex with men in HIV care in Plateau State Nigeria | Subject |
|  | O. Agede; O. Daramola; A. Joseph; M. Jimoh; S. Ibrahim et al. | 2025 | Quality of hypertension management and health insurance impact: an assessment of insured and uninsured patients with systemic hypertension in a teaching Hospital in Ilorin, Nigeria | Subject |
|  | K. Agot; N. Okeyo; J. Onyango; M. Ochillo; G.-N. Wango et al. | 2025 | Jitegemee (rely on yourself): a cross-sectional study on acceptability, feasibility and design considerations for a personal savings intervention to reduce HIV risk among female sex workers in Siaya County, Kenya | Subject |
|  | K. Agot; J. Onyango; M. Ochillo; T. O. Okello; S. Carol et al. | 2024 | Jitegemee (rely on yourself): a multi-phase process of co-creating a personal savings intervention with female sex workers in western Kenya to reduce their HIV risk | Subject |
|  | N. A. F. Agyapong; R. A. Annan; F. Kroll; C. Apprey; L. N. E. Aduku; R. Aidoo; E. C. Swart | 2024 | Food acquisition and consumption by NOVA food classification and lived poverty index among rural and urban households in South Africa and Ghana | Subject |
|  | A. M. Ahmed; A. L. Sisay; M. N. Gebre | 2025 | Incidence and predictors of loss to follow-up among adult HIV patients attending antiretroviral therapy at public health facilities in Agaro town, Southwest Ethiopia, 2023 | Subject |
|  | D. Aigbonoga; B. Adewale; J. Igwilo; V. Adeyeye; T. Olajide et al. | 2025 | Efficacy of short message service (SMS) intervention on medication adherence and knowledge of stroke prevention among clinic attendees at risk of stroke: a randomized controlled trial | Subject |
|  | B. Ainembabazi; R. N. Ssebunya; W. Akobye; A. Mugume; P. Nahirya-Ntege et al. | 2024 | Viral load suppression and retention in care among children and adolescents receiving multi-month anti-retroviral therapy refills: a program data review in Uganda | Population |
|  | P. Ainembabazi; A. M. Gwokyalya; N. Twinamasiko; R. B. Kihumuro; T. M. Kintu; F. Bongomin | 2024 | Assessment of the readiness of health facilities in urban areas to deliver geriatric-friendly care services: a cross-sectional study in Kampala City, Uganda | Subject |
|  | F. A. Akoto; A.-S. Yakubu; F. Agyekum; A. Doku; J. A. Akamah | 2024 | Nocturnal blood pressure dipping and left ventricular hypertrophy among hypertensive outpatients in a Ghanaian hospital | Subject |
|  | K. A. Alatishe; O. K. Idowu; M. Alimi | 2024 | Allograft bone banking experience in Nigeria: a review of first 2 years | Subject |
|  | J. B. Alege; J. P. Oyore; R. C. Nanyonga; P. Musoke; A. S. S. Orago | 2025 | Barriers and facilitators of integrated hepatitis B, C, and HIV screening among pregnant mothers and newborns attending maternal and newborn clinics in Koboko District, Uganda: a qualitative inquiry of providers' perspective | Subject |
|  | S. Alem; H. Gulema | 2024 | Intention to use short messaging services for promoting drug adherence among individuals with diabetes in Addis Ababa, Ethiopia | Subject |
|  | T. T. Alemayehu; Y. A. Wassie; A. F. Bekalu; A. A. Tegegne; W. Ayenew et al. | 2024 | Prevalence of potential drug‒drug interactions and associated factors among elderly patients in Ethiopia: a systematic review and meta-analysis | Subject |
|  | C. Alemu; H. Wudu; B. Bogale; Z. Getachew; A. Nega | 2024 | Time to death and its determinant factors of stroke patients at Gambella General Hospital, Gambella, Ethiopia | Subject |
|  | G. S. Alemu; S. B. Mengistu; Y. Gedamu; E. Ayele; H. A. Admas et al. | 2025 | Dose optimization of non-selective beta blockers among esophageal varices patients at University of Gondar Comprehensive Specialized Hospital, Northwest Ethiopia: a hospital-based cross-sectional study | Subject |
|  | S. Y. Ali; M. I. Sadik; A. M. Seid; A. H. Mohammed; A. A. Yimam et al. | 2025 | Association of systemic immune inflammation index with depression among adult type 2 diabetic patients in a tertiary hospital, Ethiopia, 2022 | Subject |
|  | C. J. Aliche; E. S. Idemudia | 2024 | Diabetes Distress and Health-Related Quality of Life among Patients with Type 2 Diabetes-Mediating Role of Experiential Avoidance and Moderating Role of Post-Traumatic Growth | Subject |
|  | R. Alinaitwe; N. Nakasujja; H. Birabwa-Oketcho; A. Dickens; F. van Loggerenberg et al. | 2024 | Acceptability of a resource-oriented approach (DIALOG+) among patients with chronic physical illnesses in primary health care-Uganda, a qualitative study | Subject |
|  | M. S. Aloni | 2025 | Drivers of HIV self-test kit among Tanzanian men aged 15-49: findings from the 2022 TDHS-MIS cross-sectional study | Subject |
|  | S. K. Alor; I. A. Kretchy; F. N. Glozah; P. B. Adongo | 2024 | Community beliefs and practices about diabetes and their implications for the prevention and management of diabetes in Southeast Ghana | Included |
|  | A. R. Aluisio; S. J. Bergam; J. Kinuthia; J. W. Maina; S. Pirirei et al. | 2025 | HIV testing services and HIV self-testing programming within emergency care in Kenya: a qualitative study of healthcare personnel to inform enhanced service delivery approaches | Subject |
|  | N. Amedzro; D. Anaseba; A. G. Darkwa; A. Twumasi; A. Ayim et al. | 2024 | Uncovering the determinants of health in deprived urban neighborhoods in Accra, Ghana: a qualitative and participatory reconnaissance study | Subject |
|  | E. A. Amegashie; C. K. Kwayisi-Darkwah; M. Adusei-Poku; R. O. Sikeola; L. A. E. Ativi et al. | 2025 | Lipid Peroxidation and Glutathione Levels Among People Living With HIV Co-infected With Human Coronaviruses in Ghana | Subject |
|  | A. F. Amhare; G. G. Goyomsa; Y. M. A. Al Issa | 2024 | Investigating the follow-up discontinuation among people living with HIV in North Shoa Zone, Oromia, Ethiopia | Subject |
|  | A. F. Amhare; M. Shen; Z. Zou; R. Xia; J. Han; L. Gao; L. Zhang | 2024 | Assessing the impact of COVID-19 on HIV care cascade for people living with HIV in Ethiopia: a retrospective longitudinal study | Subject |
|  | S. Amon; M. Aikins; H. Haghparast-Bidgoli; I. A. Kretchy; D. K. Arhinful et al. | 2024 | Household economic burden of type-2 diabetes and hypertension comorbidity care in urban-poor Ghana: a mixed methods study | Included |
|  | B. A. Andargie; E. B. Lealem; D. A. Angaw | 2024 | Trend, spatial distribution, and factors associated with HIV testing uptake among pregnant women in Ethiopia, based on 2005-2016 Ethiopia demographic and health survey: A multivariate decomposition analysis and geographically weighted regression | Subject |
|  | K. Anderson; H. Rabie; B. S. Eley; L. Frigati; J. Nuttall et al. | 2025 | Mother-child dyads living with HIV in the Western Cape, South Africa: Undetectable = Undetectable? | Subject |
|  | C. C. Anikwe; O. S. Umeononihu; I. H. Anikwe; A. C. Ikeotuonye; C. C. Ikeohaet al. | 2024 | The birth satisfaction scale: Igbo adaptation, validation, and reliability study | Subject |
|  | S. Anjorin; B. Nabatte; S. Mpooya; B. Tinkitina; C. K. Opio; N. B. Kabatereine; G. F. Chami | 2024 | Epidemiology of periportal fibrosis and relevance of current Schistosoma mansoni infection within the context of repeated mass drug administration in rural Uganda: a population-based, cross-sectional study | Subject |
|  | R. T. Annose; A. M. Nur; A. Z. Tsige; L. H. Juhar; A. G. Zegergsh | 2024 | Hepatitis B vaccination status among patients with end-stage kidney disease on haemodialysis in Ethiopia: a multi-center cross-sectional study | Subject |
|  | A. B. Anteneh; Z. G. Asfaw | 2025 | Time to recovery of COVID-19 patients and its predictors: a retrospective cohort study in HUCSH, Sidama, Ethiopia | Subject |
|  | A. B. Anteneh; M. LeBlanc; A. A. Natnael; Z. G. Asfaw | 2024 | Survival of hospitalised COVID-19 patients in Hawassa, Ethiopia: a cohort study | Subject |
|  | N. Anyaegbunam; Q. E. Igabari; E. O. Egbule; I. B. Agbigwe; K. R. Ede et al. | 2024 | Stress management strategy of Education Scientists with high blood pressure: A case for multivariate analysis and implications for school management system | Subject |
|  | R. Apondi; H. Bastiaens; C. Nöstlinger; J. Galbraith; T. M. Aholou et al. | 2025 | Community and familial dynamics influencing risk behavior for HIV acquisition among adolescent girls and young women in Uganda: Qualitative analysis using Protective Motivation Theory | Subject |
|  | C. Apprey; H. Y. Addae; M. Osei; I. Danquah; R. Annan | 2025 | Dietary diversity and opportunistic infections among adults living with human immunodeficiency virus on antiretroviral therapy in Kumasi metropolis; a facility-based cross-sectional study | Subject |
|  | A. Apreku; C. Guure; S. Dery; A. Yakubu; G. R. Abu-Ba'are; S. A. Addo; K. Torpey | 2025 | Awareness, willingness, and uptake of pre-exposure prophylaxis (PrEP) among men who have sex with men in Ghana | Subject |
|  | D. F. Apuulison; B. Nabawanuka; M. Muhoozi; J. Aryampa; P. Irumba et al. | 2025 | Enablers of and barriers to ART adherence among female sex workers in mid-western Uganda: a qualitative study | Subject |
|  | M. Ardehali; C. Kafu; M. Vazquez Sanchez; M. Wilson-Barthes; B. Mosong et al. | 2024 | Food insecurity is associated with greater difficulty accessing care among people living with HIV with or without comorbid non-communicable diseases in western Kenya | Subject |
|  | M. Arefaine; A. Johannessen; T. Teklehaymanot; A. Mihret; D. H. Alemayehu et al. | 2024 | A prospective, multicenter study of hepatitis B birth-dose vaccine with or without hepatitis B immunoglobulin in preventing mother-to-child transmission of hepatitis B virus in Ethiopia | Subject |
|  | B. Arega; M. Mengistu; A. Mersha; A. Agunie | 2024 | Evaluation of hospital quality of care outcomes in a teaching hospital in Ethiopia: a retrospective database study | Subject |
|  | G. S. Argaw; K. A. Gelaye; A. M. Lakew; F. M. Aragaw; Y. M. Chekol etal. | 2024 | Survival and predictors of mortality among HIV-infected adults after initiation of antiretroviral therapy in Eastern Ethiopia Governmental hospitals, from January 2015 to December 2021 (multi-center retrospective follow-up study) | Subject |
|  | M. Armstrong-Hough; T. Shelby; J. Ggita; J. Nangendo; A. J. Gupta; J. L. Davis; A. Katamba | 2024 | An adapted scale to measure perceived TB and HIV stigma during household contact investigation | Subject |
|  | S. Arunga; K. E. Morley; T. Kwaga; M. G. Morley; L. F. Nakayama et al. | 2024 | Assessment of Clinical Metadata on the Accuracy of Retinal Fundus Image Labels in Diabetic Retinopathy in Uganda: Case-Crossover Study Using the Multimodal Database of Retinal Images in Africa | Subject |
|  | H. E. Aschmann; A. Musinguzi; J. L. Kadota; C. Namale; J. Kakeeto et al. | 2024 | Preferences of people living with HIV for features of tuberculosis preventive treatment regimens in Uganda: a discrete choice experiment | Subject |
|  | J. M. Asingwire; I. Isiko; K. F. Rombe; A. Mwesigwa; E. A. Ikwara et al. | 2025 | Prevalence and determinants of HIV testing-seeking behaviors among women of reproductive age in Tanzania: analysis of the 2022 Demographic and health survey | Subject |
|  | H. Assefa; T. Ali; I. Mussa; T. Misgana; D. Abdi; A. Zewudie; A. Temesgen | 2025 | Common mental disorders and associated factors among adult patients admitted in non-psychiatric wards of public hospitals in Harari regional State, Eastern Ethiopia | Subject |
|  | H. S. Aswani; W. Mdluli; A. Khathi | 2025 | A Retrospective Analysis of the Changes in Prediabetes-Associated Markers of Thyroid Function in Patients from Durban, South Africa | Subject |
|  | K. Atkins; D. A. Walker; K. Noon; C. Nwakama; A. Snyder et al | 2025 | Recent Incarceration and HIV Risk Among Women Who Use Heroin | Subject |
|  | B. Atuhaire; L. Muteebwa; R. Nabunya; R. Muhindo; T. D. Ngabirano et al. | 2024 | Brief Communication: Factors associated with willingness to use long-acting injectable Cabotegravir for HIV pre-exposure prophylaxis (PrEP) among female undergraduate students at a Ugandan university | Subject |
|  | C. Atuhaire; K. Taseera; E. C. Atukunda; D. Atwine; L. T. Matthews; G. Z. Rukundo | 2025 | Prevalence of postpartum depression and its association with diabetes mellitus among mothers in public health facilities in Mbarara, Southwestern Uganda | Subject |
|  | C. Atuhairwe; L. Atuhaire; S. O. Wandera; D. Amongin; T. Ochieng; C. Misinde | 2025 | Predictors of survival among older adults with HIV in Uganda's AIDS support organization centers of excellence (1987-2023): a retrospective longitudinal study | Subject |
|  | R. Atwine; M. Yekosani; A. Birungi; B. Ssenkumba; B. Tuhamize; R. Ezinga; K. Male; T. Kabanda | 2025 | Spectrum and trends of cancer among HIV patients in Southwestern Uganda | Subject |
|  | O. A. Awosoga; O. O. Oyewole; O. M. Adegoke; A. C. Odole; O. K. Onyeso et al. | 2024 | Cardiovascular disease risk perception among community-dwelling adults in southwest Nigeria: A mixed-method study | Subject |
|  | T. W. Ayalew; K. D. Gashu; A. T. Jemere; N. D. Baykemagn | 2025 | Intention to use mobile phone-based TB screening among HIV patients in Debre Tabor Town public health facilities, Northwest Ethiopia: a cross-sectional study | Subject |
|  | M. Ayele; B. D. Tilahun; G. Yilak; A. Alamrew; A. Zewdie; E. S. Lake | 2024 | Prevalence of failed induction of labor and associated factors among women who underwent induction of labor in Ethiopia: A systematic review and meta-analysis | Subject |
|  | B. Ayenew; P. Kumar; A. Hussein | 2024 | Incidence and predictors of unplanned 30-day hospital readmissions among heart failure patients in Ethiopia: a 5-year retrospective cohort study | Subject |
|  | I. N. Azia; A. Nyembezi; S. Carelse; F. C. Mukumbang | 2025 | Beliefs of Pentecostal pastors on the use of antiretroviral treatment among Pentecostal Christians living with HIV in a suburb of Cape Town-South Africa: a community health systems lens | Subject |
[truncated: 195,865 more chars]
