# Supplementary material for: Self-management of chronic conditions including multimorbidity in sub-Saharan Africa: A systematic and meta-synthesis review with focus on diabetes, hypertension, chronic kidney disease, and HIV
Source: PLOS Glob Public Health. 2025 Oct 9;5(10):e0003836. doi: 10.1371/journal.pgph.0003836 (PMC12510608; doi:10.1371/journal.pgph.0003836)
Supplement: S5 Table — (DOCX) [file pgph.0003836.s005.docx]

**S5 Table: Full paper screening**

|  | **Authors** | **Year** | **Title** | **Decision** |
| --- | --- | --- | --- | --- |
|  | V. Mogre, N. A. Johnson, F. Tzelepis and C. Paul | 2019 | Barriers to diabetic self-care: A qualitative study of patients' and healthcare providers' perspectives | Population |
|  | K. Hjelm and G. Nambozi | 2008 | Beliefs about health and illness: a comparison between Ugandan men and women living with diabetes mellitus | Methods |
|  | K. Adjaye-Gbewonyo; I. A. Kretchy; L. Baatiema; C. S. Grijalva-Eternod; O. A. Sanuade et al. | 2025 | Non-communicable diseases, psychosocial wellbeing, and quality of life in Ga Mashie, Accra, Ghana: analysis from a community-based cross-sectional study | Methods |
|  | E. Adjei-Mensah; Y. Alhassan; R. Owusu; I. A. Kretchy | 2025 | Determinants of antiretroviral therapy adherence among people living with HIV in a poor urban setting in Ghana: a hospital-based cross-sectional study | Methods |
|  | W. Adraro; G. Abeshu; F. Abamecha | 2024 | Physical and psychological impact of HIV/AIDS toward youths in Southwest Ethiopia: a phenomenological study | Subject |
|  | O. Agede; O. Daramola; A. Joseph; M. Jimoh; S. Ibrahim et al. | 2025 | Quality of hypertension management and health insurance impact: an assessment of insured and uninsured patients with systemic hypertension in a teaching Hospital in Ilorin, Nigeria | Subject |
|  | A. T. Ahmed; M. Abduku; A. A. Aliyi; A. Tahir | 2024 | Knowledge about diabetic retinopathy, eye check-up service utilisation and associated factors among adult diabetic patients at public hospitals of southeastern Ethiopia, 2022 | Methods |
|  | S. K. Alor; I. A. Kretchy; F. N. Glozah; P. B. Adongo | 2024 | Community beliefs and practices about diabetes and their implications for the prevention and management of diabetes in Southeast Ghana | Included |
|  | J. S. Ambikile; S. S. Ngulupi; A. F. Massae | 2025 | Factors associated with chronic kidney disease knowledge and preventive practices: An analytical cross-sectional study among patients with hypertension at Amana Regional Referral Hospital in Dar es Salaam, Tanzania | Methods |
|  | S. Amon; M. Aikins; H. Haghparast-Bidgoli; I. A. Kretchy et al. | 2024 | Household economic burden of type-2 diabetes and hypertension comorbidity care in urban-poor Ghana: a mixed methods study | Included |
|  | M. Ardehali; C. Kafu; M. Vazquez Sanchez; M. Wilson-Barthes; B. Mosong et al. | 2024 | Food insecurity is associated with greater difficulty accessing care among people living with HIV with or without comorbid non-communicable diseases in western Kenya | Subject |
|  | A. Belete; G. Teshale; A. Yalew; E. Delie; G. Getu; A. Atnafu | 2024 | Adherence of healthcare providers to Enhanced Adherence Counseling (EAC) intervention protocol in West Amhara Public Health Facilities, Northwest Ethiopia, 2023: mixed method evaluation | Subject |
|  | G. K. Belete; H. L. Sithole | 2025 | Prevalence and associated factors of diabetes among adult populations of Hawassa town, southern Ethiopia: A community based cross-sectional study | Subject |
|  | N. K. Belete; T. F. Gadore; D. T. Assefa; M. Y. Teshale; M. B. Sorrie; E. Z. Tariku | 2025 | Association between poor drug adherence and undernutrition among adult HIV patients in southern Ethiopia: an institution based cross sectional study | Subject |
|  | V. Boima; C. Hayfron-Benjamin; A. Doku; A. A. A. Twumasi; D. Ottie-Boakye et al. | 2024 | Detection, linkage to care, treatment and monitoring of hypertension in coastal communities in Accra, Ghana: protocol for a quasi-experimental study (The Ghana Heart Initiative Hypertension Study) | Protocol |
|  | K. Elzorkany; M. A. Alhamad; B. M. Albaqshi; M. Y. Alhassan; M. H. Alahmed et al. | 2024 | Knowledge about peritoneal dialysis among patients with end-stage kidney disease on hemodialysis: a cross-sectional study | Subject |
|  | E. E. Endrias; T. Geta; B. Desalegn; B. A. Ataro; E. Israel et al. | 2024 | Exploring experiences and perspectives of patients on hypertension management in Southern Ethiopia: a phenomenological study | Included |
|  | M. Galvin; L. Coetzee; P. Leshabana; N. Masebe; S. Lebepe et al. | 2024 | Perceptions of HIV and mental illness as Western" or "Traditional" illnesses: a cross-sectional study from Limpopo Province, South Africa" | Methods |
|  | A. W. Garedo; G. T. Tesfaye; R. Tamrat; E. Wynendaele | 2024 | Glycemic control and associated factors in patients with type 2 diabetes in Southwest Ethiopia: a prospective observational study | Methods |
|  | H. Mokoena; S. E. Mabhida; J. Choshi; M. D. Sekgala; B. B. Nkambule et al. | 2024 | Soluble P-selectin as an inflammatory mediator potentially influencing endothelial activation in people living with HIV in sub-rural areas of Limpopo, South Africa | Subject |
|  | H. Mulugeta Abate; P. Kumar; S. Anteneah; M. Girma; W. Yimam; B. Desu | 2025 | Health literacy and associated factors among adult type 2 diabetic patients in Woldia Comprehensive Specialized Hospital, North-East Ethiopia, 2022 | Methods |
|  | Y. M. Negussie; M. Sento; N. M. Fati | 2024 | Diabetic microvascular complications among adults with type 2 diabetes in Adama, central Ethiopia | Subject |
|  | E. S. Okello; R. N. Peck; B. Issarow; G. Kisigo; K. Abel et al. | 2025 | Ashamed of being seen in an HIV clinic": a qualitative analysis of barriers to engaging in HIV care from the perspectives of patients and healthcare workers in the Daraja clinical trial" | Methods |
|  | E. O. Owolabi; A. I. Ajayi | 2024 | Adherence to medication, dietary and physical activity recommendations: Findings from a multicenter cross-sectional study among adults with diabetes in rural South Africa | Methods |
|  | R. Owusu; S. A. Bawua; E. B. Kwarteng; L. Baatiema; J. Nonvignon | 2024 | A qualitative exploration of policy interventions to improve the health-related quality of life of people living with HIV AIDS and co-morbidities of hypertension and/or diabetes in Ghana | Subject |
|  | A. Rashid; D. Ejara; H. A. Deybasso | 2024 | Adherence to antihypertensive medications and associated factors in patients with hypertension, Oromia, Ethiopia: a multicenter study | Subject |
|  | M. Sibazo; T. Sookan-Kassie | 2024 | Knowledge, Attitudes, and Practice Regarding Physical Exercise in Type 2 Diabetic and Non-Diabetic Staff at a Tertiary Institution | Subject |
|  | T. M. Swambulu; Y. S. Mundedi; Y. L. Nsimbi; F. L. Bompeka; A. N. Natuhoyila et al. | 2024 | Masked uncontrolled hypertension among elderly black sub-saharan africans compared to younger adults: a cross-sectional in-hospital study | Subject |
|  | K. Tani; B. Osetinsky; G. Mhalu; S. Mtenga; G. Fink; F. Tediosi | 2024 | Seeking and receiving hypertension and diabetes mellitus care in Tanzania | Methods |
|  | N. Tarantino; B. Norman; A. Enimil; S. Osei Asibey; C. Martyn-Dickens; K. Guthrie; A. Kwara; B. Bock; M. J. Mimiaga; L. Brown | 2024 | HIV symptom severity and associated factors among young people with HIV in Ghana | Population |
|  | E. T. Tarekegn; M. Y. Gobezie; M. B. Haile; A. A. Zerga | 2025 | Glycemic control and associated factors among type 2 diabetes patients attending at Dessie comprehensive specialized hospital outpatient department | Methods |
|  | G. K. Yirga; G. S. Mekonen; E. G. Hiruy; K. Shiferaw; B. Bantie | 2024 | Non-adherence to appointment follow-up and its associated factors among hypertensive patients in follow-up clinics in South Gondar hospitals | Methods |
|  | R. BeLue, M. Diaw, F. Ndao, T. Okoror, A. Degboe and B. Abiero | 2012 | A cultural lens to understanding daily experiences with type 2 diabetes self-management among clinic patients in M'bour, Senegal | included |
|  | M. J. Rotheram-Borus, M. Tomlinson, M. Gwegwe, W. S. Comulada, N. Kaufman and M. Keim | 2012 | Diabetes buddies: peer support through a mobile phone buddy system | Subject |
|  | P. Matwa, M. M. Chabeli, M. Muller and N. S. Levitt | 2003 | Experiences and guidelines for footcare practices of patients with diabetes mellitus | Included |
|  | E. Mendenhall and S. A. Norris | 20152 | Diabetes care among urban women in Soweto, South Africa: a qualitative study | Subject |
|  | J. O. Adeleye, N. O. Agada, W. O. Balogun, O. R. Adetunji and H. O. Onyegbutulem | 2006 | Diabetes care in Nigeria: time for a paradigm shift | Subject |
|  | Awah | 2009 | Diabetes mellitus: indigenous naming, indigenous diagnosis and self-management in an African setting: the example from Cameroon |  |
|  | E. F. Chikumbu, C. Bunn, S. Kasenda, A. Dube, E. Phiri-Makwakwa et al. | 2022 | Experiences of multimorbidity in urban and rural Malawi: an interview study of burdens of treatment and lack of treatment | Included |
|  | N. B. D. Magobe, M. Poggenpoel and C. Myburgh | 2017 | Experiences of patients with hypertension at primary health care in facilitating own lifestyle change of regular physical exercise | Included |
|  | M.A. Pienaar and M.A. Reed | 2021 | A diabetes peer support intervention: Patient experiences using the Mmogo-metho | Population |
|  | A. d. G. Aikins, R. B. Awuah, T. A. Pera, M. Mendez and G. Ogedegbe | 2015 | Explanatory models of diabetes in urban poor communities in Accra, Ghana | Included |
|  | A. de-Graft Aikins, R. B. Awuah, T. A. Pera, M. Mendez and G. Ogedegbe | 2015 | Explanatory models of diabetes in urban poor communities in Accra, Ghana | Included |
|  | M. S. Abdulrehman, W. Woith, S. Jenkins, S. Kossman and G. L. Hunter | 2014 | Exploring Cultural Influences of Self-Management of Diabetes in Coastal Kenya: An Ethnography | Included |
|  | A. d. G. Aikins | 2005 | Healer shopping in Africa: new evidence from rural-urban qualitative study of Ghanaian diabetes experiences | Included |
|  | C. Moucheraud, K. Phiri and R. M. Hoffman | 2022 | Health behaviours and beliefs among Malawian adults taking antihypertensive medication and antiretroviral therapy: a qualitative study | Included |
|  | L. Drown; A. J. Adler; L. N. Schwartz; J. Sichali; F. Valeta et al. | 2023 | Living with type 1 diabetes in Neno, Malawi: a qualitative study of self-management and experiences in care | Included |
|  | H. Amu, E. K. M. Darteh, E. E. Tarkang and A. Kumi-Kyereme | 2021 | Management of chronic non-communicable diseases in Ghana: a qualitative study using the chronic care model | Included |
|  | T. Steyl and J. Phillips | 2014 | Management of type 2 diabetes mellitus: adherence challenges in environments of low socio-economic status | Included |
|  | V. Angwenyi, C. Aantjes, M. Kajumi, J. De Man, B. Criel et el. | 2018 | Patients experiences of self-management and strategies for dealing with chronic conditions in rural Malawi | Included |
|  | E. N. Bosire | 2021 | Patients' Experiences of Comorbid HIV/AIDS and Diabetes Care and Management in Soweto, South Africa | Included |
|  | G. Mphwanthe, M. Carolan, D. Earnesty and L. Weatherspoon | 2021 | Perceived barriers and facilitators to diet and physical activity among adults diagnosed with type 2 diabetes in Malawi | Included |
|  | A. I. Okurumeh, O. A. Akpor, O. E. Okeya and O. B. Akpor | 2022 | Type 2 diabetes mellitus patients' lived experience at a tertiary hospital in Ekiti State, Nigeria | Included |
|  | P. Bleah, R. Wilson, D. Macdonald and P. Camargo Plazas | 2023 | “When I Don’t Have Money, I Don’t Eat”: A Critical Hermeneutic Study of Diabetes in Liberia | Included |
|  | P. Bleah, R. Wilson, D. Macdonald and P. Camargo-Plazas | 2023 | ‘The solution is we need to have a centre’: a study on diabetes in Liberia | Included |
|  | S. Tyabazeka, W. Phiri and R. R. Marie Modeste | 2024 | HIV self-management perceptions and experiences of students at one university in South Africa | Included |
|  | B. O. Ukoha-Kalu; M. O. Adibe; C. V. Ukwe | 2023 | A qualitative study of patients’ and carers’ perspectives on factors influencing access to hypertension care and compliance with treatment in Nigeria | Included |
|  | Masupe at al. | 2022 | Diabetes self-management: a qualitative study on challenges and solutions from the perspective of South African patients and health care providers | Population |
|  | Adeniyi et al. | 2015 | Diabetic patients’ perspectives on the challenges of glycaemic control | Population |
|  | Mafunda et al. | 2025 | Differences in Health and Illness Beliefs in Zimbabwean Men and Women with Diabetes | Population |
|  | Mwila et al. | 2019 | Experiences and challenges of adults living with type 2 diabetes mellitus presenting at the University Teaching Hospital in Lusaka, Zambia | Methods |
|  | Lynch et al. | 2019 | Exploring patient experiences with and attitudes towards hypertension at a private hospital in Uganda: a qualitative study | Methods |
|  | Areri | 2020 | Exploring Self-Management of Adults Living with HIV on Antiretroviral Therapy in North-West Ethiopia: Qualitative Study | Methods |
|  | Hushie | 2019 | Exploring the barriers and facilitators of dietary self-care for type 2 diabetes: a qualitative study in Ghan | Methods |
|  | Abrajhams, Gilson | 2019 | Factors that influence patient empowerment in inpatient chronic care: early thoughts on a diabetes care intervention in South Africa | Methods |
|  | Russel et al. | 2016 | Finding meaning: HIV self-management and wellbeing among people taking antiretroviral therapy in Uganda | Methods |
|  | Mamatsharaga et al. | 2020 | I lack ‘me-time’: The experiences of family caregivers of elders with Diabetes Mellitus in a selected village in South Africa | Methods |
|  | Tuha | 2021 | Knowledge and Practice on Diabetic Foot Self-Care and Associated Factors Among Diabetic Patients at Dessie Referral Hospital, Northeast Ethiopia: Mixed Method | Methods |
|  | Owolabi et al. | 2022 | Knowledge, attitude and perception towards lower limb amputation amongst persons living with diabetes in rural South Africa: A qualitative study | Population |
|  | Tokwe | 2020 | Knowledge, attitude and perception towards lower limb amputation amongst persons living with diabetes in rural South Africa: A qualitative study | Methods |
|  | Russel | 2019 | Men's Refashioning of Masculine Identities in Uganda and Their Self-Management of HIV Treatment | Methods |
|  | Oppoku- Adai | 2022 | Nutritional self-care practices and skills of patients with diabetes mellitus: A study at a tertiary hospital in Ghana | Methods |
|  | Skovdal et al. | 2020 | Parental obligations, care and HIV treatment: How care for others motivates self-care in Zimbabwe | Methods |
|  | Bossman et al. | 2021 | Patients’ knowledge of diabetes foot complications and self-management practices in Ghana: A phenomenological study | Methods |
|  | Omasakin | 2019 | A qualitative study of SM needs of people living with AIDS/HIV in rural KwaZulu Natal, Southern Africa | Methods |
|  | Murphy et al. | 2015 | A qualitative study of the experiences of care and motivation for effective self-management among diabetic and hypertensive patients attending public sector primary health care services in South Africa | Population |
|  | Matima et al. | 2018 | A qualitative study on the experiences and perspectives of public sector patients in Cape Town in managing the workload of demands of HIV and type 2 diabetes multimorbidity | Methods |
|  | Masupe et al. | 2018 | Redefining diabetes and the concept of self-management from a patient's perspective: implications for disease risk factor management | Methods |
|  | Bogale et al. | 2022 | Self-care practice, lived experience of type 1 diabetes mellitus patients at Kemisse General Hospital, North Eastern Ethiopia: Phenomenological study | Methods |
|  | Tewahido | 2017 | Self-Care Practices among Diabetes Patients in Addis Ababa: A Qualitative Study | Methods |
|  | Dube et al. | 2017 | Self-Management Support Needs of Patients with Chronic Diseases in a South African Township: A Qualitative Study | Methods |
|  | Hjelm et al. | 2008 | Zimbabwean diabetics' beliefs about health and illness: an interview study | Methods |
|  | De Klerk et al. | 2012 | “A Body Like a Baby”: Social Self-Care among Older People with Chronic HIV in Mombasa | Methods |
|  | M. J. Rotheram-Borus et al. 2012 | 2012 | Diabetes buddies: peer support through a mobile phone buddy system | Subject |
|  | A. Adjei; K. T. C. Brightson; M. M. Mensah; J. Osei; M. Drah et el. | 2024 | Determinants of glycemic control among persons living with type 2 diabetes mellitus attending a district hospital in Ghana | Methods |
|  | E. Adjei-Mensah; Y. Alhassan; R. Owusu; I. A. Kretchy | 2025 | Determinants of antiretroviral therapy adherence among people living with HIV in a poor urban setting in Ghana: a hospital-based cross-sectional study | Methods |
|  | T. O. Afolaranmi; B. Chaplin; A. I. Zoakah; P. J. Kanki | 2025 | HIV-related stigma among young men who have sex with men in HIV care in Plateau State Nigeria | Methods |
|  | S. Alem; H. Gulema | 2024 | Intention to use short messaging services for promoting drug adherence among individuals with diabetes in Addis Ababa, Ethiopia | Subject |
|  | S. Lee; R. Nantale; S. Wani; S. Kasibante; A. Marvin Kanyike | 2024 | Influence of women's decision-making autonomy and partner support on adherence to the 8 antenatal care contact model in Eastern Uganda: A multicenter cross-sectional study | Subject |
|  | M. Leone; L. Giani; M. Mwazangati; D. Uluduz; T. Şaşmaz et al. | 2024 | Cost of transport is a barrier to access to headache care in sub-Saharan Africa: An observational study in an HIV-positive population | Methods |
|  | E. T. Tarekegn; M. Y. Gobezie; M. B. Haile; A. A. Zerga | 2025 | Glycemic control and associated factors among type 2 diabetes patients attending at Dessie comprehensive specialized hospital outpatient department | Methods |
|  | N. S. West; W. Ddaaki; S. M. Murray; N. Nakyanjo; D. Isabirye et al. | 2024 | Someone who hates themself doesn't come for their drugs": Experiences of mental health along the HIV care continuum in South-Central, Uganda" | Subject |
|  | S. A. Wondm; T. K. Zeleke; S. B. Dagnew; T. A. Moges; G. Y. Tarekegn ei. Al. | 2024 | Association between self-care activities and glycemic control among patients with type 2 diabetes mellitus in Northwest Ethiopia general hospitals : a multicenter cross-sectional study | Methods |
|  | A. D. Worku; A. W. Gessese | 2024 | Uncontrolled hypertension among adult hypertensive patients in Addis Ababa public hospitals: A cross-sectional study of prevalence and associated factors | Methods |
|  | G. K. Yirga; B. Bantie; E. G. Hiruy; A. A. Baye; G. Kerebeh et al. | 2024 | Health checkup practice and its associated factors among adults in South Gondar zone Ethiopia | Methods |

**Included:** Included in the review

**Excluded:** Subject, methods, population, setting, review. protocol, invalid, repetition
