# Supplementary figures and images for: Self-management of chronic conditions including multimorbidity in sub-Saharan Africa: A systematic and meta-synthesis review with focus on diabetes, hypertension, chronic kidney disease, and HIV
Source: PLOS Glob Public Health. 2025 Oct 9;5(10):e0003836. doi: 10.1371/journal.pgph.0003836 (PMC12510608; doi:10.1371/journal.pgph.0003836)

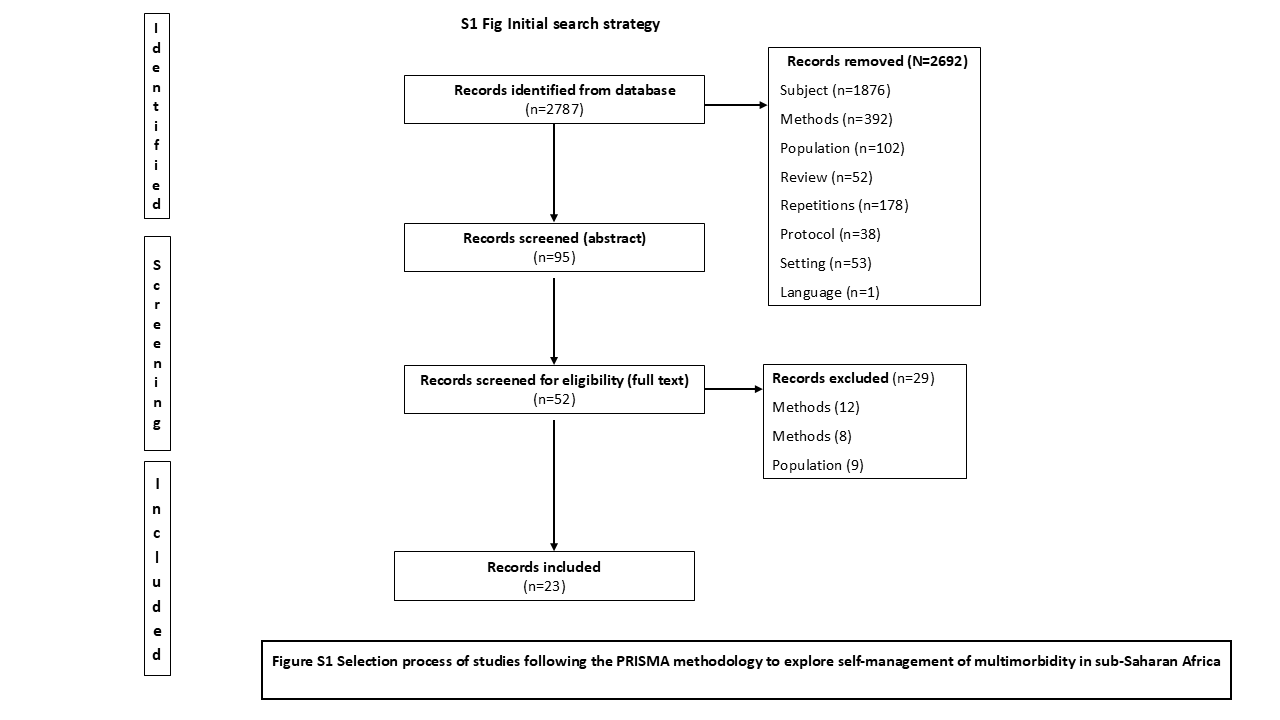

Supplement: S1 Fig — (TIF) [file pgph.0003836.s006.tif]
