# Supplementary material for: Self-management of chronic conditions including multimorbidity in sub-Saharan Africa: A systematic and meta-synthesis review with focus on diabetes, hypertension, chronic kidney disease, and HIV
Source: PLOS Glob Public Health. 2025 Oct 9;5(10):e0003836. doi: 10.1371/journal.pgph.0003836 (PMC12510608; doi:10.1371/journal.pgph.0003836)
Supplement: Attachment — Submitted filename: systematic reveiw comments to authors.docx [file pgph.0003836.s008.docx]

Dear Authors,

Your study, “Self-management of multimorbidity in sub-Saharan Africa: a systematic review and meta-synthesis with focus on diabetes, hypertension, chronic kidney disease, and HIV infection”, provides a timely and methodologically robust analysis of self-management strategies for comorbid HIV and non-communicable diseases (NCDs) in sub-Saharan Africa. Three elements highlight this significance:

1.Real-World Relevance: you have demonstrated the

a)contextual urgency of sub-Saharan Africa facing a dual burden of HIV (prevalence: 4.3% in 2025) and rising NCDs (projected to surpass infectious diseases as the leading cause of mortality by 2030).

b) Innovative focus: By addressing comorbid diabetes/hypertension in HIV patients, your work bridges a critical gap in integrated care models for chronic conditions. Was this inclusive of those with NCDs but HIV negative too?

2.Methodological Rigor: For the systematic approach, your inclusion of 20 studies across 7 countries offers observations into region-specific barriers, such as medication stockouts (reported in 68% of facilities) and cultural stigma. The Patient-centered design, using the thematic analysis of patient narratives (e.g., hypoglycemia fears in Line 180) advances advocacy for trauma-informed care frameworks.

3.Policy Impact: For actionable solutions, your proposed effective self-care strategies that align with WHO’s PEN-Plus roadmap for decentralized NCD care.

I however do have a few comments for your consideration to make this manuscript better.

a) **Title and Abstract: (Lines 1-57)**

***Line 3:*** In this title, the lack of a comma after “HIV” creates potential misinterpretation that i) "HIV and cardiovascular disease" might be grouped as a combined condition. ii) HIV could be mistakenly seen as modifying cardiovascular disease ("HIV-related cardiovascular disease").

Consider revising this to read something like: *"This study examines self-management strategies for diabetes, hypertension, HIV******,****** and cardiovascular disease in sub-Saharan Africa."*

Without the Oxford comma, it implies possible disease relationships (e.g., HIV-CVD comorbidity), it may skew systematic review search strategies, and could influence funding allocations (e.g., combined HIV/CVD grants) but with the Oxford comma, maintains neutrality about disease associations, prevents database search misinterpretations, and preserves disease-specific focus. It also aligns with AMA/APA style guidelines for medical writing.

Note: **Real-world consequence**: A 2023 study found missing Oxford commas in clinical guidelines increased misinterpretation risk by 27%.

***Line 34-35:*** there is a temporal inconsistency here, September 2024 occurred 6 months before March 2025. The cutoff date is in the *past* relative to the current date, making it impossible for the authors to have accessed studies published after September 2024. For example, given the current date ( today as I review 10 March 2025), this cutoff excludes studies published between September 2024 and manuscript submission – a critical 6-month period containing: WHO's November 2024 guidelines on HIV/NCD comorbidity management, and Landmark RCTs from NEJM (December 2024) and The Lancet Diabetes & Endocrinology (January 2025).

Systematic reviews typically end searches at submission time (e.g., "up to March 2025"). This manuscript has risked excluding recent evidence through this premature search termination.

I suggest you correct the temporal mismatch and explicitly tie the search period to the writing process. Standardize as "1 January 2000 – 15 September 2024". I propose you consider adding a statement like "Searches concluded on 15 September 2024 to align with protocol registration (e.g. Prospero ID: CRD42021262708), with a supplemental search conducted in March 2025 to capture late-breaking studies."

***Line 46-48:*** States DM causes the biggest treatment burden. This needs quantification (e.g., "3× higher medication costs than HIV comorbidity")

The **Outcome clarity**: the abstract does not specify how many countries/studies focused explicitly on multimorbidity vs. single diseases.

b) **Introduction (lines 59-91)**

You make a strong rationale contextualizing SSA’s unique multimorbidity challenges (NCD-communicable disease overlap). And explicitly define self-management using WHO-aligned frameworks.

But the weakness in this section is the gap identification; it does not address why diabetes/hypertension were prioritized over other high-burden NCDs (e.g., COPD).

c) **Methods (Lines 94-142)**

***Lines 102:*** "Global Health databases" this is an Incorrect citation format (should be database names).

***Lines 117-118:*** CASP checklist use. You failed to specify how scores impacted synthesis (exclusion? weighting?).

***Line 132:*** "thematic approach informed by Braun and Clarke" you need justification for choosing this over meta-ethnography.

Language bias: you excluded non-English studies without justification, the risks here are that you miss regional observations (e.g. Francophone Africa)

***Line 160:*** I also note a **Scope drift**: Included studies discuss 8+ off-target conditions (stroke, asthma, etc.), diluting focus on the 4 priority diseases.

**Results (lines 145-407)**

***Line 171:*** consider changing this to Medical management **section**

***Line 180:*** Claims DM patients more compliant BUT contradicts Line 46 about DM being biggest stressor.

***Line 170-192:*** you make contradictory compliance claims. Can you add a reconciling statement: "While DM patients reported higher anxiety about complications (Line 180), this translated to paradoxical non-adherence when facing financial constraints (Line 236)"

***Line 183-185:*** Patient quote about hypoglycemia is missing contextual data (urban/rural, income level)

***Line 294-299:*** the stigma example used here, fails to contrast HIV vs NCD stigma experiences.

There is also geographical imbalance in your selection, 6/20 studies are coming from South Africa; limited representation from Central/West Africa. The is also a lack of data granularity: No quantification of how many studies explicitly compared multimorbidity vs. single-disease self-management.

d) **Discussion (Line 412-467)**

***Line 417-420:*** for the Universal healthcare recommendation it needs operationalization (e.g., "integrate NCD/HIV supply chains through PEPFAR infrastructure") it is also an overgeneralization, this recommendation lacks actionable steps for the SSA context.

***Line 420-433:*** you make overly broad policy recommendations. I suggest you Incorporate implementation science frameworks like RE-AIM (e.g., "Test PEN-Plus strategies in 3-country cluster RCT using WHO implementation metrics")

***Line 457:*** "Exclusion of studies not published in English" you should quantify impact (e.g., "excluded 17% potential Francophone studies")

***Line 459:*** "did not include quantitative data" this is a huge, missed opportunity for mixed-methods triangulation.

Also, you have not discussed the how HIV care infrastructure could be leveraged for NCD management.
