# Supplementary material for: Self-management of chronic conditions including multimorbidity in sub-Saharan Africa: A systematic and meta-synthesis review with focus on diabetes, hypertension, chronic kidney disease, and HIV
Source: PLOS Glob Public Health. 2025 Oct 9;5(10):e0003836. doi: 10.1371/journal.pgph.0003836 (PMC12510608; doi:10.1371/journal.pgph.0003836)
Supplement: Attachment — Submitted filename: Ressponse to editor.odt [file pgph.0003836.s010.odt]

20th April 2025
Dear Editor,
Thank you for your feedback on our manuscript titled "Self-management of multimorbidity in sub-Saharan Africa: a systematic review and meta-synthesis with focus on diabetes, hypertension, chronic kidney disease and HIV infection." PGPH-D-24-02249R1

We have addressed the data availability requirement as follows:
1. As required by our policy on Data Availability, please ensure your manuscript or supplementary information includes the following:

a) A numbered table of all 2787 studies identified in the literature search, including those that were excluded from the analyses.
Response: We have provided a table of all 2,787 studies identified through our literature search has been prepared and is attached as Supplementary Information. We have indicated papers that were included and reasons for exclusion for the rest.

i). For every excluded study, the table should list the reason(s) for exclusion.
ii). If any of the included studies are unpublished, include a link (URL) to the primary source or detailed information about how the content can be accessed.
Response:
i). We have also pulled out the 95 studies that underwent full-text review and provided a separate table which includes detailed reasons for exclusion.
ii). All papers included were published and publicly accessible. No unpublished studies were included.
2. Please amend your online detailed Financial Disclosure statement. This is published with the article. It must therefore be completed in full sentences and contain the exact wording you wish to be published.

a) State the initials, alongside each funding source, of each author to receive each grant, if applicable. For example: "This work was supported by the National Institutes of Health (####### to AM; ###### to CJ) and the National Science Foundation (###### to AM)."
Response: We have amended and added more names as they apply.

b) State what role the funders took in the study. If the funders had no role in your study, please state: “The funders had no role in study design, data collection and analysis, decision to publish, or preparation of the manuscript.”
Response: We have added a statement that details the role of the funder.

3. Please ensure that the funders and grant numbers match between the Financial Disclosure field and the Funding Information tab in your submission form. Note that the funders must be provided in the same order in both places as well.
Response: We confirm that the funder and grant number have been matched. For information’s sake, we have one funder.

4. Please update your online Competing Interests statement. If you have no competing interests to declare, please state: “The authors have declared that no competing interests exist.”
Response: We have amended the response as per your recommendations.

5. We notice that your revision was submitted on April 11, 2025, but the manuscript file in your submission's file inventory was uploaded on October 16, 2024. Please upload the latest version of your revised manuscript as the main article file, with the item type 'Manuscript,' ensuring that it does not contain any tracked changes or highlighting. This will be used in the production process if your manuscript is accepted.
Response: We have uploaded the latest version of the revised manuscript as requested. The file does not contain any tracked changes. 

6. Please provide separate main figure files in .tif or .eps format only and ensure that all files are under our size limit of 10MB.
Response: We have converted Figure S1 to TIF format and is provided as a separate file.

7. Please include a separate legend or caption for Figure 1 in your manuscript.
Response: We have included a separate legend for Figure 1, as requested.

8. Please ensure that you refer to Figure 1 in your text as, if accepted, production will need this reference to link the reader to the figure.
Response: We have ensured that Figure 1 is now appropriately referenced in the main text to facilitate linking during production

9. Please add a full list of legends for all your Supporting Information files after the References list.
Response: We have added a full list of legends for all Supporting Information files after the References section in the manuscript, as requested.

We trust this fulfills your comments, and we are happy to provide any further clarification if needed.
Kind regards,

Sangwani Nkhana Salimu
