# Supplementary material for: Self-management of chronic conditions including multimorbidity in sub-Saharan Africa: A systematic and meta-synthesis review with focus on diabetes, hypertension, chronic kidney disease, and HIV
Source: PLOS Glob Public Health. 2025 Oct 9;5(10):e0003836. doi: 10.1371/journal.pgph.0003836 (PMC12510608; doi:10.1371/journal.pgph.0003836)
Supplement: Attachment — Submitted filename: Response to reviewers.docx [file pgph.0003836.s011.docx]

**Response to reviewers**

Dear Dr Ivan Namakoola,

Thank you for your email and for the opportunity to revise our manuscript submitted to *PLOS Global Public Health*. We appreciate the thoughtful feedback provided by the reviewers and the editorial team. We have carefully considered all comments and revised the manuscript accordingly as indicated below:

**Comment 1:** The issue of language bias justification; the exclusion of non-English studies lacks rationale, risking underrepresentation of Francophone Africa (17% SSA population). I strongly suggest you add a rationale (e.g., "English-language studies represent 89% of accessible qualitative research on this topic in SSA").

**Response- Page 20:** We have acknowledged the exclusion of non-English articles as a limitation which may may introduce potential bias and limit the generalizability findings to non-English speaking contexts. We have made recommendations for future work to include non-English papers.

**Comment 2:** The CASP scoring method wasn’t properly justified, which affects comparability with other reviews. Your CASP tool adaptation using non-standard 0-1 scoring instead of CASPs yes/no/can’t tell, obscures quality assessment transparency. Can you justify the deviation (e.g., "Adapted CASP scoring to enhance granularity in appraising cultural context validity").

**Response- Page 7:** Thank you for your comment. In response, we have revised the scoring approach to reflect Yes/Average/Unclear ratings. We now use *Yes* for studies that clearly met the appraisal criteria, *Average* for those where some criteria were unmet, and *Unclear* where reporting was insufficient or ambiguous. This adjustment aligns more closely with the original CASP framework while allowing us to maintain clarity and consistency in assessing study quality. The revised description has been added to the manuscript.

**Comment 3:** There is still overrepresentation of South Africa/Malawi (6/23 studies) vs Central/West Africa (1 study). I suggest that the authors acknowledge this limitation: "Findings may not fully generalize to Francophone/West African contexts."

**Response- (Page 20):** Thank you for raising this issue. We acknowledge the uneven geographic distribution of included studies, with a concentration in Southern and parts of Eastern Africa. We have added a statement to the manuscript noting that this may limit the transferability of findings to underrepresented regions, particularly Central, West, and Francophone Africa. This limitation has now been clearly acknowledged in the revised discussion.

**Comment 4:** Quantitative data exclusion: this is a missed opportunity, you have ignored quantitative insights from the included mixed-methods studies. In the discussion, the authors excluded mixed-methods data from included studies. This creates a lack of triangulation (e.g., medication adherence rates vs. qualitative narratives). I suggest the authors add something like "Future reviews should integrate quantitative data from mixed-methods studies." Or integrate adherence rates (e.g., "42% of hypertensive patients rationed medications monthly") to triangulate qualitative themes.

**Response- Page 20:** We acknowledge this limitation and have now added a statement in the discussion recommending that future reviews integrate quantitative data from mixed-methods studies to enable triangulation and strengthen insights.

**Comment 5:** The scope drift improved a bit but was not eliminated. The authors now clarify that "3 studies on multimorbidity vs. 20 on single diseases" The remaining issue is: Includes 8+ off-target conditions (stroke, asthma) without stratification. Consider adding a table stratifying the findings by target vs incidental conditions.

**Response- Page 8 of manuscript and Pages 3-8 Supplemental Materials:** Thank you for this helpful comment. We acknowledge the concern regarding the inclusion of off-target conditions. *Table S2: Summary of included studies* (pages 3–8 of the supplementary materials) provides a detailed overview of all included studies, indicating whether they focus on multimorbidity, single conditions of interest (CKD, DM, HTN, HIV), or incidental conditions such as stroke or asthma.

We have ensured that all participant quotes and findings presented in the main synthesis relate specifically to multimorbidity or the primary conditions of interest. However, where off-target conditions are mentioned, they are included to illustrate the complexity and interconnectedness of multimorbidity, rather than to highlight those conditions in isolation. For example, the quote on page 12 comes from a participant living with HIV, HTN, DM, and arthritis, but the issues discussed specifically relate to HIV, HTN, and DM—three of the conditions within our defined scope. We have now clarified this approach in the methods section of the manuscript

**Comment 6:** On stigma contrast, the authors have described HIV stigma, but this lacks a comparison to NCD stigma. Quantify differences: "HIV stigma prevalence (62%) exceeded NCD-related stigma (28%) in included studies."

**Response- Pages 14-15:** Thank you. We have now qualitatively distinguished HIV stigma from NCD-related stigma by highlighting their different social meanings.

**Comment 7:** There still exists a compliance paradox. Unresolved tension between DM patients’ "heightened fear of complications" and "medication rationing due to costs" consider adding "Financial constraints drove DM patients to prioritize acute symptom management over long-term adherence." OR "While DM patients reported heightened fear of complications, financial constraints drove rationing behaviors, creating a compliance–affordability tension."

**Response- Page 19:** We agree that this paradox is an important insight, and in fact, it emerged inductively during our data synthesis. We have now made this tension more explicit in the discussion, highlighting the contrast between patient expressed fear of diabetes complications and their need to ration medication due to financial constraints. This has been added in the discussion to clarify how this finding contributes to current understanding.

**Comment 8:** For implementation frameworks, the authors could propose evaluation metrics, like "Test PEN-Plus using RE-AIM (Reach: 80% clinic coverage; Effectiveness: HbA1c reduction ≥1%)."

**Response- Page 21:** We have incorporated a brief recommendation to evaluate future multimorbidity programs in SSA using the RE-AIM framework, highlighting its relevance for assessing impact and sustainability. This has been added to the discussion section in relation to supporting self-management and patient-centred care for PLWMM.

We thank you for your thoughtful and constructive feedback. Your suggestions have helped us to clarify key arguments, address important nuances in patient experiences, and strengthen the practical implications of our findings. We sincerely appreciate your contributions to improving the quality and relevance of our manuscript, and we hope our revisions meet your favourable considerations for publication with PLOS Global Public Health.

Sincerely,

Sangwani Nkhana Salimu
