# Supplementary material for: Self-management of chronic conditions including multimorbidity in sub-Saharan Africa: A systematic and meta-synthesis review with focus on diabetes, hypertension, chronic kidney disease, and HIV
Source: PLOS Glob Public Health. 2025 Oct 9;5(10):e0003836. doi: 10.1371/journal.pgph.0003836 (PMC12510608; doi:10.1371/journal.pgph.0003836)
Supplement: Attachment — Submitted filename: Response letter to reviewers.docx [file pgph.0003836.s012.docx]

Dear Reviewer,

We are grateful for the time and effort you have taken to provide constructive feedback on our manuscript, *“Self-management of chronic conditions including multimorbidity in sub-Saharan Africa: a systematic review and meta-synthesis with focus on diabetes, hypertension, chronic kidney disease, and HIV infection.”* Your comments have helped us clarify and improve the manuscript.

We address your comments as follows:

**Comment 1:** *Clarification on focus on multimorbidity versus single conditions*

**Response**

**(Line 2):** We have revised our title to reflect the methods and now reads **‘Self-management of chronic conditions including multimorbidity in sub-Saharan Africa: a systematic review and meta-synthesis with focus on diabetes, hypertension, chronic kidney disease, and HIV infection.’**

We have made revisions in the following sections:

**Abstract**

**(Lines 30-32):** We now explicitly describe that for studies on single conditions, we extracted first- and second-order constructs that were pertinent to multimorbidity, ensuring that the synthesis included both single-condition and multimorbidity perspectives.

**(Lines 35-38):** We state that we used thematic synthesis approach and explicitly describe that for studies on single conditions, we extracted first- and second-order constructs that were pertinent to multimorbidity, ensuring that the synthesis included both single-condition and multimorbidity perspectives.

**(Lines 61-68):** We have revised the results and discussion sections have been revised to clearly indicate that the findings represent a synthesis of experiences from both single conditions and multimorbidity, and we explicitly describe which findings relate to multimorbidity. This ensures the population of interest is clearly defined and that our conclusions appropriately reflect the evidence.

**(Lines 105-111):** We have clarified in the introduction that, although the review was initially designed to capture qualitative evidence on multimorbidity, the available literature was limited and most studies focused on single conditions. Therefore, we included studies on each condition individually (HIV, diabetes, hypertension, CKD) while explicitly extracting and synthesizing insights relevant to multimorbidity wherever possible. This approach ensures that our findings reflect both single-condition experiences and issues pertinent to people living with multimorbidity (PLWMM).

**(Lines 466-472):** We summarise the conditions included in the study and the population to which our findings apply.

**Comment 2:** *Correction of methodological reference*

**(Lines 156-163):** We have corrected all references to the thematic synthesis method to cite Thomas and Harden (2008) instead of Braun and Clarke.

The Methods section has been updated to clearly explain the three stages of thematic synthesis (line-by-line coding, development of descriptive themes, and generation of analytical themes) and the role of the first author in conducting the coding and synthesis.

**Comment 3:** *Repetition and missing files*

**(Line 178):** We have removed the repetition regarding the PRISMA 2020 checklist.

**Supplemental files S4, S5, and Figure 1 (PRISMA flow diagram)** have been included in the revised submission for review.

We believe these revisions address your concerns and improve clarity regarding the scope, methodological approach, and population focus of the manuscript. We thank you again for your careful review and constructive suggestions.

Sincerely,

Sangwani Nkhana-Salimu
